# Terpene-Derived Carvyl Acetate Dicarbonate: A Novel Monomer for Biobased Nonisocyanate Poly(hydroxyurethane) Thermoplastics and Thermosets

**DOI:** 10.1021/acs.biomac.6c01553

**Published:** 2026-08-10

**Authors:** Marc Martínez de Sarasa Buchaca, Jesús Naranjo, Alberto Moreno-Fernández, Carmen Moya-López, Pablo Salcedo-Abraira, Antonio Rodríguez-Diéguez, Carlos Gracia-Fernández, Antonio M. Rodríguez-García, José A. Castro-Osma, Felipe de la Cruz-Martínez, Agustín Lara-Sánchez

**Affiliations:** † 16733 Universidad de Castilla-La Mancha, Departamento de Química Inorgánica, Orgánica y Bioquímica-Centro de Innovación en Química Avanzada (ORFEO−CINQA), Facultad de Ciencias y Tecnologías Químicas and Instituto Regional de Investigación Científica Aplicada-IRICA, 13071 Ciudad Real, Spain; ‡ Departamento de Química Inorgánica, Orgánica y Bioquímica-Centro de Innovación en Química Avanzada (ORFEO−CINQA), Facultad de Farmacia, Universidad de Castilla-La Mancha, 02071 Albacete, Spain; § Departamento de Química Inorgánica, Facultad de Ciencias, 16741 Universidad de Granada, 18071 Granada, Spain; ∥ TA Instruments Waters Chromatography, Tres Cantos, 28760 Madrid, Spain

## Abstract

New thermoplastic nonisocyanate poly(hydroxyurethanes) (NIPUs) have been designed by polyaddition reactions of carvyl acetate dicarbonate (CADC) with a range of commercial diamines, yielding thermoplastic nonisocyanate poly(hydroxyurethanes) with moderate molecular weights and glass transition temperatures (*T*_g_) ranging from −30.5 to 62 °C. Thermal curing of the Jeffamine-derived poly(hydroxyurethane) with the polyacrylate compound LAROMER LR 9000 resulted in the formation of sticky thermoset materials, showing potential for adhesive applications. In addition, curing of bis(glydycyl ether dicarbonate) (BGDC)/CADC blends with polyethylene imine (PEI) enables the formation of tailor-made NIPU thermosets with high stiffness, tensile strength, and low elongation at break.

## Graphical abstract

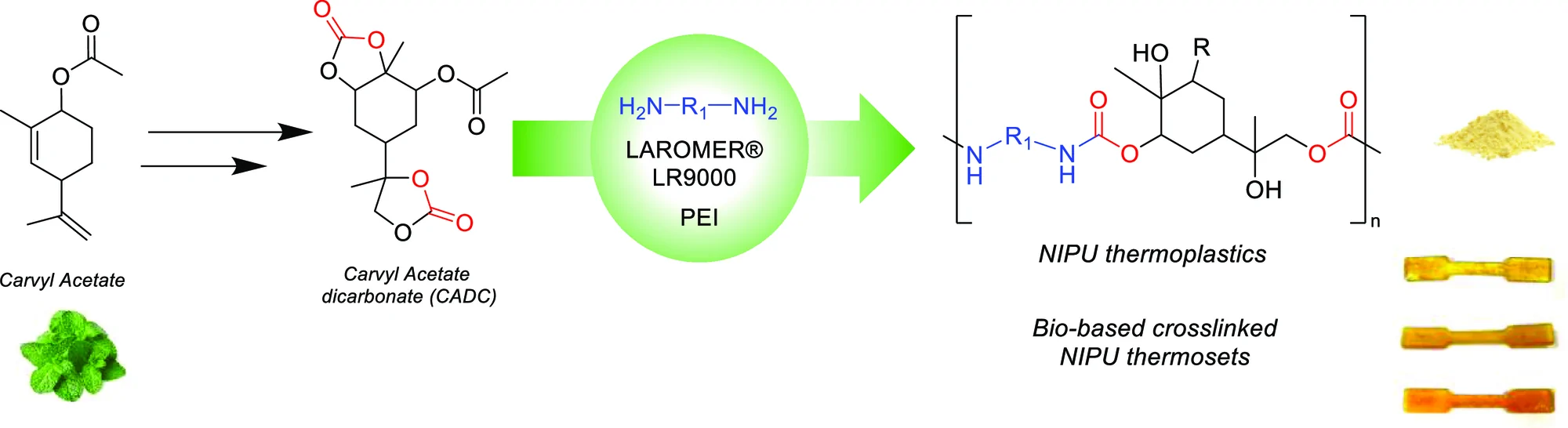

## Introduction

Polyurethanes (PUs) are versatile polymers widely employed across various industries given their chemical, mechanical, and physical properties, including abrasion resistance, durability, low weight, and high tensile strength.^1–6^ Owing to these desirable characteristics, global demand for PU materials continues to rise, with production exceeding 22 million tons worldwide in 2024 alone.^7^ Currently, PUs are extensively utilized in consumer products such as adhesives, foams, fibers, paints, varnishes, and sealants, among others.^8–11^

Traditionally, PUs have been synthesized through the polyaddition reaction between poly(isocyanates) and polyols. Despite the maturity and efficiency of this method, it relies on isocyanate precursors that are typically produced via the reaction of amines with highly toxic phosgene gas.^12–14^ Additionally, the limited availability of fossil resources presents a long-term sustainability challenge. Moreover, the production of polyols is also largely dependent on petroleum-derived chemicals, with benzene, propylene, and ethylene being the most commonly used feedstocks.^15^ These environmental and health concerns have prompted research efforts toward the development of greener alternatives, including the use of biobased intermediates and more sustainable synthetic routes for PU production.^2,16,17^

In this regard, the synthesis of nonisocyanate polyurethanes (NIPUs) via the polyaddition reaction between di- or multifunctional cyclic carbonates and diamines has received significant attention and research interest.^18–20^ The resulting NIPUs are often referred to as polyhydroxyurethanes (PHUs), due to the ring-opening of the cyclic carbonate moieties during the reaction, which generates pendant primary and secondary hydroxyl groups. These hydroxyl functionalities not only enhance the physical and chemical properties of the resulting polymers but also enable further postfunctionalization of the polymer backbone, broadening their potential applications.^21^

Despite the ongoing interest for the development of PHUs, the cyclic carbonate/amine route suffers from the lack of renewable, biosourced monomers, restraining the access to fully green and biobased PUs. Nevertheless, various research groups have reported the successful synthesis of PHUs using biobased cyclic carbonates as alternatives to their petroleum-derived counterparts.^22,23^ These efforts include the use of fatty acids and vegetable oils, as well as naturally occurring molecules such as furan, lignin, d-mannitol, and sorbitol (Figure 1).^24–32^ Lignin has recently garnered significant attention as a promising green alternative for the synthesis of cyclic carbonates, owing to its aromatic backbone and abundant hydroxyl functionalities, which make it a suitable precursor for nonisocyanate polyurethanes.^33,34^ However, the direct use of bulk lignin often results in materials with inconsistent and unpredictable properties, primarily due to its complex, heterogeneous structure and inherently low reactivity.^35,36^ To address these challenges, lignin can be depolymerized into smaller molecules which retain key functional groups, improving reactivity and enabling better control over the properties of the resulting NIPU materials.^36^ Vegetable oils have also been used as raw materials to produce carbonated oils and therefore they are also potential building blocks for NIPU synthesis.^37–40^ Nevertheless, the uncontrolled functionality of triglycerides restrains the control of PHUs structure and properties. Thus, the use of such poly(cyclic carbonates) can lead to thermoset PHUs when the cross-linking density is high. To overcome the ill-defined cross-linked PHUs obtained from high carbonated triglycerides, well-defined bis-carbonated vegetable-based fatty acids have emerged as an alternative for the synthesis of linear PHUs.^41^

**Figure 1:**
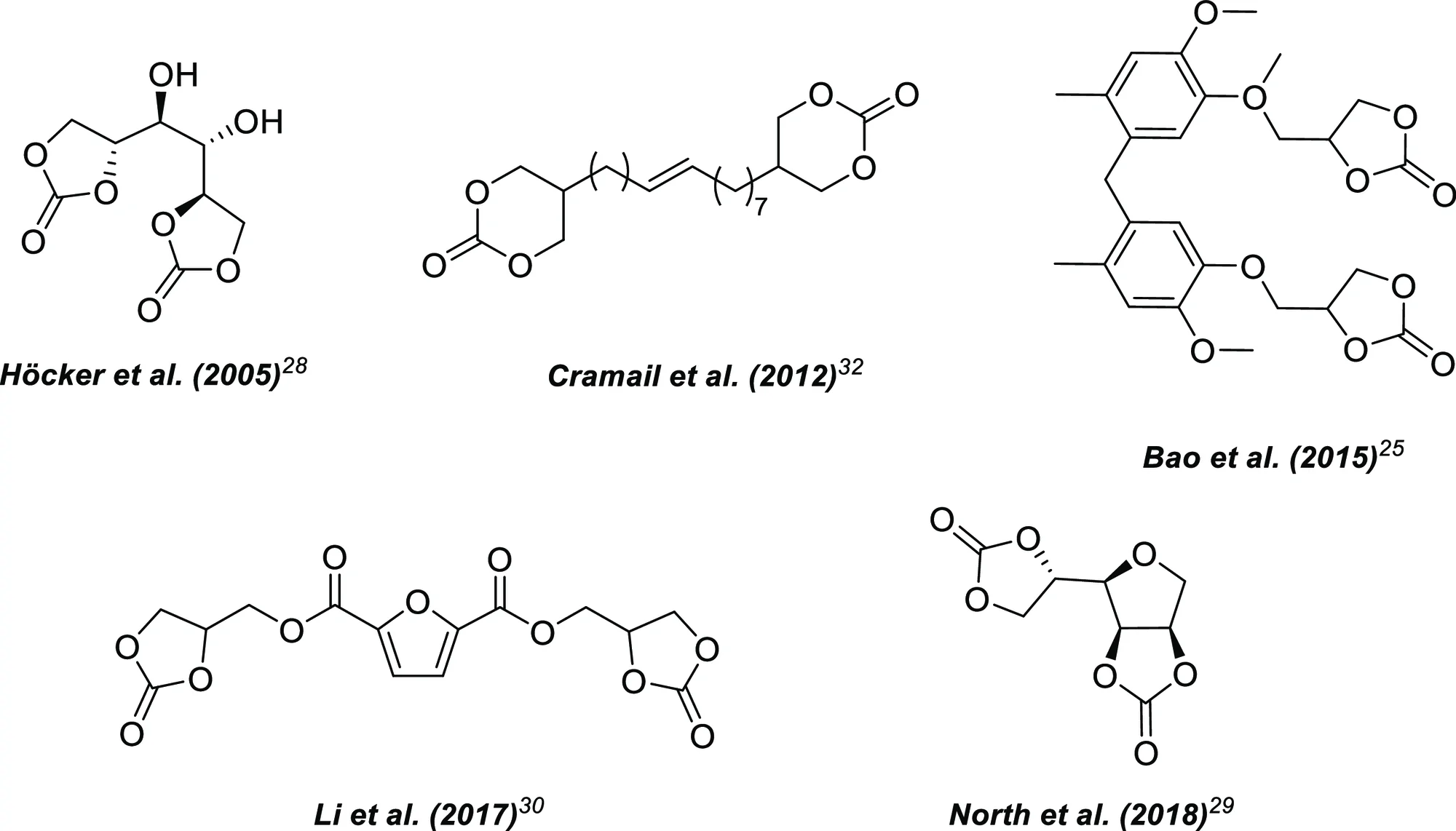
Representative biobased bis(cyclic carbonates) employed for the synthesis of NIPUs.

On the other hand, and in line with the present work, only a few studies have reported the use of terpene-based bis-cyclic carbonates as monomers for NIPU synthesis (Figure 2).^42–45^ Mulhaupt and co-workers were the first to explore this approach, employing limonene dicarbonate as a precursor for the preparation of various linear NIPUs and thermosetting materials.^42^ Shortly thereafter, the same group explored the impact of limonene dicarbonate purity on the thermal and mechanical properties of the resulting NIPUs.^43^ Interestingly, higher-purity limonene dicarbonate also reduced the intense coloration typically observed in limonene-based NIPUs, enabling, for the first time, their potential use in coating applications. In a related study, Kleij and co-workers reported the use of β-elemene dicarbonate as a renewable building block for the synthesis of NIPUs, achieving molecular weights up to 6.3 kDa and glass transition temperatures (*T*_g_) as high as 84 °C.^44^ The presence of free alkene functionalities on the oligomeric chains allowed for postpolymerization cross-linking, and the physical properties of the cured materials were evaluated for potential use as coatings. More recently, Meier and Scheelje utilized the renewable terpenes limonene and carvone to synthesize PHUs via a step-growth thiol–ene strategy.^45^ This approach leveraged the terminal double bonds of monomers obtained from the reaction of cyclic carbonate units with allylamine, yielding NIPUs with molecular weights exceeding 10 kDa under mild conditions.

**Figure 2:**
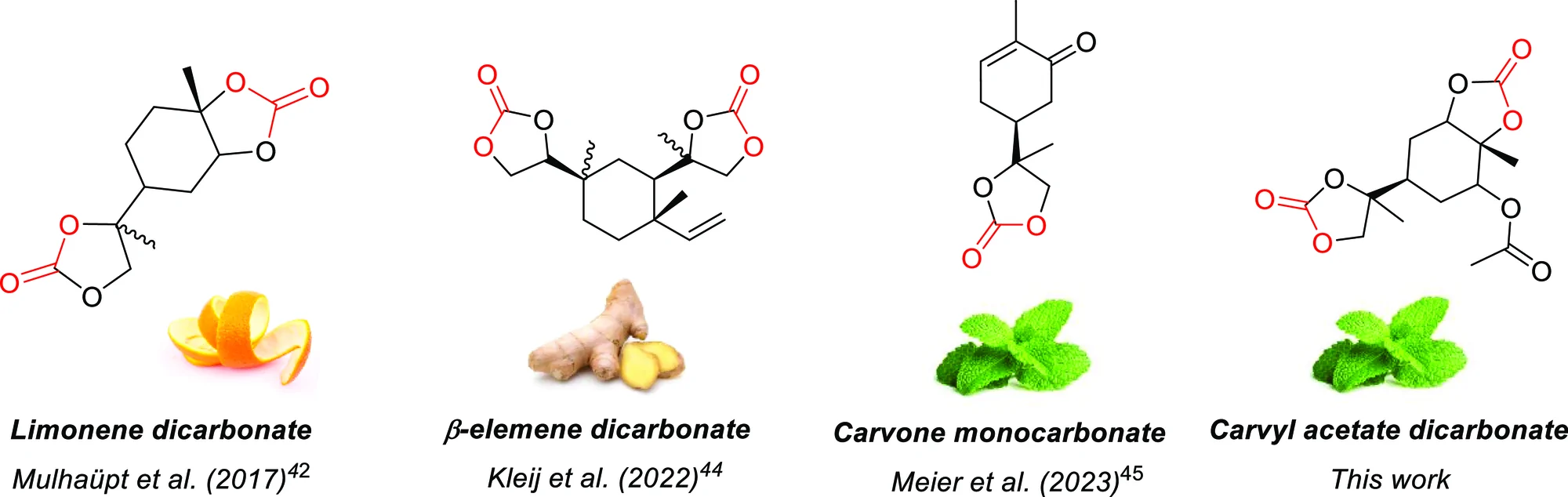
Terpene-based cyclic carbonates employed as precursors for the synthesis of NIPUs.

Herein, building upon these previous studies and given our research group’s extensive expertise in CO_2_ fixation reactions into cyclic carbonates,^46^ we report the use of carvyl acetate dicarbonate (CADC) as a novel biobased precursor for the development of PHUs. The polyaddition reaction of CADC with a diverse range of diamines afforded a series of linear nonisocyanate polyurethanes, while subsequent cross-linking yielded thermoset materials. Notably, thermal curing of the Jeffamine-derived PHU with the polyacrylate LAROMER LR 9000 produced adhesive-like thermosets and curing of CADC/bis(glycidyl ether dicarbonate) (BGDC) blends with polyethylene imine (PEI) afforded highly rigid NIPU thermosets with excellent tensile strength and low elongation at break.

## Results and Discussion

### Synthesis and Reactivity of Carvyl Acetate Dicarbonate

The multigram-scale preparation of carvyl acetate diepoxide (CADE) was achieved by standard epoxidation methods.^46^ Subsequently, solvent-free carbonation of CADE with CO_2_ was explored using various commercial halide salts, under conditions similar to those previously reported for other terpene-derived (bis)epoxides (Table S1),^43,44^ and updating the synthetic procedure previously employed by our group, in which an aluminum–metal complex was used as catalyst.^46^ Initial optimization of reaction time and temperature was performed using tetrabutylammonium bromide (TBAB) as catalyst (Table S1, entries 1–6). Under optimized conditions—120 °C for 72 h—, CADE was fully converted into a mixture of carvyl acetate monocarbonate (CAMC) and dicarbonate (CADC) in a 10:90 ratio (Table S1, entry 6), yielding to a brown viscous liquid. When CADE amount was increased to 50 g, the CADC/CAMC ratio decreased to 60:40 (Table S1, entry 11, Figure S1), likely due to the lower reactivity of the *endo*-epoxide and mass transfer limitations caused by increased viscosity of the reaction mixture. Both products were easily isolated by flash column chromatography, also needed to remove the halide catalyst, obtaining a complex mixture of CADC stereoisomers (Figure S2). Single crystals of *trans*-CADC were obtained upon placing a solution of hexane/ethyl acetate (2:1) in the freezer, enabling analysis of the CADC crystal structure by NMR spectroscopy (Figures S3–S9) and X-ray diffraction analysis (Tables S2–S4 and Figure S10). Although the reaction scale and yield for the carbonatation toward *trans*-CADC increased significantly compared to our previous report (50.0 g vs 1.0 g, 50 vs 34% respectively),^46^ the synthetic procedure is currently being optimized to minimize the carbon footprint associated with the solvents used for CADC purification and crystallization, further enhancing the sustainability of the process.

Once CADC was isolated and purified by crystallization, we studied the reactivity of *trans*-CADC by carrying out the reactions depicted in Scheme 1. For that purpose, we used 0.5 and 2 equiv of the corresponding diamines with respect to CADC to obtain hydroxycarbamates containing cyclic carbonate (Scheme 1, carbamates **1a** and **1b**) or amine end groups (Scheme 1, carbamates **2a** and **2b**). Two different amines containing alkyl (1,4-diaminobutane) and aromatic (*m*-xylene diamine) groups were tested, in order to study the influence of the amine moiety in the reactivity with *trans*-CADC. Reactions were carried out at 80 °C in MeCN during 16 h, and *trans*-CADC was quantitatively converted into the corresponding hydroxycarbamate regardless the diamine used. Depending on the diamine/*trans*-CADC ratio employed, the ring-opening of the cyclic carbonate moieties may proceed through different pathways, leading to the formation of distinct regioisomers bearing differently substituted hydroxyl groups. As illustrative examples, the possible regioisomeric structures of hydroxycarbamates **1b** and **2b** are shown in the Supporting Information (Figures S11–S12). These hydroxycarbamates not only represent a promising prepolymer building block for NIPU synthesis but also provided valuable insight into the polyaddition reaction, which will be further discussed in the next section.

**Scheme 1:**
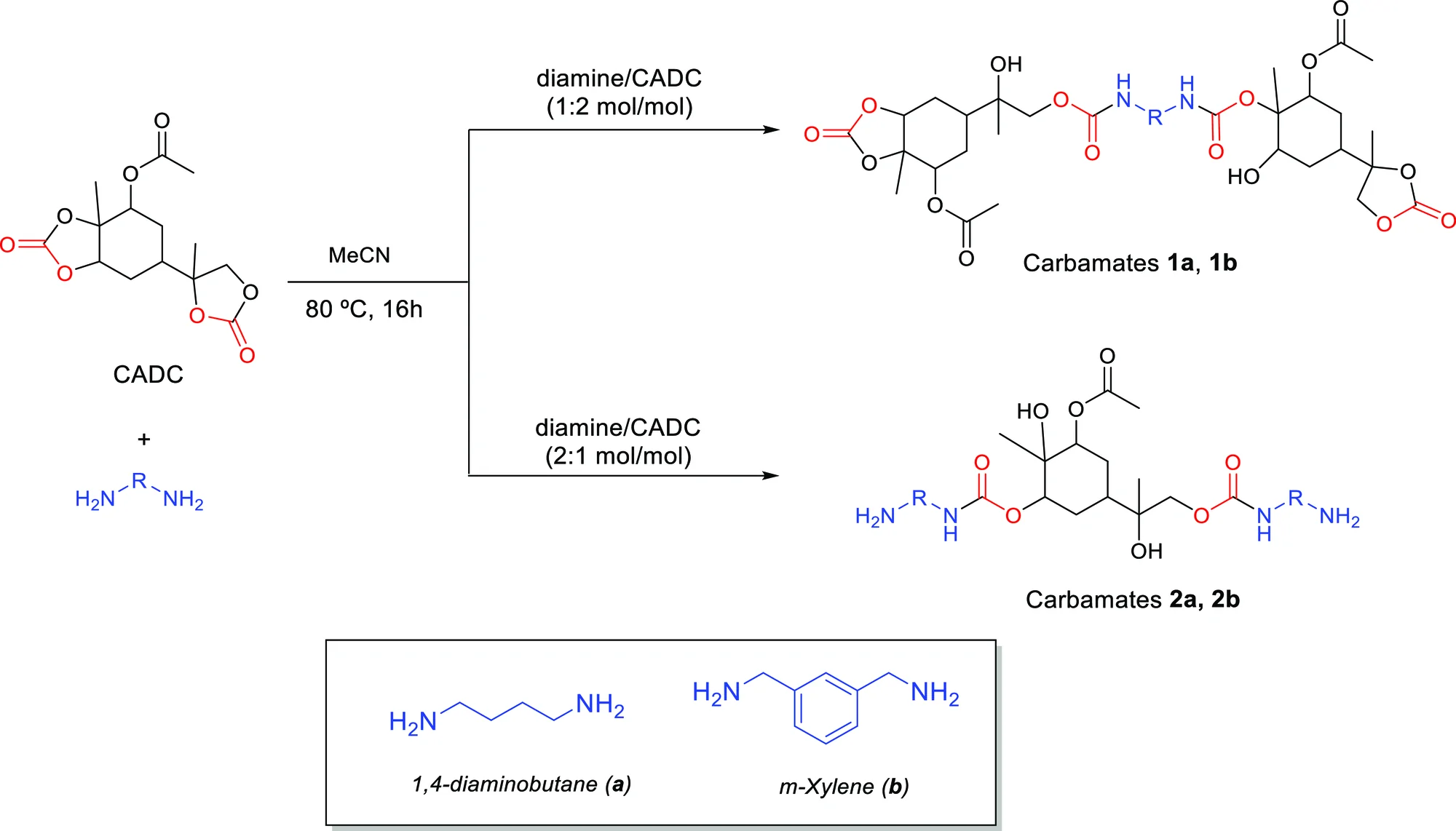
Synthesis of Hydroxycarbamates **1** and **2** (Only the Structure of the Proposed Main Regioisomer has Been Drawn for Clarity)

The formation of the carbamate moieties could be easily concluded from the hydroxycarbamates **1** and **2** IR spectra (Figures S13–S16), while comparing it with the one from *trans*-CADC. The latter displays two sharp absorption bands at 1791 and 1733 cm^–1^ corresponding to the carbonate and acetate moieties respectively (Figure S3). While hydroxycarbamates **1a** and **1b** still exhibit a band at 1794 cm^–1^ corresponding to the carbonate ending groups (Figures S13 and S14 respectively), the appearance of a new band at 1708 and 1730 cm^–1^, respectively, evidence the formation of the urethane moiety.^44^ On the other hand, hydroxycarbamates **2a** and **2b** only show the urethane absorption band at 1693 cm^–1^ (Figures S15 and S16). In order to elucidate the possible regioisomers obtained in the reactions, hydroxycarbamates **1** and **2** were also characterized by NMR spectroscopy (Figures S17–S28). The initial aim of preparing hydroxycarbamates **1** was to investigate the different reactivity between the *endo* and *exo* cyclic carbonates. Unfortunately, due to the high complexity of the NMR spectra obtained (Figures S17–S22), we could not identify the regioisomers obtained. On the other hand, NMR spectra for hydroxycarbamates **2** allowed us to detect fragments in the regioisomers obtained (Figures S23–S28). ^13^C-{^1^H}-NMR spectrum for hydroxycarbamate **2b** (Figure S26) exhibited two different signals for the C=O acetyl groups in the region around 170 ppm indicating the presence of two different regioisomers. ^13^C-{^1^H}-NMR HSQC spectrum (Figure S27) allowed us to identify the *CH* methine and *CH*_2_ methylene protons of the *endo*/*exo*-cylic carbonate moieties respectively, and both showed correspondence with the C=O urethane moieties in the ^13^C-{^1^H}-NMR HMBC spectrum (Figure S28), suggesting the presence of an *endo*-tertiary alcohol and *exo*-tertiary alcohol fragments in the regioisomers obtained.

### Preparation of Linear NIPU Thermoplastics

Next, the polyaddition reaction between *trans*-CADC with various diamines was investigated. Initial experiments were carried out at 80 °C for 16 h and several solvents were tested to optimize the reaction medium, including green solvents such as 2-methyltetrahydrofuran (2-MeTHF). However, quantitative conversions were only achieved in dimethylformamide (DMF) and acetonitrile. Given the high boiling point and toxicity of DMF, acetonitrile was selected as the optimal solvent for the polymerization reactions, in line with our previous reports.^47^ A range of commercially available diamines with diverse structural and chemical properties were tested (Scheme 2).

**Scheme 2:**
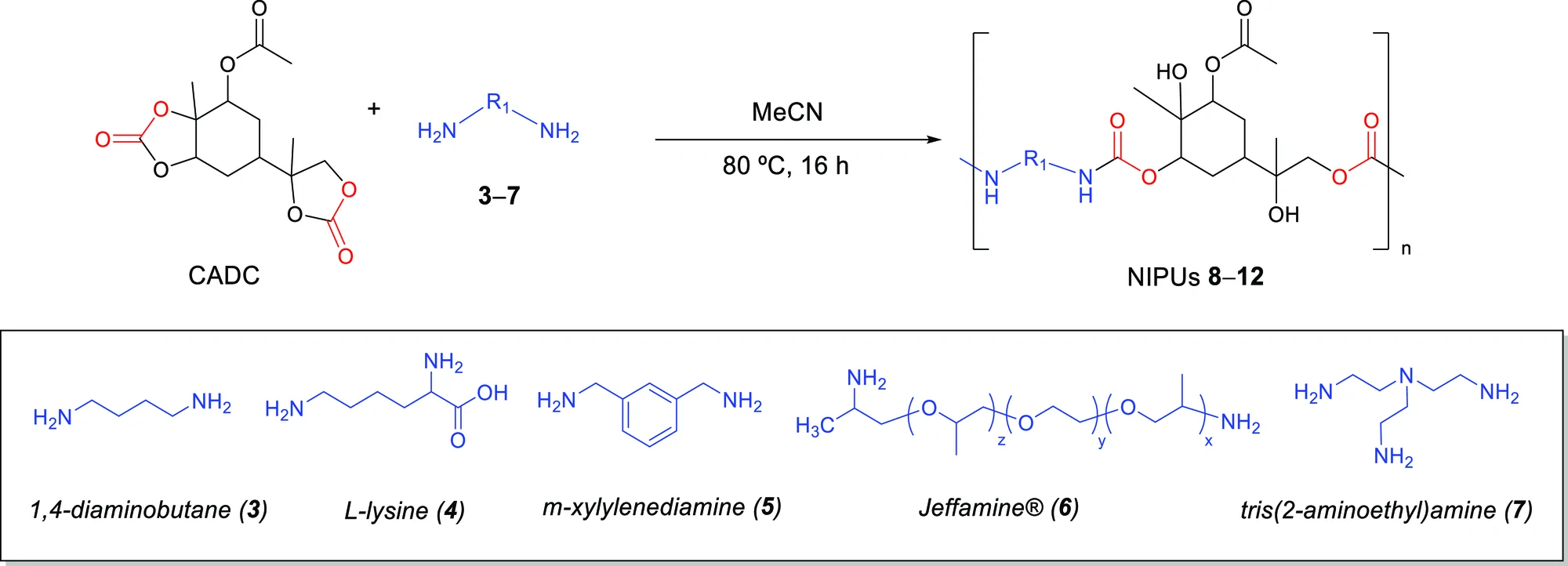
Synthesis of CADC-Based Poly(hydroxyurethanes) NIPUs **8**–**12** Using Diamines **3**–**7**

Thus, bioderived amines such as 1,4-diaminobutane (BDA) and l-lysine were employed to obtain fully bioderived NIPUs (Table 1, entries 1 and 3 respectively). Recent reports have demonstrated a strong correlation between the sterocomplexity of the bis(cyclic carbonate) starting material and the PHUs properties.^48^ Thus, to investigate the influence of the stereochemical complexity of CADC on NIPU formation, the reaction with BDA was also performed with the uncrystallized CADC mixture obtained after column chromatography (Table 1, entry 2). The presence of aromatic rigidity was explored employing *m*-xylylenediamine (Table 1, entry 4). The effect of incorporating flexible long-chain polyether segments was also evaluated using Jeffamine. Under the specified conditions above, NIPU **11** was achieved in a low conversion. Thus, we decided to perform the polyaddition reaction at neat conditions, increasing the reaction temperature and time to 120 °C and 72 h, respectively (Table 1, entry 5), following previously reported results.^44^ Finally, tris(2-aminoethyl)amine, containing a pendant primary amine group, was tested to promote cross-linking and obtain a networked NIPU structure (Table 1, entry 6). All NIPUs **8**–**12** were obtained as pale-yellow powders, with the exception of NIPU **11**, which exhibited rubbery behavior.

**Table 1:** Reaction Conditions and Thermal Properties of NIPUs Thermoplastics **8**–**12** ^a^

entry	product	diamine	yield (%)	*M*_n_ (kg·mol^–1^)^b^	PDI	*T*_g_ (°C)^c^	*T*_d_ (°C)^d^
1	NIPU **8**	1,4-diaminobutane	84	3.1	2.1	57.1	197.8
2^e^	NIPU **8**	1,4-diaminobutane	83	-	-	49.0	180.0
3^f^	NIPU **9**	L-lysine	81	22.4	2.8	42.0	186.4
4	NIPU **10**	*m*-xylylenediamine	81	4.5	1.9	58.1	210.0
5^g^	NIPU **11**	Jeffamine	93	7.8	1.8	–30.1	310.2
6	NIPU **12**	tris(2-aminoethyl)amine	88	2.5	1.7	61.5	185.7

a Reaction conditions: 0.5 g of *trans*-CADC (1.6 mmol), diamine (1.6 mmol), MeCN, 16 h, 80 °C.

b SEC (DMAc, PMMA).

c DSC (10 K/min, second heating curve).

d Data refers to the values at 10% weight loss.

e Uncrsytallized CADC mixture obtained after column crompatography was employed.

f 1 equiv 1,5,7-triazabicyclo[4.4.0]dec-5-ene (TBD) added.

g Neat, 120 °C, 72 h.

FTIR spectroscopy was used to confirm the final structure of the NIPUs synthesized (Figures S29–S33). CADC was found to be quantitatively converted into NIPUs **8**–**12** as indicated by the almost complete disappearance of the characteristic carbonyl group absorption band at 1791 cm^–1^ (Figure S3). New peaks emerged at around 3300 cm^–1^ and 1700 cm^–1^ corresponding to the hydroxyl group and C=O stretching vibrations of the urethane linkages, respectively.^49,50^ The formation of urethane linkages was further confirmed by the appearance of N–H bending and C–N stretching bands.^49^ Further analysis of the NMR spectra supported the formation of the NIPU products. The disappearance of the cyclic carbonate signals at 5.05 ppm (*endo*-cyclic carbonate), 4.30 and 4.13 ppm (*exo*-cyclic carbonate) (Figure S4) confirmed the complete consumption of the carbonate functionalities. Concurrently, a new signal in the 6.88–7.63 ppm range corresponding to the amine moiety of the urethane linkage was observed (Figures S34–S43).

On the other hand, the polyaddition reaction between CADC and l-lysine showed no conversion whatsoever under the reaction conditions specified. This lack of reactivity was attributed to the low nucleophilicity of the amine group in the α–position relative to the carboxylic acid, which is not able to ring open the cyclic carbonate moiety of CADC. Since previous studies showed the addition of strong bases such as 1,5,7 triazabicyclo[4.4.0]dec-5-ene (TBD) catalyze the polyaddition reaction efficiently, we decided to explore its use.^51,52^ Results showed full conversion to the corresponding NIPU **9** when 1 equiv of the TBD as organocatalyst was employed (Table 1, entry 3). However, the presence of such bases can also promote the formation of polyurea fragments.^53^ This was evidenced by the appearance of characteristic urea bands in the IR spectrum, with C=O stretching and N–H bending vibrations observed at 1644 and 1551 cm^–1^, respectively (Figure S30). In addition, ^1^H NMR spectrum also showed a new signal at 6.23 ppm corresponding to the amine moiety of the urea group (Figure S36).^53^

The thermal properties of the synthesized NIPUs were evaluated by thermogravimetric analysis (TGA) and different scanning calorimetry (DSC) (Figures S44–S55). The glass transition temperatures (*T*_g_) of all NIPUs ranged between 42.0 and 61.5 °C, with the exception of NIPU **11**, which exhibited a significantly lower *T*_g_ of −30.1 °C (Figure S53). This reduction is due to the flexible polyether long-chain segments derived from Jeffamine (Table 1, entry 5). Moreover, upon heating above the *T*_g_, NIPU **11** exhibits an endothermic signal centered at 27.9 °C which corresponds to the melting of the Jeffamine segment (ca. 75 J/g), suggesting that the Jeffamine-derived polymeric chain crystallizes during the purification process of the sample. In addition, upon cooling from the melt, an exothermic signal centered at −11.8 °C exhibiting a similar enthalpy to the endothermic signal (ca. 76 J/g) confirms the crystallization of the Jeffamine segment in NIPU **11**. In contrast, thermogravimetric analysis (TGA) revealed that NIPU **11** displays the highest thermal degradation onset (*T*_d_), with a value of 310.2 °C (Figure S52). Both the values for NIPU **11** fall within the typical range observed for terpene-based NIPUs synthesized using similar diamines.^44^ The effect of the CADC fraction used for NIPU **8** synthesis—*trans*-CADC and uncrystallized CADC mixture (Table 1, entries 1 and 2 respectively)—was reflected in the *T*_g_ and thermal stability of the resulting materials. NIPU **8** derived from *trans*-CADC exhibited a slightly higher *T*_g_ and greater stability than the analogue obtained from the uncrystallized mixture (57.1 and 49.0 °C respectively), consistent with the higher crystallinity and rigidity of the *trans*-CADC precursor. NIPU **12** exhibited the highest *T*_g_ among all NIPUs, exhibiting a value of 61.5 °C (Table 1, entry 6). This result is consistent with our initial idea of designing a more highly cross-linked polymer architecture, although the increase was only modest compared with NIPUs **8** and **10,** which displayed *T*_g_ values of 57.1 and 58.1 (Table 1, entries 1 and 3 respectively). The relatively low molar masses obtained for the synthesized NIPUs, ranging from 2.5 to 7.8 kg·mol^–1^, indicate the formation of oligomeric species (Figure S56). These results are consistent with previously reported results for other terpene-derived bis(cyclic-carbonates), such as limonene and β-elemene dicarbonate, which show similar structure and reactivity.^43,44^ The SEC trace for NIPU **12** (Figure S56e) exhibits a broad shape with important polydisperse populations, probably due to the cross-linked architecture, which may lead to polymer chains with high molecular weights. On the other hand, the NIPU derived from l-lysine (NIPU **9**) displayed a notably higher molecular weight (22.4 kg·mol^–1^, Table 1, entry 3). This increase is attributed to the use of the organocatalyst (TBD), which effectively catalyzes the aminolysis reaction and promotes chain growth. The enhanced reaction kinetics may allow higher degrees of polymerization before diffusion limitations associated with the increasing viscosity of the reaction medium become significant.^54^

### Preparation of Cross-Linked NIPU Thermosets

Given the rubbery nature of NIPU **11** (Table 1, entry 5) and inspired by previous work on β-elemene cross-linking tests,^44^ we scaled up the synthesis of this NIPU to investigate its reactivity with LAROMER LR9000, a polyacrylate compound containing reactive isocyanate groups kindly provided by *Badische Anilin*-and *Soda*-*Fabrik* (BASF) (Scheme 3). Reaction from the amines of the urethane moiety and the hydroxylic pending groups of NIPU **11** with the isocyanate groups of polyacrylate LAROMER LR9000 afforded the cross-linked corresponding materials. For the curing process, NIPU **11** was first melted at 90 °C and mixed with LAROMER LR9000 for 45 s. The resulting viscous mixture was then poured into a Teflon mold (Figure S57) and cured in an oven at 100 °C for 2 h, followed by postcuring at room temperature for 24 h.

**Scheme 3:**
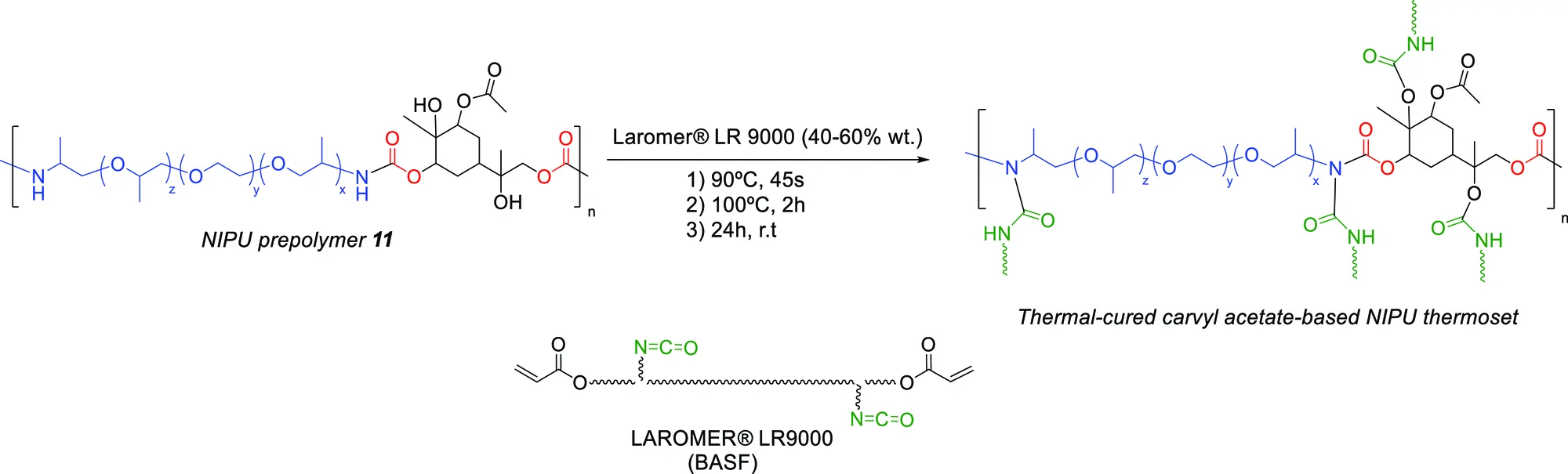
Curing of NIPU **11** with LAROMER LR9000 as Cross-Linking Agent

Several formulations were tested containing different NIPU **11**/LAROMER LR9000 weight ratios (Table 2 and Table S5). However, only **11**_LAR40 formulation resulted in a solid, dry coating suitable for mechanical testing (Table 2, entry 3; Figure 3a). The stress–strain curves (Figure S58) exhibited an S-shaped profile, which is characteristic of cross-linked elastomeric polymers. Formulations with higher LR9000 content (50 and 60 wt %) were overly sticky and could not be demolded from the Teflon mold, preventing further analysis (Table 2, entries 1 and 2), and exhibiting potential adhesive applications. The **11**_LAR40 formulation exhibited a Young’s modulus of 872 kPa, significantly lower than those of previously described for other terpene-derived NIPU thermosets.^43^ This reduced stiffness is likely attributed to the presence of the long, flexible poly(propylene glycol) chain from Jeffamine in NIPU **11**, together with the formulation based on LAROMER LR9000. This may lead to a more layered or phase-separated structure within the network and explains the thermal degradation profile found for **11**_LAR40 (Figure S59), and the lower glass transition temperature of −54.2 °C (Figure S60) compared to NIPU **11**. In addition, upon heating above the *T*_g_, **11**_LAR40 exhibits an exothermic signal centered at −26.1 °C related to the cold crystallization of the Jeffamine segment within the **11**_LAR40 sample that confirms the effective cross-linking accomplished during the curing and postcuring processes, since the reduced mobility of the polymeric chain hampers the crystallization from the melt.

**Table 2:** Thermal and Mechanical Properties of Bio-Based NIPU Thermosets Based on NIPU **11** and Cured with LAROMER LR9000^a^

entry	NIPU	LAROMER LR9000 [wt %]	*T*_g_ (°C)^b^	Young’s modulus [Kpa]	σ_B_ [Kpa]^[^ ^c^ ^]^	ε_B_ [%]^c^	EWC (%)^d^
1	**11**_LAR60	60	–	–	–	–	–
2	**11**_LAR50	50	–	–	–	–	–
3	**11**_LAR40	40	–52.6	871.5 ± 7.2	85.9 ± 7.2	9.9 ± 0.5	141.4 ± 10.6

a For more experimental details, see Supporting Information.

b Determined by different scanning calorimetry analysis with a scan rate of 10 °C min^–1^.

c Tensile testing (ISO 37); σ_B_ represents the maximum tensile strength, ε_B_ is the elongation at rupture.

d Equilibrium water content EWC (%) = [(*W*_s_ – *W*_d_)/*W*_s_] × 100.

**Figure 3:**
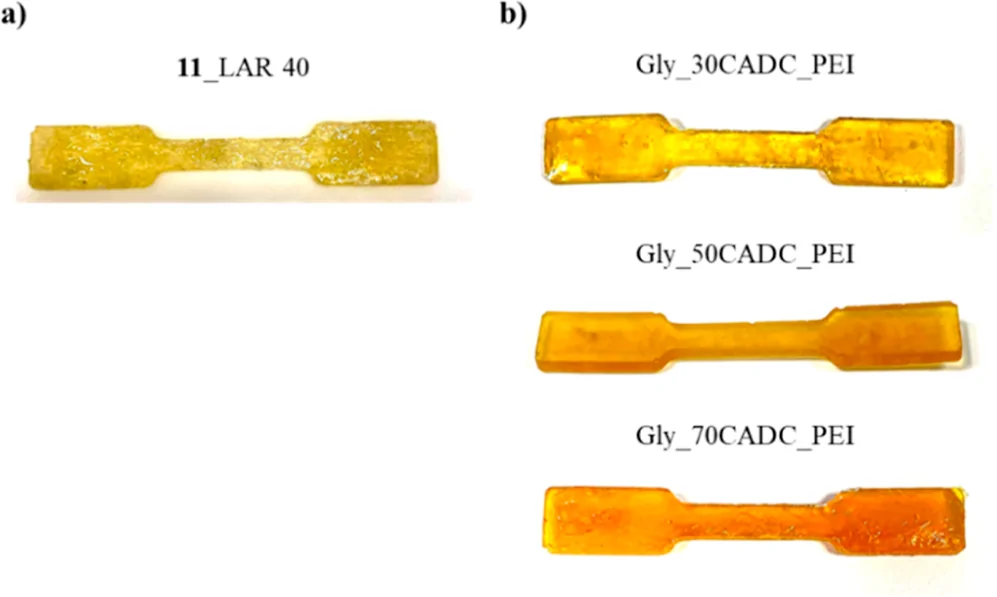
(a) **11**_LAR40 tensile test specimen; (b) Gly_CADC_PEI tensile test specimens.

To investigate the interactions between the **11**_LAR40 NIPU thermoset and water, water swelling experiments were also performed (Figure S61). For this purpose, three specimens (0.5 cm × 0.5 cm × 0.1 cm) were immersed in 20 mL of pure water at room temperature. Water uptake was measured at predetermined time intervals until the sample weights reached a constant value. At equilibrium, **11**_LAR40 NIPU exhibited an equilibrium water content (EWC) of 141.4 ± 10.6%, indicating a high-water absorption capacity, which can be attributed to its layered or phase-separated structure.

Since the materials synthesized showed poor mechanical properties in terms of Young modulus and tensile strength, we decided to cure *trans*-CADC with the polyfunctional polyethylene imine (PEI), aiming to increase the weight % of *trans*-CADC in the final cross-linked NIPU and hoping to boost the stiffness of the material without the need for adding external additives (Scheme 4 and Tables S6–S8).

**Scheme 4:**
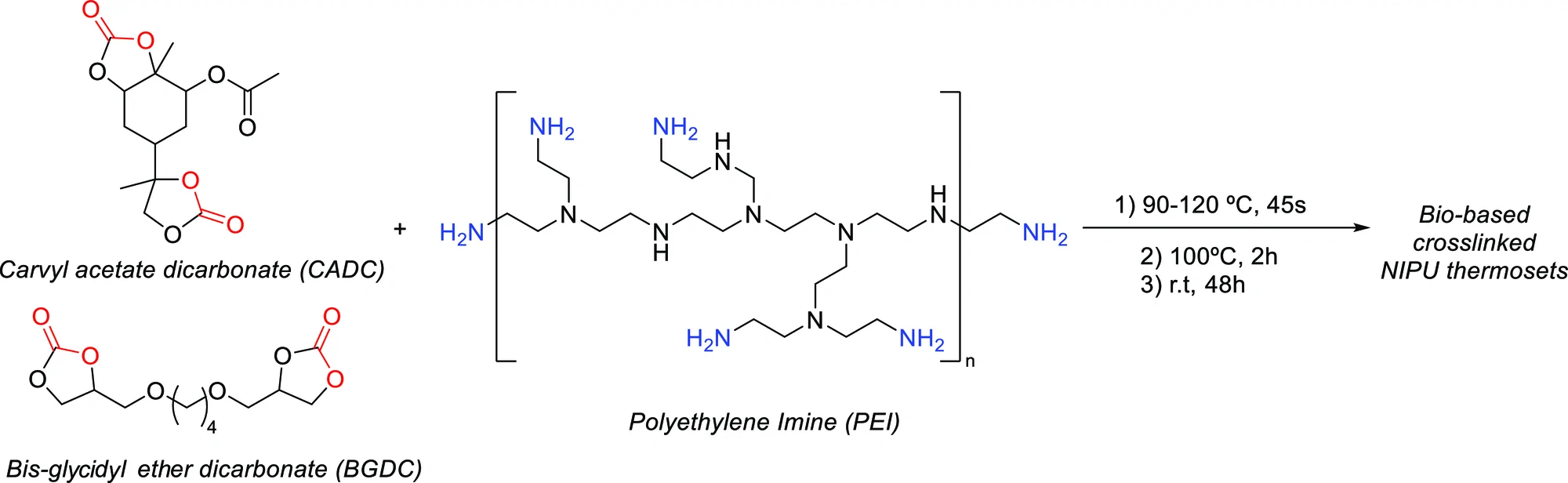
Curing of CADC and CADC/BGDC Mixtures with Polyethyleneimine as Cross-Linking Agent

Initially, *trans*-CADC was reacted with PEI, resulting in NIPU CADC_PEI (Table 3, entry 1), which exhibited a drastic brittle behavior, and mechanical tests failed. To overcome this, we introduced a more flexible bis(cyclic carbonate) to improve both viscosity and flexibility of the cross-linked network (Table 3, entries 2–5). Based on our previous work in NIPU synthesis, we centered out attention on 1,4-butanediol diglycidyl ether carbonate (BGDC), which is readily prepared by carbonatation of the commercially available 1,4-butanediol diglycidyl ether (BGDE).^47^ The addition of 10 wt % of BGDC to the CADC/PEI mixture had no observable effect whatsoever, and the resulting Gly_90CADC_PEI formulation still exhibited a severe embrittlement (Table 3, entry 2). However, increasing amounts of BGDC lead to the formation of materials with enhanced mechanical properties, within the range of those previously reported using other terpene-based building blocks (Table 3, entries 3–5, and Figure 3b).^43^ Tensile testings revealed a significant decrease in stiffness as the BGDC content in the NIPU formulations increased as reflected by the substantial decay of Young’s modulus (Table 3 and Figures 4a and S62–S64). This was especially visible when BGDC content increased from 50 to 70%, where the tensile strength decreased by 60% from 23.0 ± 2.0 to 9.4 ± 0.4 MPa (Table 3, entries 4 and 5, respectively). On the other hand, the increase content of BDGC did not translate into any significant change in terms of elongation break for all the materials synthesized (Table 3, entries 3–5).

**Table 3:** Thermal and Mechanical Properties of Bio-Based NIPU Thermosets Based on Different CADC/BGDC Mixtures and Cured with PEI^a^

entry	NIPU	mf (BGDC) [wt %]	*T*_g_ (°C)^b^	*T*_α_ (tan δ) (°C)^c^	Young’s modulus [MPa]	σ_B_ [MPa]^d^	ε_B_ [%]^d^	EWC (%)^e^
1	CADC_PEI	–	40.1	71.4	too brittle to measure	–	–	–
2	Gly_90CADC_PEI	10	38.1	53.6	too brittle to measure	–	–	–
3	Gly_70CADC_PEI	30	13.0	31.4	752 ± 41	26.4 ± 2.3	3.6 ± 0.5	70.5 ± 4.0
4	Gly_50CADC_PEI	50	10.6	14.2	440 ± 42	23.0 ± 2.0	5.4 ± 0.9	81.1 ± 5.3
5	Gly_30CADC_PEI	70	3.6	21.8	324 ± 29	9.4 ± 0.4	3.0 ± 0.2	92.3 ± 5.7

a For more experimental details, see section below, and Supporting Information.

b Determined by different scanning calorimetry analysis with a scan rate of 10 °C min^–1^.

c DMA (1 K/min, 0.1%, 1 Hz), *T*_α_ determined from the maximum of the tan δ.

d Tensile testing (ISO 37); σ_B_ represents the maximum tensile strength, ε_B_ is the elongation at rupture.

e Equilibrium water content EWC (%) = [(*W*_s_ – *W*_d_)/*W*_s_] × 100.

**Figure 4:**
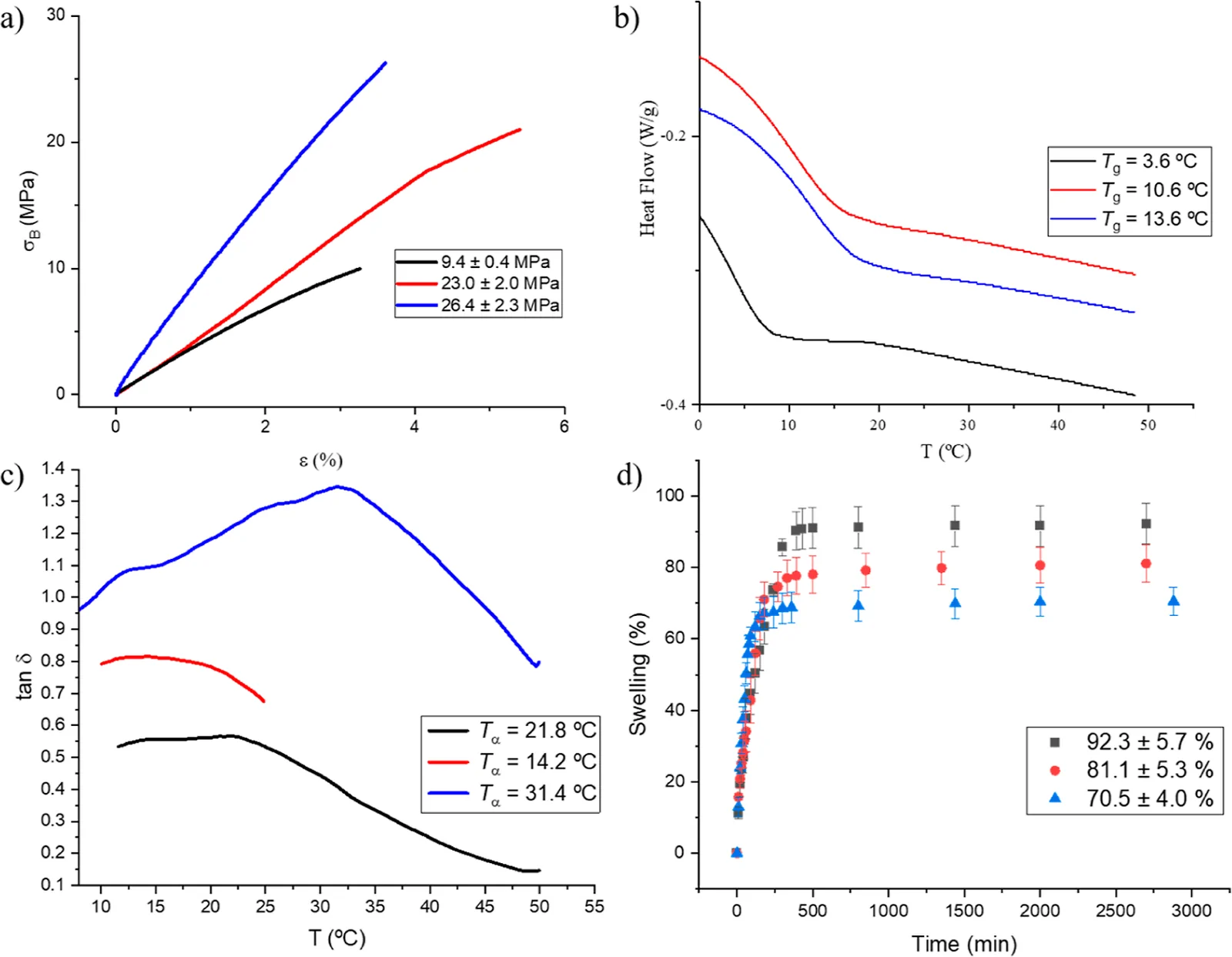
(a) Tensile test experiments (only average graphs have been included, for further information see Figures S62–S64); (b) DSC graphs; (c) DMA graphs and (d) water swelling experiments for Gly_70CADC_PEI (blue), Gly_50CADC_PEI (red) and Gly_30CADC_PEI (red) (Table 3, entries 3–5 respectively).

The thermal properties for the thermosetting materials synthesized also followed this same tendency (Figures S65–S74), with glass transition temperatures decreasing as the BGDC content in the formulation increased, from 40.1 °C for CADC_PEI to 3.5 °C for Gly_30CADC_PEI, reflecting the increasing flexibility of the polymer network as the BGDC-derived aliphatic segment is increased (Figure 4b). Moreover, dynamic mechanism analysis (DMA) (Figures 4c and S75–S79) further confirmed the influence of the monomer composition on the viscoelastic behavior of the resulting NIPUs. The glass transition temperatures (*T*_α_) were determined from the maximum of the tan δ curves, and the corresponding storage (*G*′) and loss modulus (*G*″) curves, calculated from tan δ, have been included in these plots (Figures S75–S79). The maximum in tan δ is associated with a pronounced decrease in *G*′ and the appearance of a shoulder in *G*″, providing further evidence of the glass transition. Although the glass transition temperatures estimated from the maximum of the tan δ peak generally followed the decrease observed by DSC, this relationship was not strictly monotonic, particularly for the Gly_30CADC_PEI polymer (Table 3, entry 5). Moreover, increasing the BGDC content resulted in a pronounced broadening of the tan δ relaxation peak, especially for the Gly_50CADC_PEI and Gly_30CADC_PEI, indicating a broader distribution of molecular relaxation times. Since the overall carbonyl-to-amine stoichiometry remained constant in all formulations, this behavior is attributed to the incorporation of more flexible aliphatic BGDC segments, which promote a wider distribution of local molecular environments and chain mobilities within the polymer network. Consequently, the temperature corresponding to the tan δ maximum becomes less representative of the average glass transition temperature determined by DSC; while broadening the relaxation reflects the increase dynamic heterogeneity of the BGDC-rich networks.

Water swelling experiments (Figures 4d and S80–S82) in purified water were also conducted on Gly_*x*CADC_PEI (*x* = 30, 50, 70, Table 3, entries 3–5) following the same procedure mentioned above for NIPU **11**_LAR40. All materials showed high water absorption, with EWC (%) ranging from 70.5 to 92.3% (Figures 4d and S80–S82). However, these values were considerably lower than that of the **11**_LAR40 NIPU (Table 2, entry 3). Clearly, the EWC increased as the BGDC content in the resulting NIPU formulation increased, exhibiting an inverse correlation with both the *T*_g_ and the Young’s modulus values. This enhanced swelling behavior may be attributed to the greater flexibility introduced by the BGDC-derived segments, which can increase chain mobility and facilitate water diffusion within the NIPU cross-linked network.^55^ The lower *T*_g_ values observed for BGDC-rich formulations further support the presence of a more flexible polymer structure, favoring water uptake and network expansion upon hydration.

## Conclusions

Carvyl acetate dicarbonate (CADC), synthesized via epoxidation and CO_2_ cycloaddition of the bioderived terpene (−)-carvyl acetate, has been demonstrated as a viable renewable monomer for the preparation of new nonisocyanate poly(hydroxyurethanes) (NIPUs). The aminolysis of CADC with various diamines afforded linear PHUs exhibiting moderate molar masses (2.5–7.8 kg mol^–1^) and glass-transition temperatures ranging from −31 to 62 °C, depending on the diamine structure. Reactions with Jeffamine yielded flexible thermoplastics, whereas the use of l-lysine under organocatalytic conditions provided higher-molecular-weight species. Cross-linking of CADC-derived oligomers with LAROMER LR9000 or polyethylenimine (PEI) produced thermosetting NIPUs spanning from ultrasoft, PSA-like networks to rigid, brittle materials. Incorporation of flexible bis(cyclic carbonate) BGDC allowed tuning of cross-link density and mechanical response, highlighting the need to optimize network architecture to balance strength and extensibility. Overall, CADC emerges as a promising terpene-based bis(cyclic carbonate) platform for biobased NIPUs.

## Experimental Section

### Optimized Carvyl Acetate Dicarbonate Synthesis

CADE (50 g, 0.22 mol) and TBAB (3.5 g, 11.0 mmol, 5 mol % with respect to the epoxide groups) were placed in a 500 mL stainless-steel reactor equipped with a magnetic stirring bar. The vessel was thoroughly closed, pressurized with 1 bar CO_2_, and vented three times to remove all the air. Then, the reactor was pressurized at the desired CO_2_ pressure of 40 bar and placed in a heating mantle at 120 °C. After stirring it for 72 h, the reactor was cooled down to room temperature yielding to a brown oily viscous mixture. An aliquot was taken for NMR analysis (CDCl_3_) to determine the conversion and selectivity. The reaction mixture was purified by flash chromatography (hexane/EtOAc gradient from 5:1 to 1:2). Isolated yield toward CADC: 35 g, 50%.

### CADC Purification and Crystallization

The CADC fractions (35.0 g) collected after column chromatography were transferred into a 500 mL round-bottom flask. Solvent was removed and the resulting solid redissolved in EtOAc (50 mL). *n*-hexane (100 mL) was then added on top, and the solution was placed at −20 °C in the freezer until a white crystalline precipitate formed. The crystalline solid was recovered by filtration, washed with cold *n*-hexane, and dried in vacuum. A small aliquot was taken for X-ray diffraction analysis. Recovered mass: 15 g, 43% yield. NMR (500 MHz, CDCl_3_): δ_H_ 5.05 (dd, *J*_H–H_ = 8.4, 4.8 Hz, 1H), 4.51 (dd, *J*_H–H_ = 9.2, 7.1 Hz, 1H), 4.30 (d, *J*_H–H_ = 8.8 Hz, 1H), 4.13 (d, *J*_H–H_ = 7.0 Hz, 1H), 2.22 (s, 1H), 2.06 (s, 3H), 1.97 (s, 1H), 1.62–1.55 (m, 3H), 1.53 (s, 3H), 1.51 (s, 3H). ^13^C-{^1^H}-NMR (125 MHz, CDCl_3_): δ_C_ 169.8, 153.7, 153.4, 84.0, 81.5, 79.6, 76.8, 72.6, 71.5, 37.0, 27.9, 26.1, 22.7, 20.8. IR (neat)_ν_max__ = 1791.14 (C=O_cyclic carbonate_), 1733.15 (C=O_acetate_).

### General Procedure for Hydroxycarbamates Synthesis

In a 10 mL Schlenk flask equipped with a small stir bar, CADC was added and dissolved in 2 mL of MeCN. Then, the corresponding diamine was added in a 1:0.5 and 1:2 molar ratio with respect to CADC respectively. The reaction mixture was then warmed up to 80 °C and left stirring for 16 h. After that time, solvent was removed in vacuo and the residue was taken up with MeOH. The mixture was filtered, and MeOH removed to afford the corresponding carbamate compound as a white-brownish solid in 85–90% yield.

### General Procedure for NIPU Thermoplastics Synthesis

In a 10 mL Schlenk flask equipped with a small stir bar, CADC was added and dissolved in 2 mL of MeCN. Then, the corresponding diamine was added in a 1:1 molar ratio with respect to CADC. The reaction mixture was then warmed up to 80 °C and left stirring for 16 h. After that time, solvent was removed in vacuo, and the residue was taken up with MeOH. The mixture was filtered, and MeOH removed to afford the corresponding NIPU thermoplastic as a white-brownish solid in 81–93% yield.

### CADC/BGDC Blends-PEI Cross-Linking Tests

In a 5 mL glass vial equipped with a small stir bar, the corresponding amount of bis(cyclic carbonates) purified CADC and BGDC were added and the solid mixture was warmed up until they melted (see Tables S6 and S7). Then, the corresponding amount of PEI (see Table S8) was added with a syringe, and the resulting mixture was left stirring for 45 s. After that time, the resulting viscous mixture was transferred with an oven preheated syringe to a Teflon mold. For the curing process, the resulting formulations were placed in the oven at 100 °C for 2 h followed by postcuring at room temperature for 24 h.

## Supplementary Material





## Data Availability

The data underlying this study are available in the published article and its Supporting Information.

## Notes

SEC traces, DSC and TGA analyses, and tests for the study of mechanical properties of NIPU materials have been included in the Supporting Information (CIF)
Supporting Information including experimental details, procedures for catalytic reactions, chemical structures of carvyl acetate dicarbonate, carbamates, nonisocyanate poly(hydroxyurethanes), thermoplastics, and thermosetting NIPUs. Spectroscopic data, NMR and FTIR spectra, for cyclic carbonates and NIPUs materials have been included in Supporting Information.
(PDF)

## Abbreviations

BASF: Badische Anilin-and Soda-Fabrik
BDA: 1,4-diaminobutane
BGDC: 1,4-butanediol diglycidyl ether carbonate
BGDE: 1,4-butanediol diglycidyl ether
CADC: carvyl acetate decarbonate
CADE: carvyl acetate diepoxide
CAMC: carvyl acetate monocarbonate
DMF: dimethylformamide
2-MeTHF: 2-methyltetrahydrofuran
DMA: Dynamic mechanical analysis
*G*′: storage modulus
*G*″: loss modulus
DSC: differential scanning calorimeter
FTIR: Fourier transform infrared spectroscopy
IR: infrared spectroscopy
*M*_n_: molecular weight number
NIPUs: nonisocyanate polyurethanes
NMR: nuclear magnetic resonance spectroscopy
PEI: polyethylene imine
PDI: polydispersity
PHUs: polyhydroxyurethanes
PU: polyurethane
PUs: polyurethanes
TBAB: tetrabutylammonium bromide
TBD: 1,5,7-triazabicyclo[4.4.0]dec-5-ene
*T*_d_: thermal decomposition
*T*_g_: glass transition temperatures
TGA: thermogravimetric analyses
σ_B_represents the maximum tensile strengthε_B_: represents the maximum tensile strengthε_B_is the elongation at rupture

## References

[1] Gomez-Lopez A., Elizalde F., Calvo I., Sardon H. (2021). Trends in non-isocyanate
polyurethane (NIPU) development. Chem. Commun..

[2] Balla E., Bikiaris D. N., Pardalis N., Bikiaris N. D. (2025). Toward Sustainable
Polyurethane Alternatives: A Review of the Synthesis, Applications,
and Lifecycle of Non-Isocyanate Polyurethanes (NIPUs). Polymers.

[3] Das A., Mahanwar P. (2020). A Brief Discussion
on Advances in Polyurethane Applications. Adv.
Ind. Eng. Polym. Res..

[4] Khan T., Acar V., Aydin M. R., Hülagü B., Akbulut H., Seydibeyoğlu M.
O. ¨. (2020). A Review on
Recent Advances in Sandwich Structures Based on Polyurethane Foam
Cores. Polym. Compos..

[5] Wendels S., Avérous L. (2021). Biobased Polyurethanes for Biomedical Applications. Bioact. Mater..

[6] Cong L., Yang F., Guo G., Ren M., Shi J., Tan L. (2019). The Use of Polyurethane for Asphalt
Pavement Engineering Applications:
A State-of-the-Art Review. Constr. Build. Mater..

[7] Plastics Europe , Plastics–the Facts 2024, available at: https://plasticseurope.org/knowledge-hub/plastics-the-fast-facts-2024/ (accessed: 29 September, 2025).

[8] Zhang J., Shen H. Z., Li H. Y., Gu L. F. (2021). Effects of Temperature,
Strain Rate, and Cyclic Loading on Mechanical Behavior of Silane-Modified
Polyurethane Sealant. J. Appl. Polym. Sci..

[9] Zhang D., Zeng J., Liu W., Qiu X., Qian Y., Zhang H., Yang Y., Liu M., Yang D. (2021). Pristine Lignin
as a Flame Retardant in Flexible PU Foam. Green
Chem..

[10] Zhu G. J., Ren P. G., Guo H., Jin Y. L., Yan D. X., Li Z. M. (2019). Highly Sensitive
and Stretchable Polyurethane Fiber Strain Sensors
with Embedded Silver Nanowires. ACS Appl. Mater.
Interfaces.

[11] Gama N. V., Ferreira A., Barros-Timmons A. (2018). Polyurethane Foams: Past, Present,
and Future. Materials.

[12] Peng W., Zhao N., Xiao F., Wei W., Sun Y. (2011). Recent Progress
in Phosgene-Free Methods for Synthesis of Dimethyl Carbonate. Pure Appl. Chem..

[13] Pauluhn J. (2021). Phosgene Inhalation
Toxicity: Update on Mechanisms and Mechanism-Based Treatment Strategies. Toxicology.

[14] Labunska, I. ; Stephenson, A. ; Brigden, K. ; Stringer, R. ; Santillo, D. ; Johnston, P. A. The Bhopal Legacy, Toxic Contaminants at the Former Union Carbide Factory Site, Bhopal, India: 15 Years After the Bhopal Accident; Greenpeace Research Laboratories, Department of Biological Sciences, University of Exeter: Exeter, 1999.

[15] Sparrow, D. J. ; Thorpe, D. Polyols for Polyurethane Production. In Telechelic Polymers: Synthesis and Applications; CRC Press, 2018; pp 181–228.

[16] Sharan I., Sinha S., Srivastava V. C. (2025). State-of-the-Art
and Recent Progress
in the Synthesis of Polyurethanes. Rev. Chem.
Eng..

[17] Maisonneuve L., Lamarzelle O., Rix E., Grau E., Cramail H. (2015). Isocyanate-Free
Routes to Polyurethanes and Poly(Hydroxy Urethane)s. Chem. Rev..

[18] Turnaturi R., Zagni C., Patamia V., Barbera V., Floresta G., Rescifina A. (2023). CO_2_-Derived Non-Isocyanate
Polyurethanes
(NIPUs) and Their Potential Applications. Green
Chem..

[19] Blattmann H., Fleischer M., Bähr M., Mülhaupt R. (2014). Isocyanate-
and Phosgene-Free Routes to Polyfunctional Cyclic Carbonates and Green
Polyurethanes by Fixation of Carbon Dioxide. Macromol. Rapid Commun..

[20] Theerathanagorn T., Kessaratikoon T., Rehman H. U., D’Elia V., Crespy D. (2024). Polyhydroxyurethanes from Biobased Monomers and CO_2_: A Bridge between Sustainable Chemistry and CO_2_ Utilization. Chin. J. Chem..

[21] Haniffa M. A. C. M., Munawar K., Ching Y. C., Illias H. A., Chuah C. H. (2021). Bio-Based
Poly(Hydroxy Urethane)s: Synthesis and Pre/Post-Functionalization. Chem.—Asian J..

[22] Wu Q., Pudukudy M., Chen B. Y., Zhi Y. F., Shan S. Y. (2023). Recent
Advances in the Synthesis of Bio-Based Carbonate by the Catalytic
Reaction of CO_2_ with Bio-Based Compounds. ChemistrySelect.

[23] Blazek K., Benes H., Walterová Z., Abbrent S., Eceiza A., Calvo-Correas T., Datta J. (2021). Synthesis and structural characterization
of bio-based bis(cyclic carbonate)s for the preparation of non-isocyanate
polyurethanes. Polym. Chem..

[24] Chen Q., Gao K., Peng C., Xie H., Zhao Z. K., Bao M. (2015). Preparation
of Lignin/Glycerol-Based Bis(Cyclic Carbonate) for the Synthesis of
Polyurethanes. Green Chem..

[25] Gregory G. L., Lopez-Vidal E. M., Buchard A. (2017). Polymers from Sugars: Cyclic Monomer
Synthesis, Ring-Opening Polymerisation, Material Properties and Applications. Chem. Commun..

[26] Tremblay-Parrado K. K., García-Astrain C., Avérous L. (2021). Click Chemistry
for the Synthesis of Biobased Polymers and Networks Derived from Vegetable
Oils. Green Chem..

[27] Abbasi A., Nasef M. M., Yahya W. Z. N. (2019). Copolymerization of Vegetable Oils
and Bio-Based Monomers with Elemental Sulfur: A New Promising Route
for Bio-Based Polymers. Sustainable Chem. Pharm..

[28] Prömpers G., Keul H., Höcker H. (2005). Polyurethanes
with Pendant Hydroxy
Groups: Polycondensation of D-Mannitol-1,2:5,6-Dicarbonate with Diamines. Des. Monomers Polym..

[29] Clark J. H., Farmer T. J., Ingram I. D. V., Lie Y., North M. (2018). Renewable
Self-Blowing Non-Isocyanate Polyurethane Foams from Lysine and Sorbitol. Eur. J. Org. Chem..

[30] Zhang L., Luo X., Qin Y., Li Y. (2017). A Novel 2,5-Furandicarboxylic Acid-Based
Bis(Cyclic Carbonate) for the Synthesis of Biobased Non-Isocyanate
Polyurethanes. RSC Adv..

[31] More A. S., Gadenne B., Alfos C., Cramail H. (2012). AB Type Polyaddition
Route to Thermoplastic Polyurethanes from Fatty Acid Derivatives. Polym. Chem..

[32] Maisonneuve L., Wirotius A. L., Alfos C., Grau E., Cramail H. (2014). Fatty Acid-Based
(Bis) 6-Membered Cyclic Carbonates as Efficient Isocyanate Free Poly(Hydroxyurethane)
Precursors. Polym. Chem..

[33] Bass G. F., Epps T. H. (2021). Recent Developments towards Performance-Enhancing Lignin-Based
Polymers. Polym. Chem..

[34] Zhao S., Abu-Omar M. M. (2021). Materials Based on Technical Bulk Lignin. ACS Sustainable Chem. Eng..

[35] Mahajan J. S., O’Dea R. M., Norris J. B., Korley L. S. T. J., Epps T. H. (2020). Aromatics from Lignocellulosic Biomass: A Platform
for High-Performance Thermosets. ACS Sustainable
Chem. Eng..

[36] O’dea R. M., Willie J. A., Epps T. H. (2020). 100th Anniversary of Macromolecular
Science Viewpoint: Polymers from Lignocellulosic Biomass. Current
Challenges and Future Opportunities. ACS Macro
Lett..

[37] Sawpan M. A. (2018). Polyurethanes
from Vegetable Oils and Applications: A Review. J. Polym. Res..

[38] Rayung M., Ghani N. A., Hasanudin N. (2024). A Review on
Vegetable Oil-Based Non-Isocyanate
Polyurethane: Towards a Greener and Sustainable Production Route. RSC Adv..

[39] Bähr M., Mülhaupt R. (2012). Linseed and Soybean Oil-Based Polyurethanes Prepared
via the Non-Isocyanate Route and Catalytic Carbon Dioxide Conversion. Green Chem..

[40] Poussard L., Mariage J., Grignard B., Detrembleur C., Jérôme C., Calberg C., Heinrichs B., De Winter J., Gerbaux P., Raquez J. M., Bonnaud L., Dubois P. (2016). Non-Isocyanate Polyurethanes from Carbonated Soybean
Oil Using Monomeric or Oligomeric Diamines to Achieve Thermosets or
Thermoplastics. Macromolecules.

[41] Rix E., Grau E., Chollet G., Cramail H. (2016). Synthesis of Fatty
Acid-Based Non-Isocyanate Polyurethanes, NIPUs, in Bulk and Mini-Emulsion. Eur. Polym. J..

[42] Bähr M., Bitto A., Mülhaupt R. (2012). Cyclic Limonene
Dicarbonate as a
New Monomer for Non-isocyanate Oligo- and Polyurethanes (NIPU) Based
upon Terpenes. Green Chem..

[43] Schimpf V., Ritter B. S., Weis P., Parison K., Mülhaupt R. (2017). High Purity
Limonene Dicarbonate as Versatile Building Block for Sustainable Non-Isocyanate
Polyhydroxyurethane Thermosets and Thermoplastics. Macromolecules.

[44] Maquilón C., Brandolese A., Alter C., Hövelmann C. H., Della Monica F., Kleij A. W. (2022). Renewable Beta-Elemene Based Cyclic
Carbonates for the Preparation of Oligo(Hydroxyurethane)s. ChemSusChem.

[45] Scheelje F. C. M., Meier M. A. R. (2023). Non-Isocyanate Polyurethanes Synthesized from Terpenes
Using Thiourea Organocatalysis and Thiol-Ene-Chemistry. Commun. Chem..

[46] de
la Cruz-Martínez F., Martínez de Sarasa
Buchaca M., Martínez J., Fernández-Baeza J., Sánchez-Barba L. F., Rodríguez-Diéguez A., Castro-Osma J. A., Lara-Sánchez A. (2019). Synthesis of Bio-Derived Cyclic Carbonates
from Renewable Resources. ACS Sustainable Chem.
Eng..

[47] Martínez
de Sarasa Buchaca M., de la Cruz-Martínez F., Francés-Poveda E., Fernández-Baeza J., Sánchez-Barba L. F., Garcés A., Castro-Osma J. A., Lara-Sánchez A. (2022). Synthesis
of Nonisocyanate Poly(Hydroxy)Urethanes from Bis(Cyclic Carbonates)
and Polyamines. Polymers.

[48] Magliozzi F., Scali A., Chollet G., Grau E., Cramail H. (2020). Enantioselective
Crystallization of Diglycerol Dicarbonate: Impact of the Microstructure
on Polyhydroxyurethane Properties. Macromol.
Rapid Commun..

[49] Błażek K., Beneš H., Walterová Z., Abbrent S., Eceiza A., Calvo-Correas T., Datta J. (2021). Synthesis and Structural Characterization
of Bio-Based Bis(Cyclic Carbonate)s for the Preparation of Non-Isocyanate
Polyurethanes. Polym. Chem..

[50] Janvier M., Ducrot P. H., Allais F. (2017). Isocyanate-Free Synthesis and Characterization
of Renewable Poly(Hydroxy)Urethanes from Syringaresinol. ACS Sustainable Chem. Eng..

[51] Kim J. G., Lee G. S., Lee A. (2024). Triazabicyclodecene:
A Versatile
Catalyst for Polymer Synthesis. J. Polym. Sci..

[52] Monmagnon A., Bayle P. A., Flaig F., Carpe C., El Harfi J., Demadrille R., Rolere S. (2024). Insights on the Polymerization Kinetics
of Non-Isocyanate Polyurethanes (NIPU) Using in Situ NMR Spectroscopy. Polym. Test..

[53] Bossion A., Aguirresarobe R. H., Irusta L., Taton D., Cramail H., Grau E., Mecerreyes D., Su C., Liu G., Müller A. J., Sardon H. (2018). Unexpected Synthesis of Segmented
Poly(Hydroxyurea-Urethane)s from Dicyclic Carbonates and Diamines
by Organocatalysis. Macromolecules.

[54] Lambeth H. R., Henderson J. T. (2013). Organocatalytic
synthesis of (poly)hydroxyurethanes
from cyclic carbonates and amines. Polymer.

[55] Gennen S., Grignard B., Thomassin J.-M., Gilbert B., Vertruyen B., Jerome C., Detrembleur C. (2016). Polyhydroxyurethane
hydrogels: Synthesis
and characterizations. Eur. Polym. J..

