# Supplementary material for: Terpene-Derived Carvyl Acetate Dicarbonate: A Novel Monomer for Biobased Nonisocyanate Poly(hydroxyurethane) Thermoplastics and Thermosets
Source: Biomacromolecules. 2026 Aug 10;27(10):6943–53. doi: 10.1021/acs.biomac.6c01553 (PMC13690295; doi:10.1021/acs.biomac.6c01553)
Supplement: Supplementary file 1 [file bm6c01553_si_003.pdf]

APPENDIX A. SUPPORTING INFORMATION

Terpene-derived carvyl acetate dicarbonate: a novel monomer for the preparation of bio-based non-isocyanate poly(hydroxyurethanes) thermoplastics and thermosets

Marc Martínez de Sarasa Buchaca,[*,a] *Jesús Naranjo,*[a] *Alberto Moreno-Fernández,*[a] *Carmen Moya-López,*[b] *Pablo Salcedo-Abraira,*[c] *Antonio Rodríguez-Diéguez,*[c] *Carlos Gracia-Fernández,*[d] *Antonio M. Rodríguez-García,* [a] *José A. Castro-Osma,* [a] *Felipe de la Cruz-Martínez,*[*,a] *Agustín Lara-Sánchez* [*,a]

[a]Universidad de Castilla-La Mancha, Departamento de Química Inorgánica, Orgánica y Bioquímica-Centro de Innovación en Química Avanzada (ORFEO-CINQA), Facultad de Ciencias y Tecnologías Químicas and Instituto Regional de Investigación Científica Aplicada-IRICA 13071-Ciudad Real, 13071-Ciudad Real, Spain. E-mail: Marc.MartinezSarasa@uclm.es; Felipe.Cruz@uclm.es; Agustin.Lara@uclm.es.

[b]Universidad de Castilla-La Mancha, Departamento de Química Inorgánica, Orgánica y Bioquímica-Centro de Innovación en Química Avanzada (ORFEO-CINQA), Facultad de Farmacia, 02071-Albacete, Spain.

[c]Universidad de Granada, Departamento de Química Inorgánica, Facultad de Ciencias, 18071-Granada, Spain

[d] TA Instruments Waters Chromatography, Tres Cantos, 28760 Madrid, Spain

Table of contents

Page S3. General Considerations

Page S5. Carvyl acetate dicarbonate (CADC) synthesis

Page S12. Crystallographic data of CADC

Page S14. General procedure for hydroxycarbamates synthesis

Page S17. Possible regioisomers in the preparation of hydroxycarbamates **1b** and **2b**.

Page S19. General procedure for NIPU thermosplastics synthesis

Page S23. FT-IR spectra of hydroxycarbamates **1**,**2a** and **1**,**2b**

Page S25. NMR spectra of hydroxycarbamates **1**,**2a** and **1**,**2b**

Page S31. FT-IR spectra of NIPUs **8–12**

Page S34. NMR spectra of NIPUs **8–12**

Page S39. TGA graphs and DSC traces of NIPUs **8–12**

Page S45. SEC traces for NIPU thermoplastics **8–12**

Page S46. NIPU **11**-LAROMER® LR9000 cross-linking tests

Page S47. Tensile testing results of **11**LAR_40 (ISO 37)

Page S48. TGA graph and DSC trace of **11**LAR_40 thermoset

Page S49. Swelling test results of **11**LAR_40 thermoset

Page S50. CADC/BGDC-PEI cross-linking tests

Page S51. Stoichiometric calculations for CADC/BGDC-PEI cross-linked NIPUs

Page S52. Tensile testing results of Gly_CADC_PEI thermosetting materials (ISO 37)

Page S55. TGA graphs and DSC traces of Gly_CADC_PEI thermosetting materials

Page S60. DMA graphs of Gly_CADC_PEI thermosetting materials

Page S63. Swelling test results of Gly_CADC_PEI thermosetting materials

Page S66. References

S3. General Considerations

All manipulations of air and water sensitive compounds were carried out under dry nitrogen using a Braun Labmaster glovebox or standard Schlenk line techniques. ^{1}H and ^{13}C NMR spectra were recorded on a Bruker Ascend TM-500/400 and referenced to the residual deuterated solvent. Thermogravimetric analysis of the products was performed on a TGA instrument (model TGA-Q50). The heating rate for the sample was 10 °C/min and the nitrogen flow rate was 60 mL/min. Differential scanning calorimetry (DSC) curves were obtained under a N_2 atmosphere on TA Instrument (model DSC-Q20). Samples were weighed into aluminum crucibles with 5 mg of sample and subjected to two heating cycles at a heating rate of 10 °C/min. Gel permeation chromatography (GPC) measurements were performed on a Polymer Laboratories PL-GPC-220 instrument equipped with a TSK-GEL G3000H column and an ELSD-LTII light scattering detector or/and a RID-20A differential Refractive Index Detector. The GPC column was eluted with dimethylacetamide (DMAc) at 50 °C at a flow rate of 1 mL min^{-1} and was calibrated using eight monodisperse polymethylmethacrylate (PMMA) standards in the range 580–48300 Da. Mechanical tests were carried out on a Mecmesin MultiTest-i 2.5-I dynamic mechanical analyser (Mecmesin, West Sussex, UK) at room temperature. Tensile tests were carried out on cross-linked NIPU samples prepared on a mould made of Teflon®, which meets the ISO 37 standard. The thickness of the NIPU samples was evaluated using a digital micrometer (0-25mm ± 0.001) (Palmer Wahl, Asheville, NC, USA). The average thickening value of the films was determined by taking 6 measurements at random positions in the gel. Samples were anchored by tweezers on the analyser and uniaxially stretched at 50 mm min^{-1} until their breaking point, using cell loads from 50–200 N. σ_B represents Maximum tensile strength, Youngs's Modulus (YM) and ε_B as the elongation at rupture were determined. Firstly, σ_B was determined as the maximum pressure value supported by the gel just before break expressed in MPa. The Young´s modulus, that indicates the elasticity of the gel, was calculated from the slope of the linear range of the stress-strain curve (between 2% and 10% of strain) and expressed as MPa. The elongation at rupture (ε_B) corresponds to the strain (%) at the point of failure, indicating the ductility of the material. The experiments were performed at least in triplicate. Dynamic mechanical analysis (DMA) were performed in torsion using an HR 10 rheometer (Waters Materials Sciences, Delaware, USA). The sample dimensions were 2 mm in thickness, 8

mm in width, and 10 mm in length.The strain amplitude was 0.1%, the frequency was 1 Hz, and the heating rate was 1 °C/min.

Crystallographic studies

Single-crystal X-ray diffraction experiments were performed on a Bruker X8 APEX II diffractometer equipped with Mo Kα radiation (0.71073 Å) at room temperature. Suitable crystals of CADC sample were mounted on a MiTeGen polymer loop using FOMBLING oil. Data reduction was performed with APEX II software, and absorption correction was applied using SADABS. The crystal was found to be twinned, and thus, processed as inversion twin. The crystalline structure was solved and refined using the SHELX software package integrated in Olex2 graphical interface. All non-H atoms were refined anisotropically. Hydrogen atoms were placed in calculated positions and refined with idealized geometries. Crystallographic data (excluding structure factors) have been deposited with the Cambridge Crystallographic Data Centre (deposition number 2516525). These data can be obtained free of charge from the Cambridge Crystallographic Data Centre via http://www.ccdc.cam.ac.uk/Community/Requestastructure.

Reagents

Solvents were pre-dried over sodium wire and distilled under nitrogen from sodium (MeCN). Deuterated DMSO was purchased from VWR Chemicals and stored under activated 4Å molecular sieves. Unless stated otherwise, all reagents were purchased from commercial sources and used as received. Carvyl acetate bis-epoxide (CADE) and bis-glicydyl ether carbonate (BGDC) were synthesized according to previous literature procedures.[1,2] Jeffamine® or Poly(propylene glycol) bis(2-aminopropyl ether) (M_w ~ 1900) was purchased from Sigma Aldrich. LAROMER® LR9000 was kindly provided by BASF company.

S5. Carvyl acetate dicarbonate (CADC) synthesis

CADE (0.5–50.0 g, 2.20–222.0 mmol) and TBAB (0.35–3.54 g, 0.011–11.0 mmol, 5 mol% with respect to the epoxide groups) were placed in a 500 mL stainless-steel reactor equipped with a magnetic stirring bar. The vessel was thoroughly closed, pressurized with 1 bar CO_2, and vented three times to remove all the air. Then, the reactor was pressurized at the desired CO_2 pressure of 40 bar and placed in a heating mantle at 120 ºC. After stirring it for 72 h, the reactor was cooled down to room temperature yielding to a brown oily viscous mixture. An aliquot was taken for NMR analysis ($CDCl_3$) to determine the conversion and selectivity. The reaction mixture was purified by flash chromatography (Hex:EtOAc gradient from 5:1 to 1:2). Isolated yield towards CADC: 35 g, 50%.

Table S1. Carbonatation of carvyl acetate diepoxide (CADE).[a]

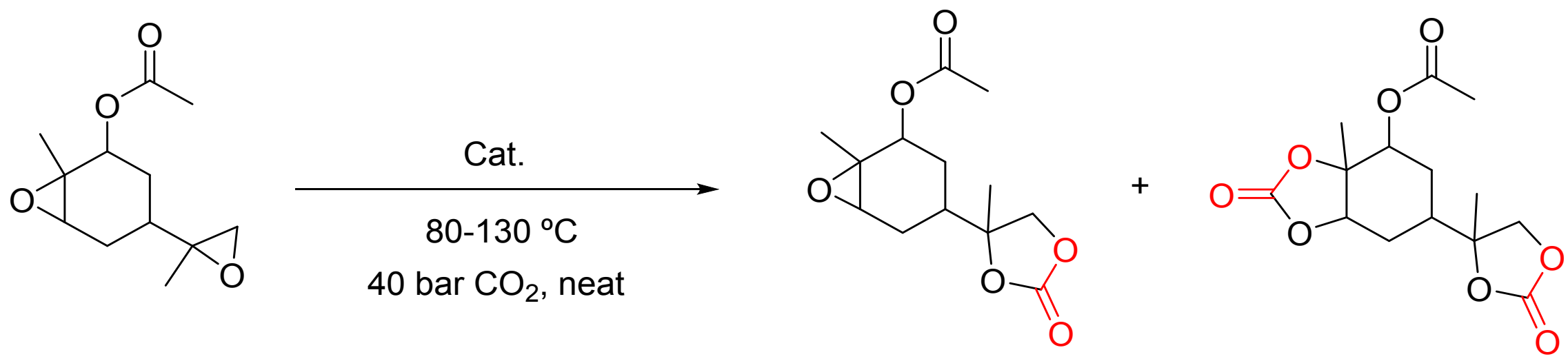

Entry	Temperature (°C)	Catalyst (mol%)	Time (h)	CAMC/CADC (%)[b]
1	80	TBAB (5)	24	66/33
2	110	TBAB (5)	24	54/46
3	120	TBAB (5)	24	32/68
4	130	TBAB (5)	24	-
5	120	TBAB (5)	48	20/80
6	120	TBAB (5)	72	10/90
7	120	TBAC (5)	72	20/80
8	120	TBAI (5)	72	29/71
9	120	PPNCl (5)	72	25/75
10[c]	120	TBAB (5)	72	30/70
11[d]	120	TBAB (5)	72	40/60

[a]Neat, 2.2 mmol CADE, 0.01 mmol TBAB. [b]Calculated by ^1H NMR integration between epoxide/dicarbonate signals. [c]8.80 mmol CADE. [d]0.22 mol CADE.

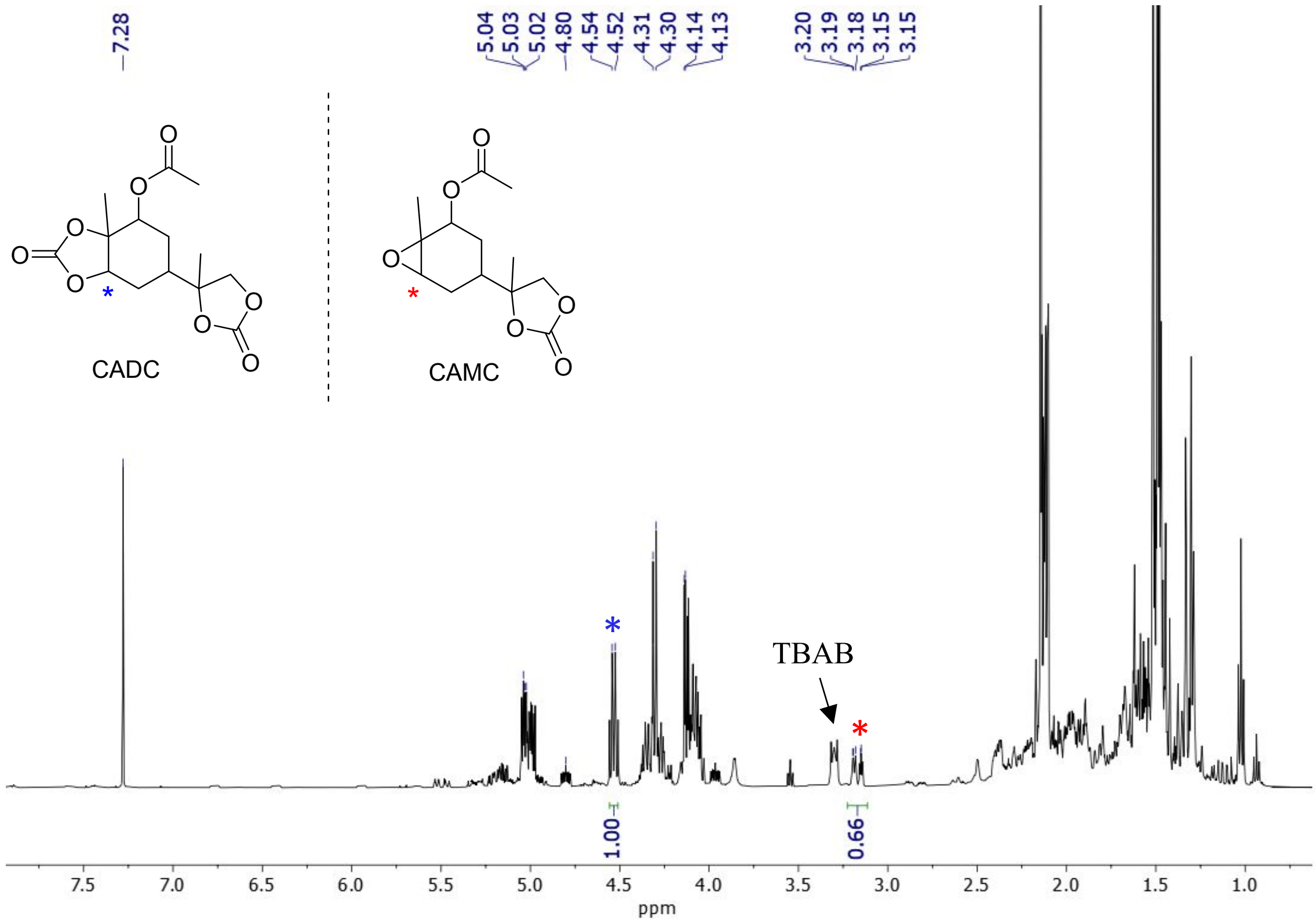

Figure S1. ^{1}H-NMR spectrum for the crude mixture after carbonatation of CADE (Table S1, entry 11).

CADC purification and crystallization

The CADC fractions (35.0 g) collected after column chromatography containing a complex mixture of different diastereomers were transferred into a 500 mL round bottom flask. Solvent was removed and the resulting solid redissolved in EtOAc (50 mL). *n*-hexane (100 mL) was then added on top and the solution was placed at −20 °C in the freezer until a white crystalline precipitate formed. The crystalline solid was recovered by filtration, washed with cold *n*-hexane, and dried in vacuum. A small aliquot was taken for X-Ray diffraction analysis. Recovered mass: 15 g, 43% yield. The CADC-crystals (from now on, *trans*-CADC) were fully characterized via NMR as shown below. ^{1}H-NMR (500 MHz, $CDCl_3$): δ_H = 5.05 (dd, $J_{H\text{-}H}$ = 8.4, 4.8 Hz, 1H), 4.51 (dd, $J_{H\text{-}H}$ = 9.2, 7.1 Hz,

1H), 4.30 (d, J_{H-H} = 8.8 Hz, 1H), 4.13 (d, J_{H-H} = 7.0 Hz, 1H), 2.22 (s, 1H), 2.06 (s, 3H), 1.97 (s, 1H), 1.62−1.55 (m, 3H), 1.53 (s, 3H), 1.51 (s, 3H). $^{13}C-\{^1H\}$-NMR (125 MHz, $CDCl_3$): δ_C = 169.8, 153.7, 153.4, 84.0, 81.5, 79.6, 76.8, 72.6, 71.5, 37.0, 27.9, 26.1, 22.7, 20.8. IR (neat) ν_{max} = 1791.14 ($C{=}O_{cyclic\ carbonate}$), 1733.15 ($C{=}O_{acetate}$).

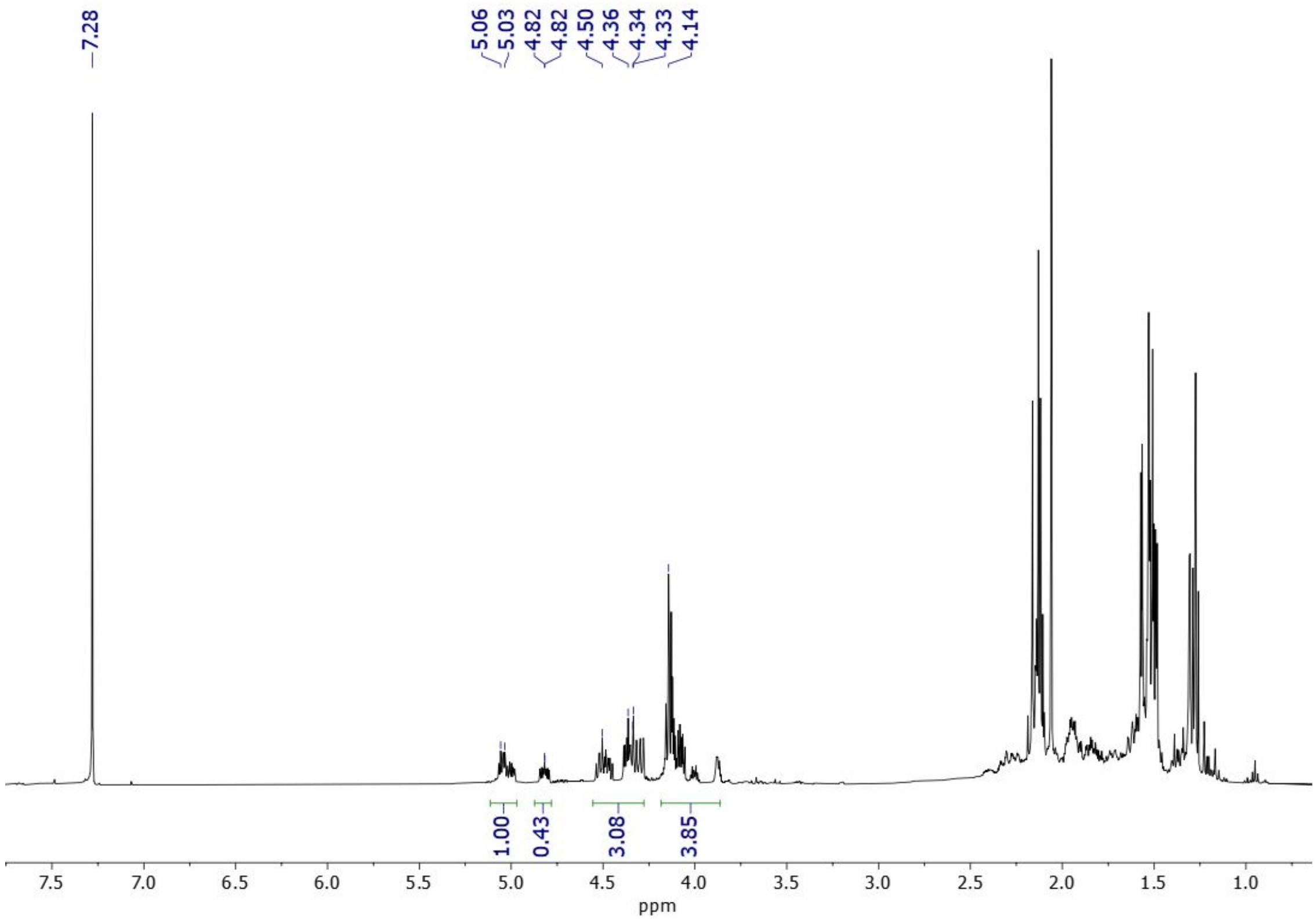

Figure S2. ^1H-NMR spectrum for the collected fractions of CADC after column chromatography

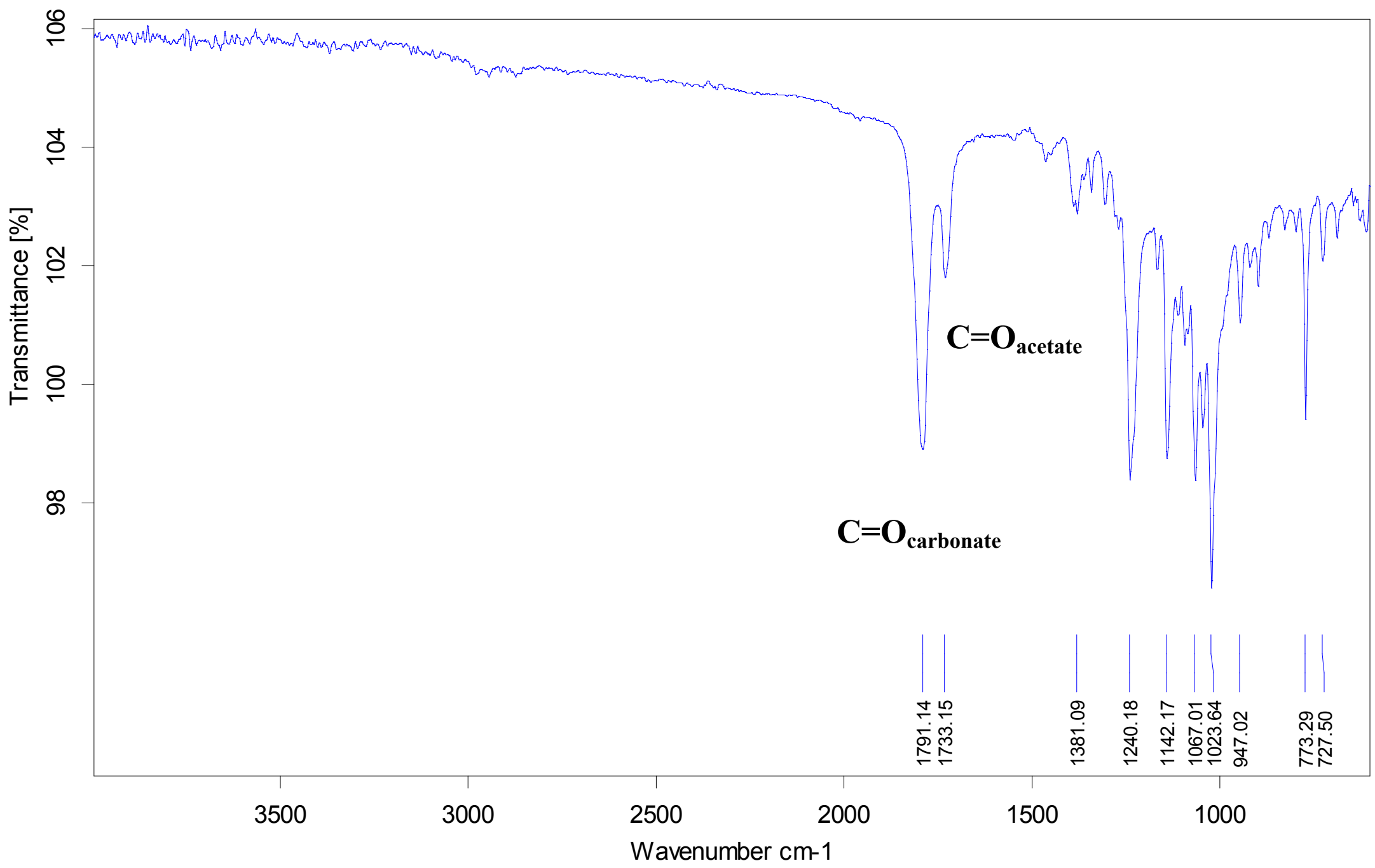

Figure S3. FT-IR spectrum for *trans*-CADC

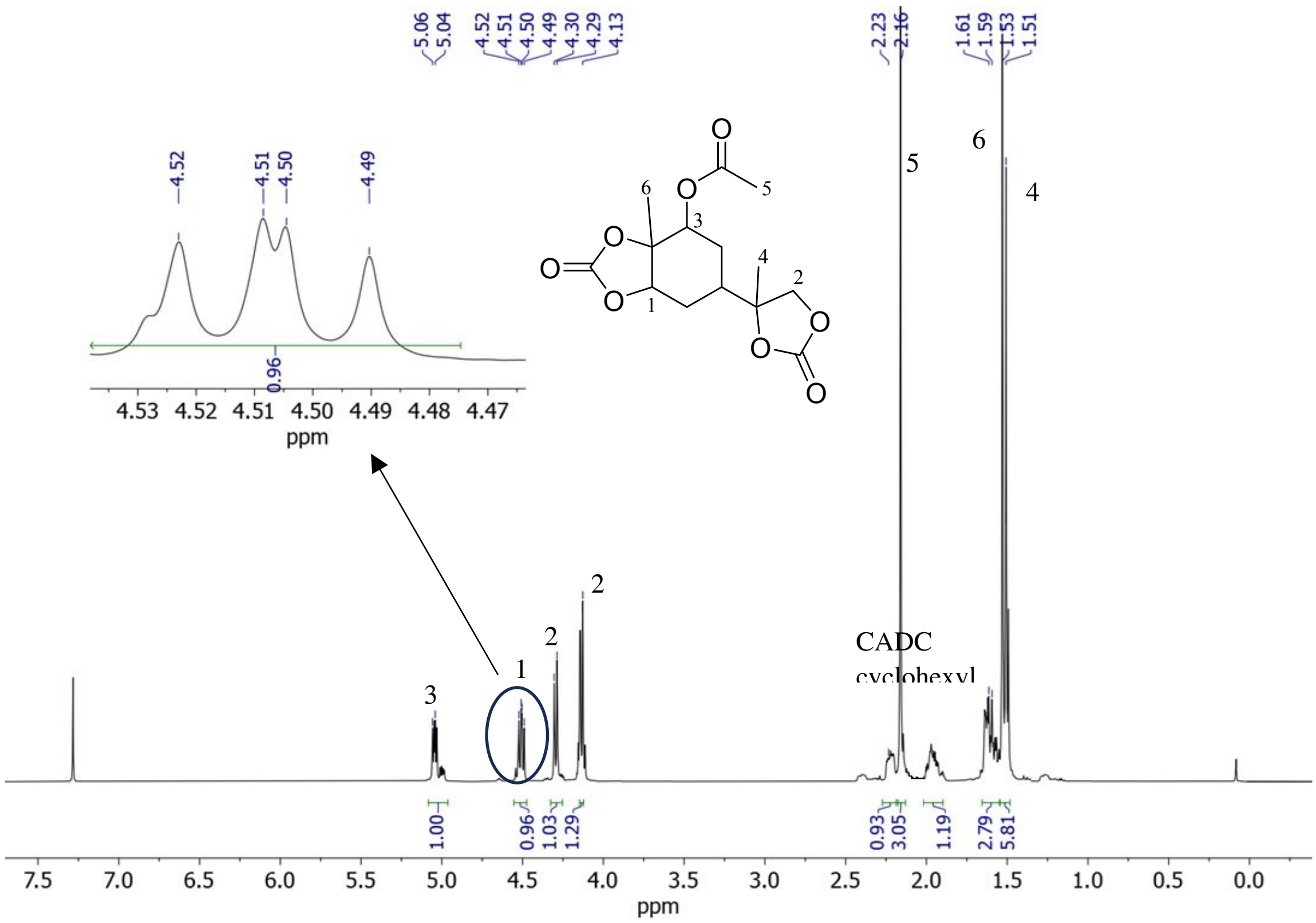

Figure S4. ^{1}H-NMR spectrum for *trans*-CADC

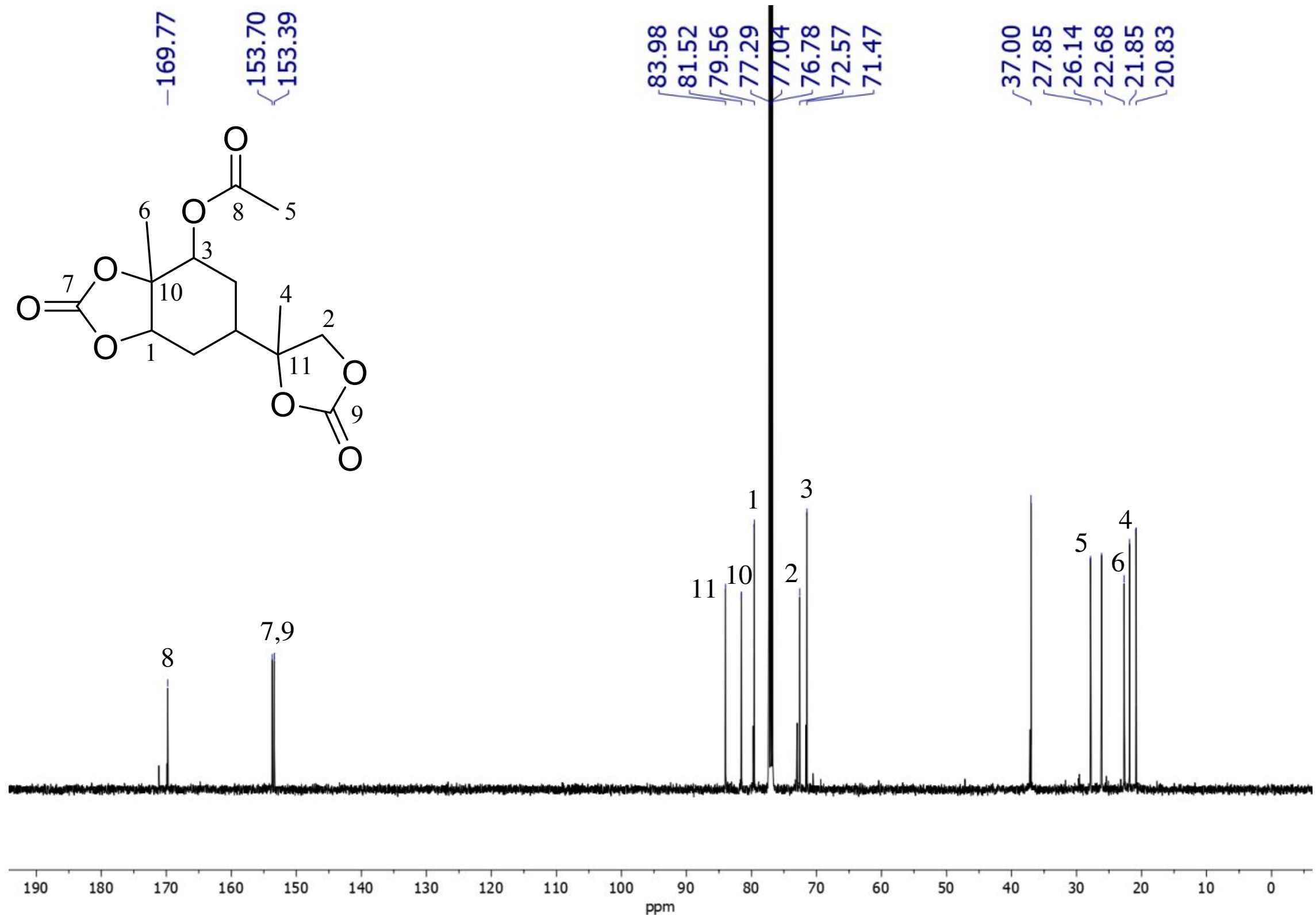

Figure S5. ^{13}C-NMR spectrum for *trans*-CADC

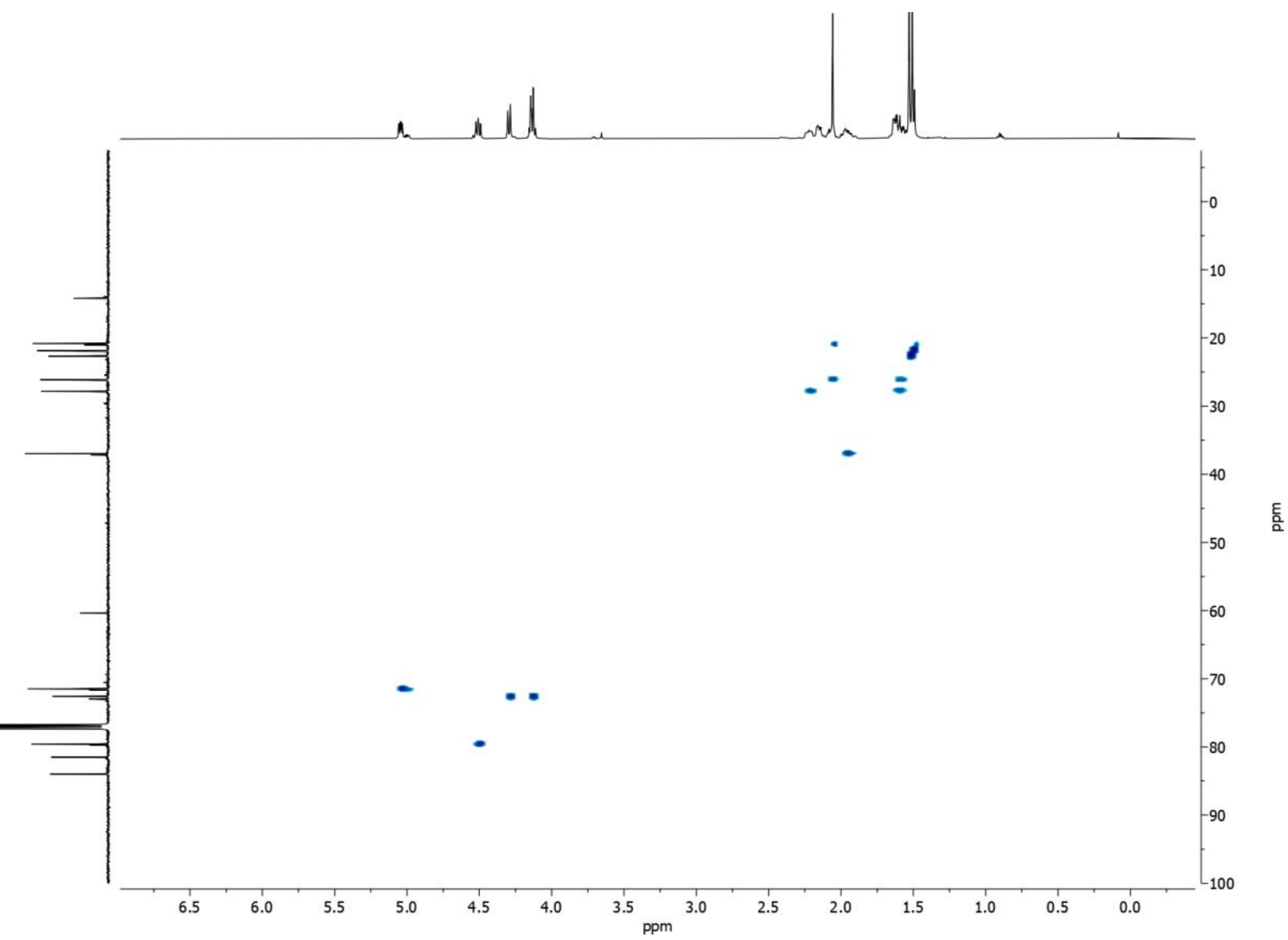

Figure S6. ^{1}H-^{13}C-NMR HSQC spectrum for *trans*-CADC

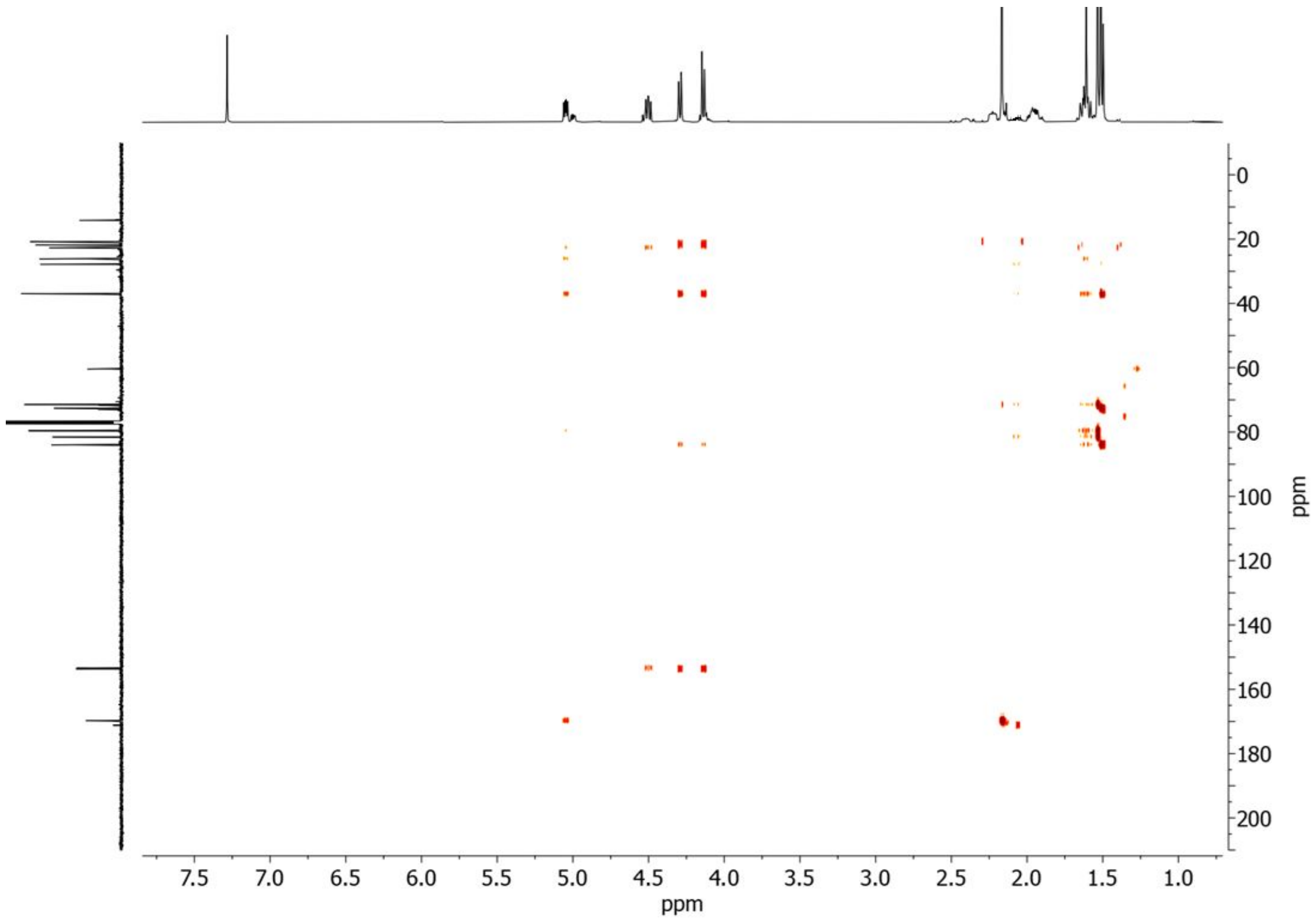

Figure S7. ^{1}H-^{13}C-NMR HMBC spectrum for *trans*-CADC

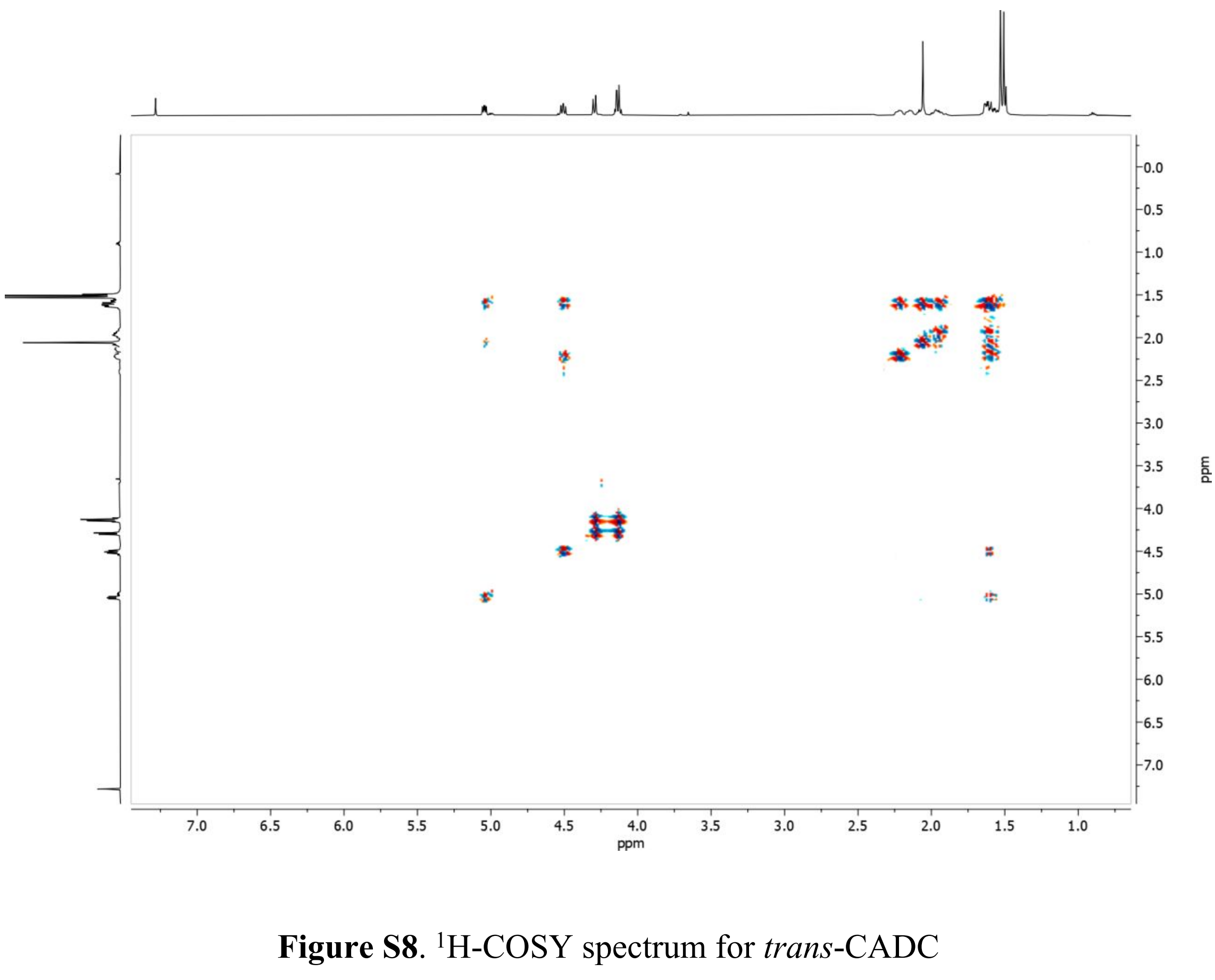

Figure S8. ^{1}H-COSY spectrum for *trans*-CADC

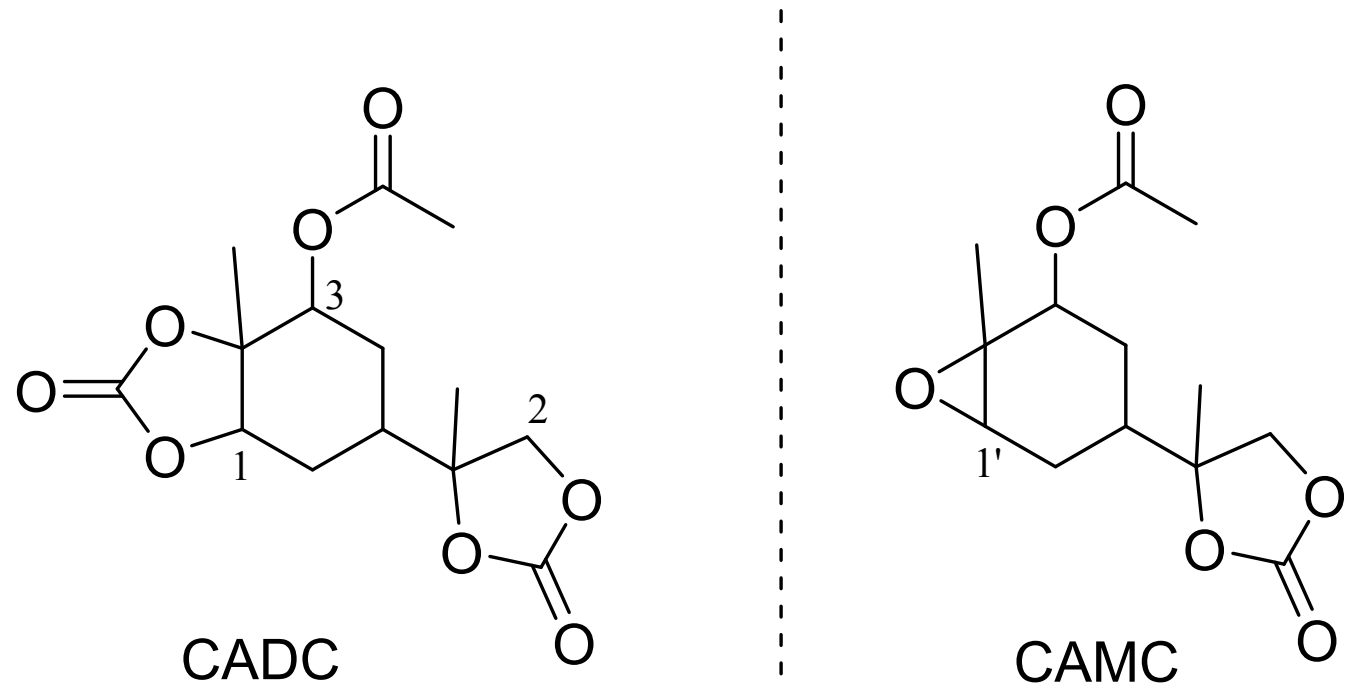

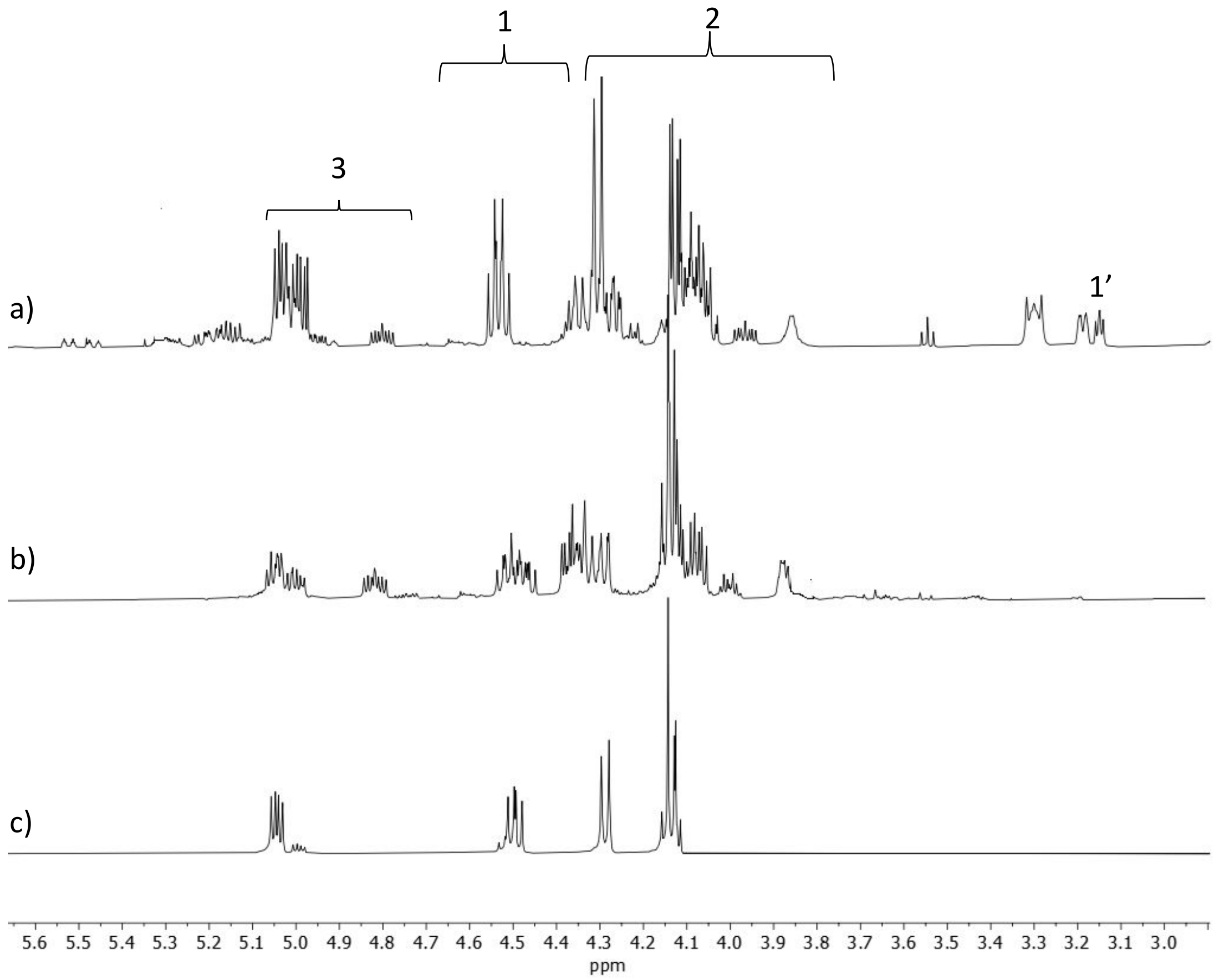

Figure S9. ^{1}H-NMR comparison between: a) crude mixture after carbonatation of CADE; b) purification after column chromatography; c) *trans*-CADC crystals obtained after crystallization in the range of epoxide and cyclic carbonate protons.

S12. Crystallographic data of CADC

Table S2. Crystal data and structure refinement for CADC.

Identification code	MMS_carvyl_sq
CCDC number	2516525
Empirical formula	$C_{14} H_{18} O_8$
Formula weight	314.28
Temperature	235(2) K
Wavelength	0.71073 Å
Crystal system	Orthorhombic
Space group	P 21 21 21
Unit cell dimensions	a = 6.9781(17) Å<br>b = 13.627(3) Å<br>c = 17.778(4) Å
Volume	1690.5(7) Å^3
Z	4
Density (calculated)	1.235 g/cm^3
Absorption coefficient	0.102 mm^{-1}
F(000)	664
Crystal size	0.320 x 0.150 x 0.032 mm^3
Theta range for data collection	2.736 to 26.372°
Index ranges	-8≤h≤8, -15≤k≤17, -21≤l≤22
Reflections collected	12686
Independent reflections	3456 [R(int) = 0.0878]
Completeness to theta = 25.242°	99.8 %
Absorption correction	Semi-empirical from equivalents
Max. and min. transmission	0.746 and 0.665
Refinement method	Full-matrix least-squares on F^2
Data / restraints / parameters	3456 / 0 / 204
Goodness-of-fit on F^2	0.959
Final R indices [I>2sigma(I)]	R1 = 0.0597, wR2 = 0.1425
R indices (all data)	R1 = 0.1384, wR2 = 0.1798
Absolute structure parameter	0(3)
Extinction coefficient	0.020(5)
Largest diff. peak and hole	0.199 and -0.159 e.Å^{-3}

Table S3. Selected bond lenghts for CADC

C(1)-O(3)	1.177(8)	C(8)-C(9)	1.516(8)
C(1)-O(1)	1.333(8)	C(9)-O(5)	1.446(7)
C(1)-O(2)	1.368(8)	C(11)-O(6)	1.200(8)
C(2)-O(2)	1.429(7)	C(11)-O(5)	1.340(8)
C(2)-C(3)	1.525(8)	C(11)-O(4)	1.351(8)
C(3)-O(1)	1.474(7)	C(13)-O(8)	1.197(7)
C(7)-O(7)	1.458(6)	C(13)-O(7)	1.356(7)
C(8)-O(4)	1.462(7)		

Table S4. Selected bond angles for CADC

O(3)-C(1)-O(1)	126.2(7)	C(11)-O(4)-C(8)	106.3(5)
O(3)-C(1)-O(2)	122.9(7)	C(11)-O(5)-C(9)	107.6(5)
O(1)-C(1)-O(2)	110.8(6)	O(8)-C(13)-O(7)	121.8(6)
C(1)-O(1)-C(3)	109.9(5)	O(8)-C(13)-C(14)	127.0(6)
C(1)-O(2)-C(2)	109.3(5)	O(7)-C(13)-C(14)	111.1(6)
O(6)-C(11)-O(5)	124.9(6)		
O(6)-C(11)-O(4)	123.5(7)		
O(5)-C(11)-O(4)	111.6(6)		

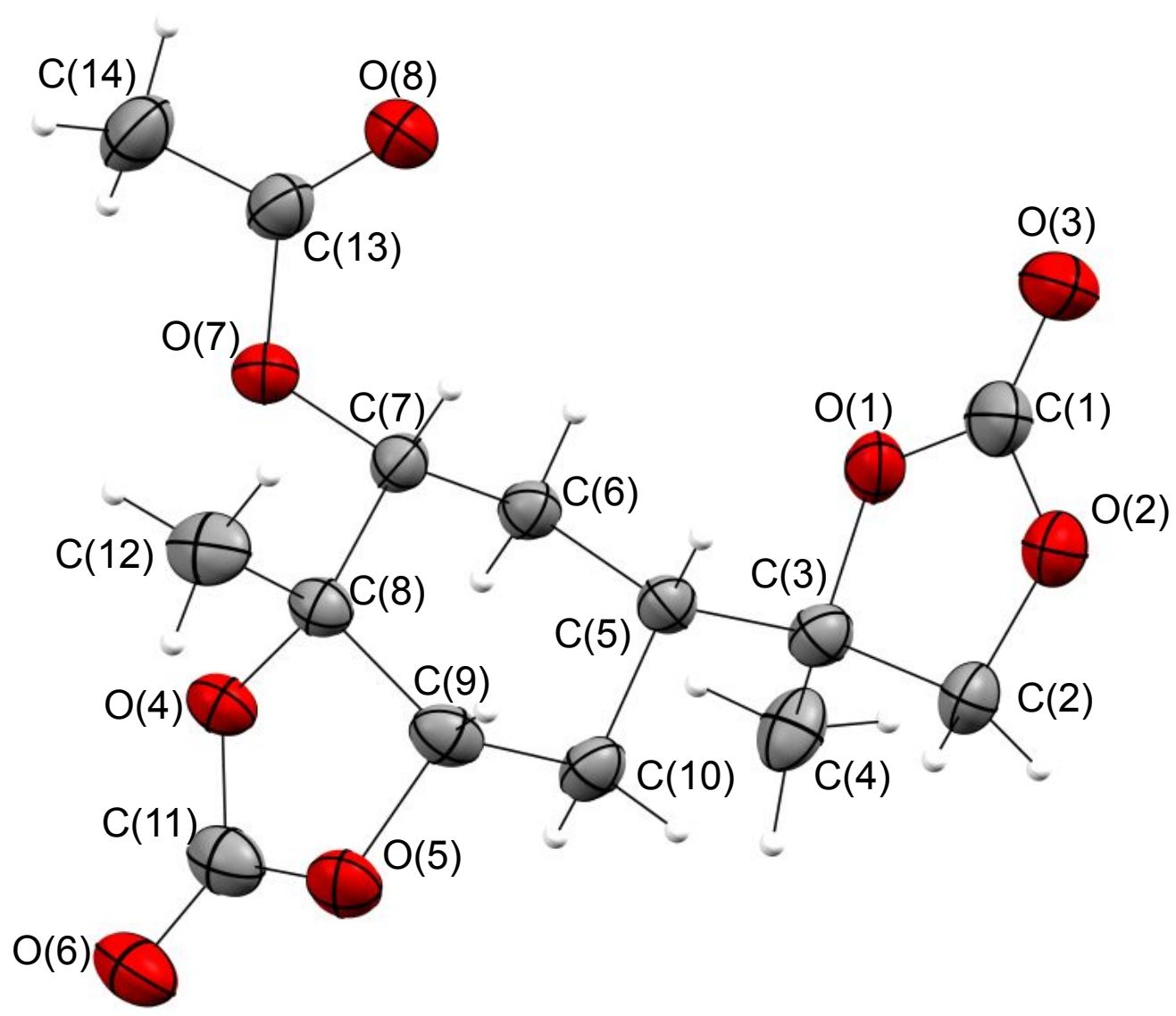

Figure S10. ORTEP view of *trans*-CADC.

S14. General procedure for hydroxycarbamates synthesis

In a 10 mL Schlenk flask equipped with a small stir bar, purified and crystallized CADC was added and dissolved in 2 mL of MeCN. Then, the corresponding diamine was added in a 1:0.5 and 1:2 molar ratio with respect to CADC respectively. The reaction mixture was then warmed up to 80 ºC and left stirring for 16 h. After that time, solvent was removed in vacuo and the residue was taken up with MeOH. The mixture was filtered, and MeOH removed to afford the corresponding carbamate compound as a white-brownish solid in 80−95% yield.

CADC + diaminobutane (1:0.5), carbamate 1a

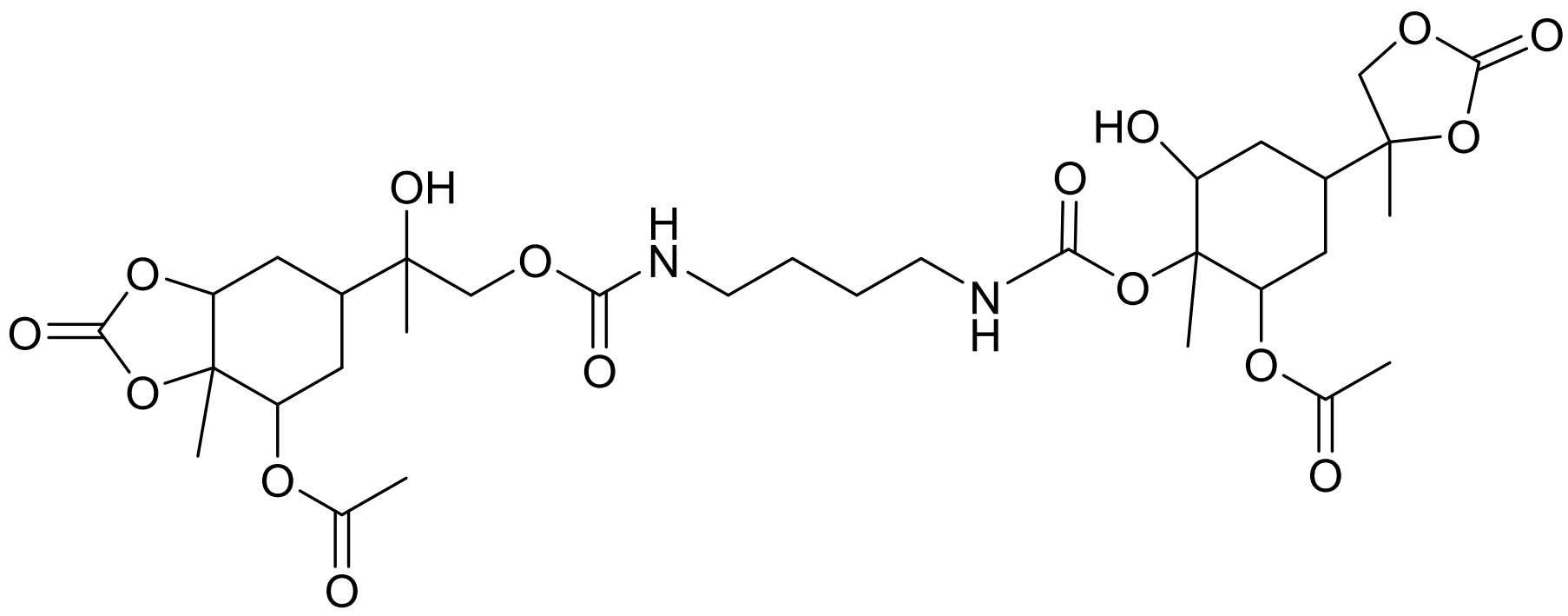

According to the general procedure, CADC (0.20 g, 0.64 mmol), 1,4-diaminobutane (32 µL, 0.32 mmol) and 2 mL of MeCN were added in a Schlenk tube and the mixture stirred at 80ºC for 16 h. After the appropriate work-up, the corresponding cyclic-carbonate ending carbamate prepolymer was obtained as a white solid (0.21 g, 90 %).

^{1}H-NMR (400MHz, DMSO-d_6): δ_H = 7.13 (s, $NH_{urethane}$), 5.12–5.03 (m, *carbonate CADC*), 4.80−4.30 (m, 5H), 4.25 (s, 1H), 3.94−3.74 (m, 2H), 3.41 (s, 2H), 3.03 (s, 4H, CH_2^{α}-$NH_{2,\text{diaminobutane}}$), 2.12 (s, 6H, CH_3-COO_{carvyl}), 1.78–1.46 (m, 16H, *CADC*, $CH_{3,\text{carvyl}}$; CH_2^{β}-$NH_{2,\text{diaminobutane}}$), 1.08z (s, 3H, $CH_{3,\text{carvyl}}$). **^{13}C-{^{1}H}-NMR** (100MHz, DMSO-d_6): δ_C = 170.3, 169.9, 156.7, 154.6, 154.3, 140.0, 85.6, 85.3, 82.4, 82.5, 80.9, 80.0, 73.0, 72.6, 71.8, 71.6, 69.3, 40.0, 38.3, 36.1, 28.0, 27.0, 26.4, 22.1, 22.0, 21.1. **IR** (neat) ν_{max} = 3308.1 (OH), 1794.8 (C=$O_{\text{carbonate}}$), 1708.3 (C=O_{urethane}), 1525.7 (NH_{urethane}), 1235.8 (C−O_{urethane}) cm^{-1}.

CADC + *m*-Xylene (1:0.5), carbamate 1b

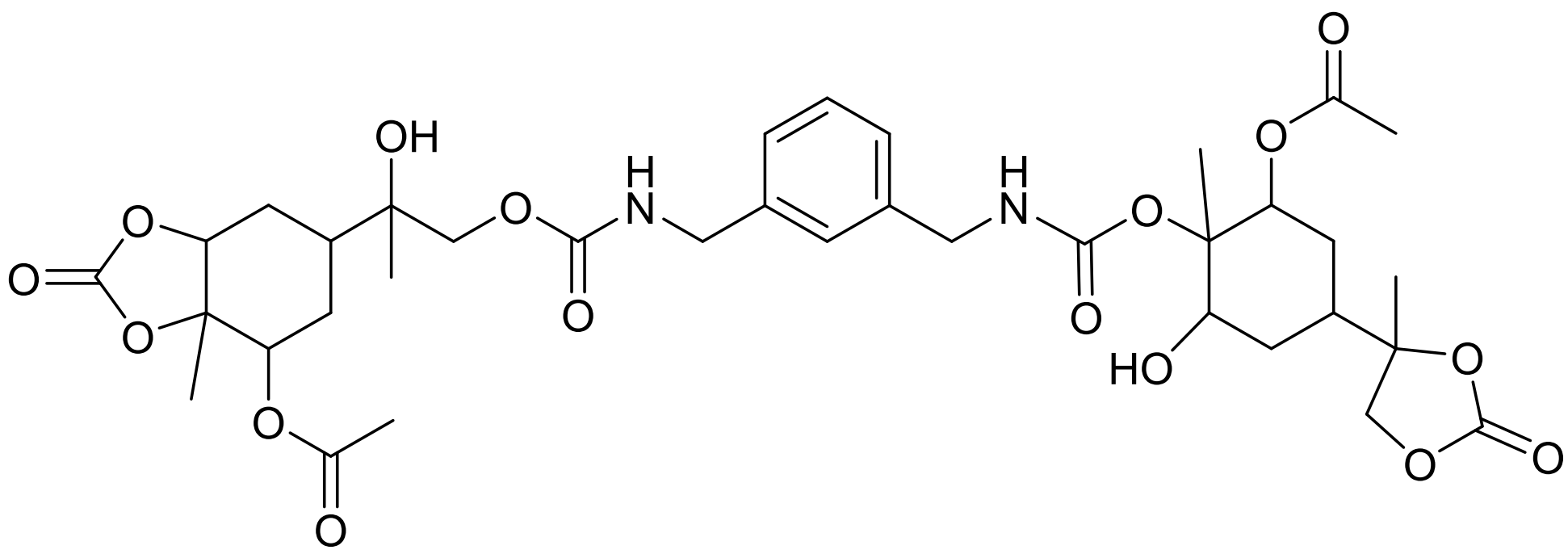

According to the general procedure, CADC (0.20 g, 0.64 mmol), *m*-Xylene (0.40 μL, 0.32 mmol) and 2 mL of MeCN were added in a Schlenk tube and the mixture stirred at 80ºC for 16 h. After the appropriate work-up, product was obtained as a white solid (0.22 g, 88 %).

^{1}H-NMR (400 MHz, DMSO-d$_6$): 7.65 (s, $NH_{urethane}$), 7.25−7.13 (m, 4H, $Ph_{m\text{-}xylene}$), 5.05–4.86 (m, *carbonate CADC*), 4.78−4.34 (m, 6H), 4.18 (m, 4H, CH_2^{α}-$NH_{m\text{-xylene}}$), 3.33 (s, 2H), 2.05 (s, 6H, CH_3-COO_{carvyl}), 1.59–1.40 (m, 12H, *CADC*, $CH_{3,carvyl}$), 1.04 (s, 6H, $CH_{3,carvyl}$). **^{13}C-{^{1}H}-NMR** (100MHz, DMSO-*d$_6$*): 170.3, 170.0, 157.0, 154.5, 144.6, 140.3, 128.7, 125.9, 85.5, 85.2, 82.5, 72.9, 71.9, 46.1, 42.3, 40.0, 36.1, 28.0, 26.2, 22.1, 21.7, 21.4, 21.1. **IR** (neat) ν_{max} = 3363.3 (OH), 1793.5 ($C{=}O_{carbonate}$), 1730.8 ($C{=}O_{urethane}$), 1540.9 ($NH_{urethane}$), 1225.2 ($C{-}O_{urethane}$) cm^{-1}.

CADC + diaminobutane (1:2), carbamate 2a

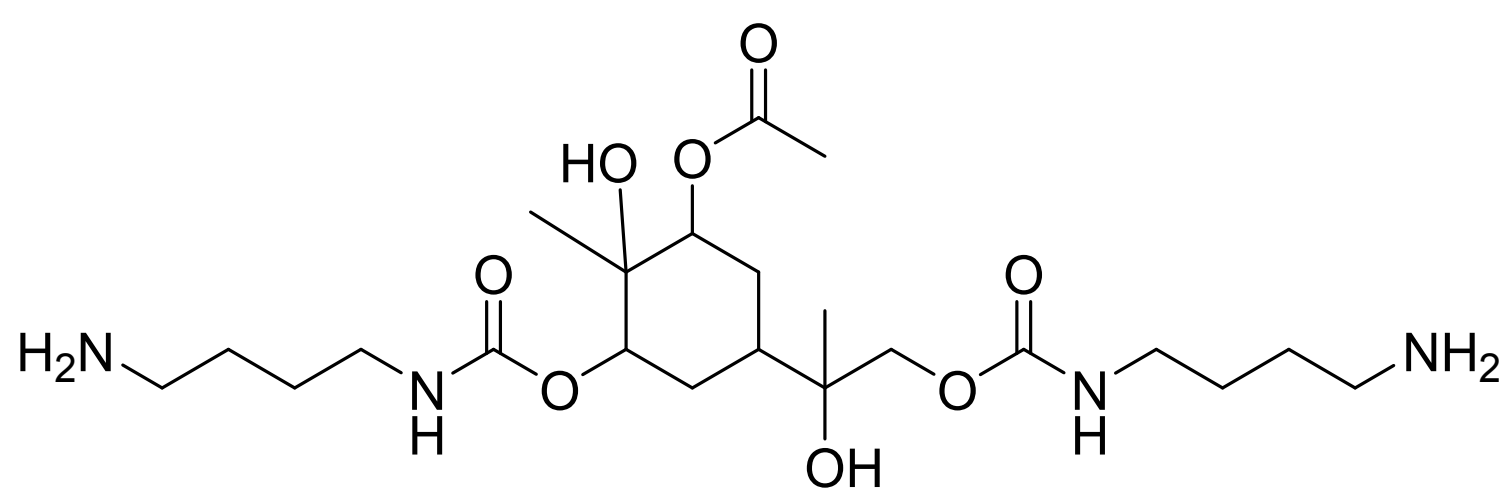

According to the general procedure, CADC (0.20 g, 0.64 mmol), 1,4-diaminobutane (0.13 mL, 1.28 mmol) and 2 mL of MeCN were added in a Schlenk tube and the mixture stirred at 80 ºC for 16 h. After the appropriate work-up, product was obtained as a white solid (0.27 g, 85 %).

^{1}H-NMR (400 MHz, DMSO-d_6): 7.07 (s, NH_{urethane}), 4.49−4.27 (m, 2H), 3.84−3.65 (m, 2H), 2.96 (m, 4H), 2.95 (m, 8H, CH_2^{α}-$NH_{\text{diaminobutane}}$), 2.04 (s, 3H, $CH_{3,\text{ carvyl}}$), 1.55−1.44 (m, 3H, *CADC*) 1.37 (s, 3H, $CH_{3,\text{ carvyl}}$), 1.39−1.28 (m, 8H, CH_2^{β}-$NH_{\text{diaminobutane}}$), 1.00 (s, 3H, $CH_{3,\text{ carvyl}}$). **^{13}C-{^{1}H}-NMR** (100MHz, DMSO-d_6): δ_C = 170.4, 169.3, 156.8, 76.4, 73.8, 72.9, 71.7, 69.6, 44.9, 42.1, 41.9, 39.9, 38.9, 38.7, 31.3, 31.1, 27.4, 27.2, 23.1, 22.5, 22.0, 21.3. **IR** (neat) ν_{max} = 3306.0 (OH), 1693.2 ($C{=}O_{\text{urethane}}$), 1536.9 (NH_{urethane}), 1242.7 ($C{-}O_{\text{urethane}}$) cm^{-1}.

CADC + *m*-Xylene (1:2), carbamate 2b

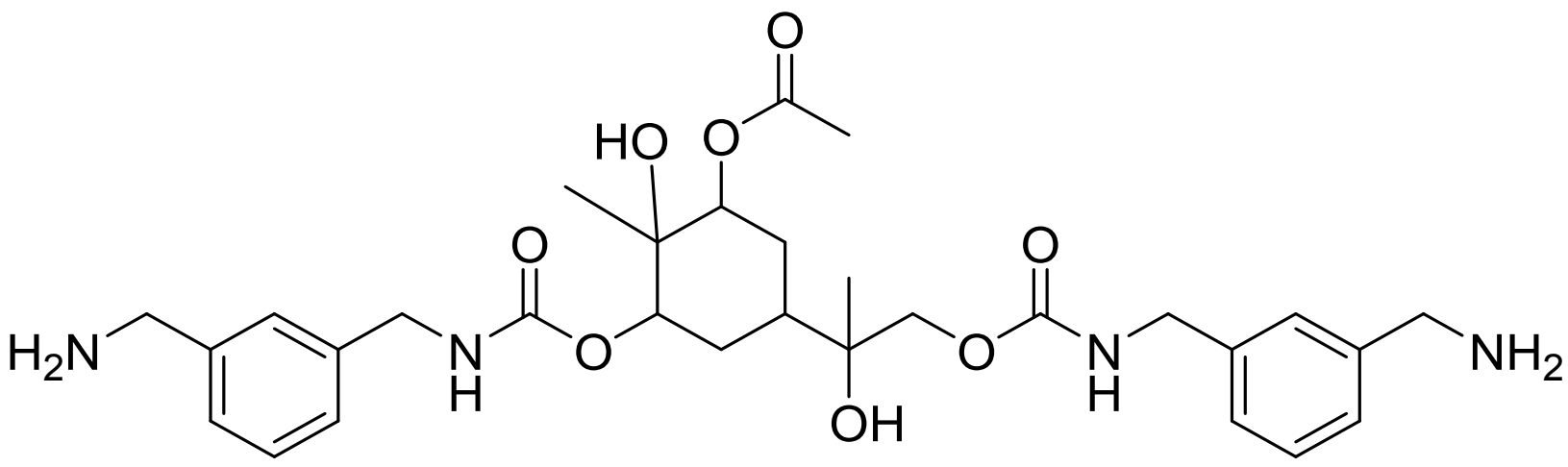

According to the general procedure, CADC (0.20 g, 0.64 mmol), *m*-Xylene (0.16 mL, 1.28 mmol) and 2 mL of MeCN were added in a Schlenk tube and the mixture stirred at 80ºC for 16 h. After the appropriate work-up, product was obtained as a white solid (0.34 g, 90%).

^{1}H-NMR (400 MHz, DMSO-d_6): 7.63 (s, NH_{urethane}), 7.28−7.10 (m, 8H, $Ph_{m\text{-}xylene}$), 4.75−4.41 (m, 2H), 4.23−4.16 (m, 4H, NHCO-CH_2^{α}-$NH_{m\text{-xylene}}$), 3.86 (m, 2H), 3.70 (m, 4H), 2.05 (s, 3H, $CH_{3,\text{ carvyl}}$), 1.88−1.45 (m, 5H), 1.45 (s, 3H, $CH_{3,\text{ carvyl}}$), 1.03 (s, 3H, $CH_{3,\text{ carvyl}}$). **^{13}C-{^{1}H}-NMR** (100MHz, DMSO-d_6): δ_C = 170.4, 169.5, 157.1, 156.9, 144.5, 144.0, 139.8, 128.5, 128.3, 126.2, 125.9, 125.3, 84.5, 79.7, 76.3, 73.9, 71.8, 70.0, 69.6, 49.1, 46.2, 46.1, 44.3, 42.7, 23.1, 22.0, 21.4, 21.3. **IR** (neat) ν_{max} = 3253.4 (OH), 1693.9 ($C{=}O_{\text{amide}}$), 1540.5 (NH_{amide}), 1245.1 ($C{-}O_{\text{urethane}}$) cm^{-1}

S.17 Possible regioisomers in the preparation of hydroxycarbamates 1b and 2b.

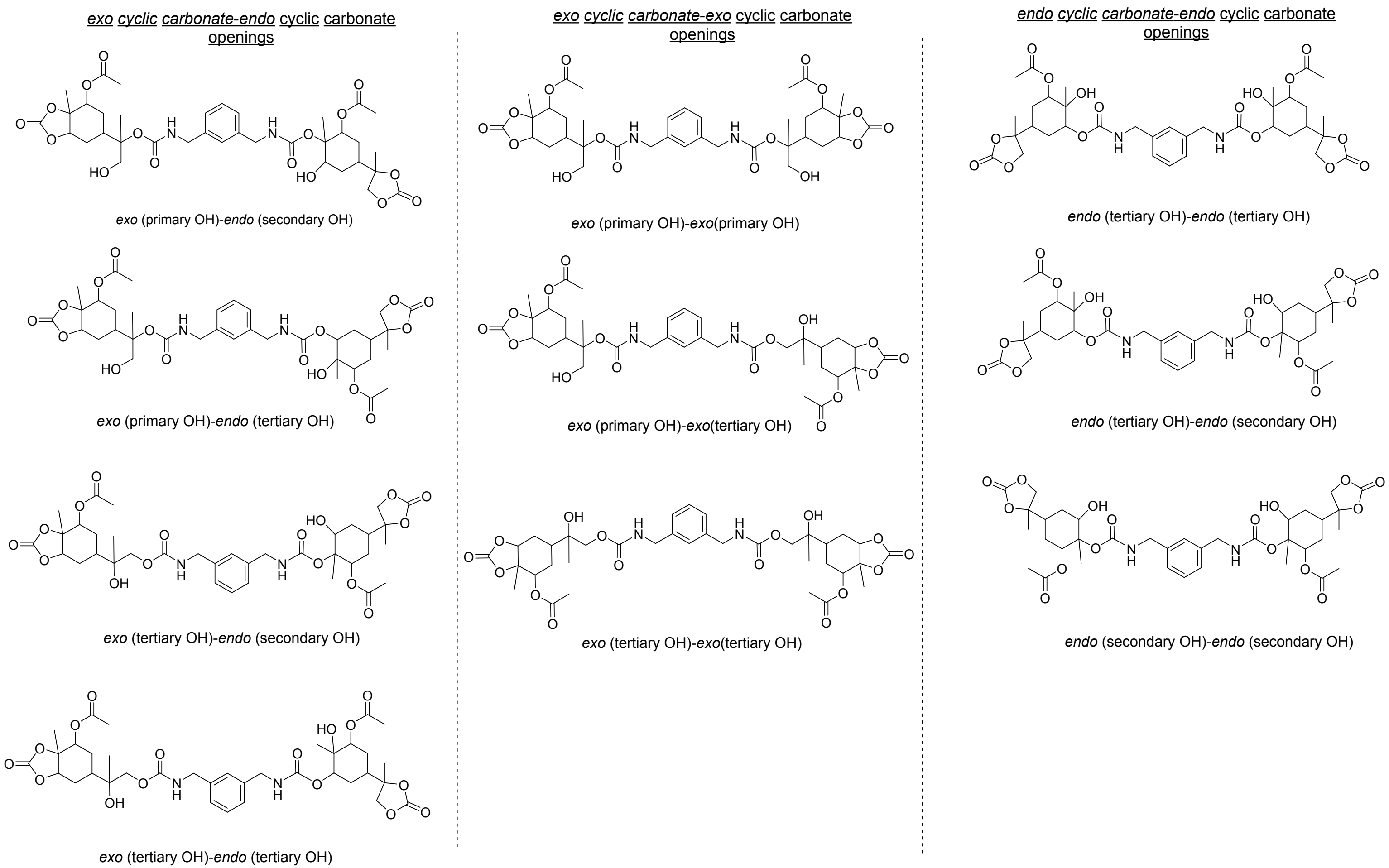

Figure S11. Possible regioisomers in the preparation of hydroxycarbamate **1b,** employing a 1:2 ratio of *m*-xylene diamine and *trans*-CADC.

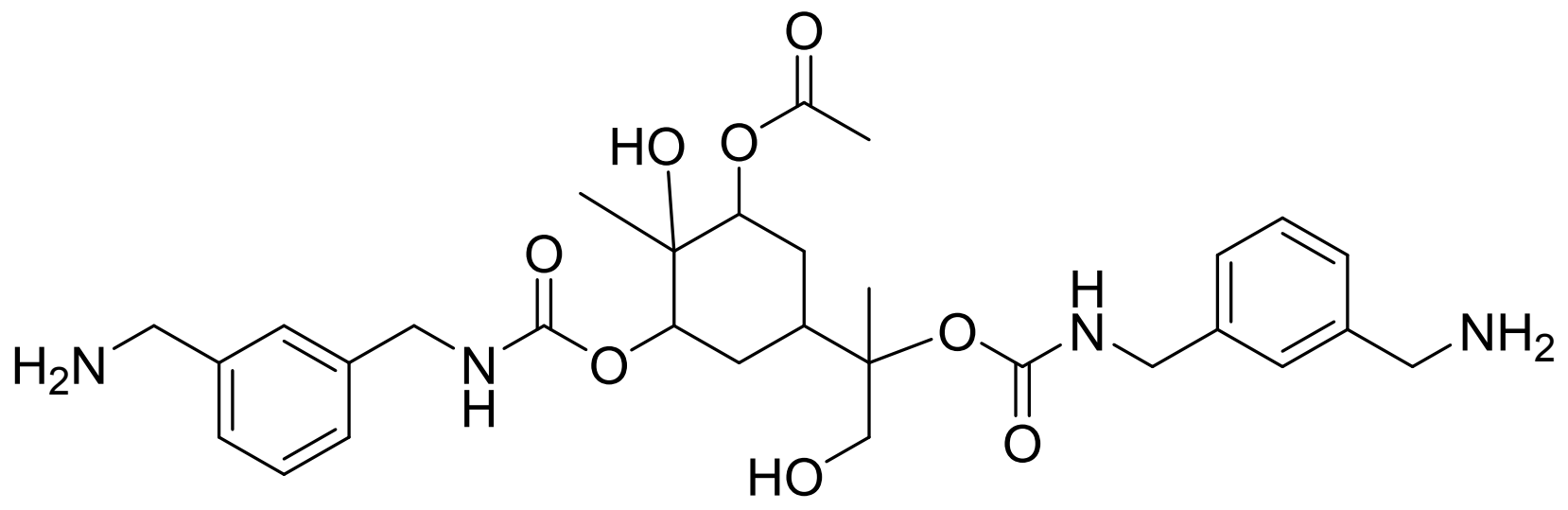

endo (tertiary OH)-*exo* (primary OH)

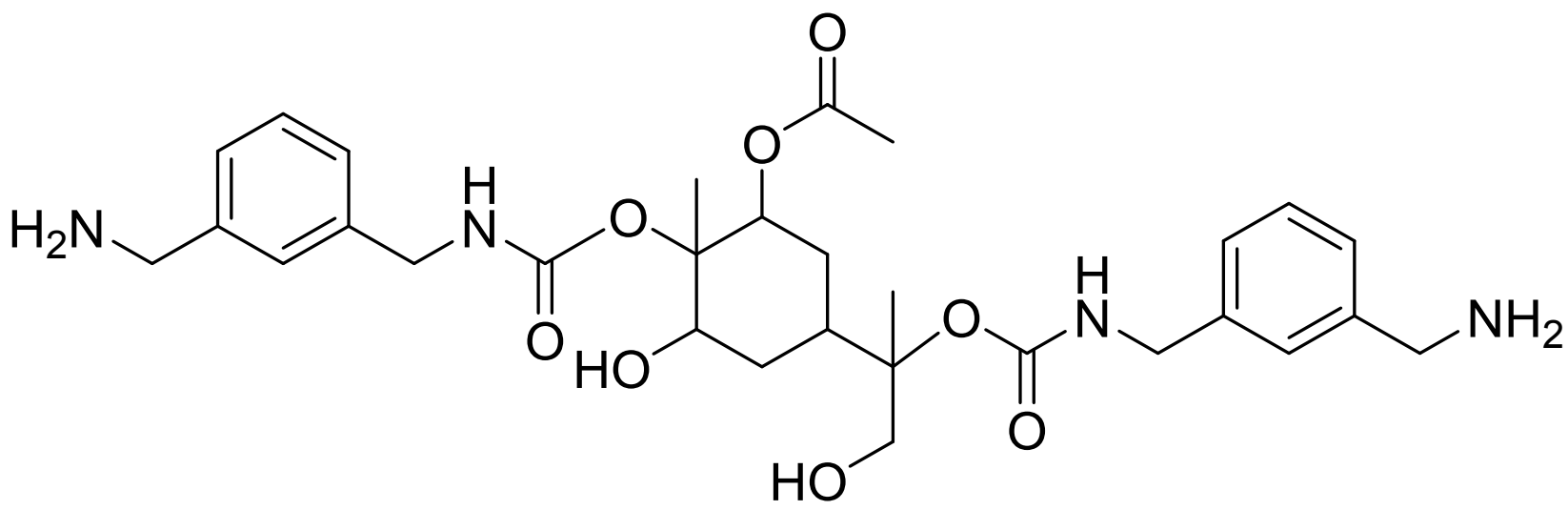

endo (secondary OH)-*exo* (primary OH)

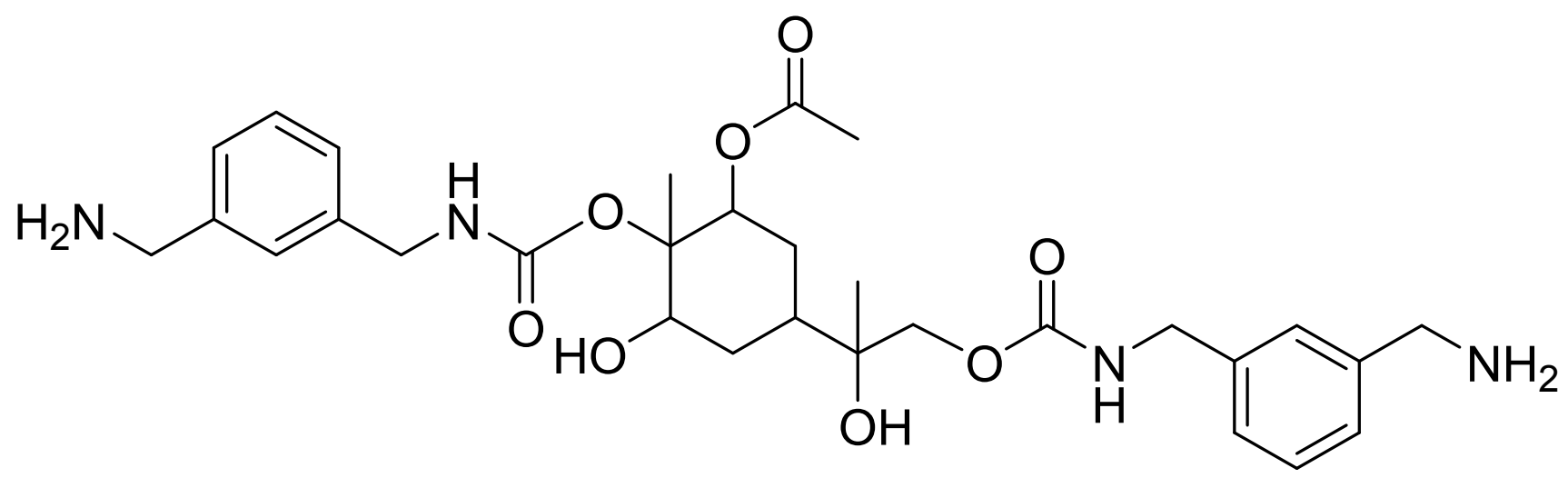

endo (secondary OH)-*exo* (tertiary OH)

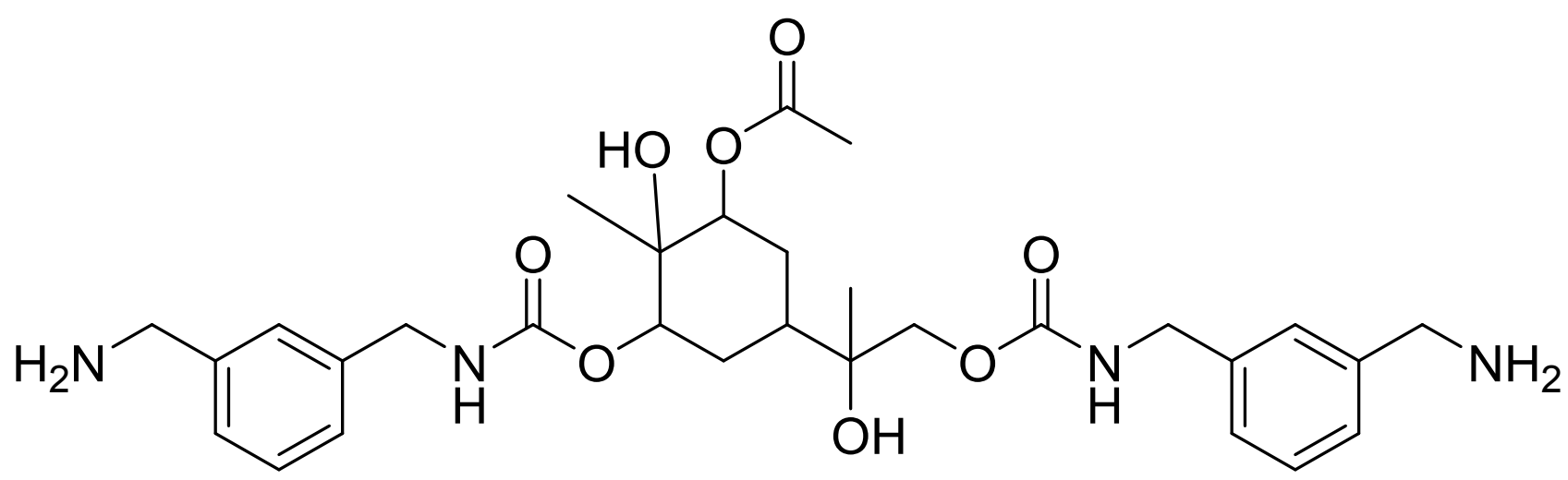

endo (tertiary OH)-*exo* (tertiary OH)

Figure S12. Possible regioisomers in the preparation of hydroxycarbamate **2b,** employing a 2:1 ratio of *m*-xylene diamine and *trans*-CADC (only *endo cylic carbonate*-*exo cyclic carbonate* possibilities can be formed).

S19. General Procedure for NIPU thermoplastics synthesis

In a 10 mL Schlenk flask equipped with a small stir bar, purified and crystallized CADC was added and dissolved in 2 mL of MeCN. Then, the corresponding diamine was added in a 1:1 molar ratio with respect to CADC. The reaction mixture was then warmed up to 80 ºC and left stirring for 16h. After that time, solvent was removed in vacuo and the residue Was taken up with MeOH. The mixture was filtered, and MeOH removed to afford the corresponding NIPU thermoplastic as a white-brownish solid in 80−90% yield.

CADC + 1,4-diaminobutane (1:1), NIPU 8

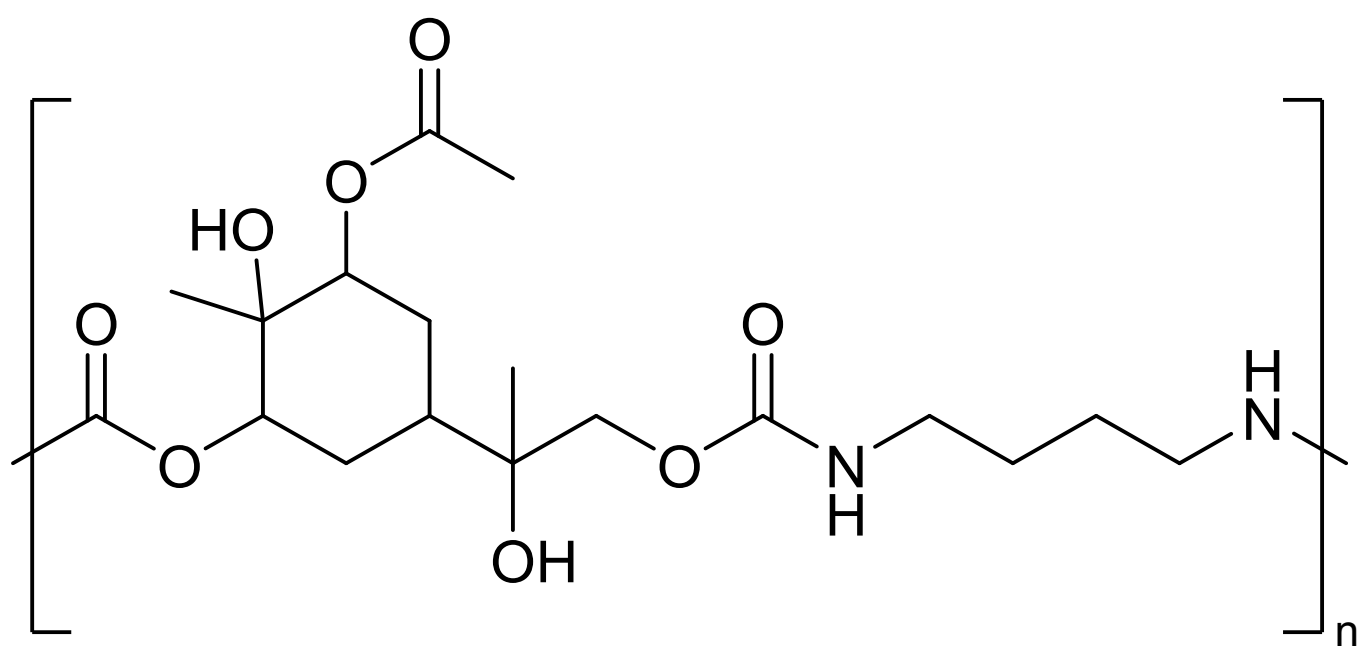

According to the general procedure, CADC (0.50 g, 1.60 mmol), 1,4-diaminobutane (0.16 mL, 1.60 mmol) and 2 mL of MeCN were added in a Schlenk tube and the mixture stirred at 80ºC for 16 h. After the appropriate work-up, product was obtained as a white solid (0.54 g, 84 %, M_n = 3.1 kg · mol^{-1} , Đ = 2.1).

^{1}H-NMR (400MHz, DMSO-d_6): δ_H = 7.13 (s, $NH_{urethane}$), 4.66−4.40 (m, 2H), 3.92−3.78 (m, 2H), 3.39−3.17 (m, 2H), 3.02 (s, 4H, CH_2^{α}-$NH_{diaminobutane}$), 2.11 (d, J_{H-H} = 4.1 Hz, 3H, $CH_{3,\ carvyl}$), 1.71−1.53 (m, 5H), 1.53−1.33 (s, 4H, CH_2^{β}-$NH_{diaminobutane}$), 1.46 (s, 3H, $CH_{3,\ carvyl}$), 1.06 (s, 3H, $CH_{3,\ carvyl}$). **^{13}C-{^{1}H}-NMR** (100MHz, DMSO-d_6): δ_C = 170.6; 170.4, 169.4, 157.9, 156.8, 74.9, 73.8, 72.9, 71.7, 71.6, 69.6, 30.4, 27.2, 23.1, 22.5, 21.5, 14.2. **IR** (neat) ν_{max} = 3307.2 (OH), 1696.2 ($C{=}O_{urethane}$), 1539.4 ($NH_{urethane}$), 1373.2 ($C{-}N_{urethane}$), 1245.4 ($C{-}O_{urethane}$) cm^{-1}.

CADC + *L*-lysine (1:1), 1eq. TBD, NIPU 9

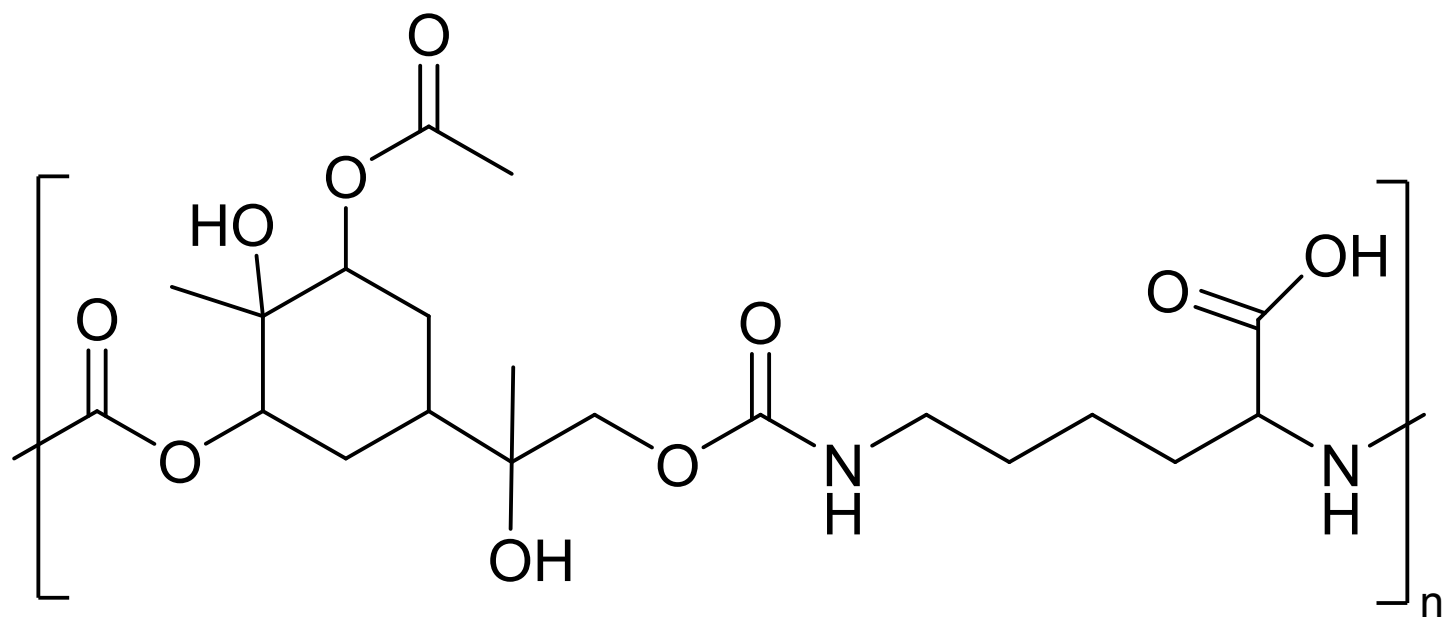

According to the general procedure, CADC (0.50 g, 1.60 mmol), *L*-lysine (0.24 g, 1.60 mmol), 1,5,7-triazabicyclo[4.4.0]dec-5-ene (0.23 g, 1.60 mmol) and 2 mL of MeCN were added in a Schlenk tube and the mixture stirred at 80 ºC for 16 h. After the appropriate work-up, product was obtained as a white solid (0.60 g, 81 %, Mn = 22.4 kg · mol^{-1} , D = 2.8).

^{1}H-NMR (400 MHz, DMSO-d_6): 10.44 ($COOH_{L\text{-lysine}}$), 6.97 (s, 1H, $NH_{urethane}$), 6.23 (s, 1H, NH_{urea}), 4.90–4.09 (m, 6H, *opening CABC*), 3.69 (m, 1H, CH^{α}-$NH_{L\text{-lysine}}$), 3.55 (s, 2H), 3.27 (s, 2H), 3.17 (d, J_{HH} = 5.2 Hz, 4H, *TBD*), 3.06 (d, J_{HH} = 4.8 Hz, 4H, *TBD*), 2.85 (s, 4H, CH_2^{α}-$NH_{L\text{-lysine}}$), 1.95 (s, 3H, $CH_{3,carvyl}$), 1.79 (d, J_{HH} = 4.7 Hz, 4H, *TBD*), 1.58 (s, 3H, $CH_{3,carvyl}$), 1.31 (m, 4H, CH_2^{β}-$NH_{L\text{-lysine}}$), 1.12 (s, 2H, CH_2^{γ}-$NH_{L\text{-lysine}}$), 0.93 (s, 3H, $CH_{3,carvyl}$). **^{13}C-{^{1}H}-NMR** (100 MHz, DMSO-d_6): 176.9, 170.5, 170.4, 169.3, 156.8, 154.8, 151.6, 77.0, 75.7, 73.6, 73.2, 73.0, 72.4, 71.8, 70.8, 69.7, 69.3, 55.9, 46.4, 40.9, 37.6, 32.8, 30.0, 25.4, 23.1, 22.5, 22.0, 21.4, 21.0. **IR** (neat) ν_{max} = 3460.4 (OH), 1692.7 ($C{=}O_{urethane}$), 1645.0 (NH_{urea}), 1551.5 ($NH_{urethane}$), 1259.1 ($C{-}O_{urethane}$) cm^{-1}.

CADC + *m*-xylylenediamine (1:1), NIPU 10

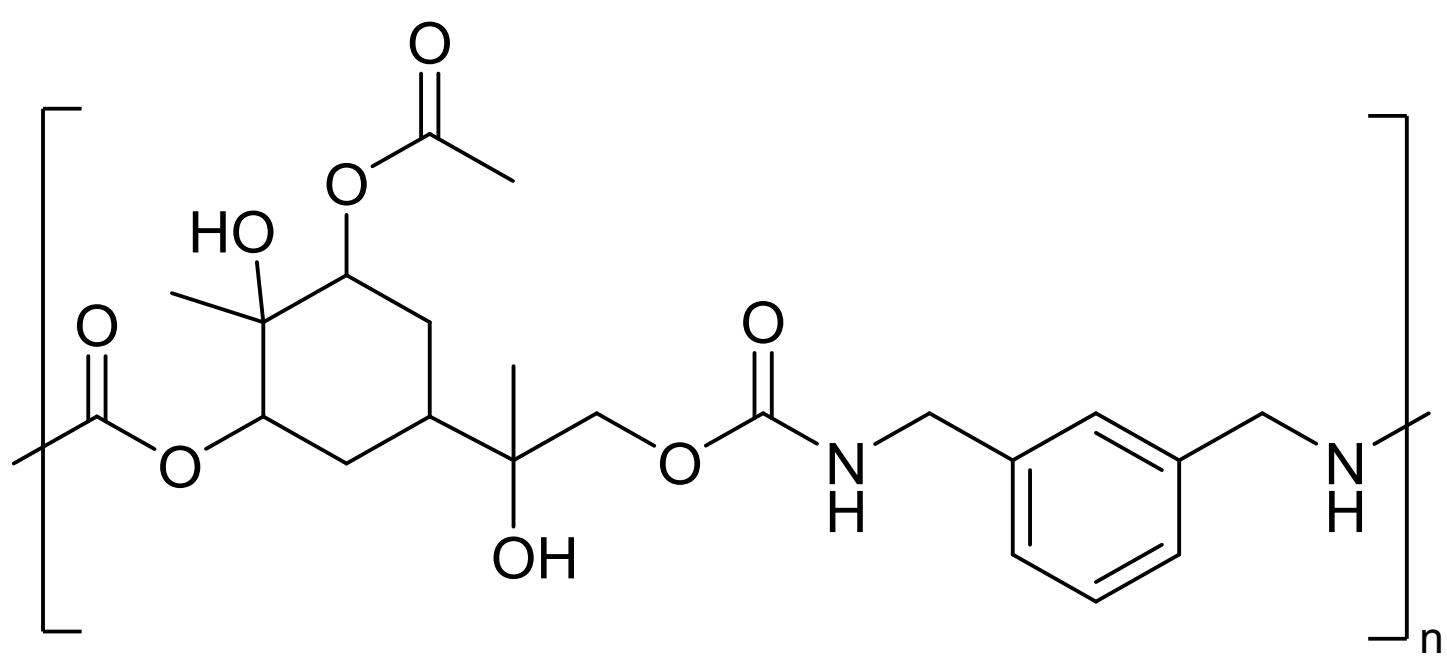

According to the general procedure, CADC (0.50 g, 1.60 mmol), m-xylylenediamine (0.21 mL, 1.60 mmol) and 2 mL of MeCN were added in a Schlenk tube and the mixture stirred at 80ºC for 16 h. After the appropriate work-up, product was obtained as a white solid (0.60 g, 81 %, M_n = 4.5 kg · mol^{-1} , D = 1.9).

^{1}H-NMR (400 MHz, DMSO-d_6): 7.63 (s, $NH_{urethane}$), 7.28−7.13 (m, 4H, $Ph_{m\text{-}xylene}$), 4.83−4.35 (m, 2H), 4.24−3.70 (m, 4H, $CH_2{}^{\alpha}$-$NH_{m\text{-xylene}}$), 3.85 (m, 2H), 3.49−2.89 (m, 2H), 2.03 (s, 3H, $CH_{3,\ carvyl}$), 1.39 (s, 3H, $CH_{3,\ carvyl}$), 1.04 (s, 3H, $CH_{3,\ carvyl}$). **^{13}C-{^{1}H}-NMR** (100MHz, DMSO-d_6): 170.6, 169.5, 157.1, 154.8, 144.2, 140.1, 139.8, 128.7, 128.3, 126.0, 125.4, 85.9, 73.6, 73.0, 72.0, 69.9, 46.1, 44.3, 42.6, 23.0, 22.5, 21.6, 21.4. **IR** (neat) ν_{max} = 3294.2 (OH), 1699.2 (C=$O_{urethane}$), 1535.5 ($NH_{urethane}$), 1372.9 (C–$N_{urethane}$), 1242.9 (C–$O_{urethane}$) cm^{-1}.

CADC + Jeffamine (1:1), NIPU 11

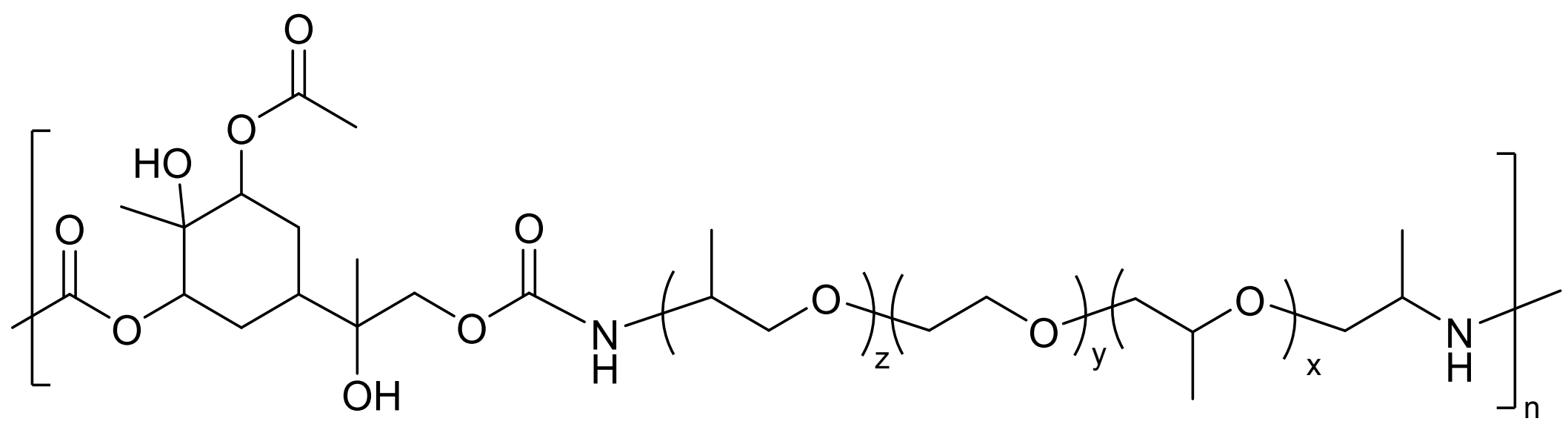

According to the general procedure, CADC (1 g, 1.60 mmol), Jeffamine® (6.4 g, 1.6 mmol) and 2mL of MeCN were added in a Schlenk tube and the mixture stirred at 80ºC for 16h. After the appropriate work-up, product was obtained as a white solid (7.1 g, 93%, M_n = 7.8 kg · mol^{-1} , Đ = 1.8)

^{1}H-NMR (400 MHz, DMSO-d_6): 6.88 (s, $NH_{urethane}$), 4.94−4.19 (m, 6H), 3.52 (bs, Jeffamine®), 3.41 (bs, Jeffamine®), 2.07 (s, 3H, $CH_{3,carvyl}$), 1.67−1.34 (m, 6H), 1.42 (s, 3H, $CH_{3,carvyl}$), 1.05 (bs, Jeffamine®), 0.98 (s, 3H, $CH_{3,carvyl}$). **^{13}C-{^{1}H}-NMR** (100MHz, DMSO-d_6): 170.4, 156.1, 154.7, 143.9, 143.6, 143.4, 113.4, 113.2, 85.4, 84.3, 82.5, 82.4, 81.2, 81.1, 80.8, 78.5, 74.7, 72.8, 70.5, 70.3, 68.3, 66.0, 62.0, 46.7, 44.9, 44.7, 23.1, 22.6, 22.0, 20.7, 19.3, 18.6, 18.0, 17.6. **IR** (neat) ν_{max} = 3464.5 (OH), 1714.4 (C=$O_{urethane}$), 1540.9 ($NH_{urethane}$), 1342.2 (C–$N_{urethane}$), 1241.0 (C–$O_{urethane}$), 1098.3 cm^{-1}.

CADC + tris-(2-aminoethyl)amine (1:1), NIPU 12

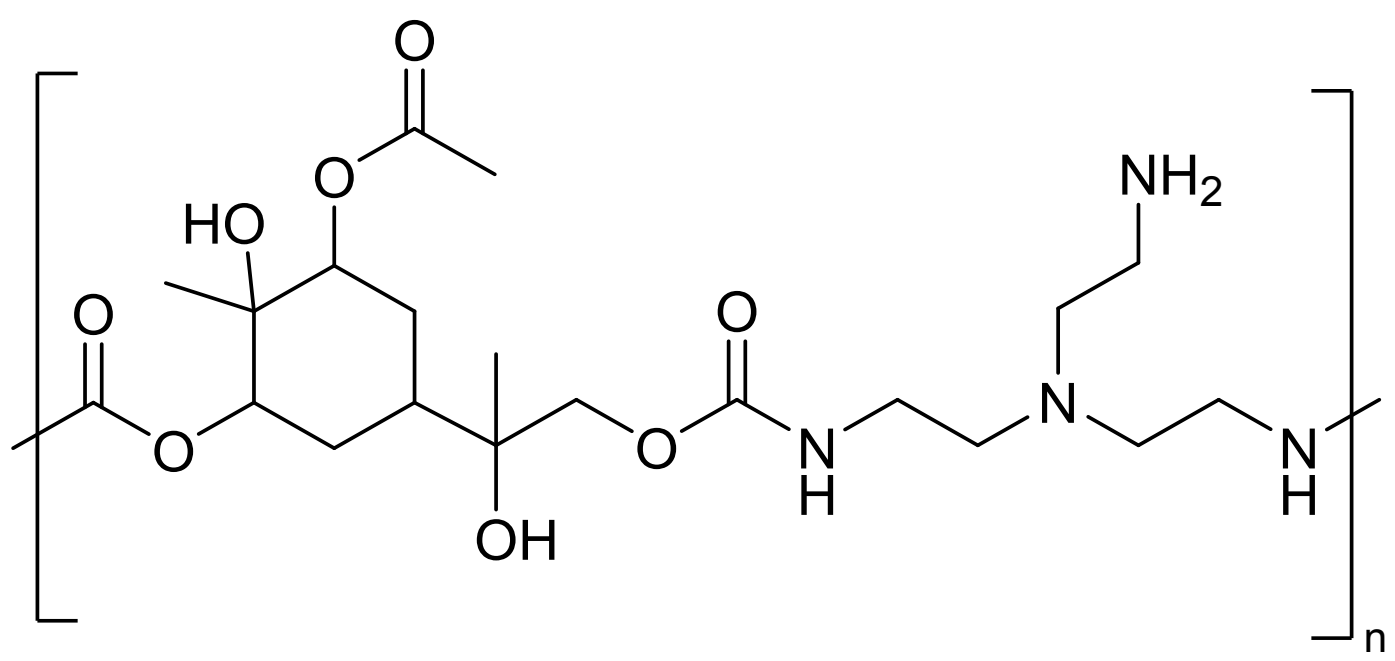

According to the general procedure, CADC (0.50 g, 1.60 mmol), tris-(2-aminoethyl)amine (0.24 mL, 1.60 mmol) and 2mL of MeCN were added in a Schlenk tube and the mixture stirred at 80ºC for 16 h. After the appropriate work-up, product was obtained as a white solid (0.65 g, 88 %, M_n = 2.5 kg · mol^{-1}, Đ = 1.7).

^{1}H-NMR (400 MHz, DMSO-d_6): 7.08–6.74 (s, $NH_{urethane}$), 4.74–4.29 (m, *CADC*), 3.81–3.77 (m, 2H), 3.02 (m, 4H, CH_2^{α}-$NH_{urethane}$), 2.56–2.37 (m, 3H), 2.56 (m, 2H, CH_2^{α}-$NH_{triamine}$), 2.40 (m, 4H, CH_2^{β}-$NH_{urethane}$), 2.35 (m, 2H, CH_2^{β}-$NH_{triamine}$), 2.08 (s, 2H), 2.04 (s, 3H, $CH_{3,carvyl}$), 1.56 (s, 3H, $CH_{3,carvyl}$), 1.01 (s, 3H, $CH_{3,carvyl}$). **^{13}C-{^{1}H}-NMR** (100MHz, DMSO-d_6): 170.4, 169.6, 169.5, 156.9, 156.7, 128.3, 126.2, 125.3, 118.5, 73.7, 72.9, 71.8, 71.7, 69.5, 58.4, 58.3, 54.1, 40.2, 40.0, 37.8, 37.6, 23.1, 22.5, 22.0, 21.3. **IR**: ν_{max} = 3279.4 (OH), 1689.9 ($C{=}O_{urethane}$), 1538.4 ($NH_{urethane}$), 1373.4 ($C–N_{urethane}$), 1247.3 ($C–O_{urethane}$) cm^{-1}.

S23. FT-IR spectra of hydroxycarbamates 1,2a and 1,2b

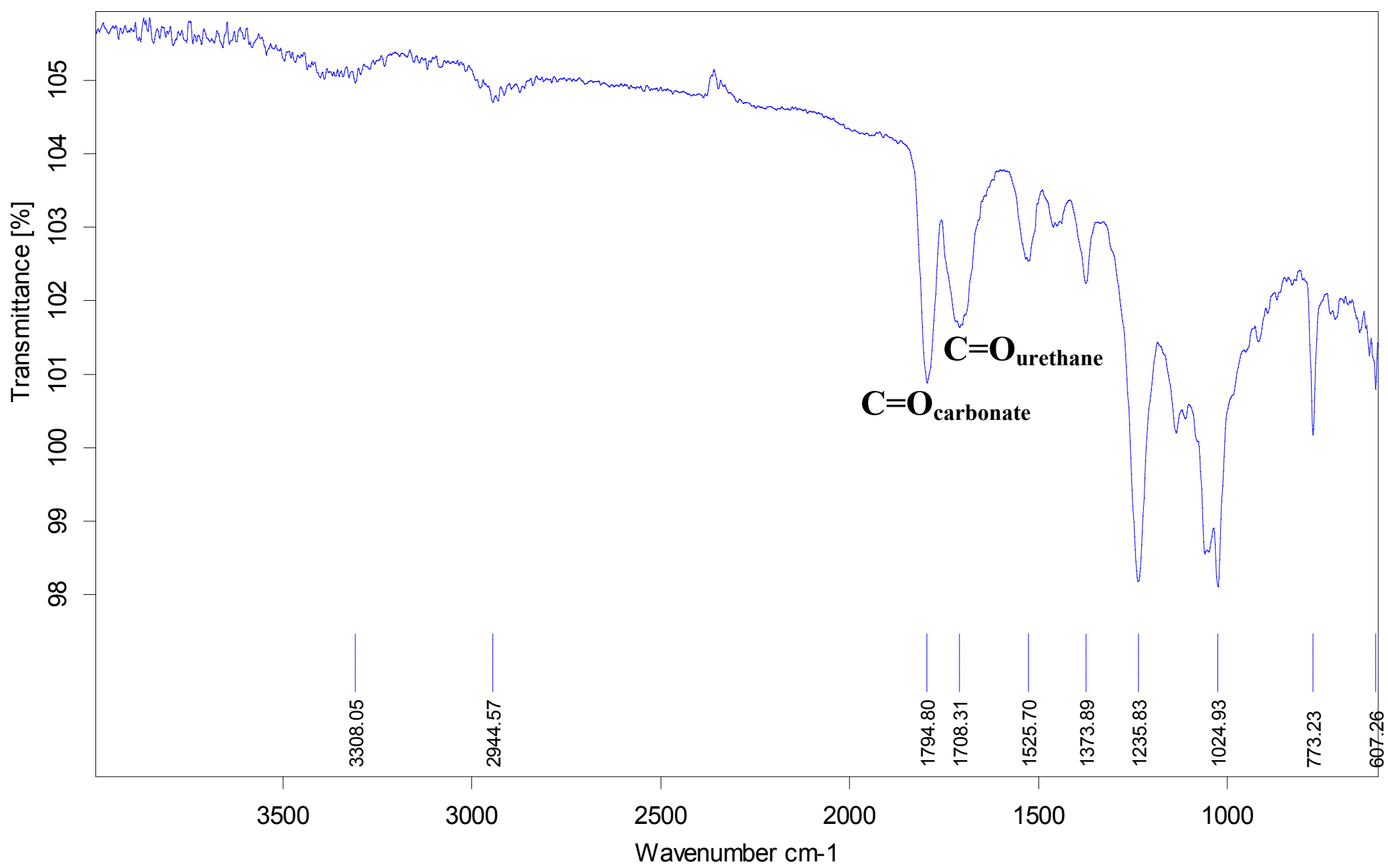

Figure S13. FT-IR spectrum for carbamate **1a**

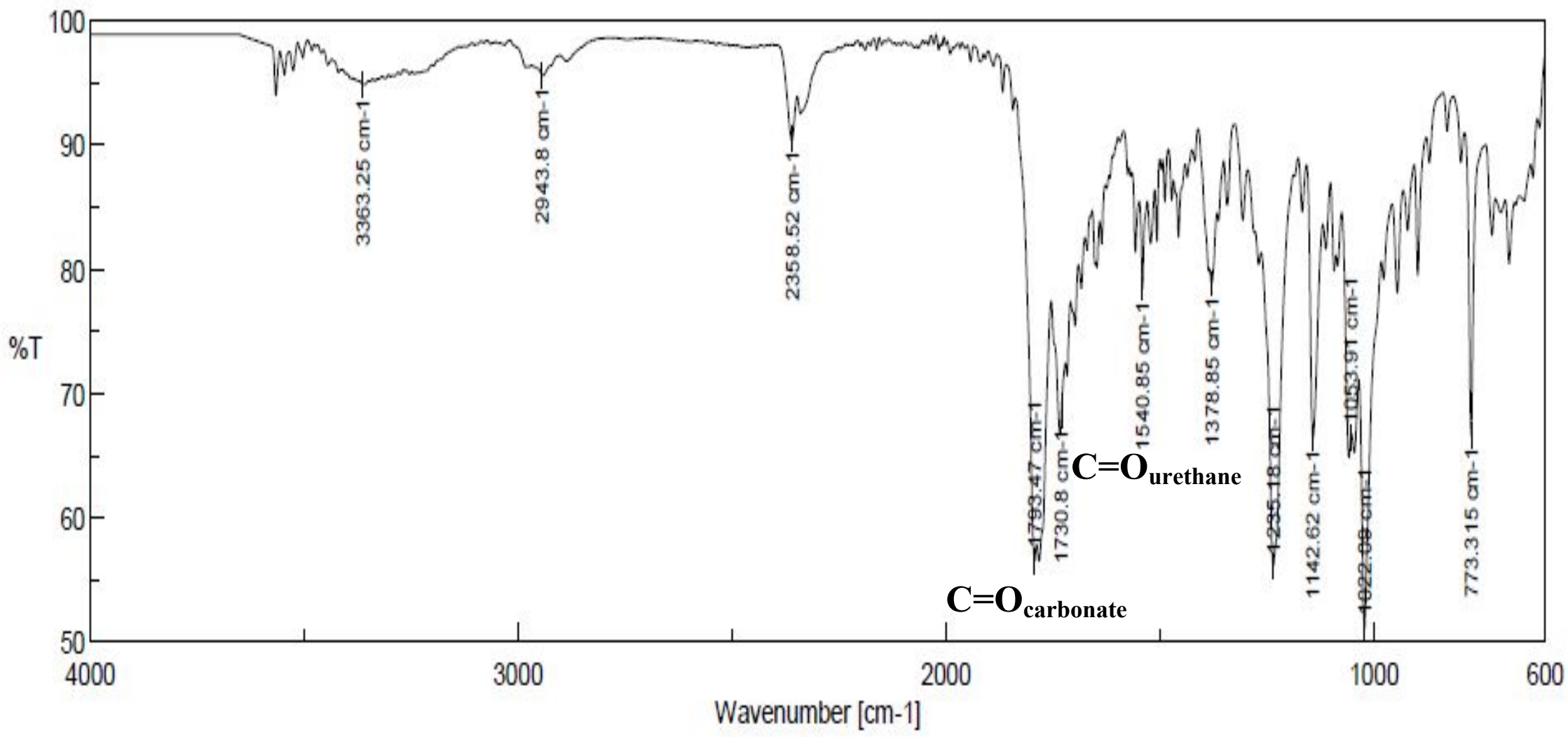

Figure S14. FT-IR spectrum for carbamate **1b**

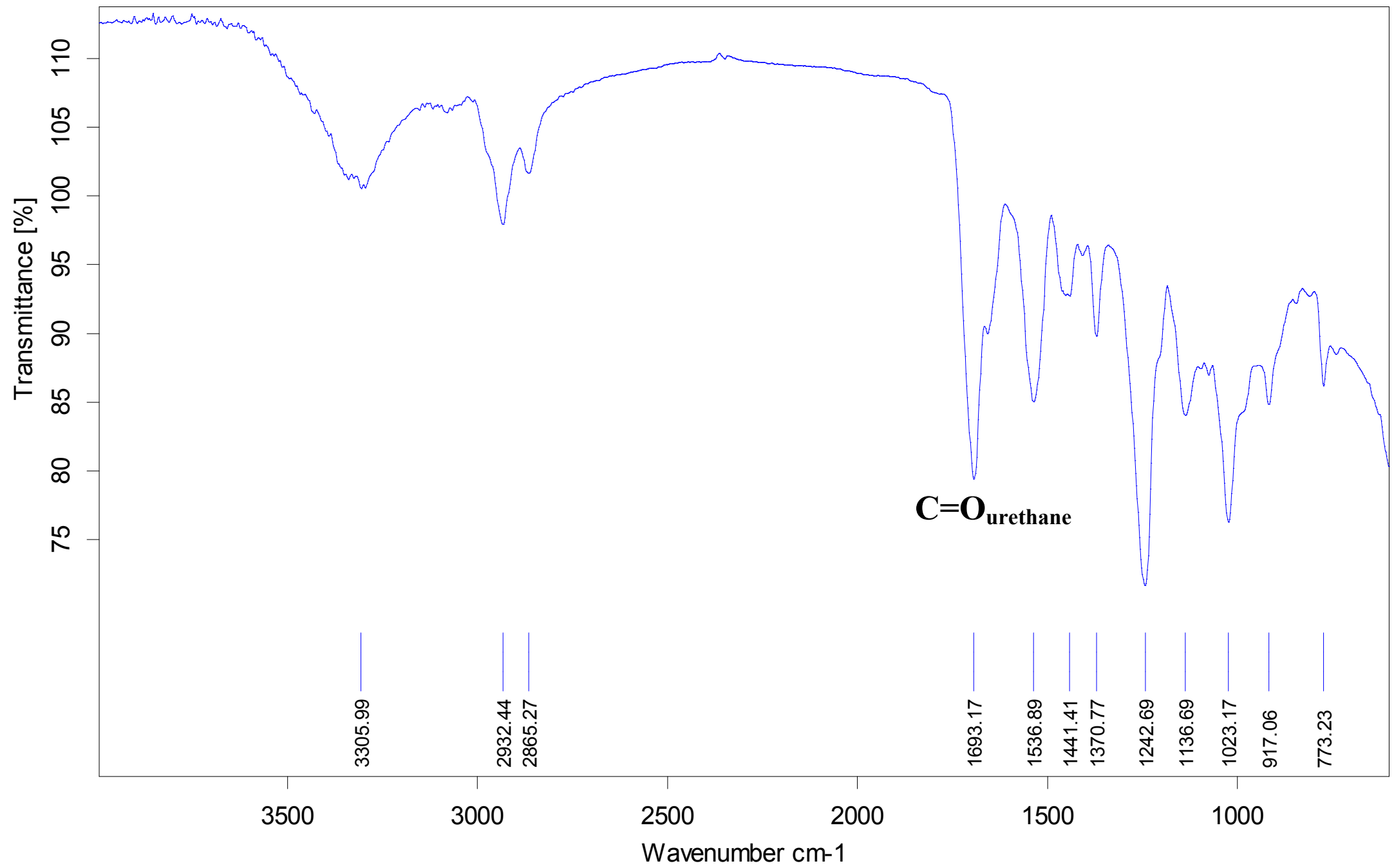

Figure S15. FT-IR spectrum for carbamate **2a**

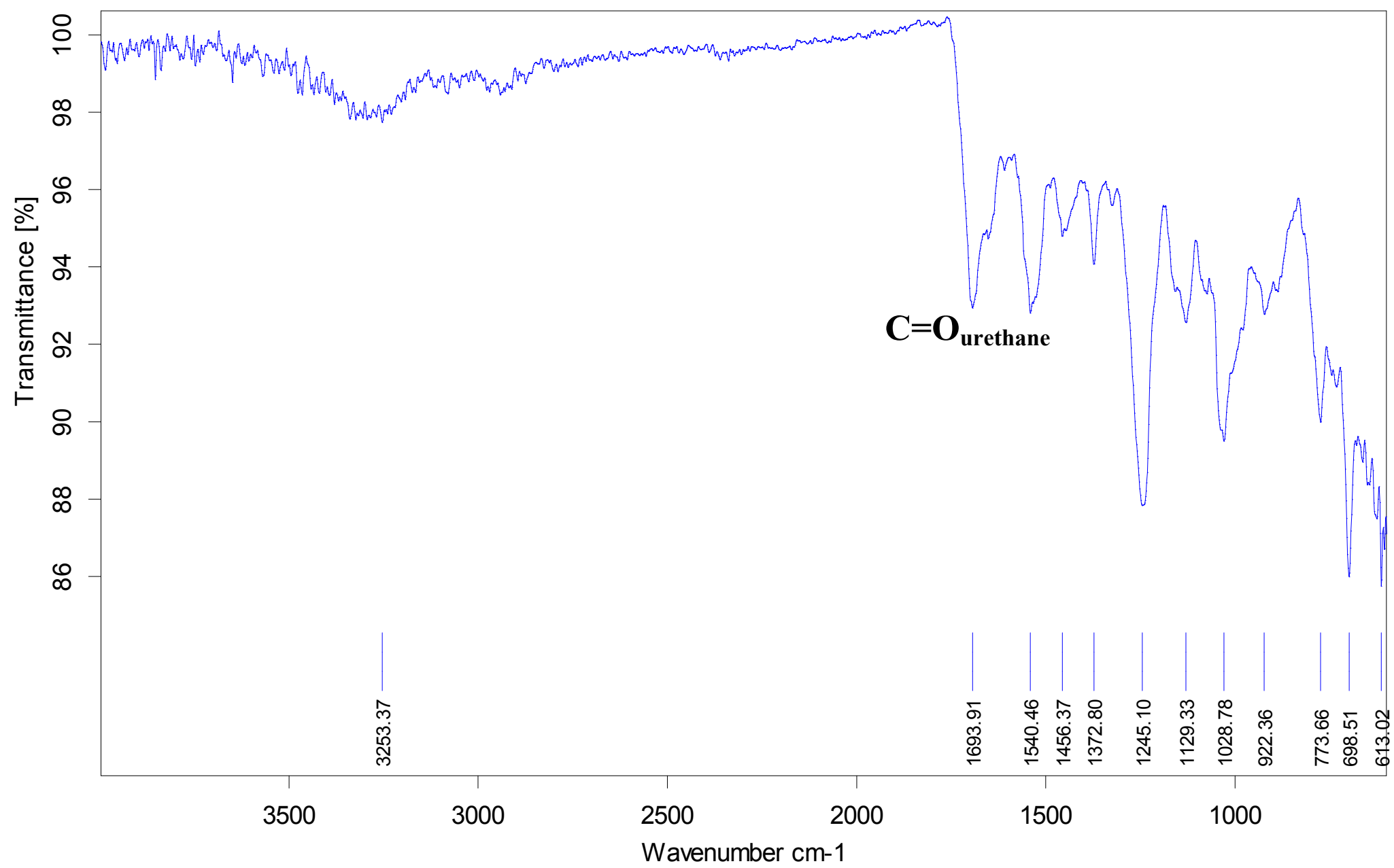

Figure S16. FT-IR spectrum for carbamate **2b**

S25. NMR spectra of hydroxycarbamates 1,2a and 1,2b.

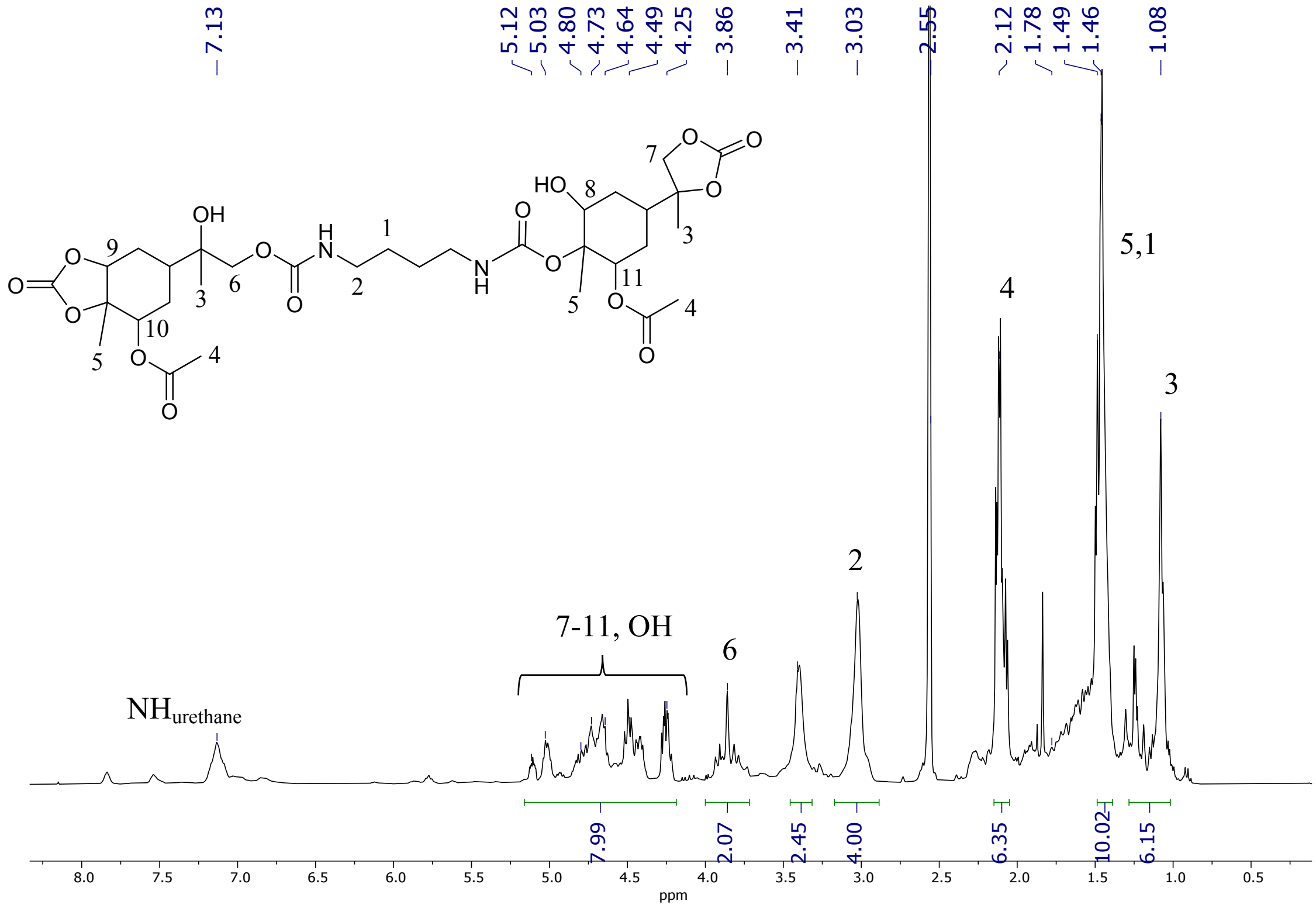

Figure S17. ^{1}H-NMR spectrum for carbamate **1a**.

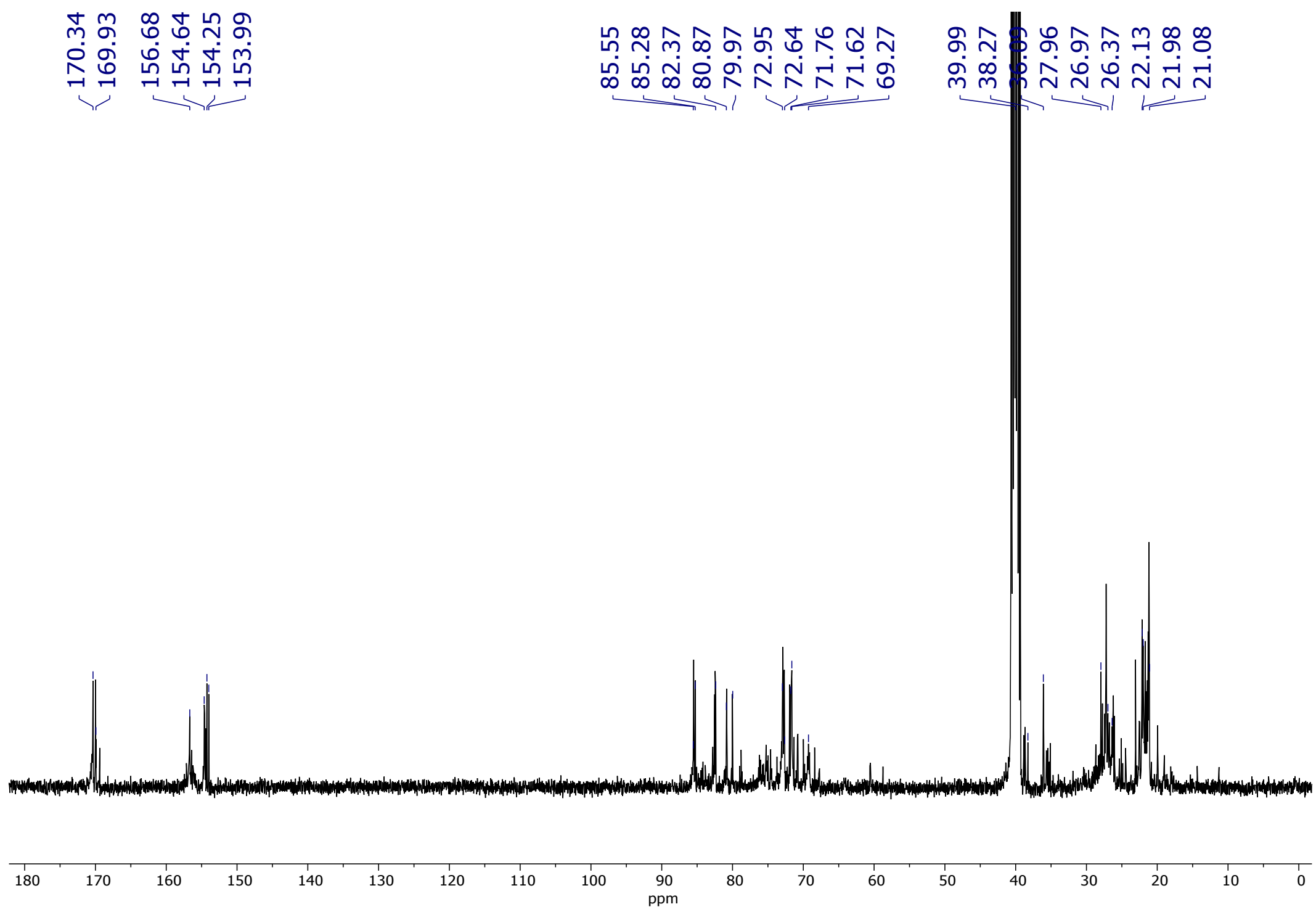

Figure S18. ^{13}C-NMR spectrum for carbamate **1a**

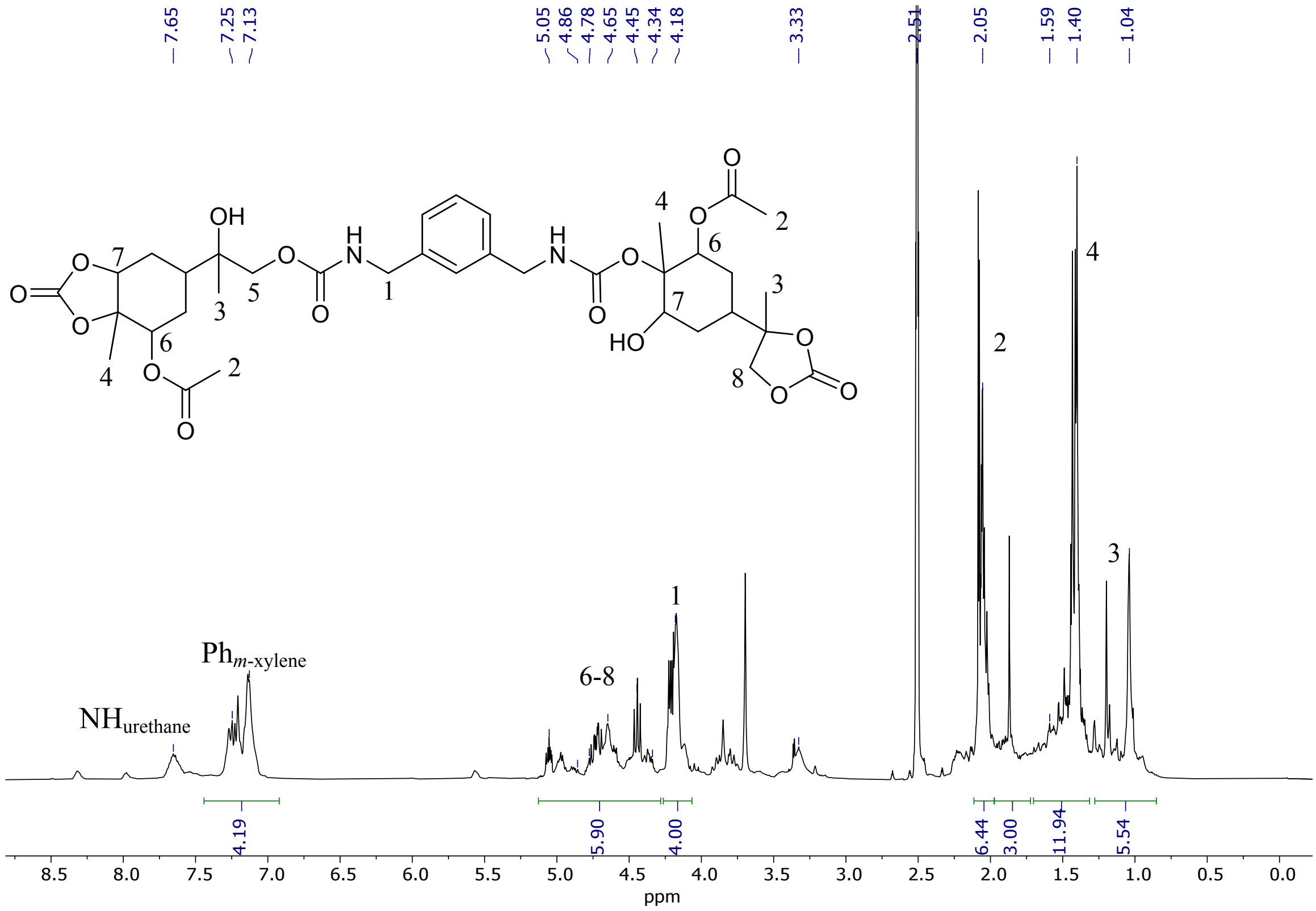

Figure S19. ^{1}H-NMR spectrum for carbamate **1b**

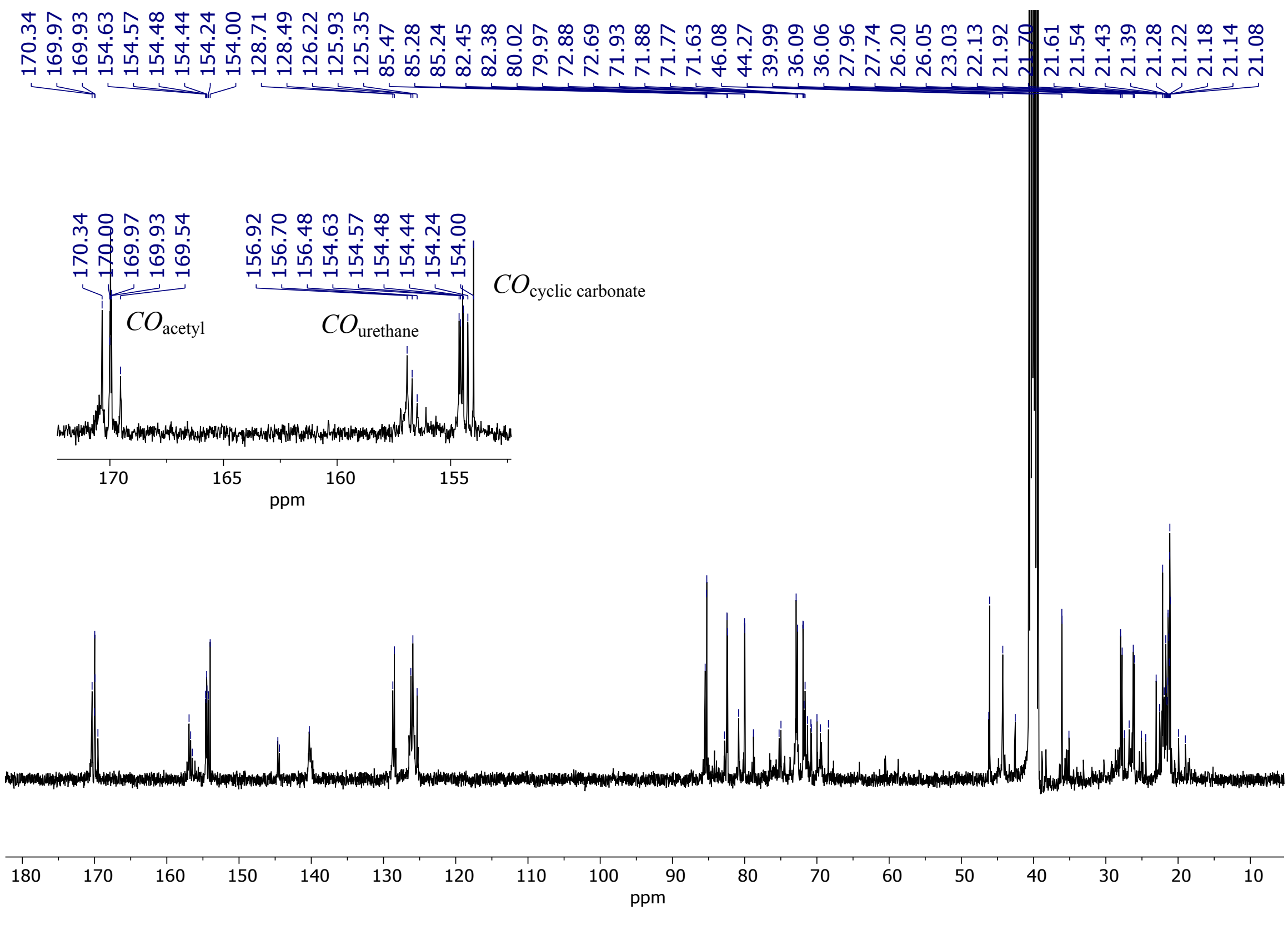

Figure S20. ^{13}C-NMR spectrum for carbamate **1b**

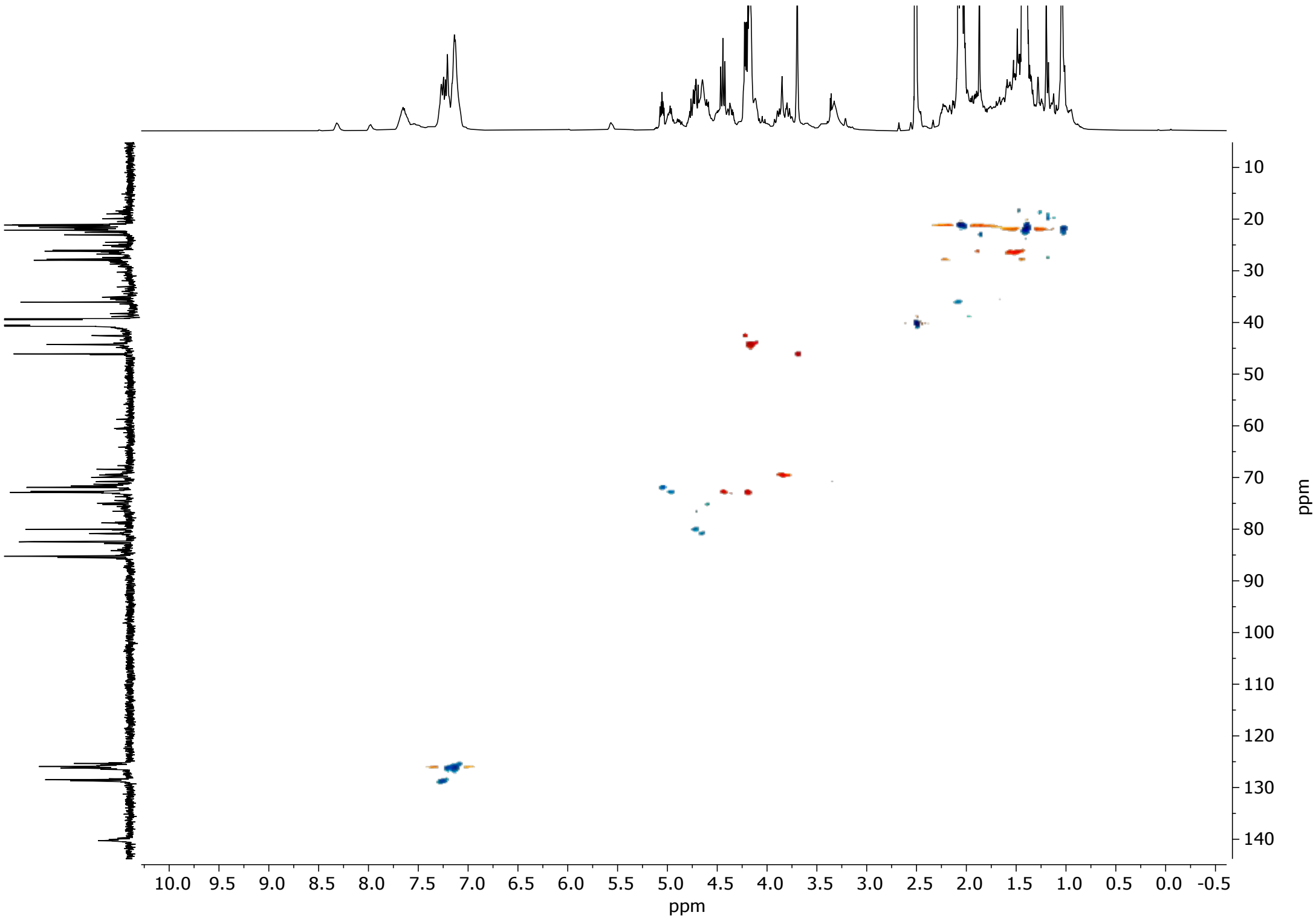

Figure S21. ^{13}C-{^{1}H}-NMR HSQC spectrum for carbamate **1b**

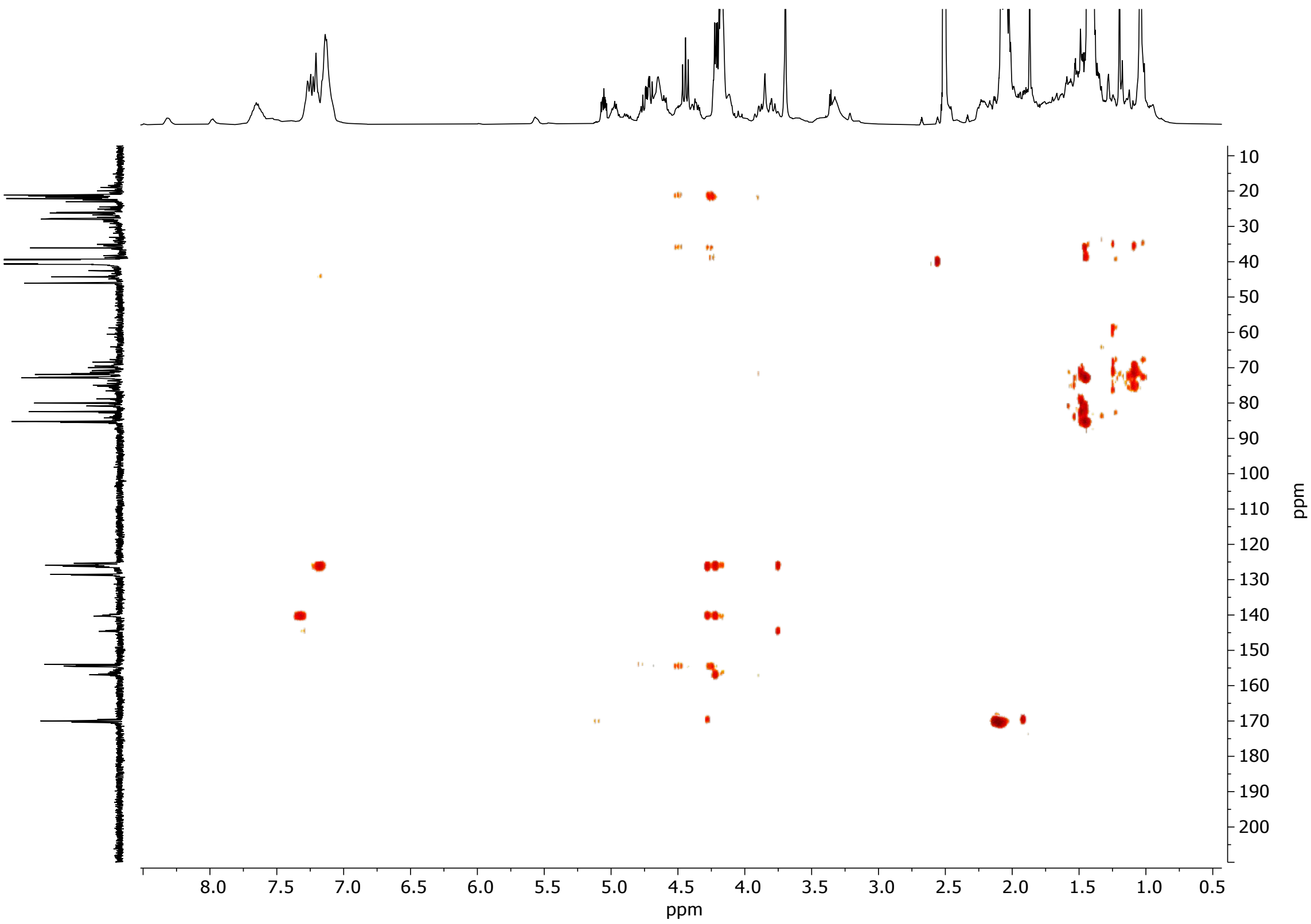

Figure S22. ^{13}C-{^{1}H}-NMR HMBC spectrum for carbamate **1b**

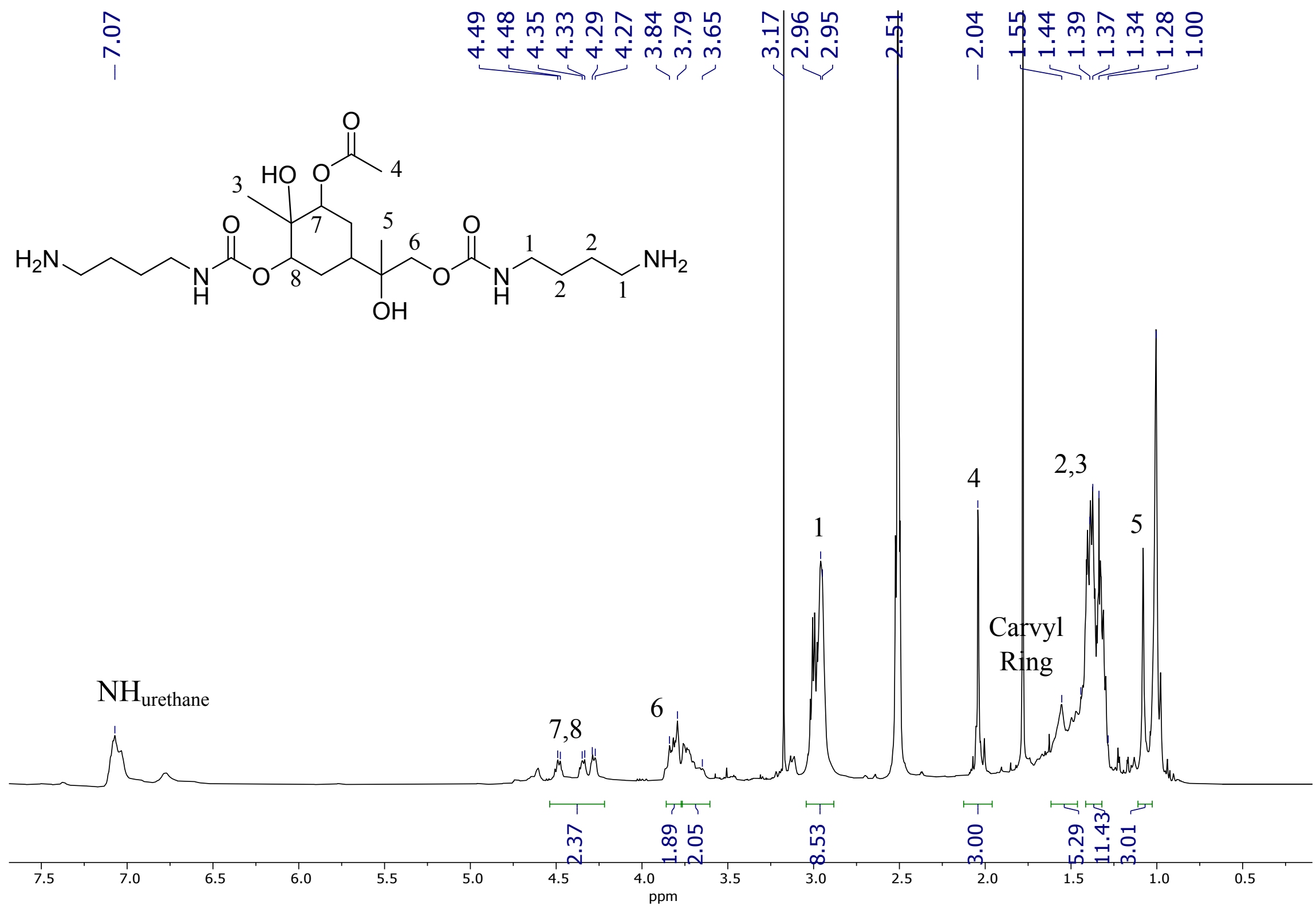

Figure S23. ^{1}H-NMR spectrum for carbamate **2a**

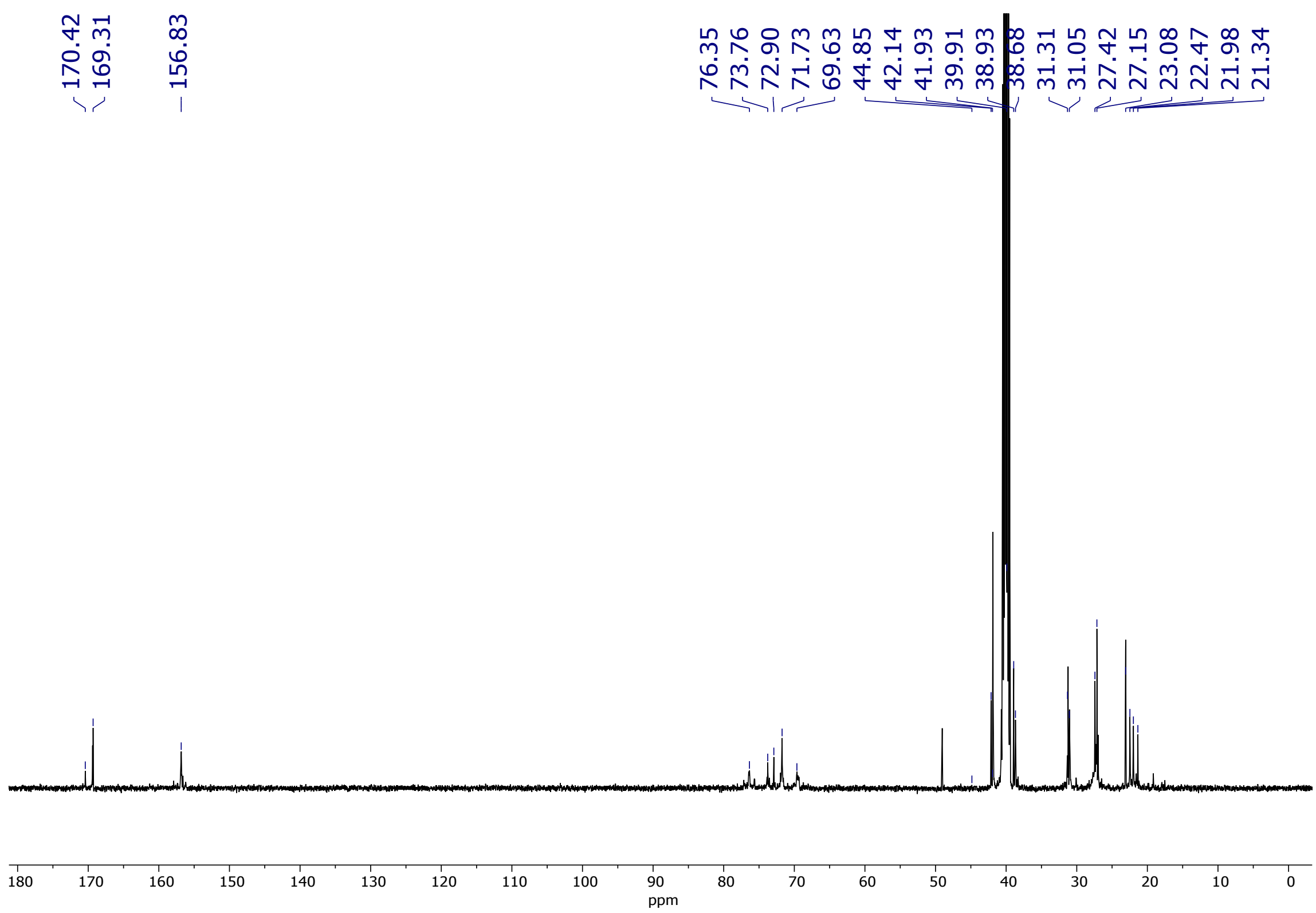

Figure S24. ^{13}C-NMR spectrum for carbamate **2a**

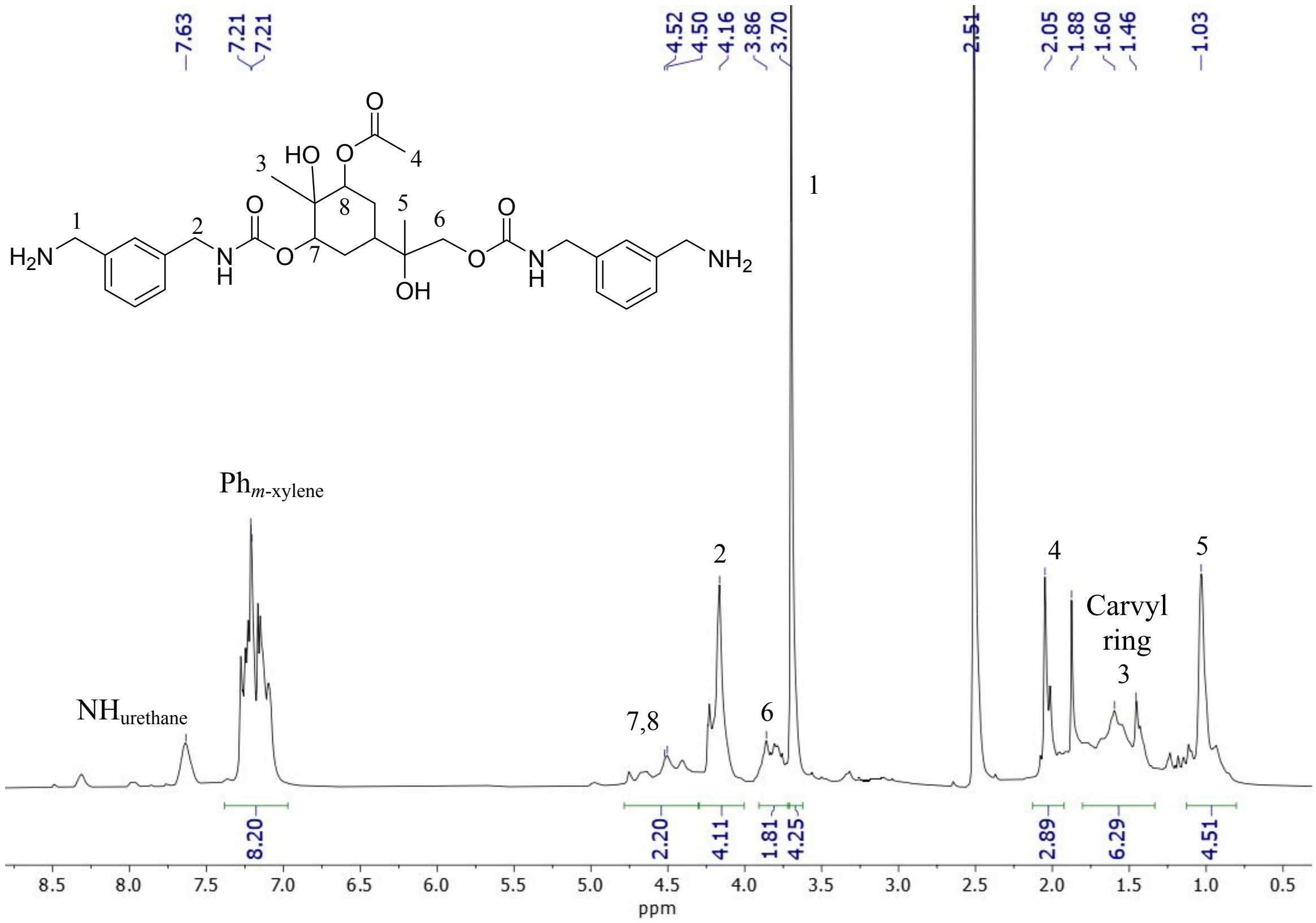

Figure S25. ^{1}H-NMR spectrum for carbamate **2b**

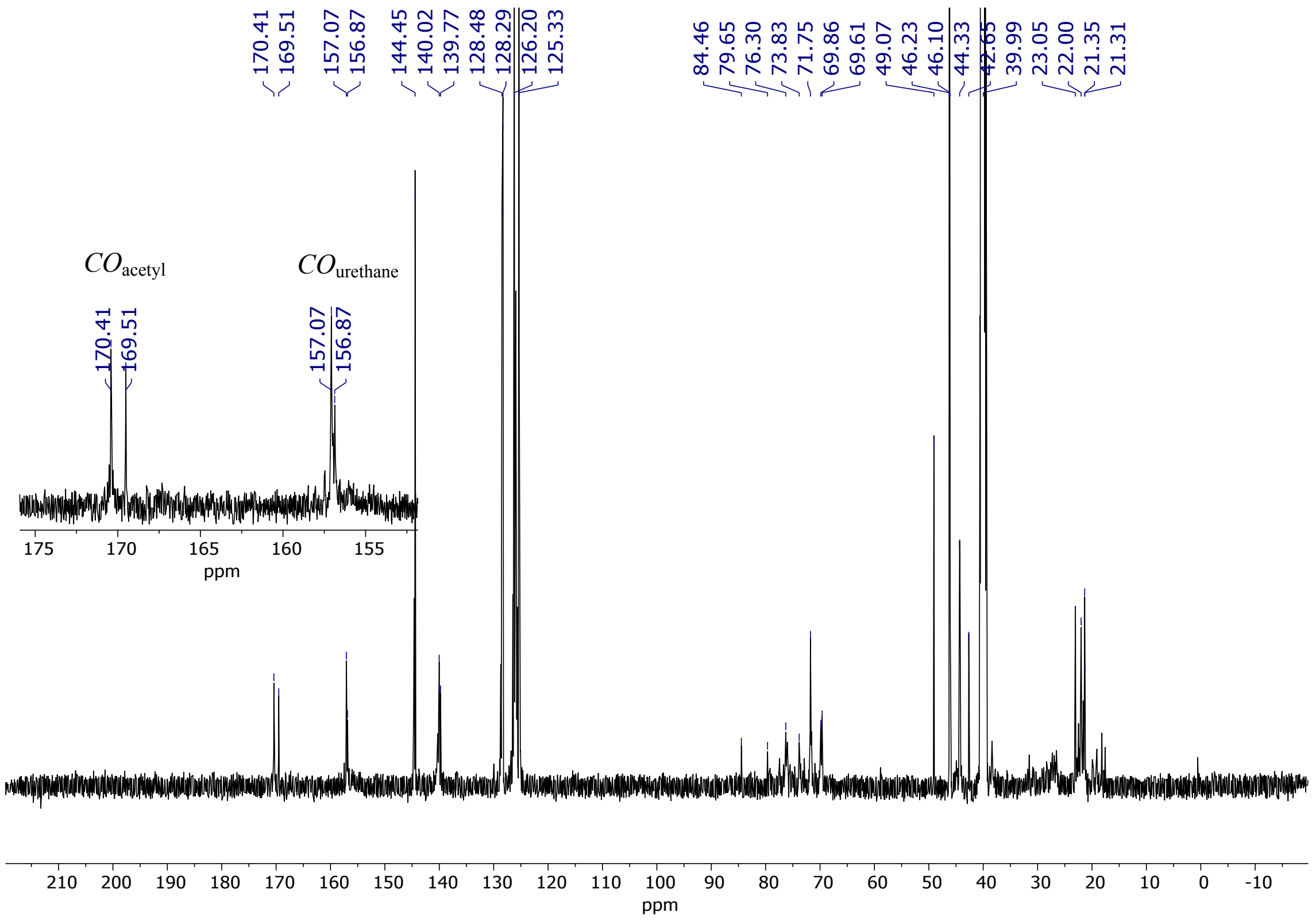

Figure S26. ^{13}C-NMR spectrum for carbamate **2b**

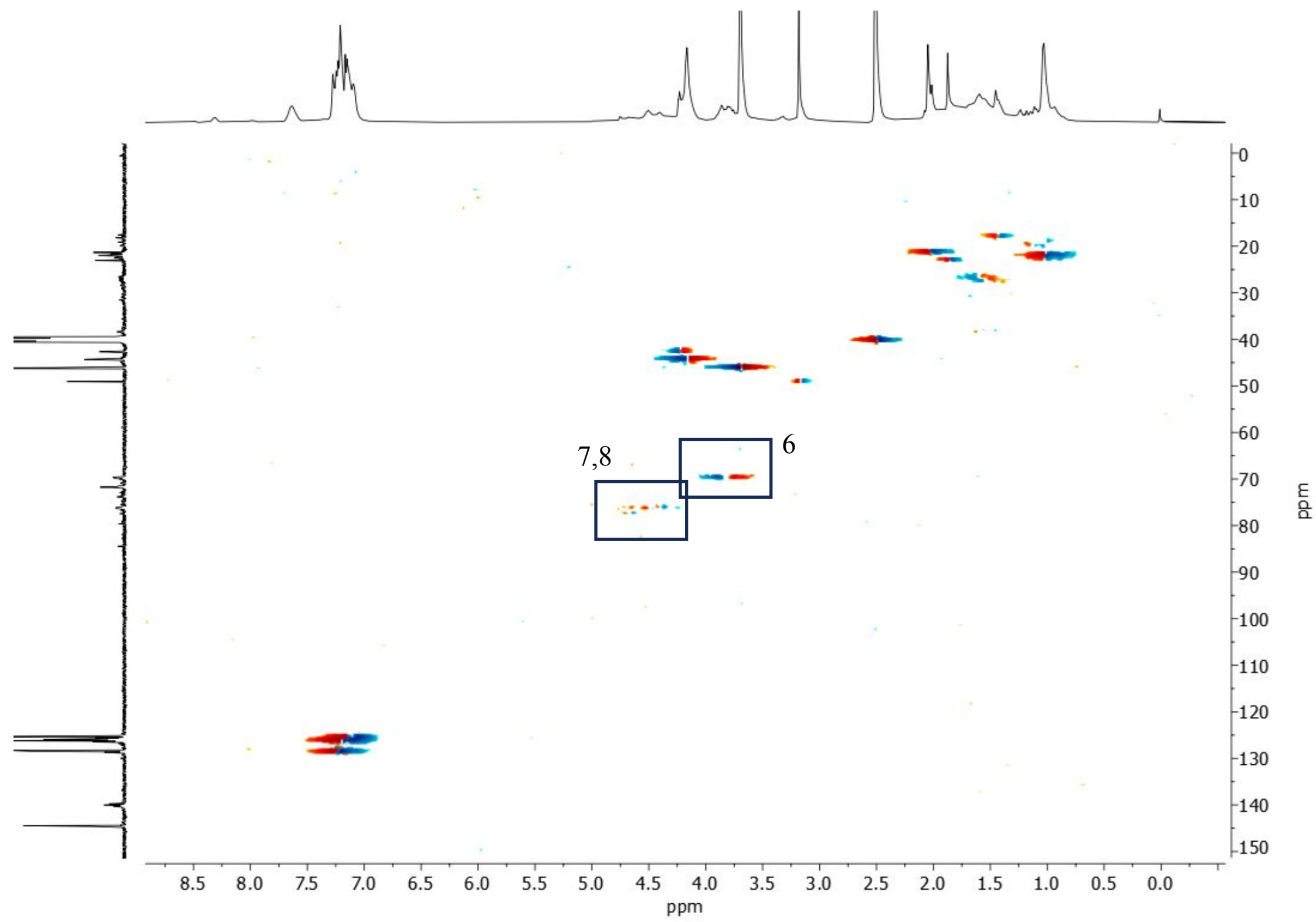

Figure S27. ^{13}C-{^{1}H}-NMR HSQC spectrum for carbamate **2b**

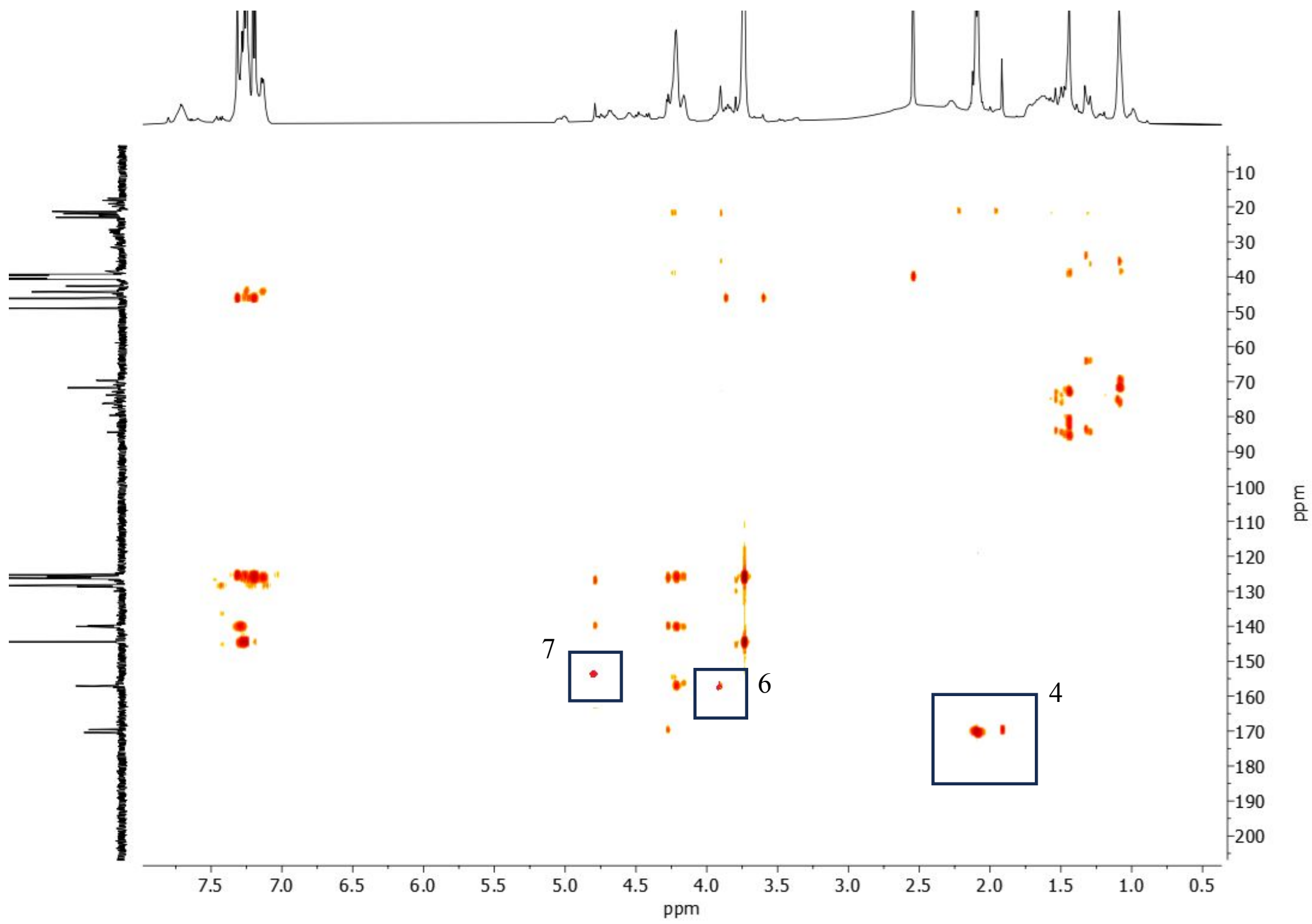

Figure S28. ^{13}C-{^{1}H}-NMR HMBC spectrum for carbamate **2b**

S31. FT-IR spectra of NIPUs 8–12.

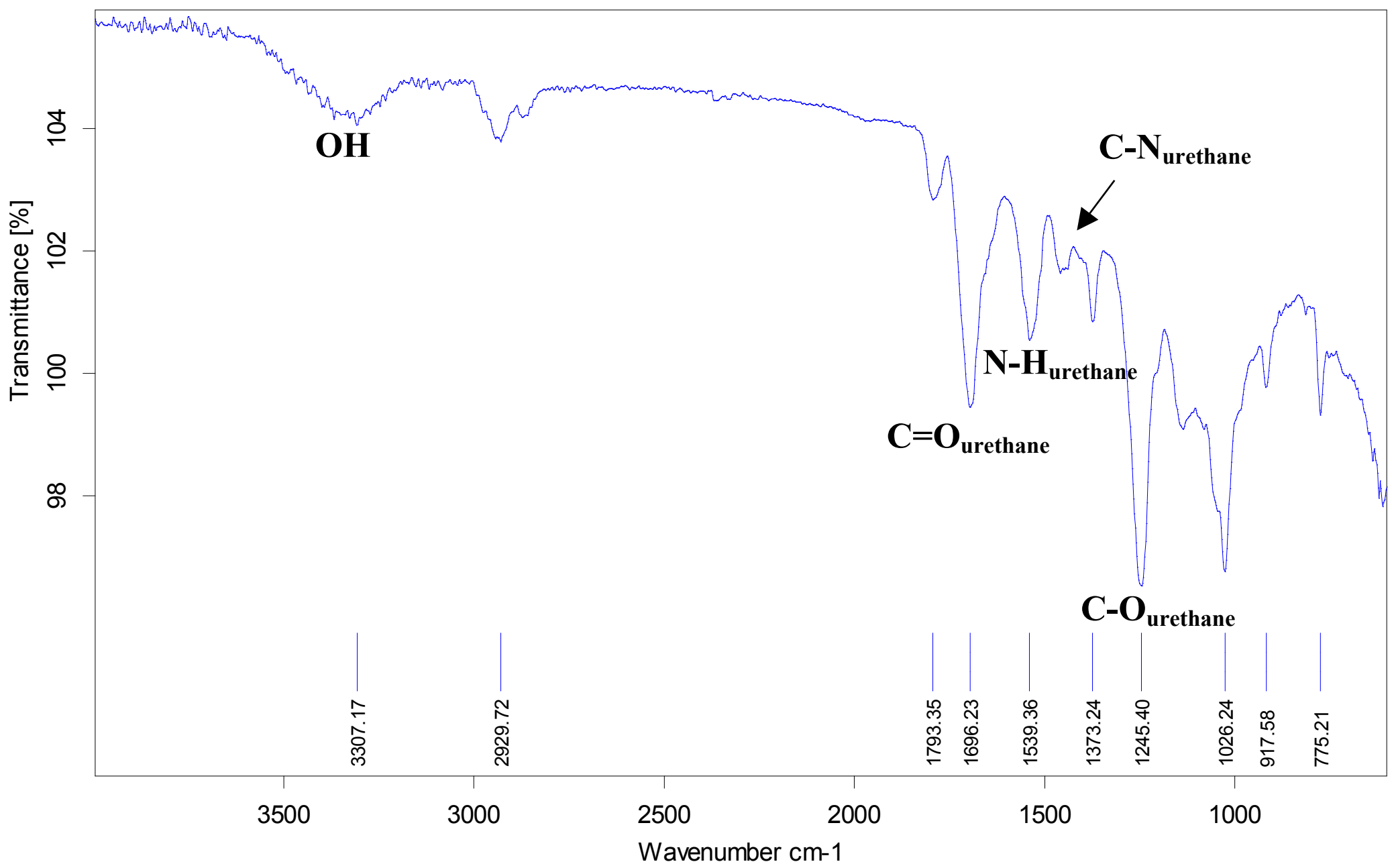

Figure S29. FT-IR spectrum for NIPU **8**

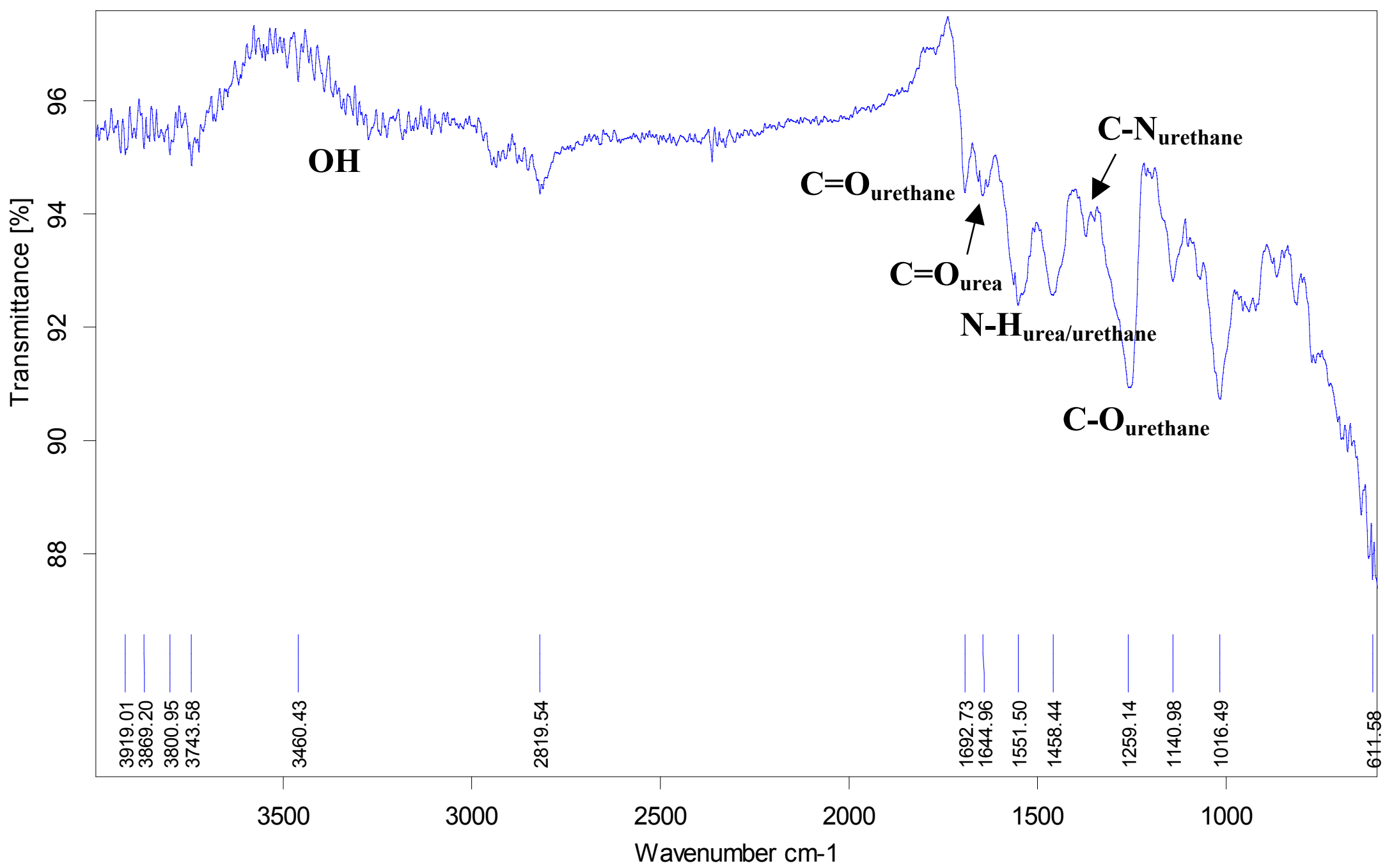

Figure S30. FT-IR spectrum for NIPU **9**

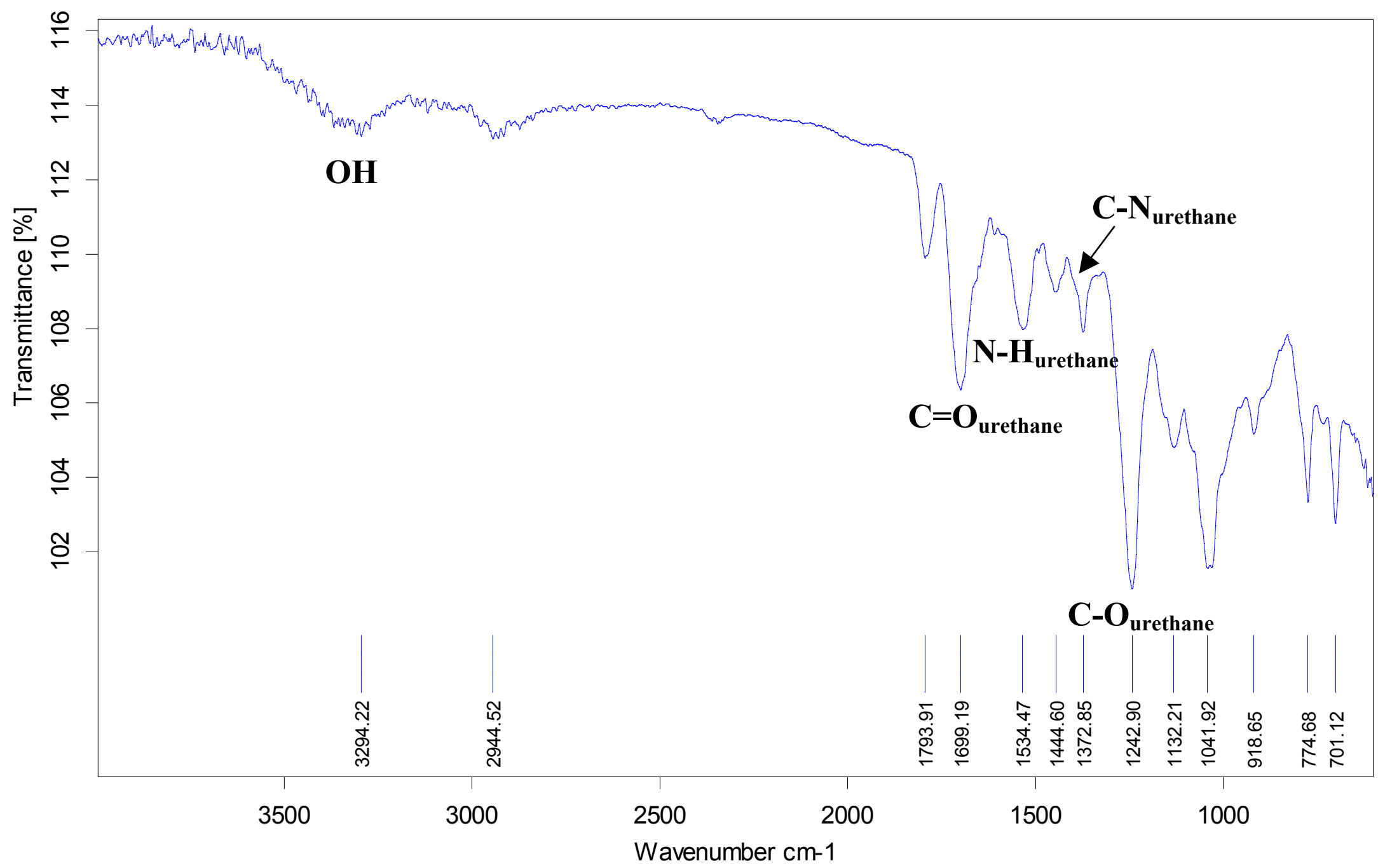

Figure S31. FT-IR spectrum for NIPU **10**

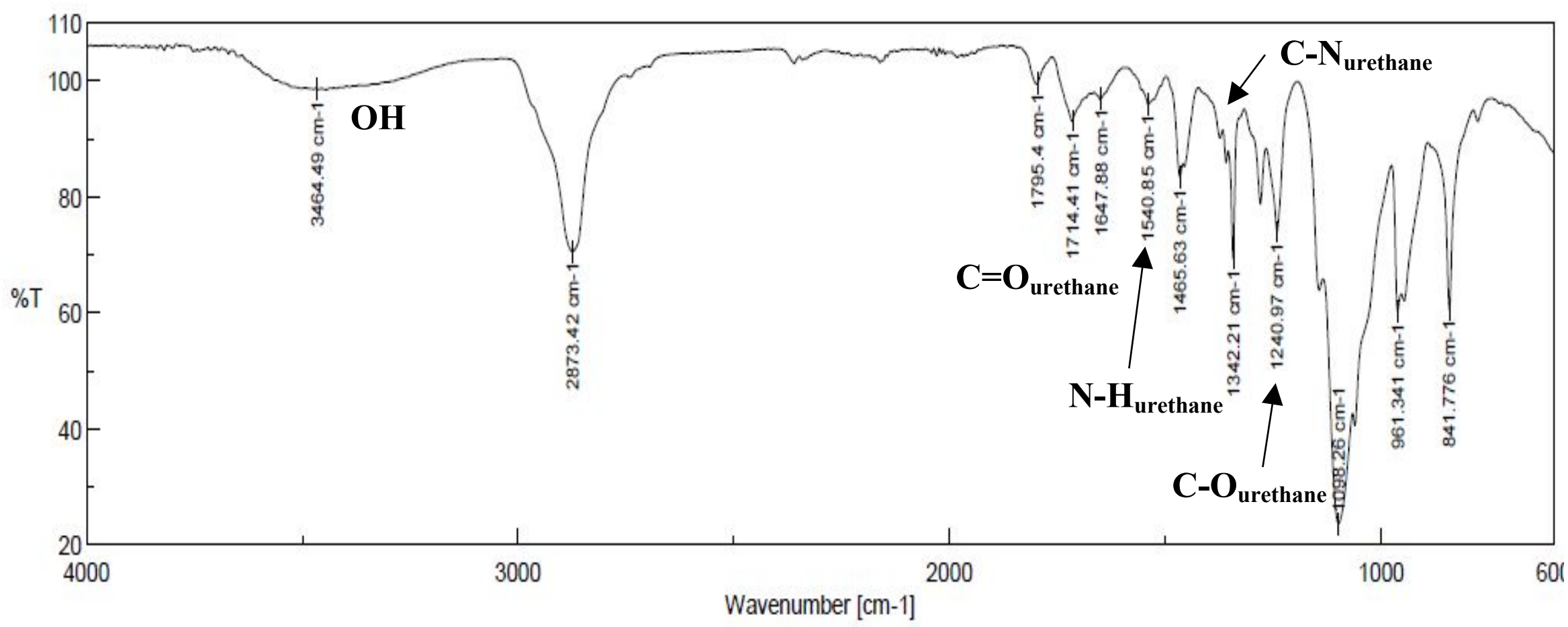

Figure S32. FT-IR spectrum for NIPU **11**

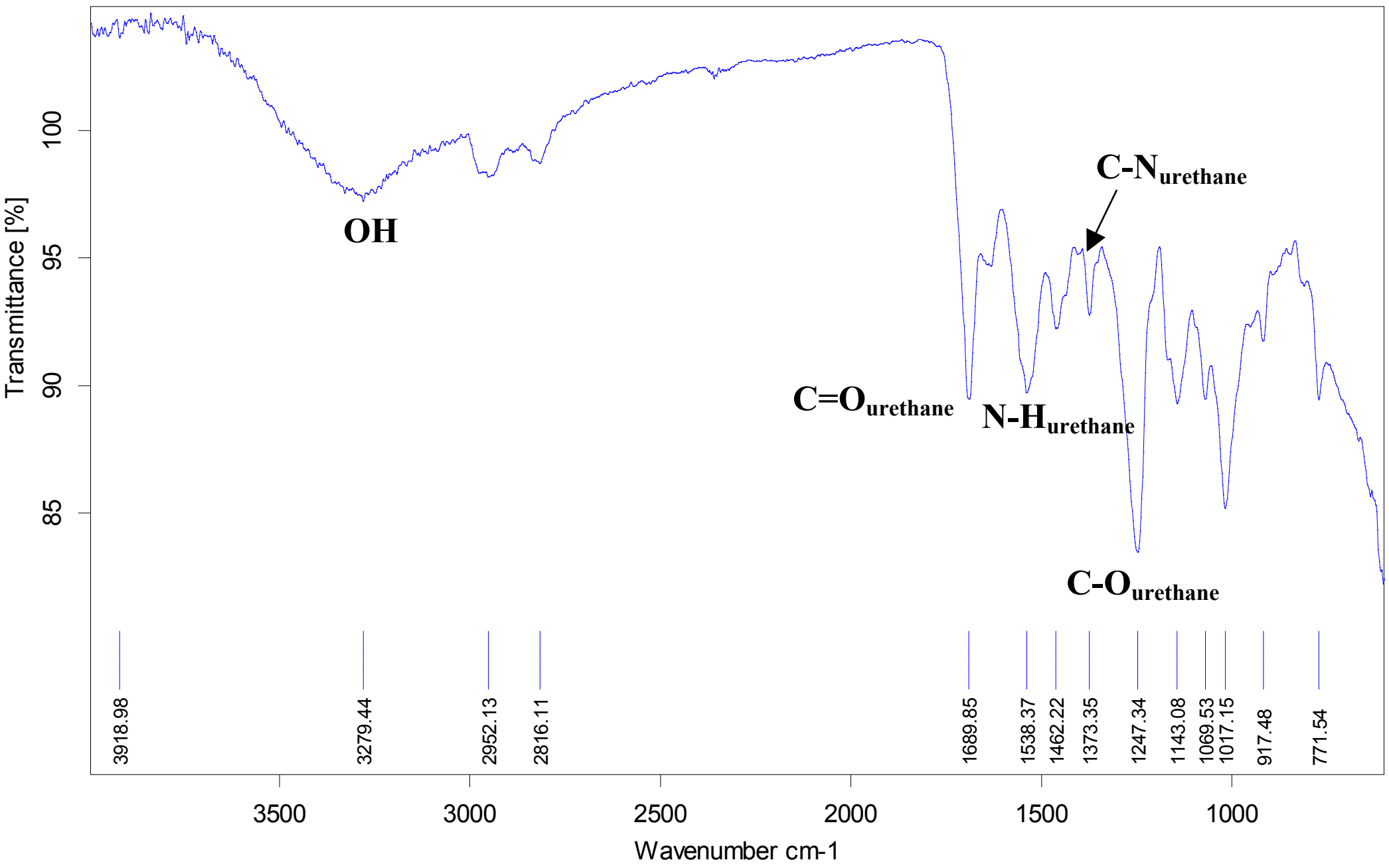

Figure S33. FT-IR spectrum for NIPU **12**

S34. NMR spectra of NIPUs 8–12.

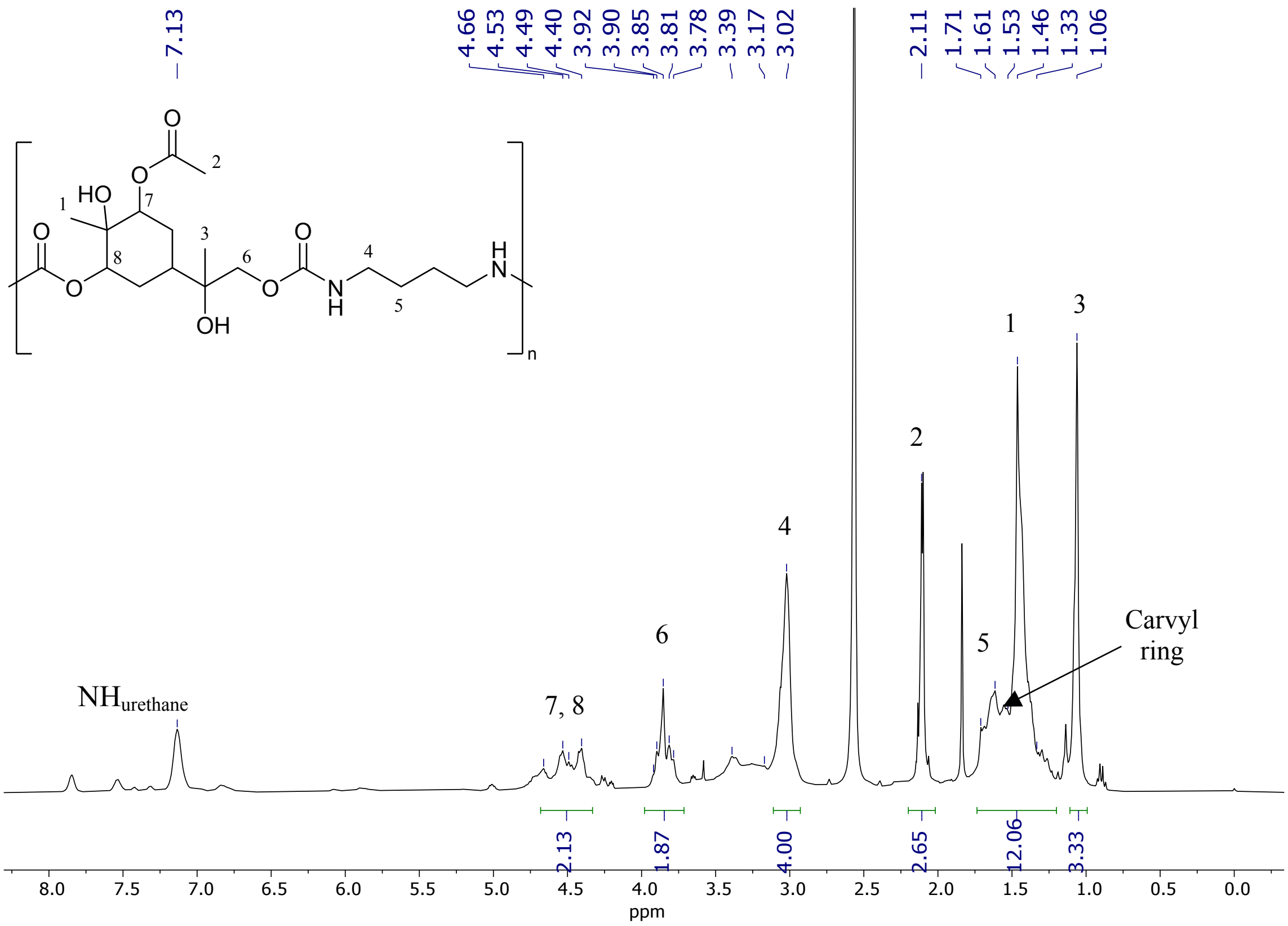

Figure S34. H[1]-NMR spectrum for NIPU **8**

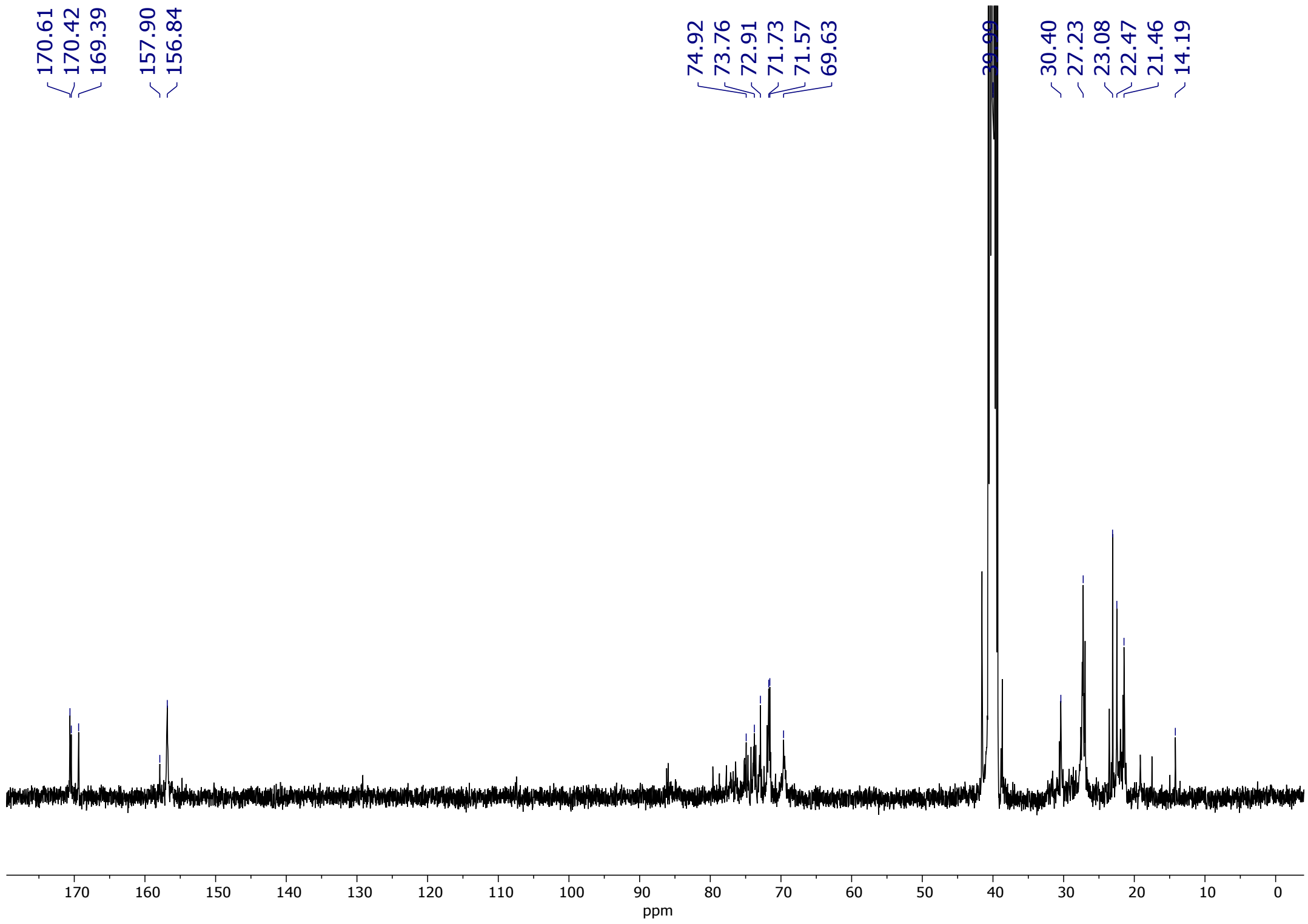

Figure S35. ^{13}C-NMR spectrum for NIPU **8**

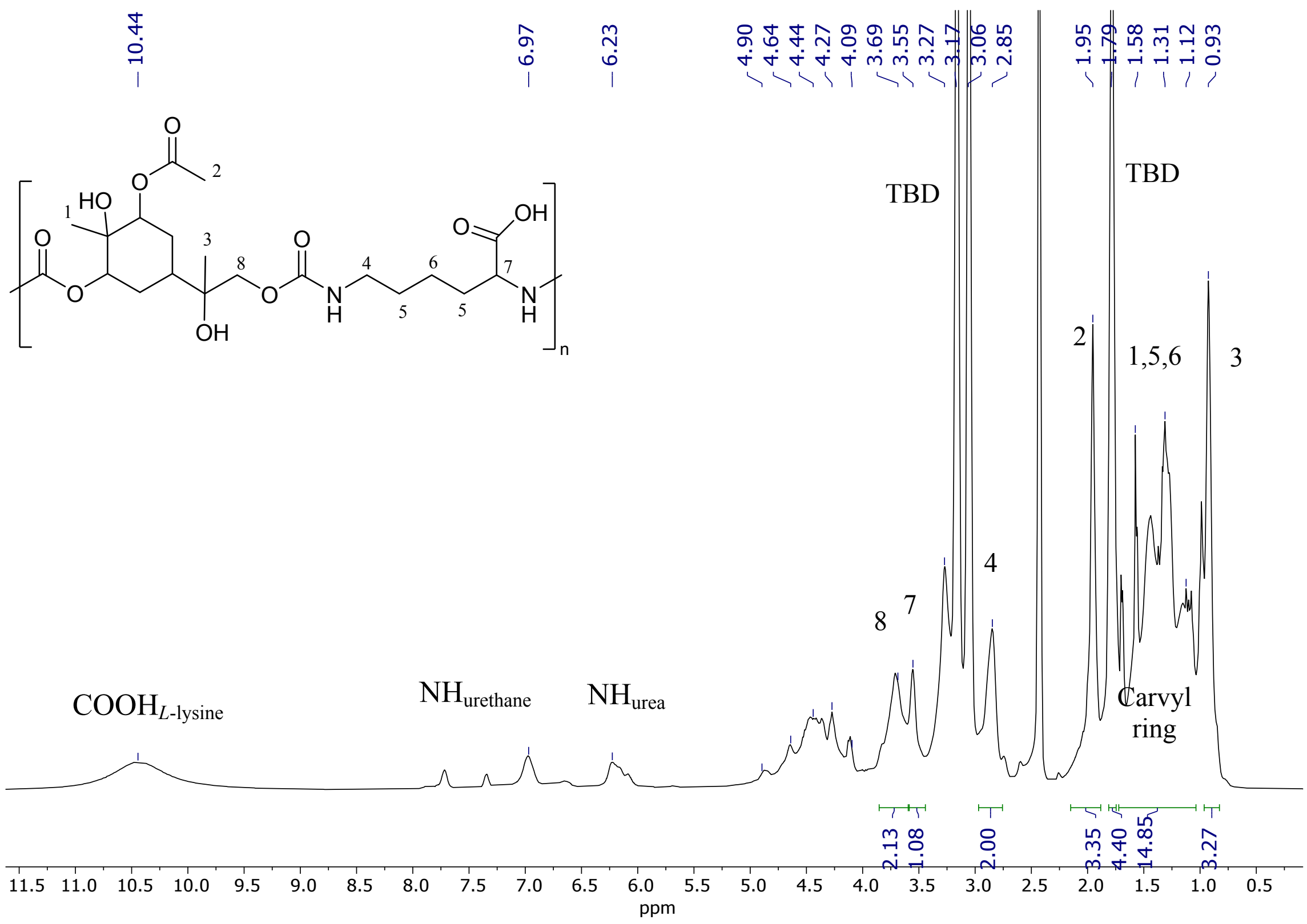

Figure S36. H^1-NMR spectrum for NIPU **9**

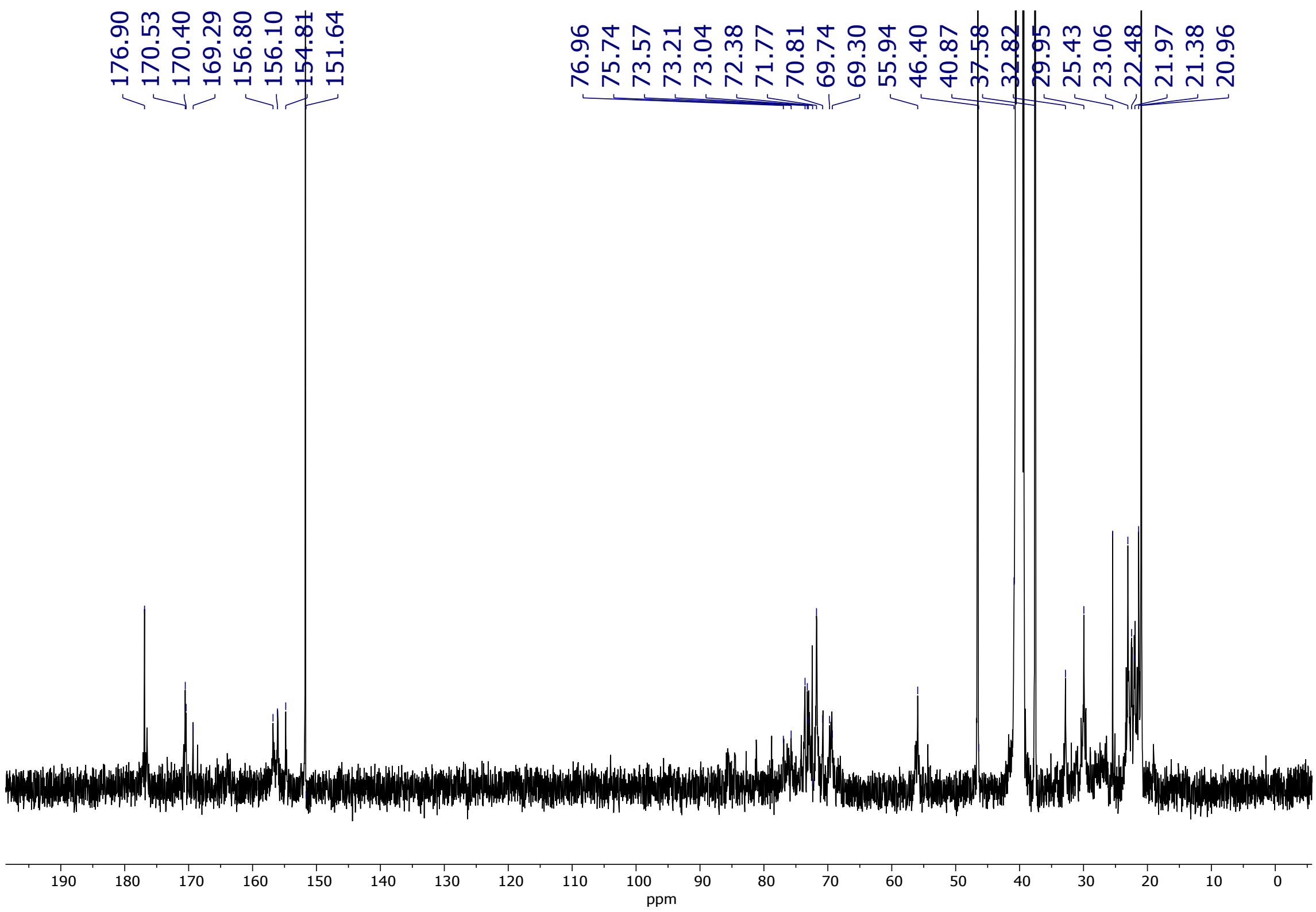

Figure S37. C[13]-NMR spectrum for NIPU **9**

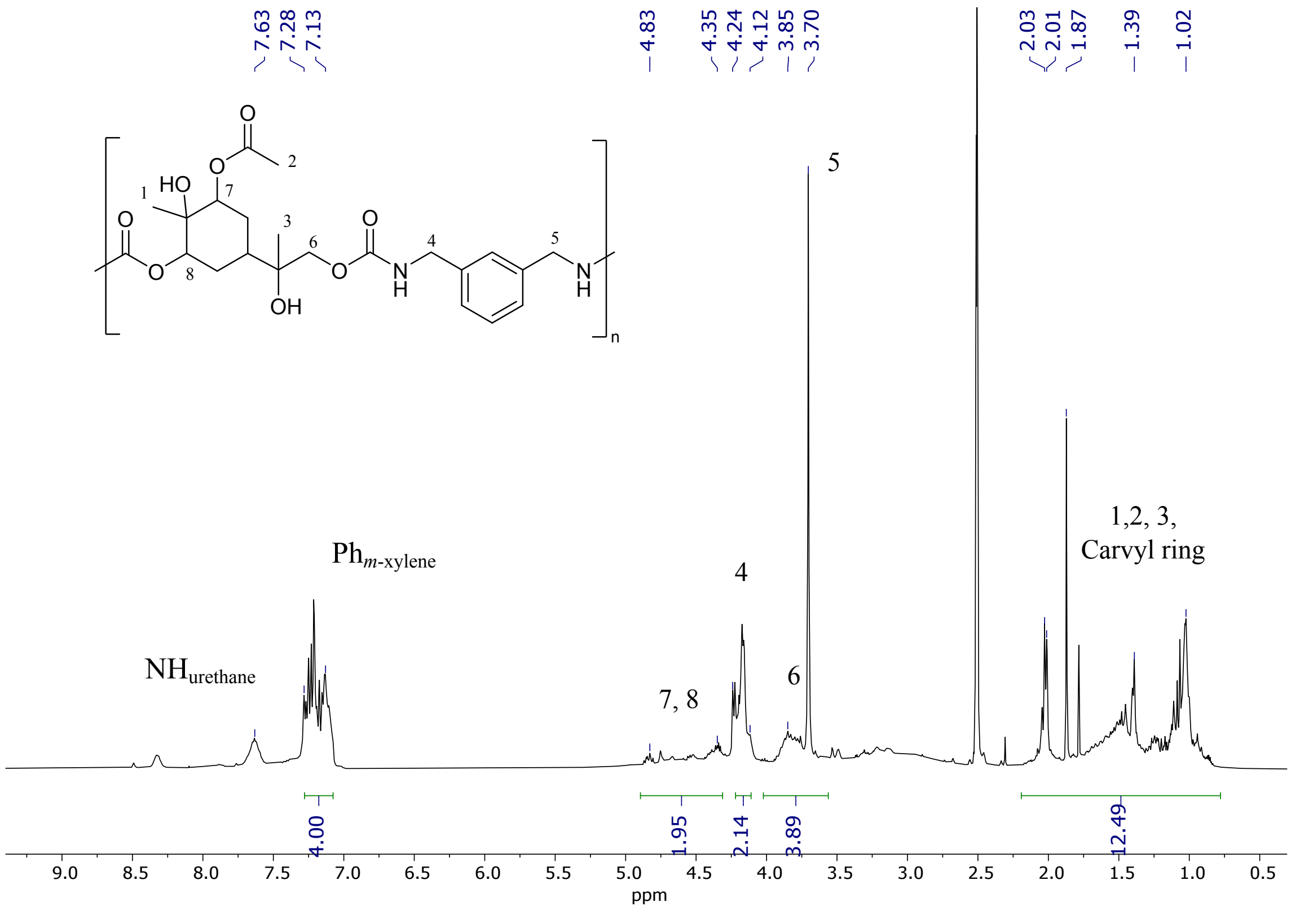

Figure S38. ^{1}H-NMR spectrum for NIPU **10**

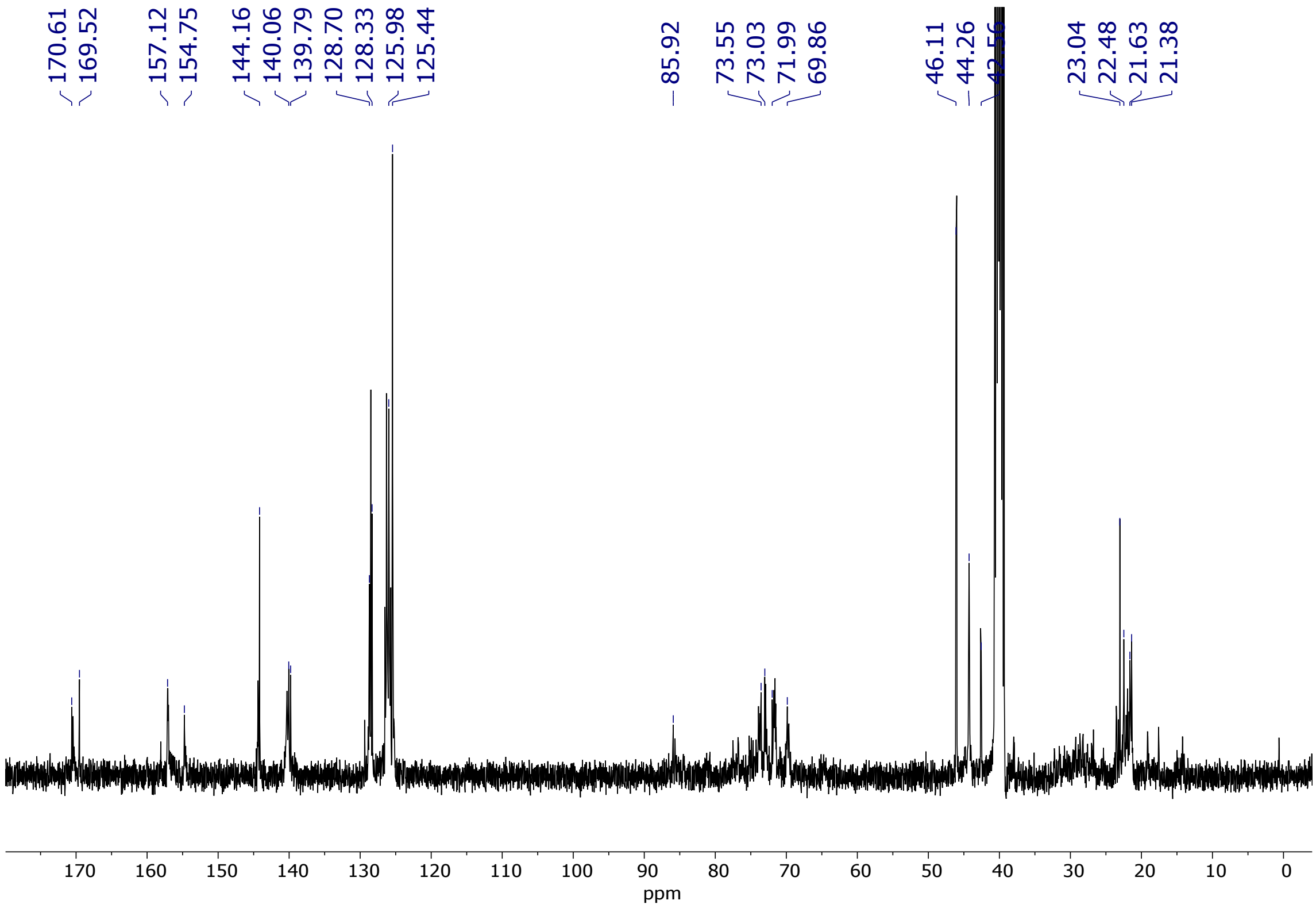

Figure S39. ^{13}C-NMR spectrum for NIPU **10**

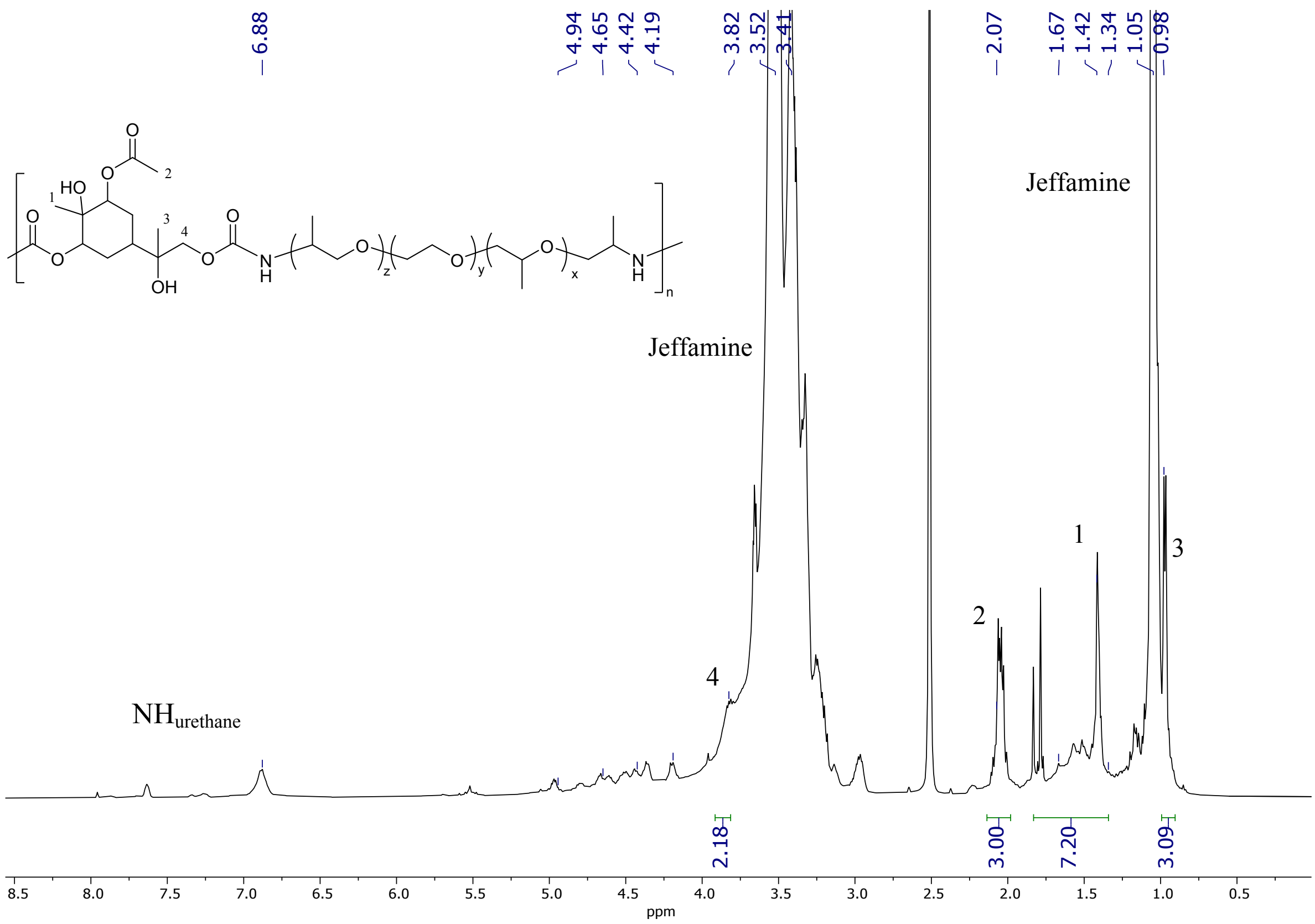

Figure S40. ^{1}H-NMR spectrum for NIPU **11**

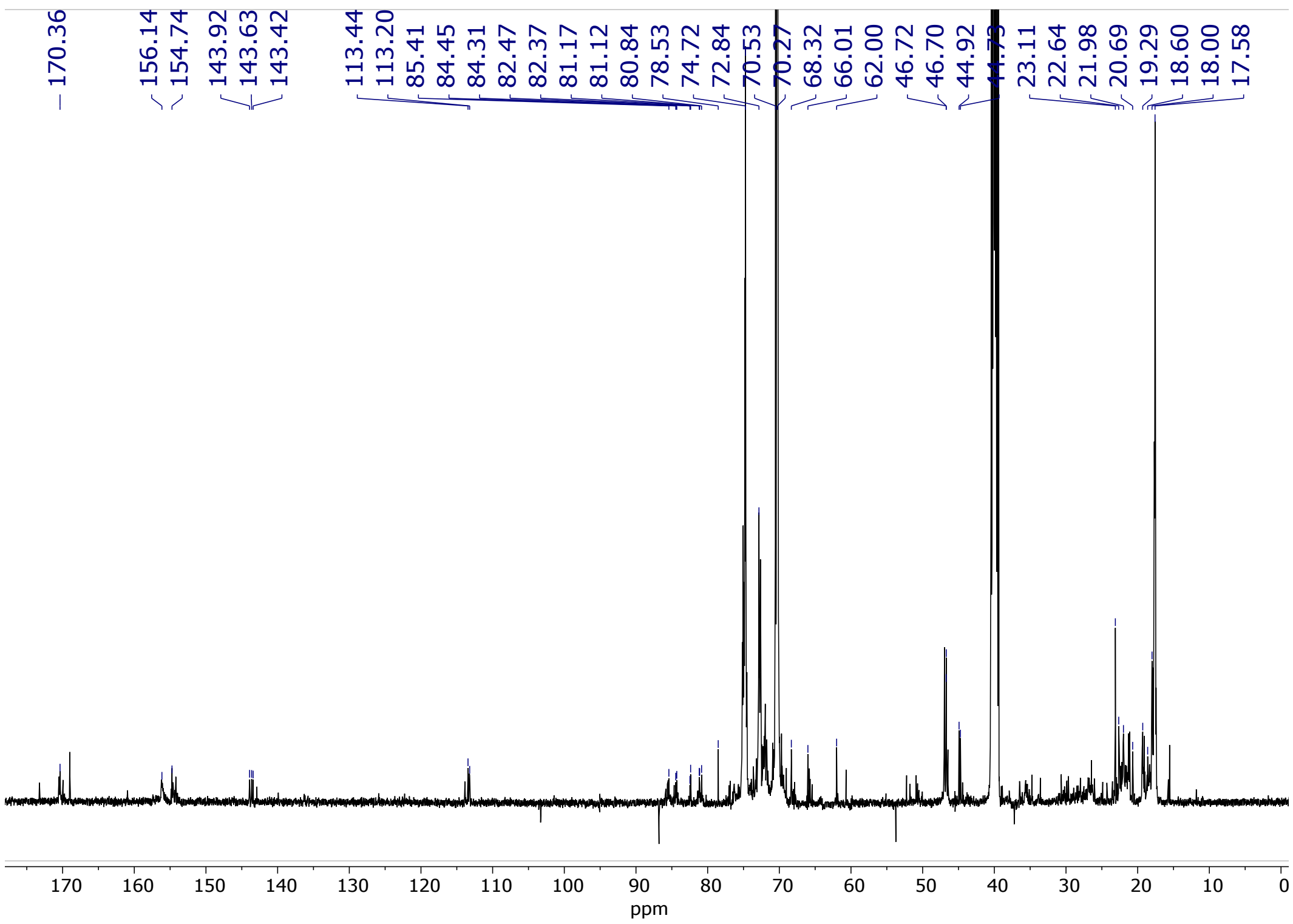

Figure S41. ^{13}C-NMR spectrum for NIPU **11**

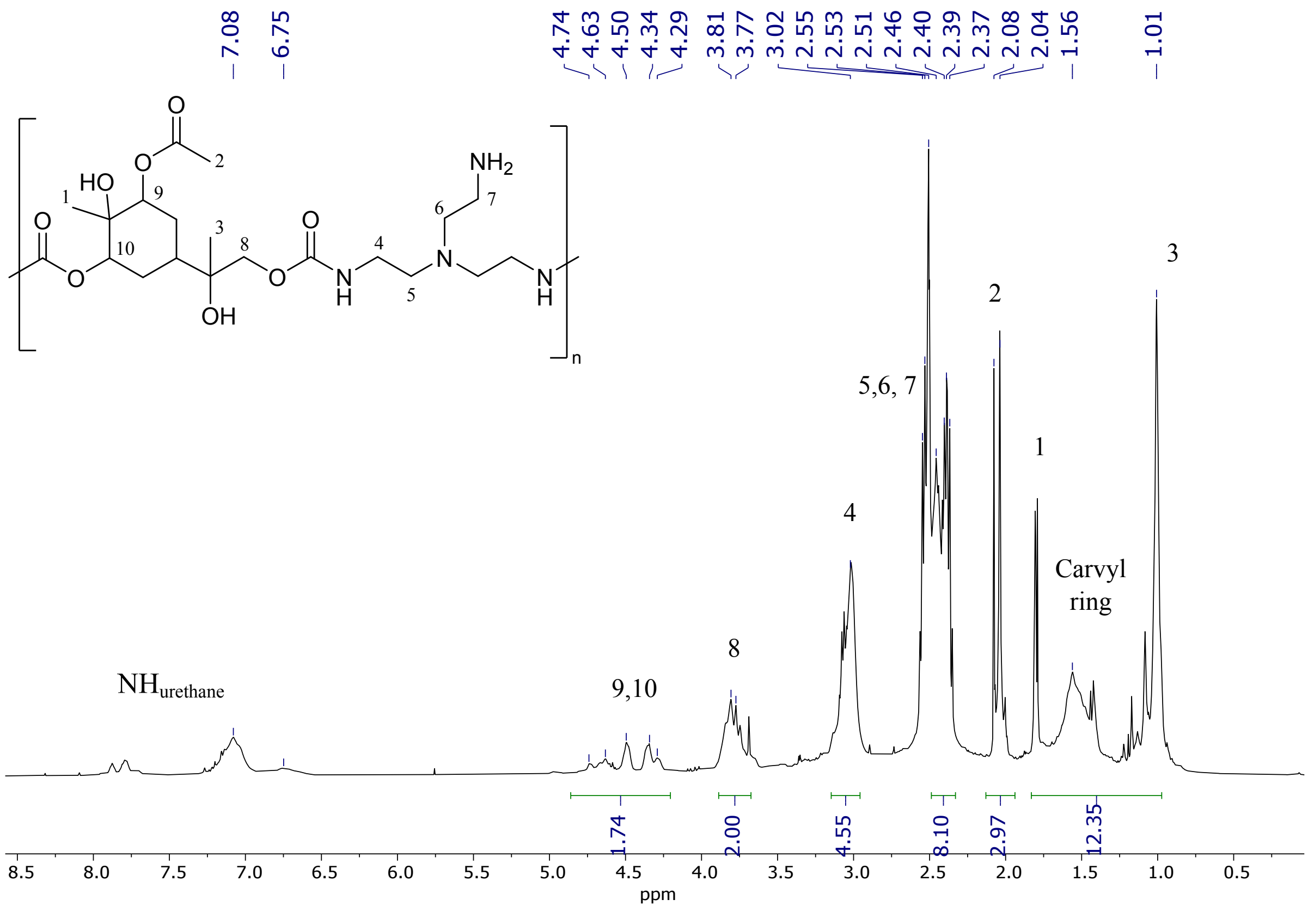

Figure S42. ^{1}H-NMR spectrum for NIPU **12**

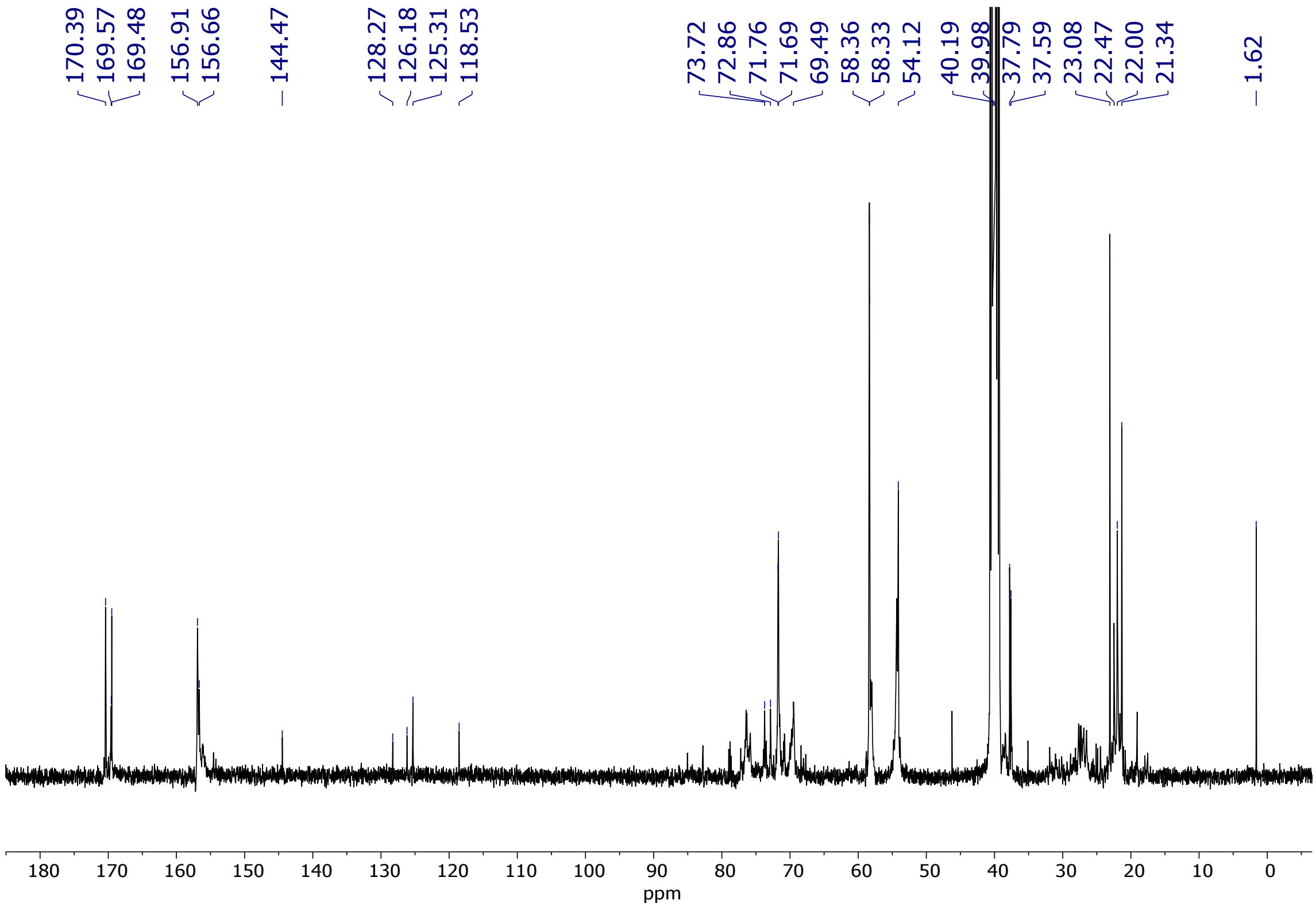

Figure S43. ^{13}C-NMR spectrum for NIPU **12**

S39. TGA graphs and DSC traces of NIPUs 8–12.

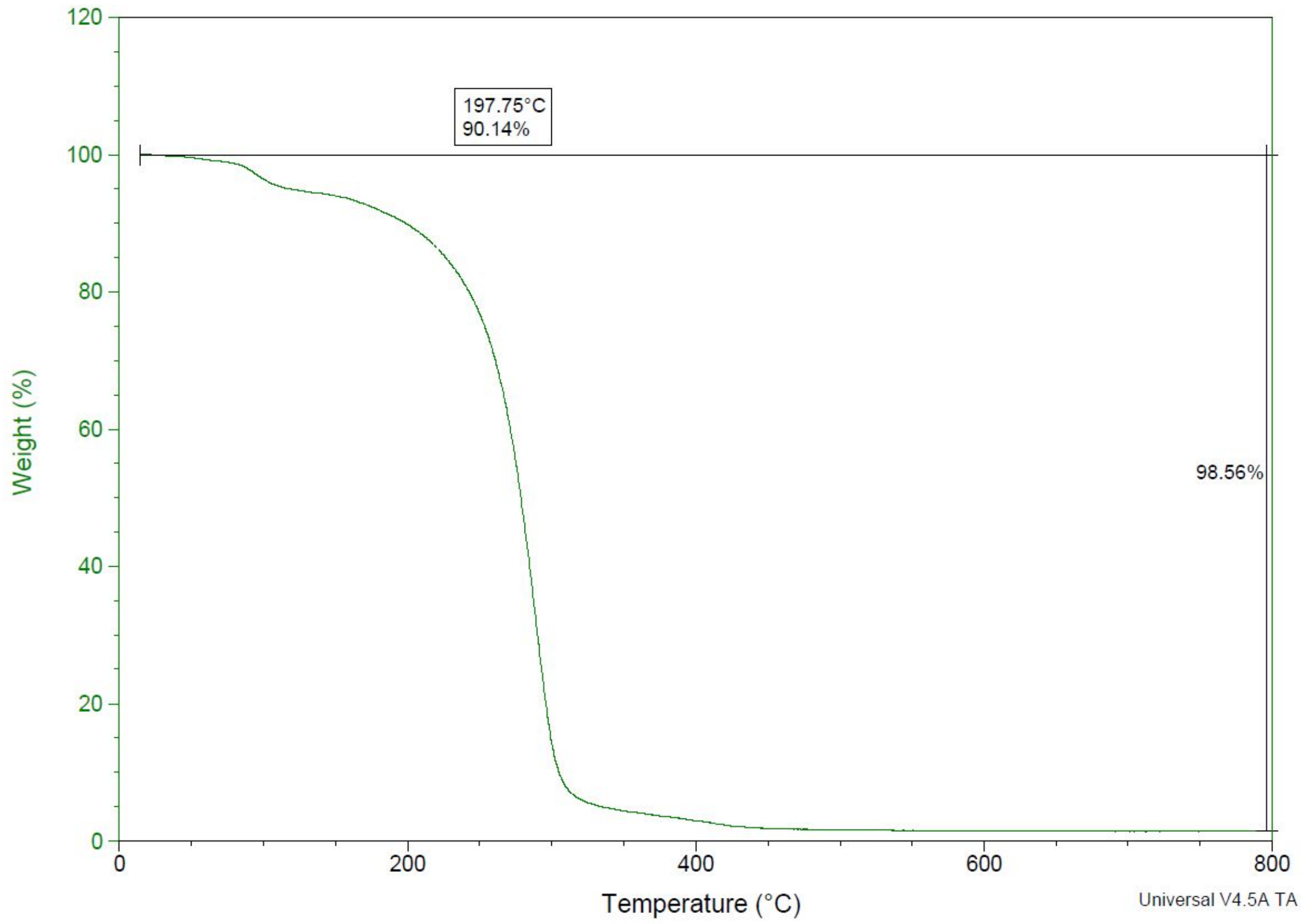

Figure S44. TGA graph for NIPU **8**

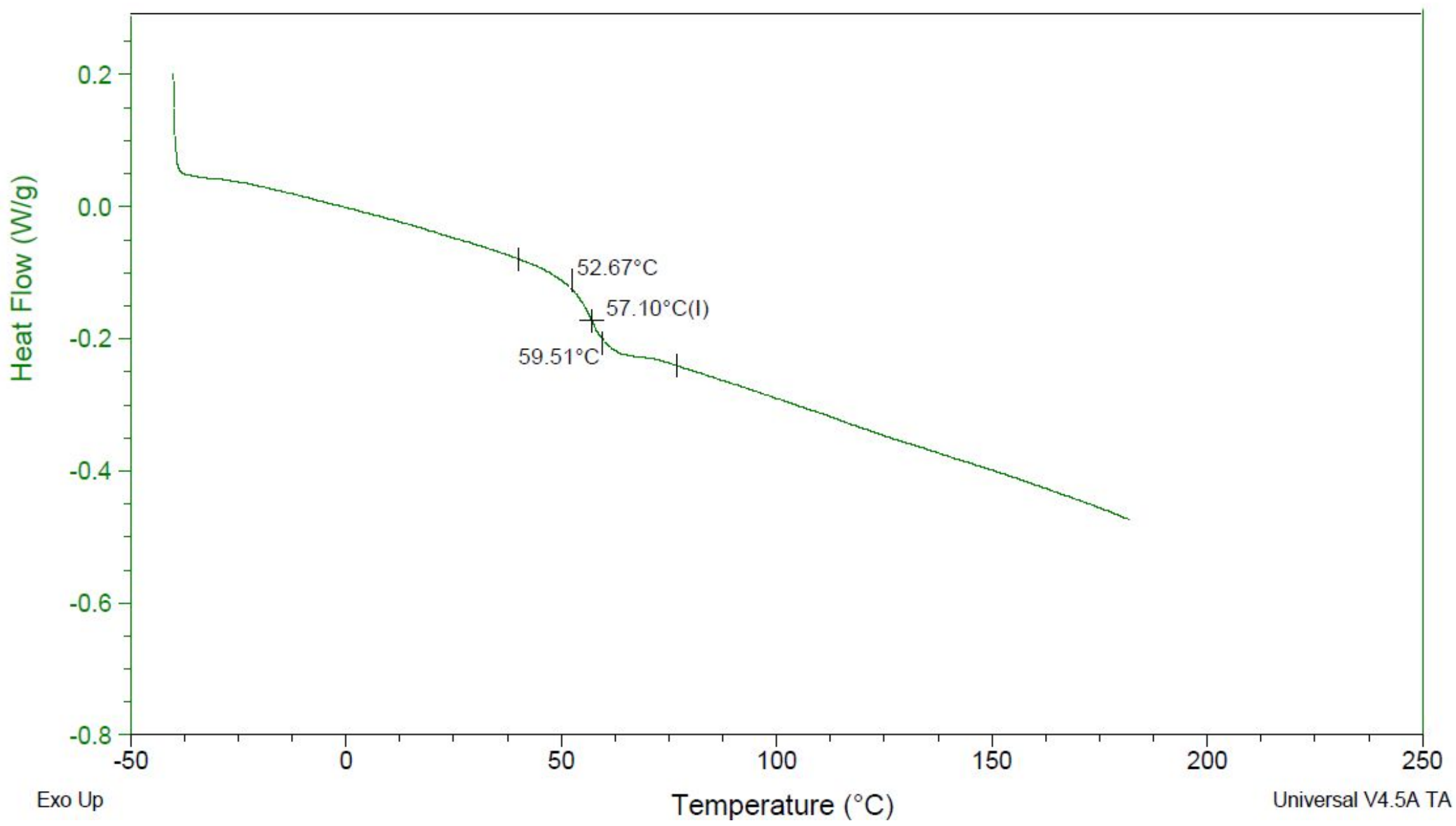

Figure S45. DSC spectrum for NIPU **8**

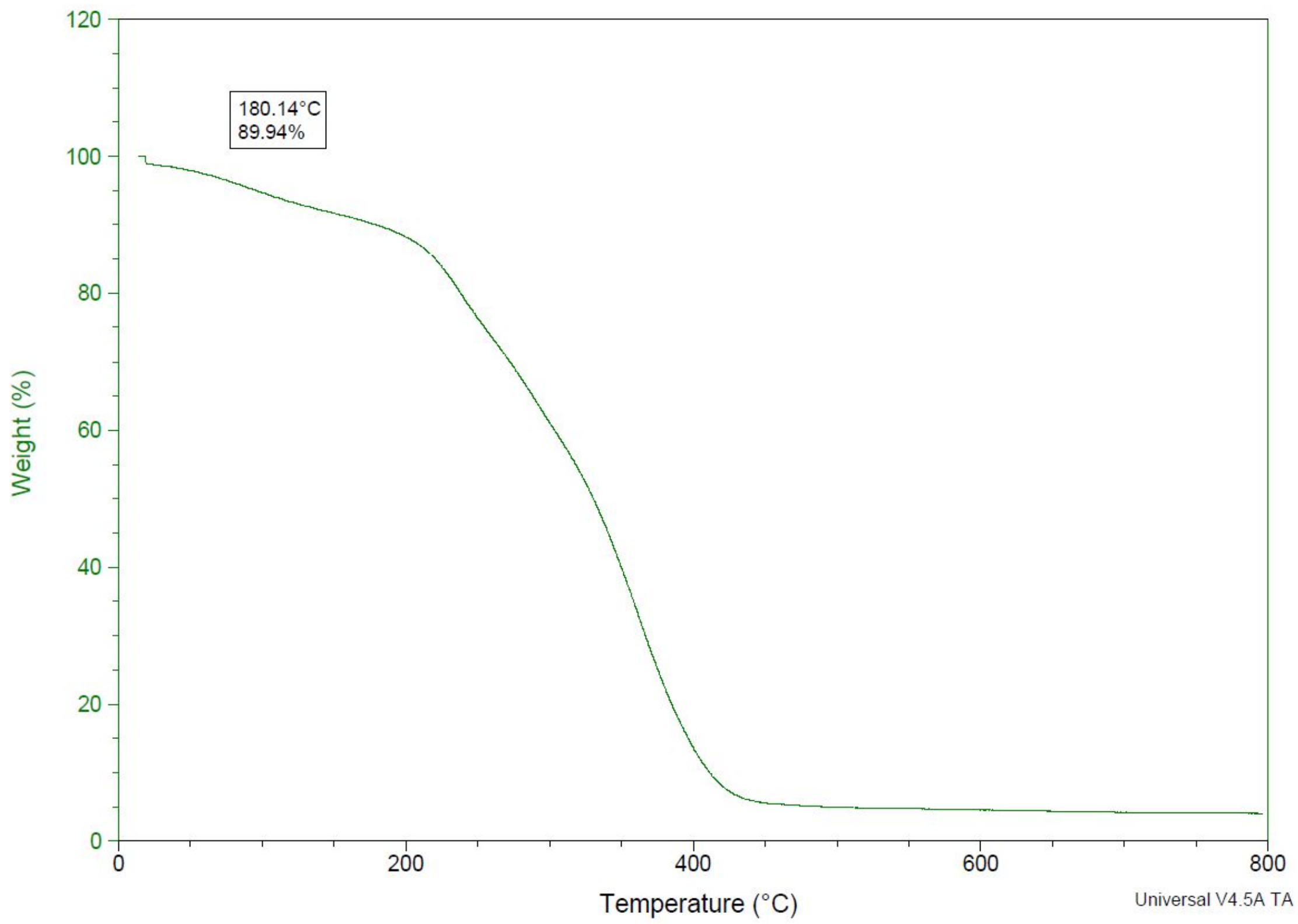

Figure S46. TGA spectrum for NIPU **8** (uncrystallized CADC mixture used)

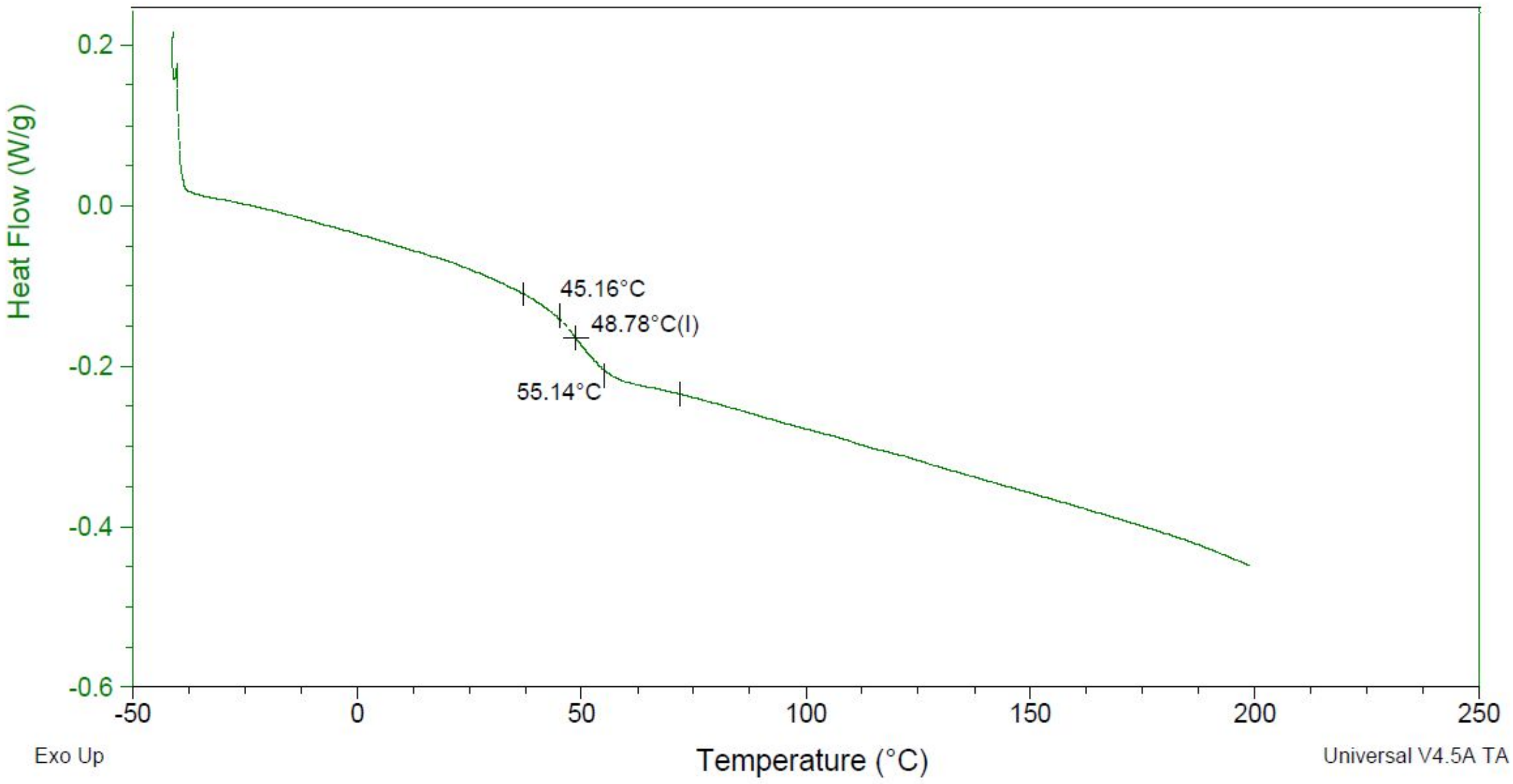

Figure S47. DSC spectrum for NIPU **8** (uncrystallized CADC mixture used)

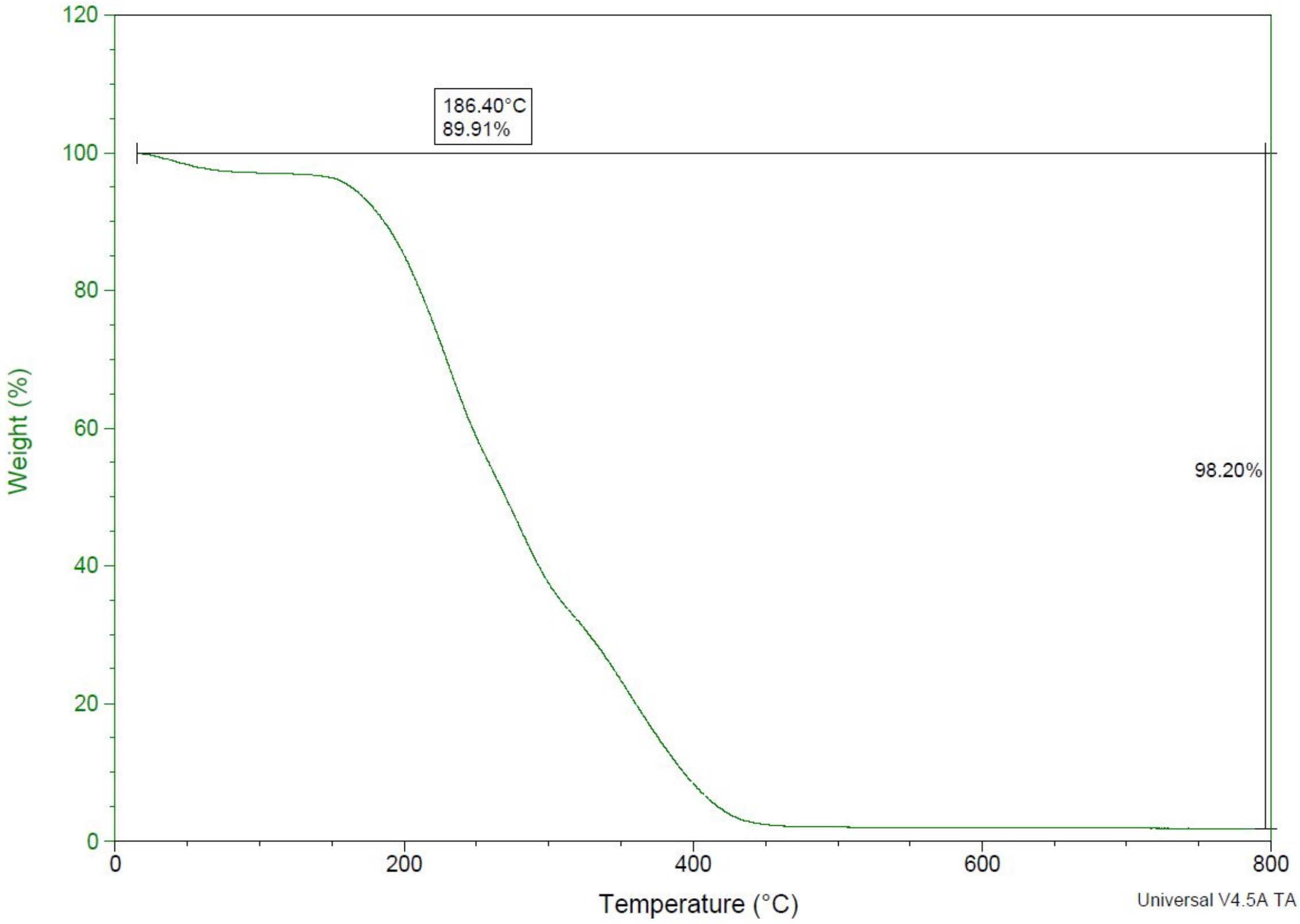

Figure S48. TGA graph for NIPU **9**

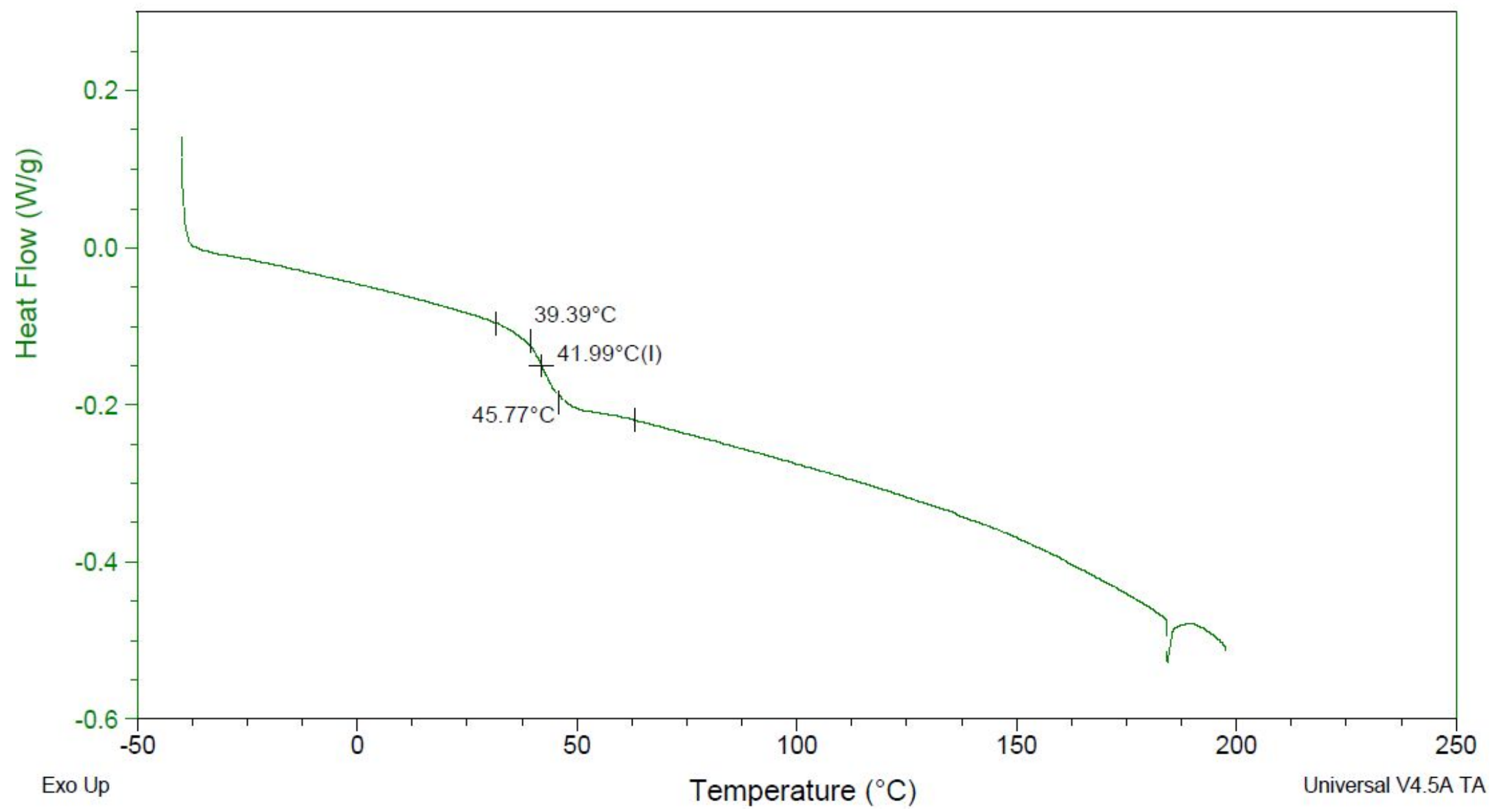

Figure S49. DSC spectrum for NIPU **9**

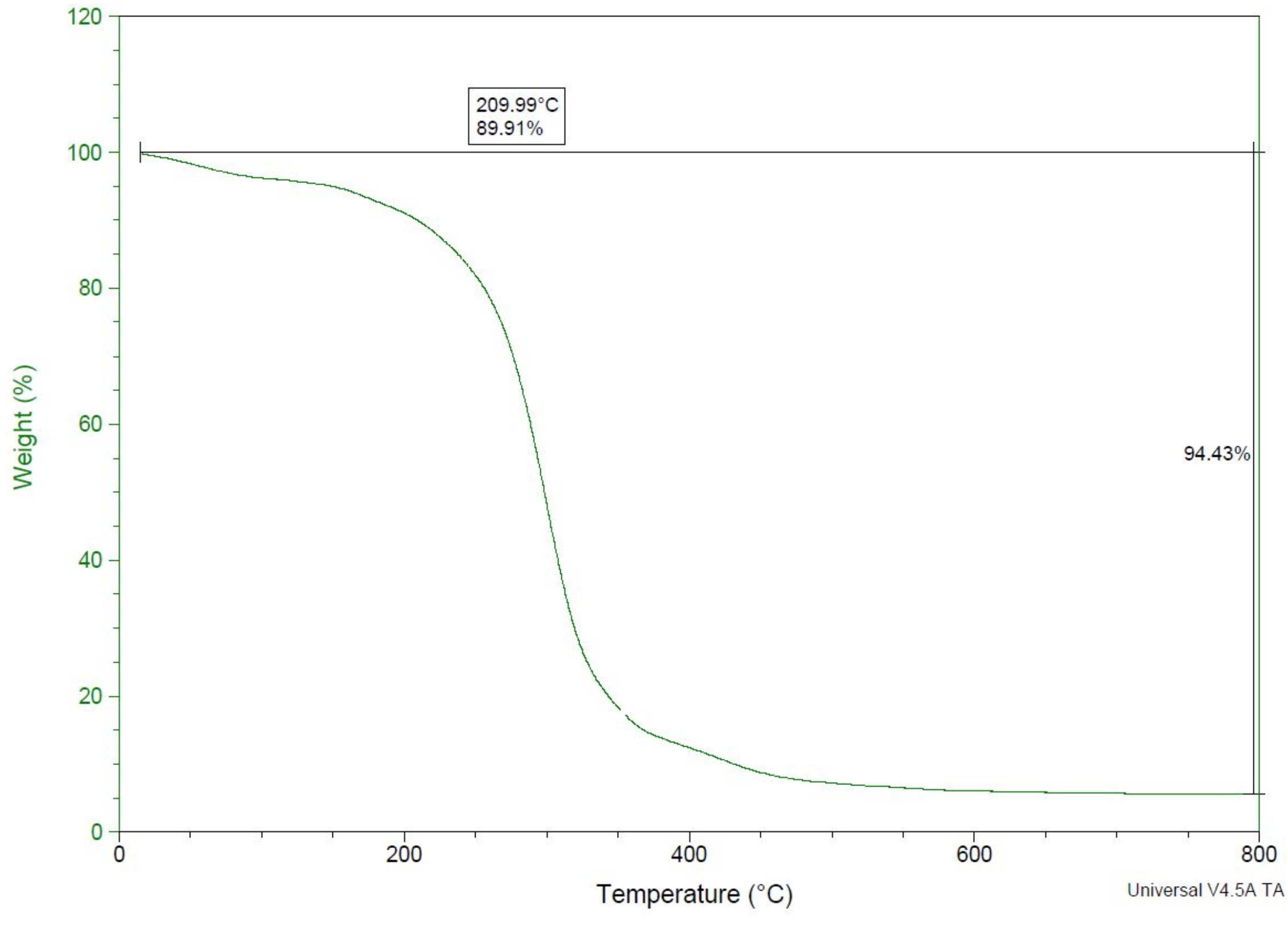

Figure S50. TGA graph for NIPU **10**

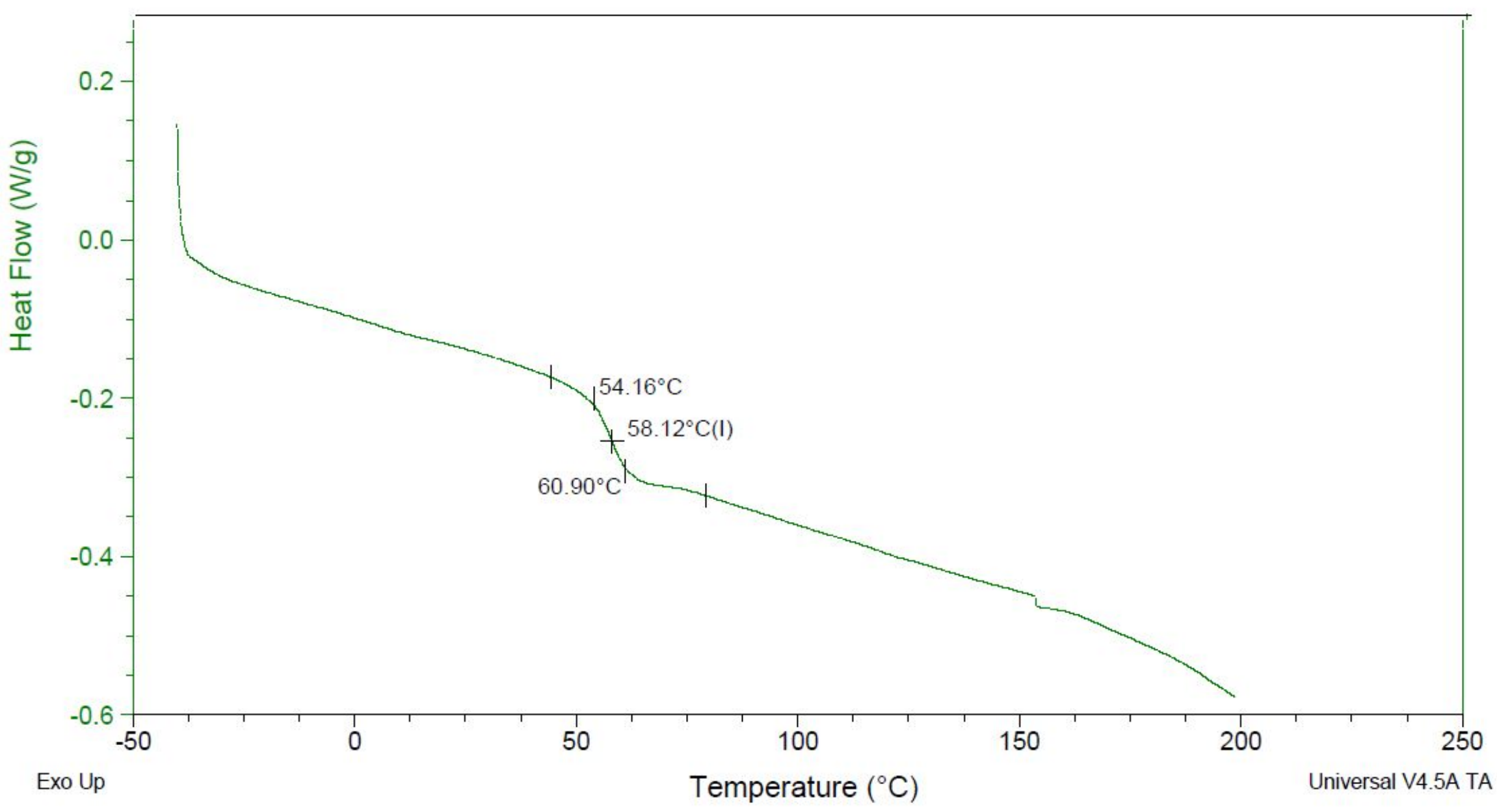

Figure S51. DSC spectrum for NIPU **10**

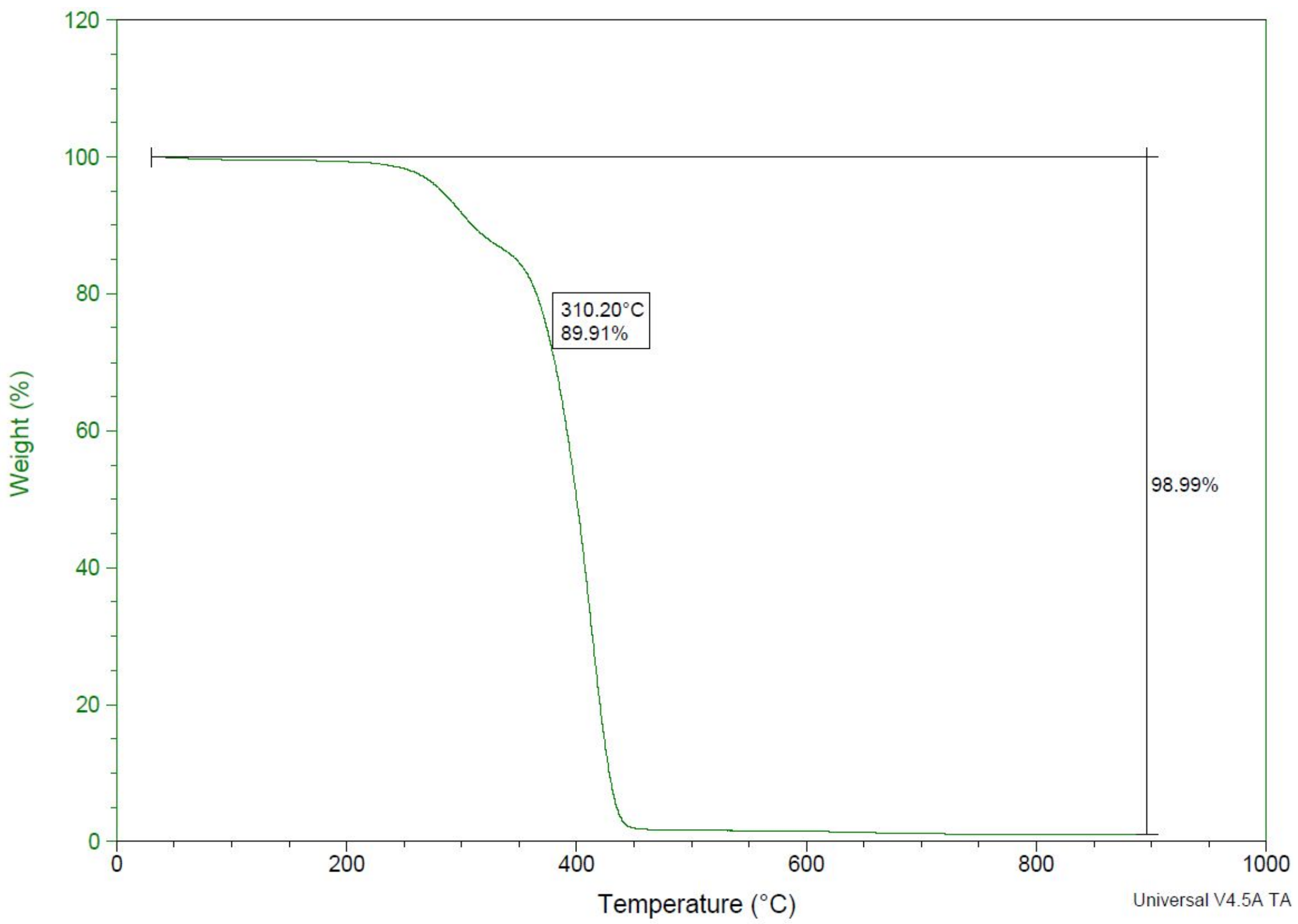

Figure S52. TGA graph for NIPU **11**

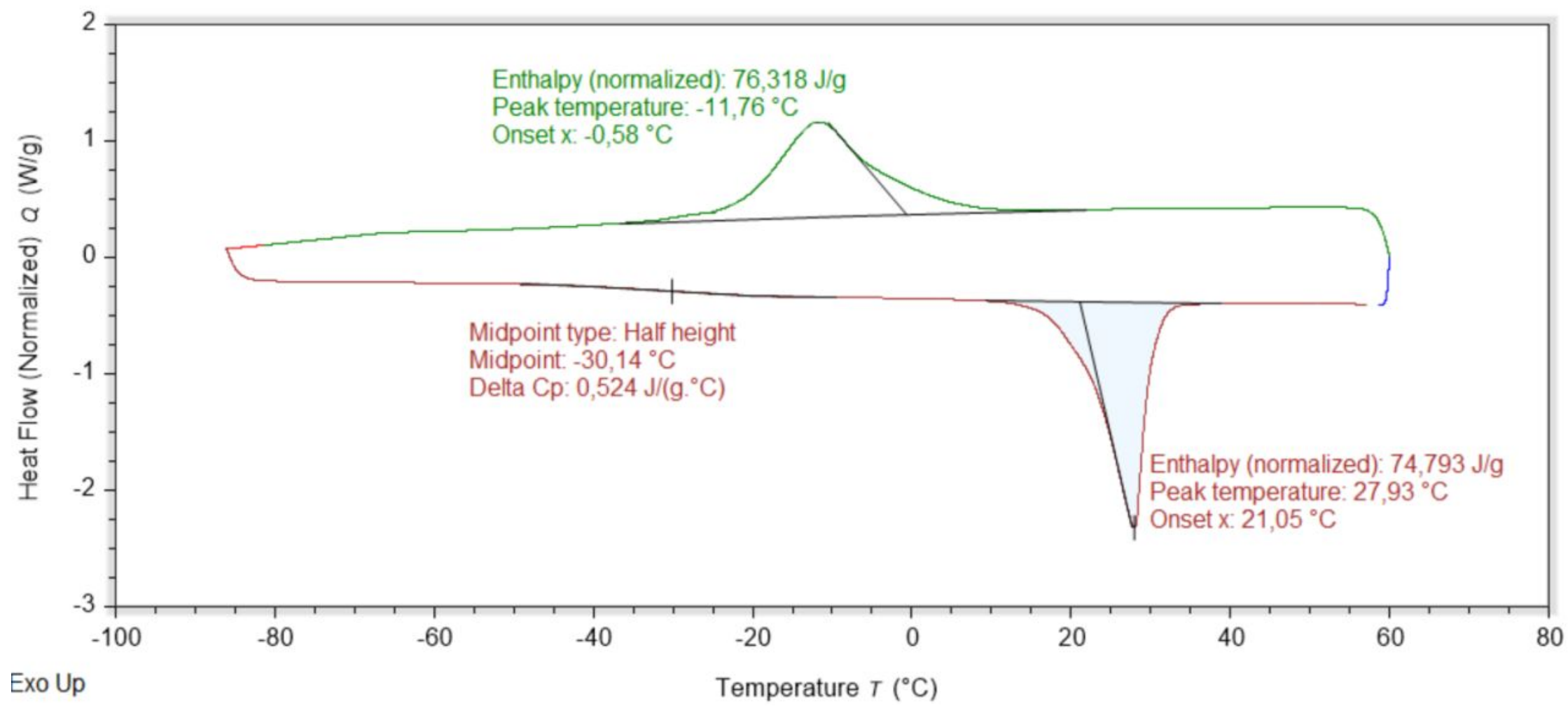

Figure S53. DSC spectrum for NIPU **11**

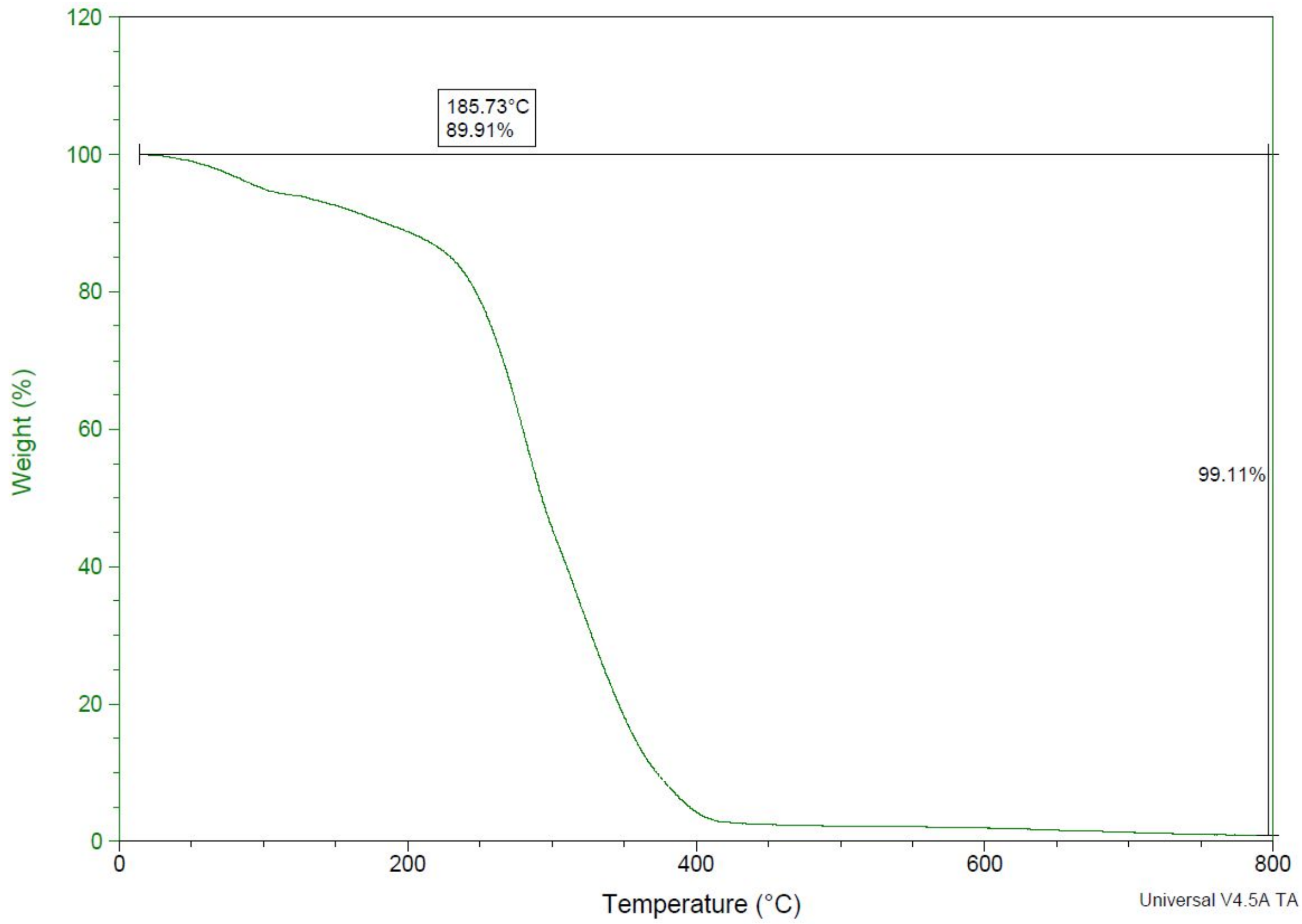

Figure S54. TGA graph for NIPU **12**

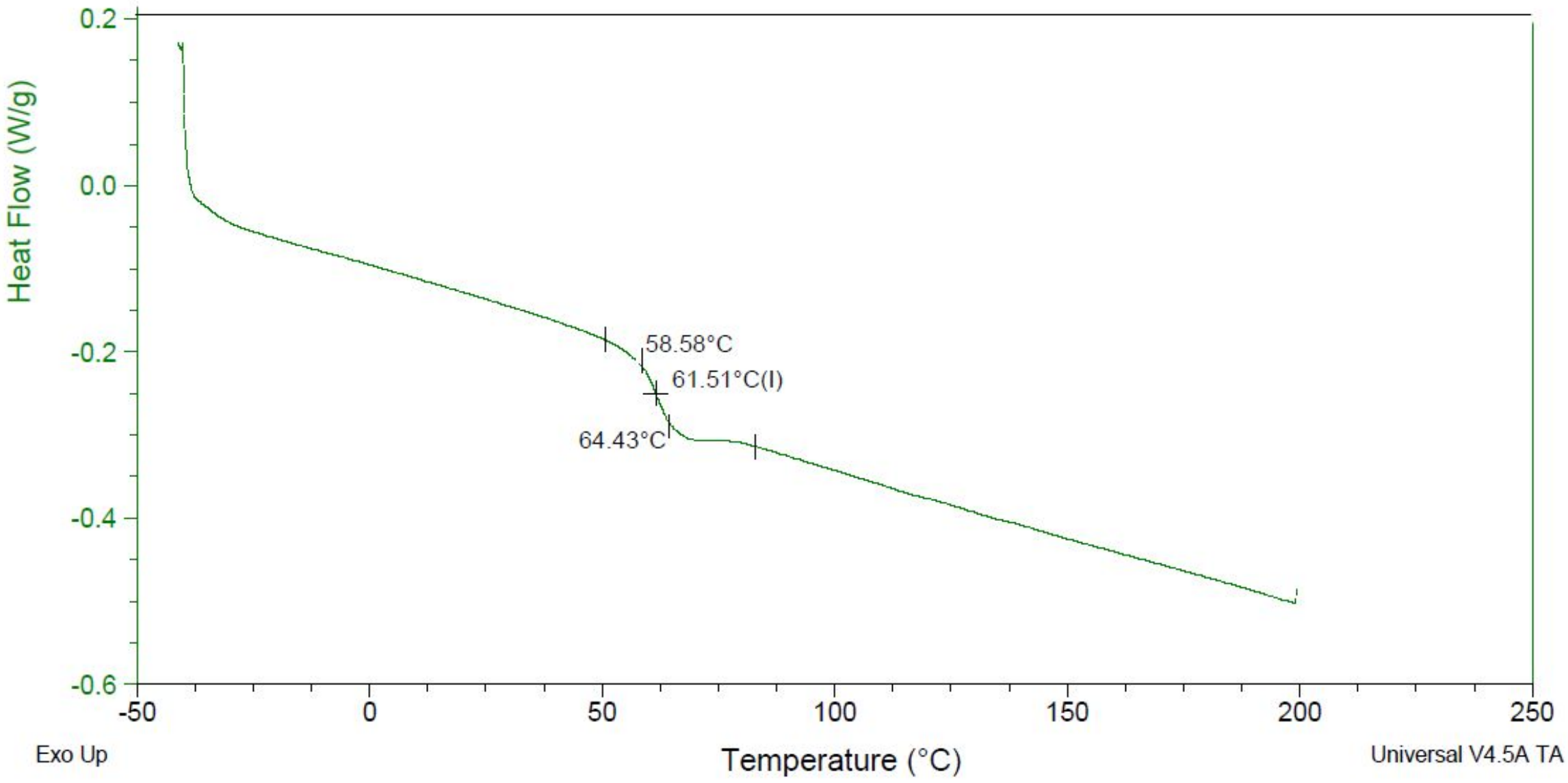

Figure S55. DSC trace for NIPU **12**

S45. SEC TRACES for NIPUs thermoplastics 8-12

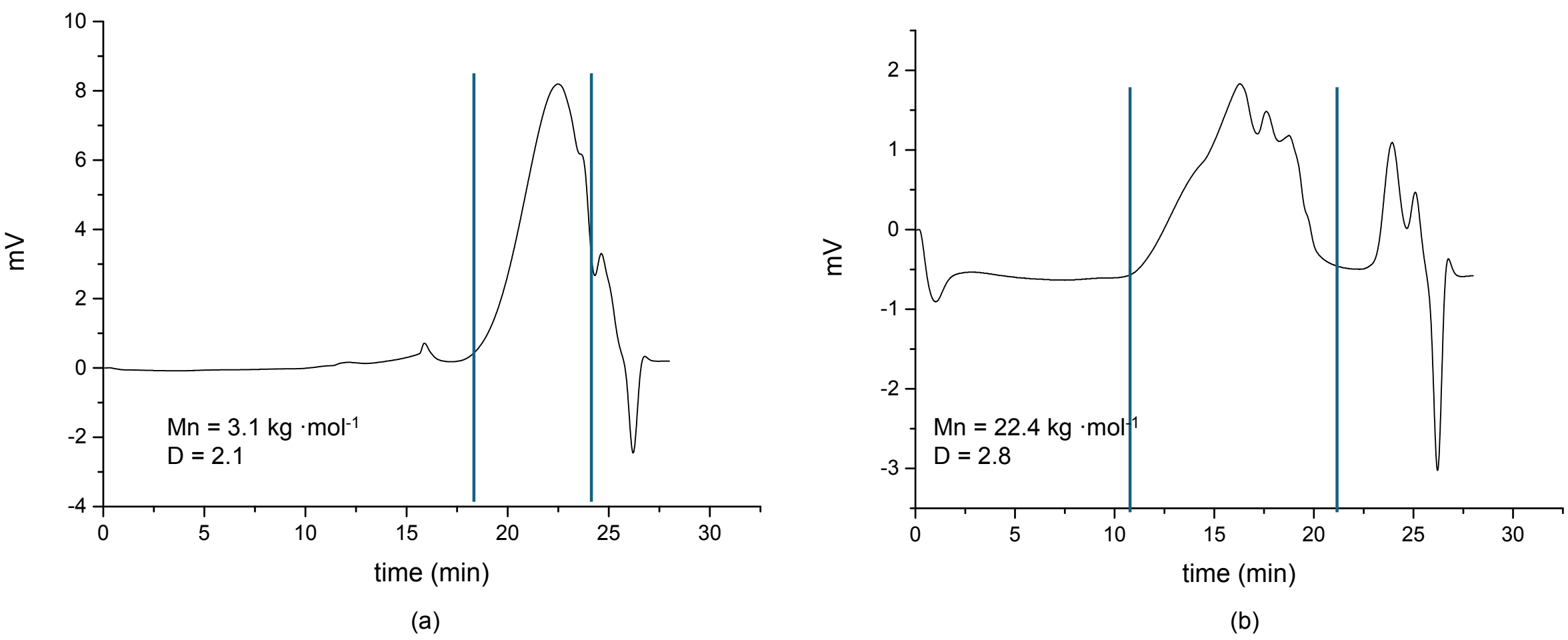

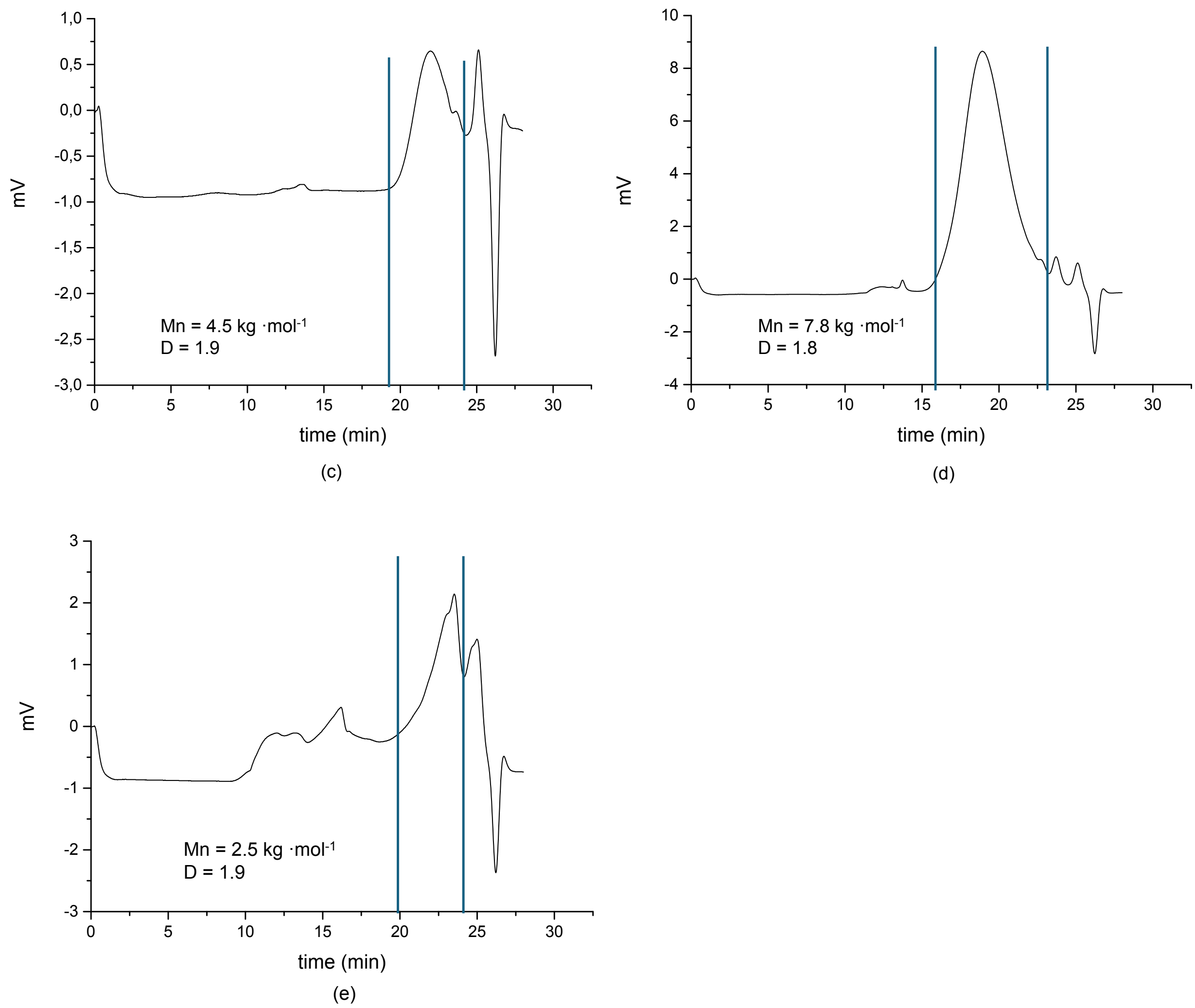

Figure S56. SEC traces for NIPUs **8–12**: a) NIPU **8**, b) NIPU **9**, c) NIPU **10**, d) NIPU **11** and e) NIPU **12**

S46. NIPU 11-LAROMER® LR9000 cross-linking tests

In a 5 mL glass vial equipped with a small stir bar, NIPU **11** (0.40–0.60 g) was added and warmed up to 90 ºC until it melted. Then, the corresponding amount of LAROMER® LR9000 (see Table S4) was added with a syringe and the mixture was left stirring for 45 seconds. After that time, the resuting viscous mixture was transferred with an oven pre-heated syringe to a teflon mold. For the curing process, the resulting formulations were placed in the oven at 100 ºC for two hours followed by post-curing at room temperature for 24 h. Altough several formulations were tested, only **11**_LAR40 NIPU resulted in a dry coating suitable for further mechanical testing. **11**_LAR50 and **11**_LAR60 formulations resulted too sticky to be demolded, preventing further analysis and showing potential adhesive applications.

Table S5. NIPU 11-LAROMER® LR9000 formulations

Entry	NIPU	LAROMER® LR9000 [wt%]	NIPU **11** [wt%]
1	**11**_LAR60	60	40
2	**11**_LAR50	50	50
3	**11**_LAR40	40	60

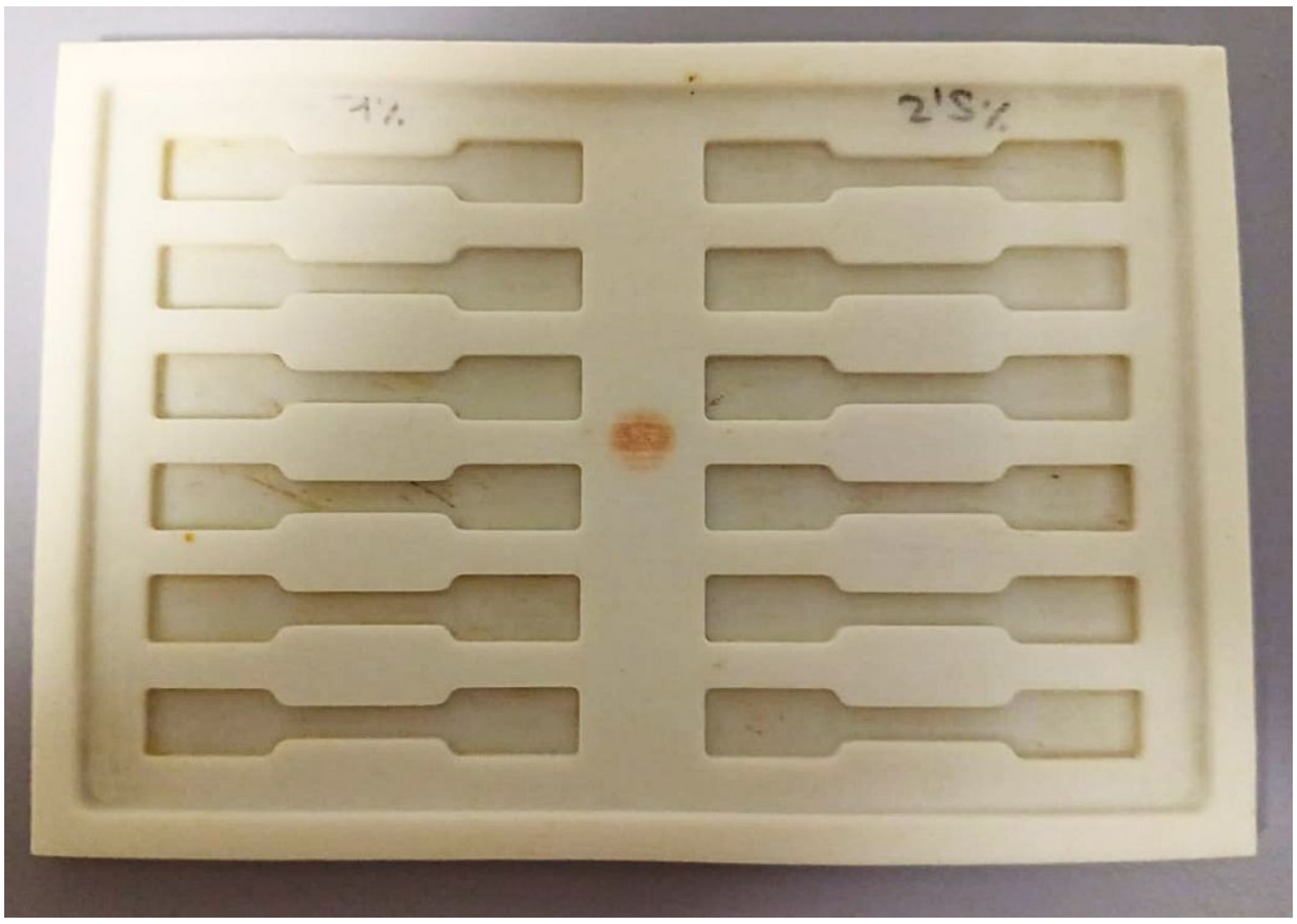

Figure S57. Teflon mold for NIPU tensile test specimens

S47. Tensile testing results of 11LAR_40 (ISO 37)

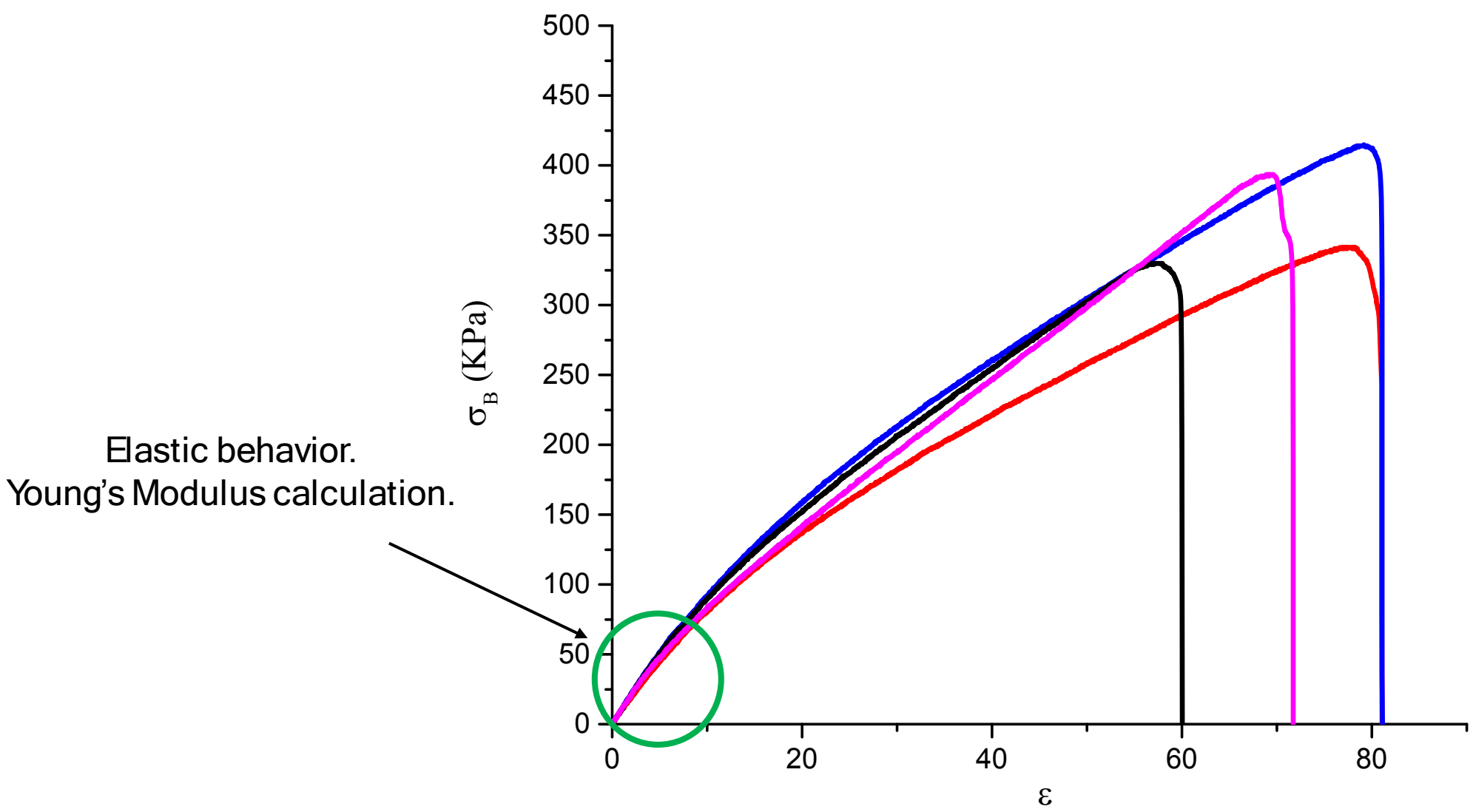

	nº	E_t (KPa)	ε_{tB} (%)	σ_B (KPa)	A_o (mm^2)	h (mm)
red	1	824.1	9.4	77.7	21.0	7.6
blue	2	913.7	10.6	95.7	20.7	8.4
black	3	913.9	10.0	89.7	21.0	8.0
magenta	4	834.1	9.6	80.6	20.9	8.0

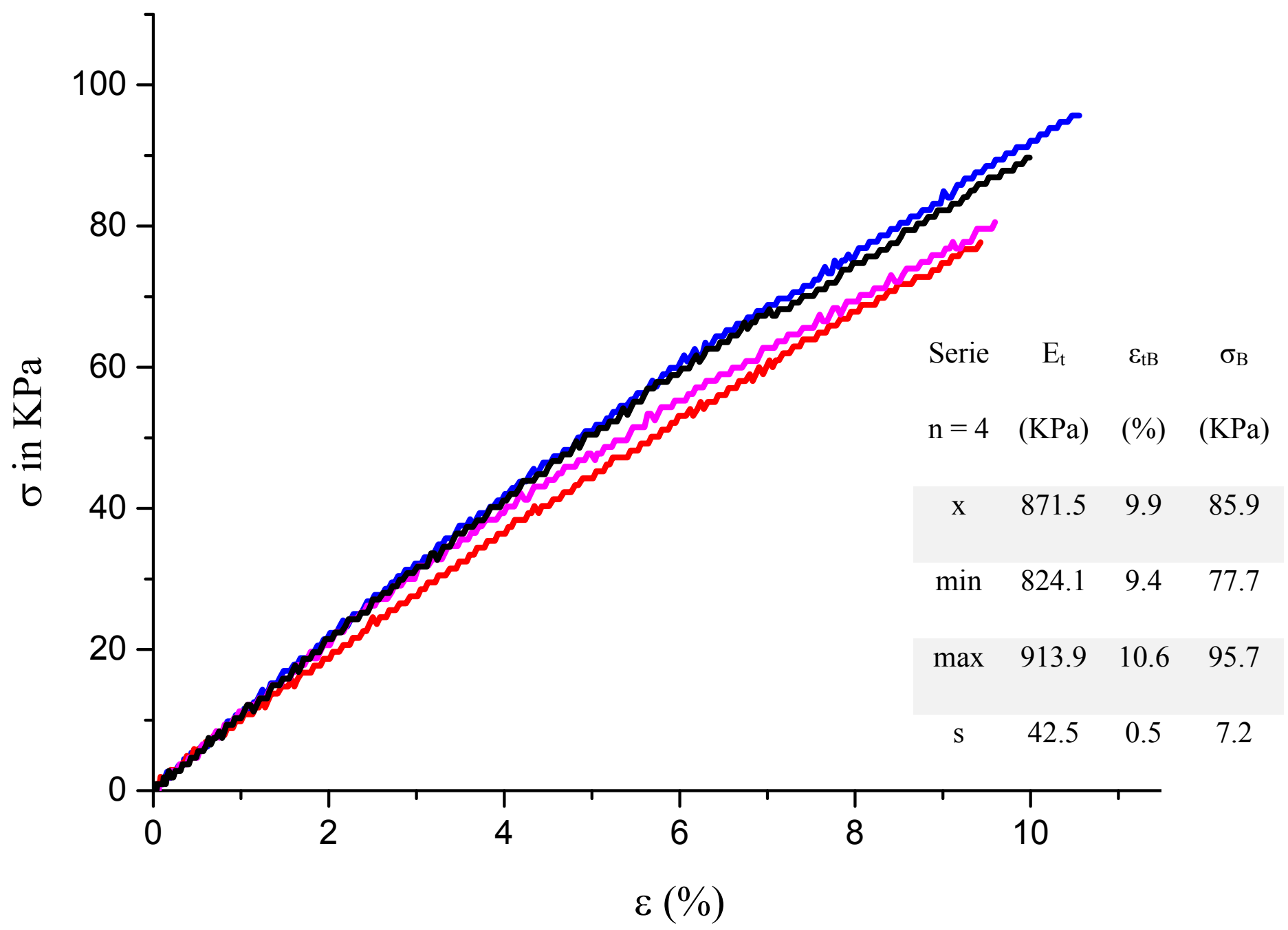

Figure S58. Tensile testing results of **11**LAR_40 (ISO 37)

S48. TGA graph and DSC trace of 11LAR_40 thermoset

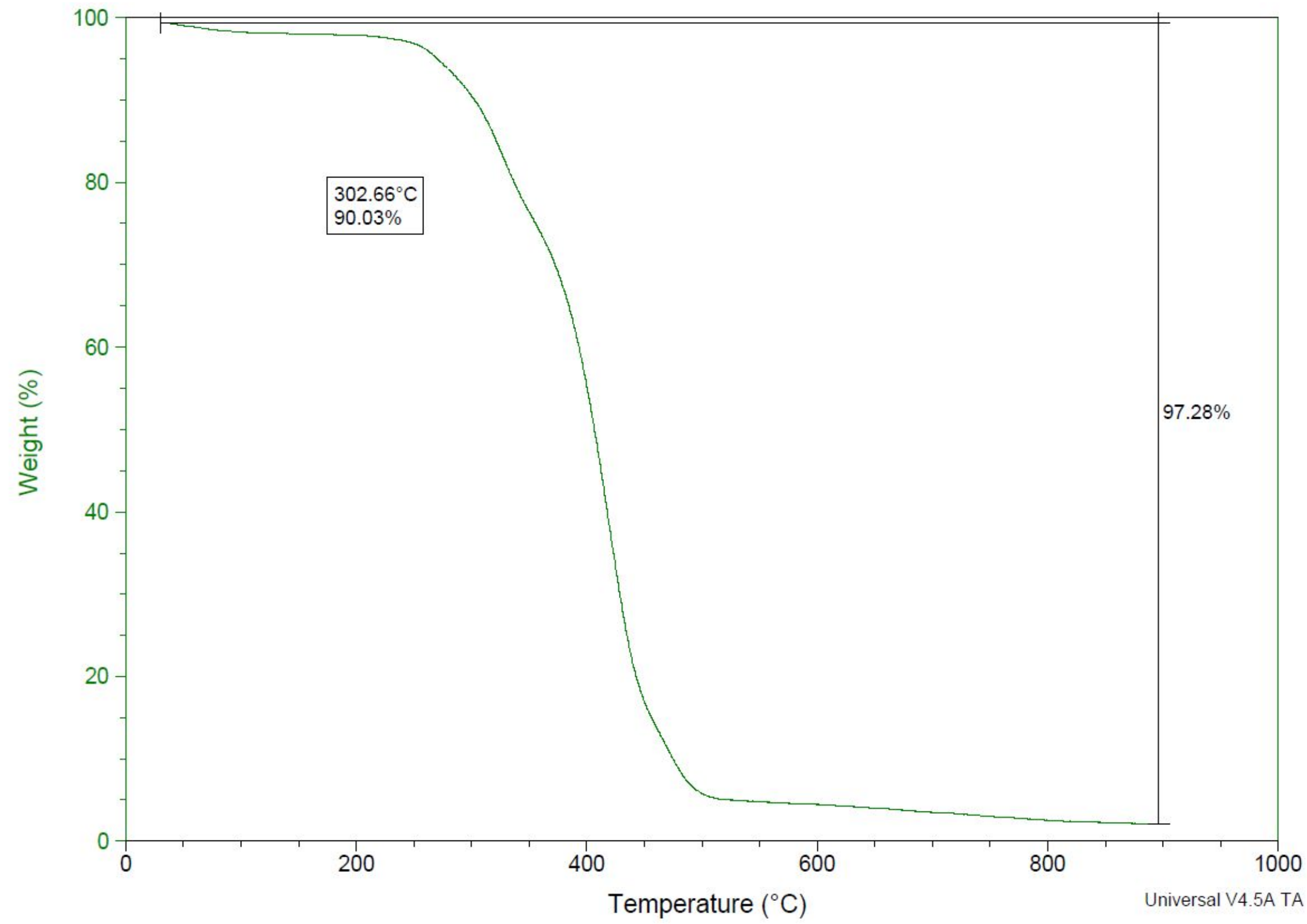

Figure S59. TGA graph of **11**LAR_40

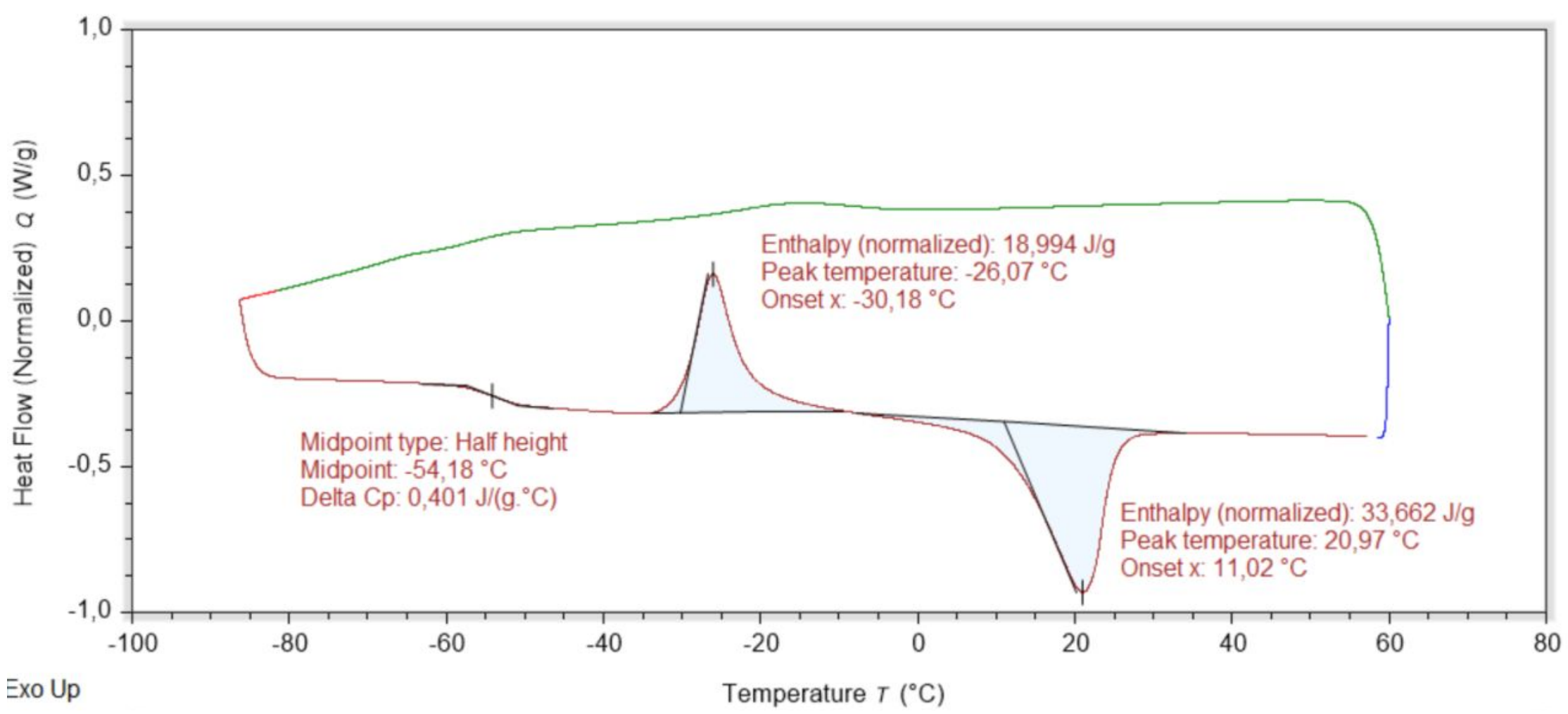

Figure S60. DSC trace of **11**LAR_40

S49. Swelling tests results of 11LAR_40 thermoset

Water swelling of **11**_LAR40 NIPU was evaluated using water content and absorption measurements at room temperature. Samples with dimensions of 0.5 cm length, 0.5 cm width, and 0.1 cm thickness were immersed in 20 mL of pure H_2O at room temperature. The water uptake was measured at the desired times until the sample weights remained constant.

SAMPLES	1	2	3
**Initial mass (g)**	0.1029	0.1043	0.0913
**Mass after 5 and ½ h**	0.2435	0.2430	0.2300
**Mass after 12 and ½ h**	0.24356	0.2437	0.2307
**Mass after 40 h**	0.24375	0.2439	0.2315
**EWC (%)**	136.9	133.8	153.6

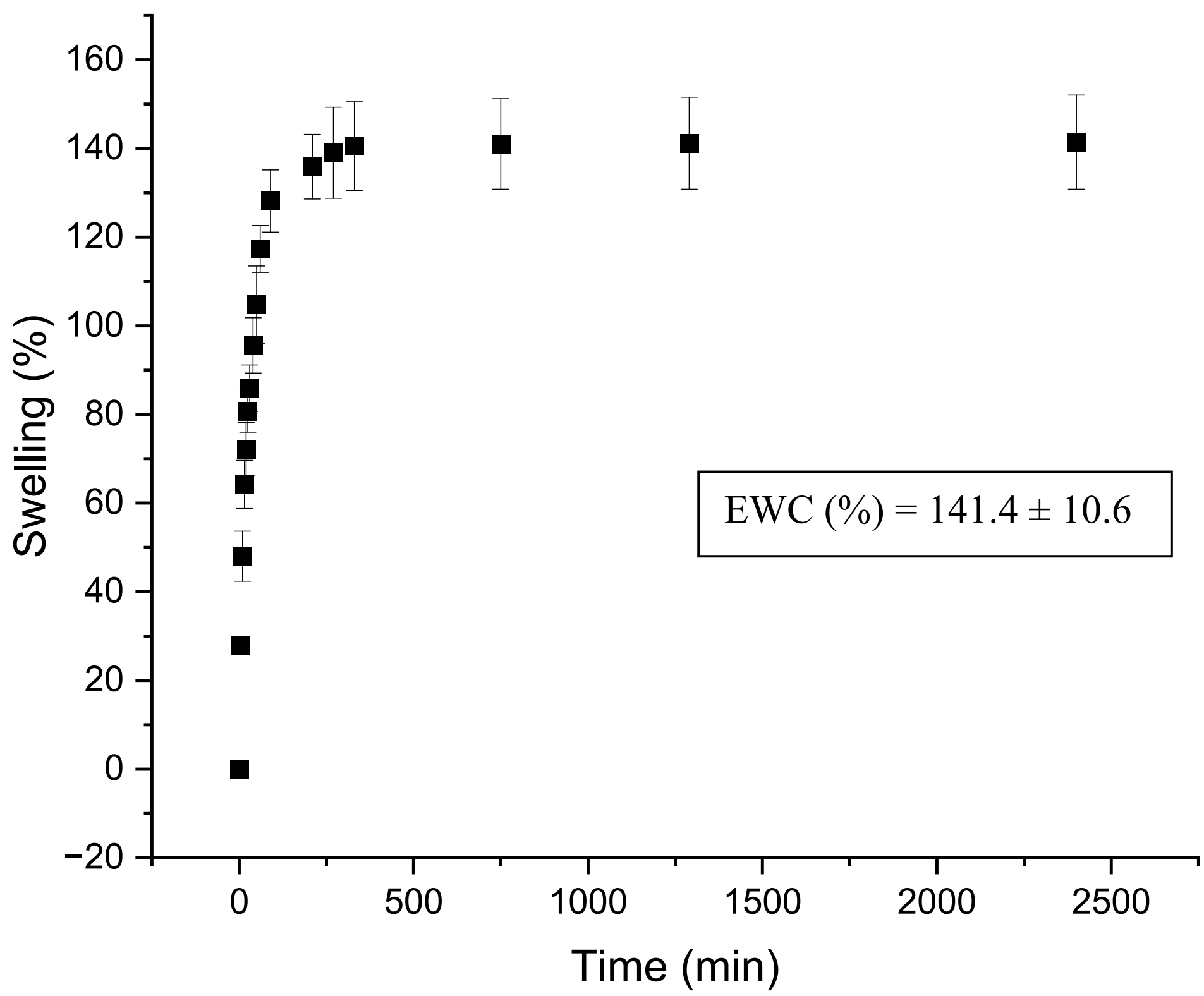

Figure S61. Swelling test results of **11**LAR_40

S50. CADC/BGDC blends-PEI cross-linking tests

In a 5 mL glass vial equipped with a small stir bar, the corresponding amount of bis(cyclic carbonates) purified CADC and BGDC were added and the solid mixture was warmed up until they melted. Then, the corresponding amount of PEI was added with a syringe and the resulting mixture was left stirring for 45 seconds. After that time, the resuting viscous mixture was transferred with an oven pre-heated syringe to a teflon mold. For the curing process, the resulting formulations were placed in the oven at 100 ºC for two hours followed by post-curing at room temperature for 24h. Altough several formulations were tested, only Gly_70CADC_PEI, Gly_50CADC_PEI and Gly_30CADC_PEI NIPUs could be demolded and further analyed by mechanical testing. On the other hand, CADC_PEI and Gly_90CADC_PEI tensile specimens turned out too brittle and broke when demolding.

Table S6. Melting temperatures CADC/BGDC blends

Entry	NIPU	m_f BGDC [wt%]	Melting T (ºC)
1	CADC_PEI	0	120
2	Gly_90CADC_PEI	10	110
3	Gly_70CADC_PEI	30	100
4	Gly_50CADC_PEI	50	90
5	Gly_30CADC_PEI	70	90

S51. Stoichiometric calculations for CADC/BGDC-PEI cross-linked NIPUs

Table S7. Preparation of cyclic carbonate blends from Carvyl Acetate (CADC) and Glycidyl (BGDC)[a]

CC blend	m (Carvyl) [g]	n (Carvyl CO_3) [mmol]	m (Glycidyl) [g]	n (Glycidyl CO_3) [mmol]	m_f (Carvyl) [%]	Carbonate Value (mmol/g)
Gly_30CADC	0.225	1.432	0.525	3.617	30	6.732
Gly_50CADC	0.375	2.386	0.375	2.584	50	6.627
Gly_70CADC	0.525	3.341	0.225	1.550	70	6.521
Gly_90CADC	0.675	4.295	0.075	0.517	90	6.417
CADC	0.750	4.773	0	0	100	6.364

[a]0.75g per blend of cyclic carbonates

Table S8. Preparation of NIPU thermosets from CADC, BGDC and polyethyleneimine (PEI)

CC blend	m (CC blend) [g]	n (CO_3 blend) [mmol]	n (NH_2 PEI)	n PEI [mmol][a]	m PEI [g]
Gly_30CADC _PEI	0.75	5.049	5.049	0.616	0.369
Gly_50CADC _PEI	0.75	4.971	4.971	0.606	0.364
Gly_70CADC _PEI	0.75	4.891	4.891	0.597	0.358
Gly_90CADC _PEI	0.75	4.812	4.812	0.587	0.352
CADC _PEI	0.75	4.789	4.772	0.582	0.349

[a]Primary amine value kindly taken from *Macromolecules* 2017, *50*, 944.

S52. Tensile testing results of Gly_CADC_PEI thermosetting materials (ISO 37)

	nº	E_t (MPa)	ε_{tB} (%)	σ_B (MPa)	A_o (mm^2)	h (mm)
red line	1	364	2.8	9.8	18.2	9.0
blue line	2	298	3.0	8.8	18.5	8.4
black line	3	309	3.3	9.7	18.8	8.1

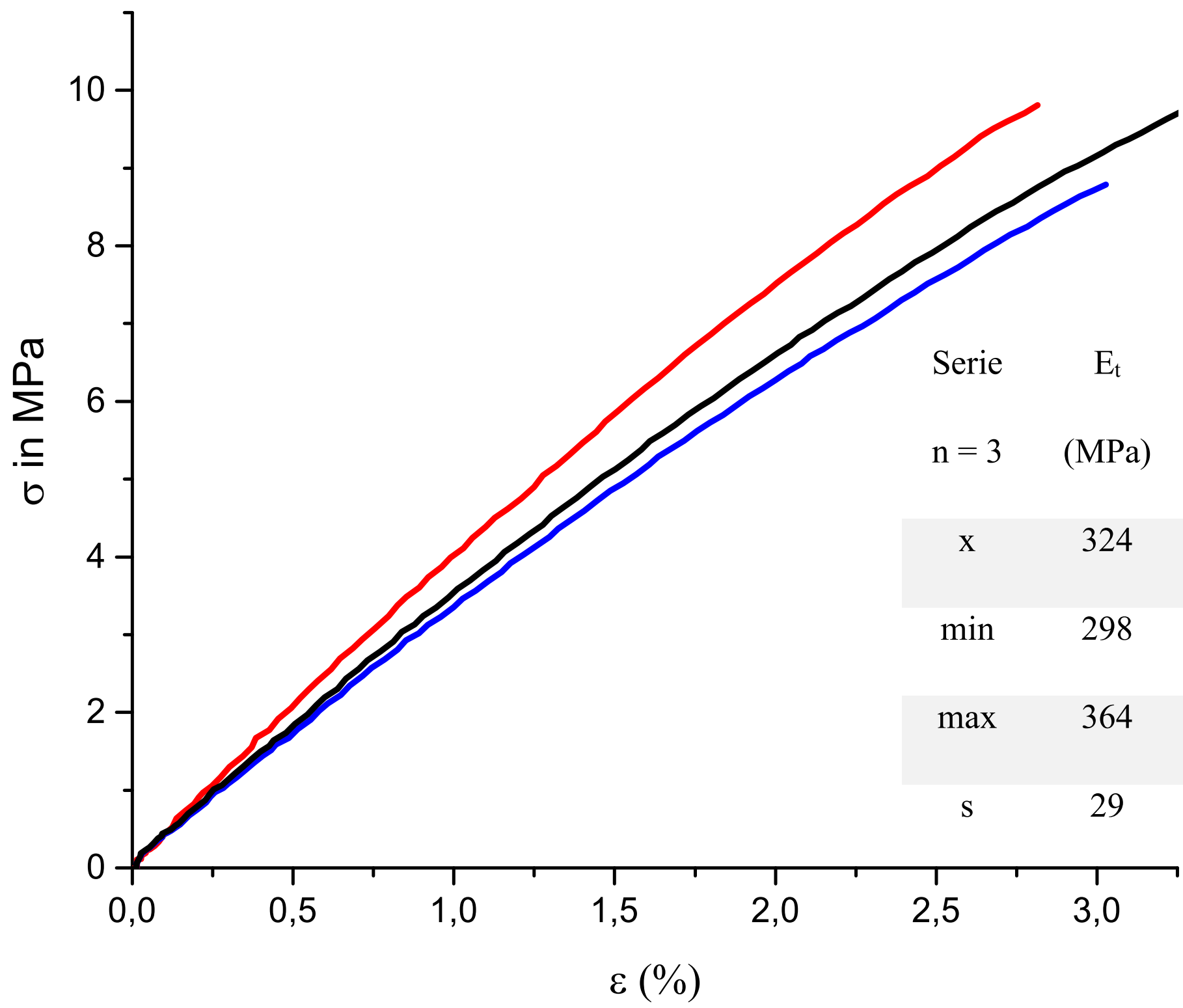

Serie n = 3	E_t (MPa)	ε_{tB} (%)	σ_B (MPa)
x	324	3.0	9.4
min	298	3.0	8.8
max	364	3.3	9.8
s	29	0.2	0.4

Figure S62. Tensile testing results of Gly_30CADC_PEI (ISO 37)

	nº	E_t (MPa)	ε_{tB} (%)	σ_B (MPa)	A_o (mm^2)	h (mm)
red	1	398	6.1	23.4	19.1	8.2
blue	2	497	4.2	20.4	18.3	9.5
black	3	426	6.0	25.2	18.4	9.0

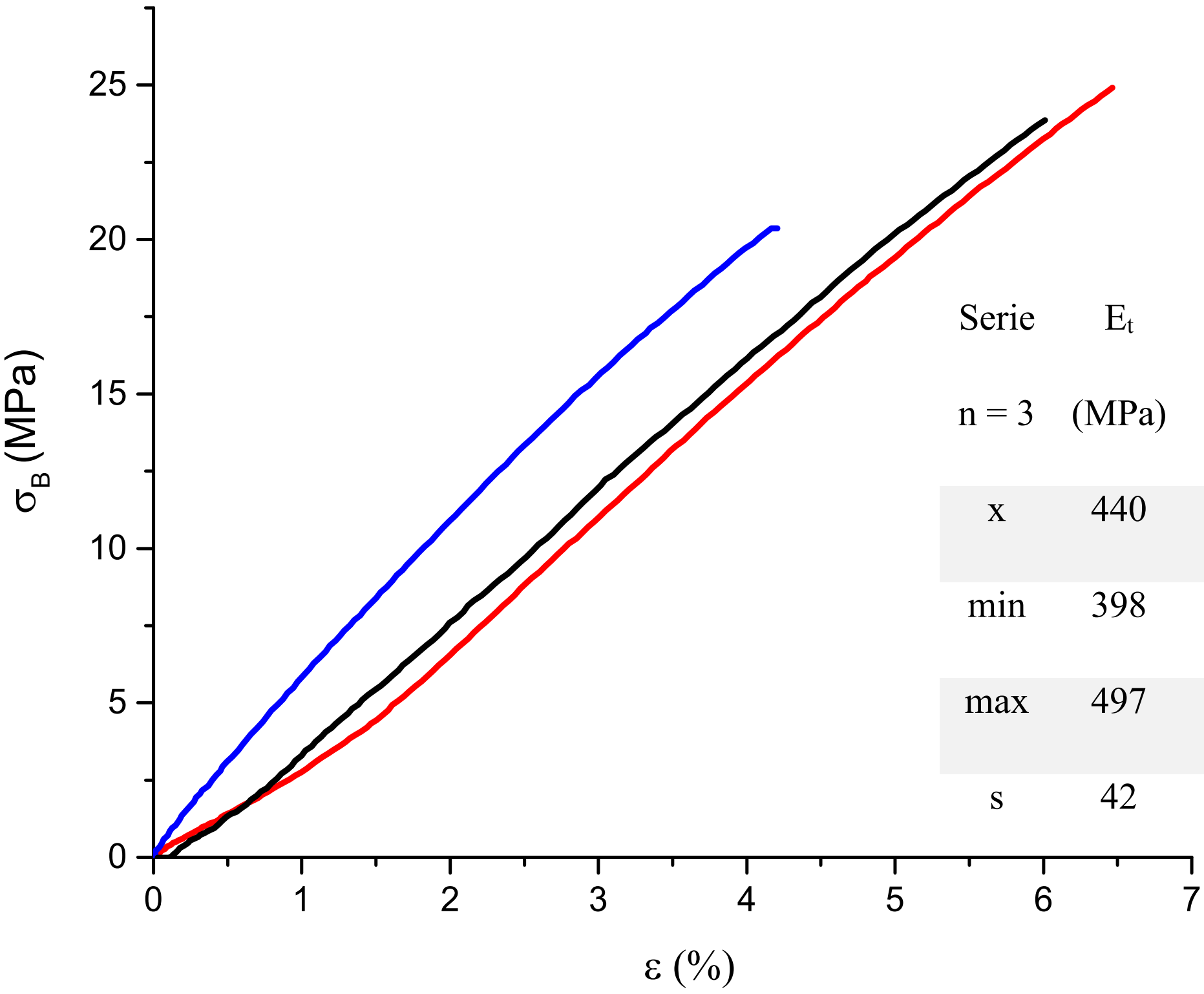

Serie n = 3	E_t (MPa)	ε_{tB} (%)	σ_B (MPa)
x	440	5.4	23.0
min	398	4.2	20.4
max	497		25.2
s	42	0.9	2.0

Figure S63. Tensile testing results of Gly_50CADC_PEI (ISO 37)

	nº	E_t (MPa)	ε_{tB} (%)	σ_B (MPa)	A_o (mm^2)	h (mm)
	1	695	4.2	28.5	18.5	8.3
	2	787	3.5	27.5	18.2	8.0
	3	774	3.1	23.1	18.2	8.0

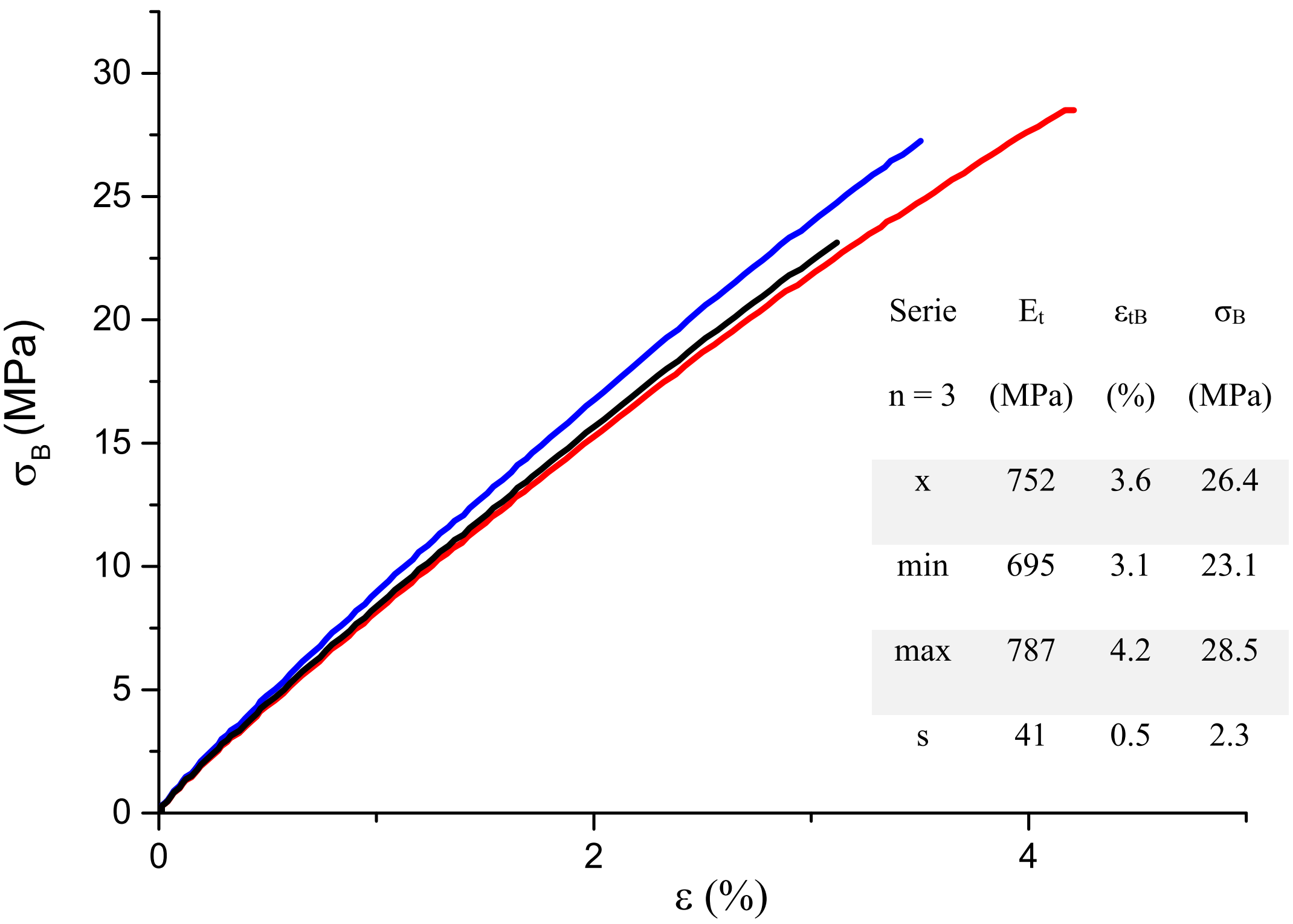

Figure S64. Tensile testing results of Gly_70CADC_PEI (ISO 37)

S55. TGA graphs and DSC traces of Gly_CADC_PEI thermosetting materials

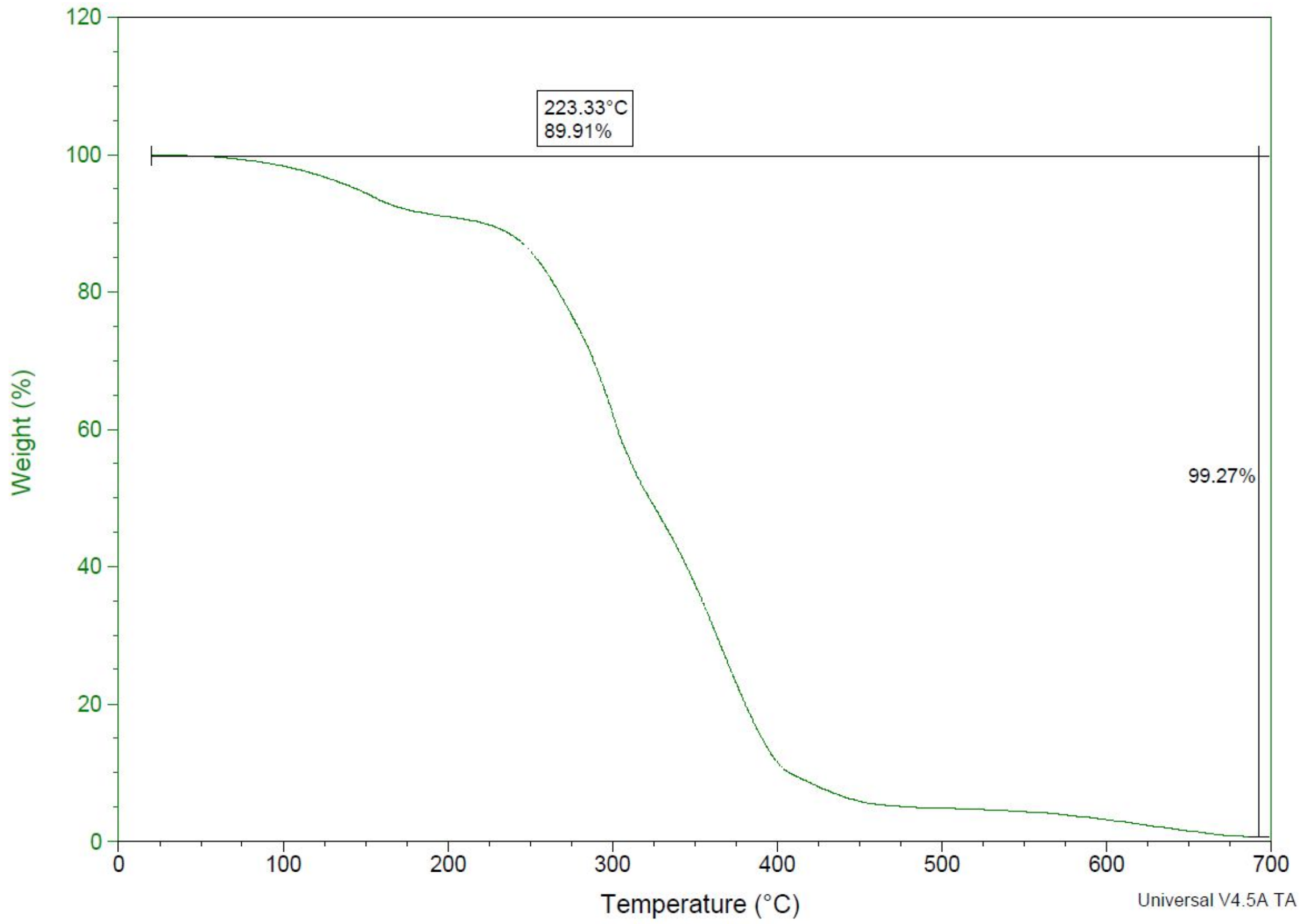

Figure S65. TGA graph of Gly_30CADC_PEI

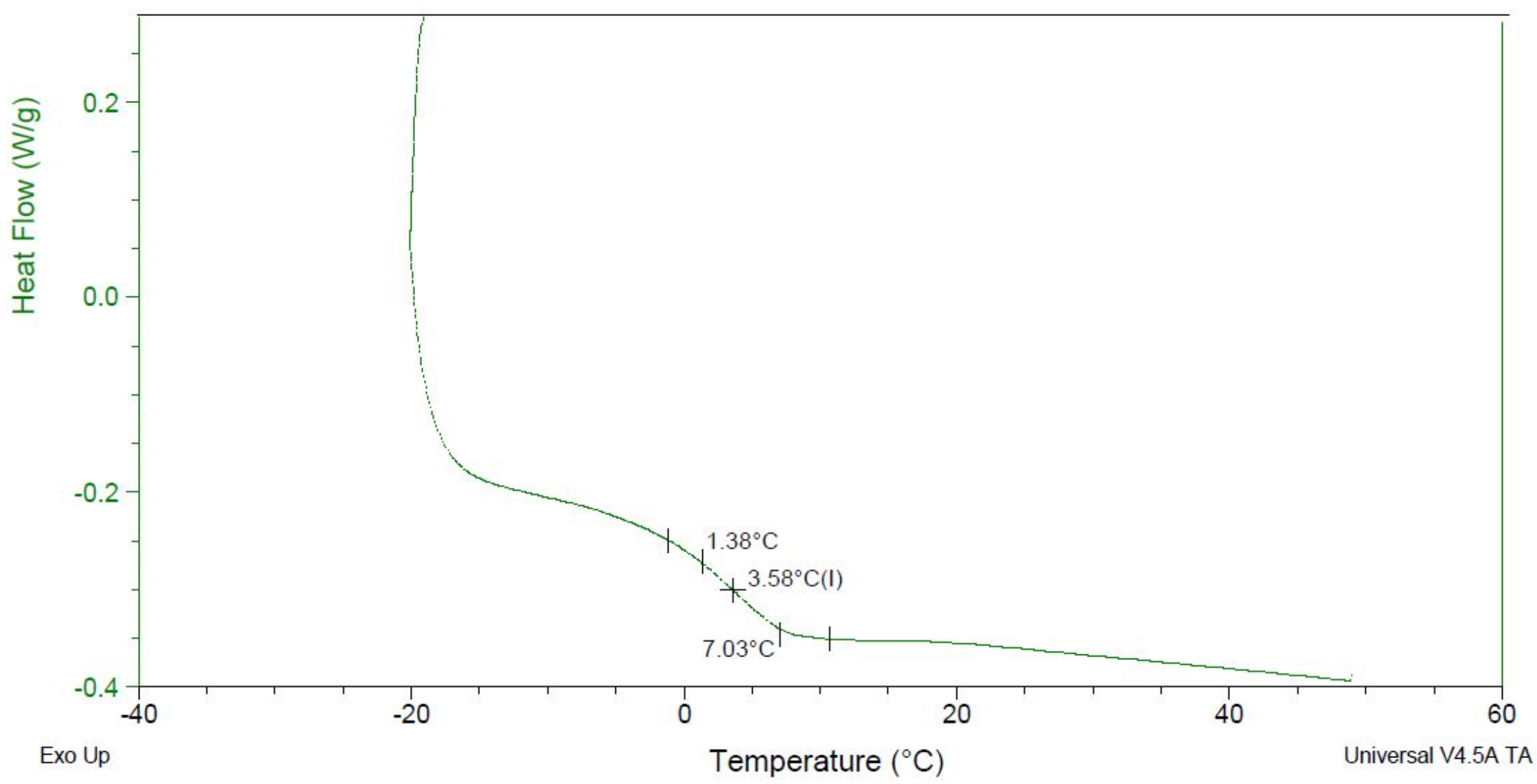

Figure S66. DSC trace of Gly_30CADC_PEI

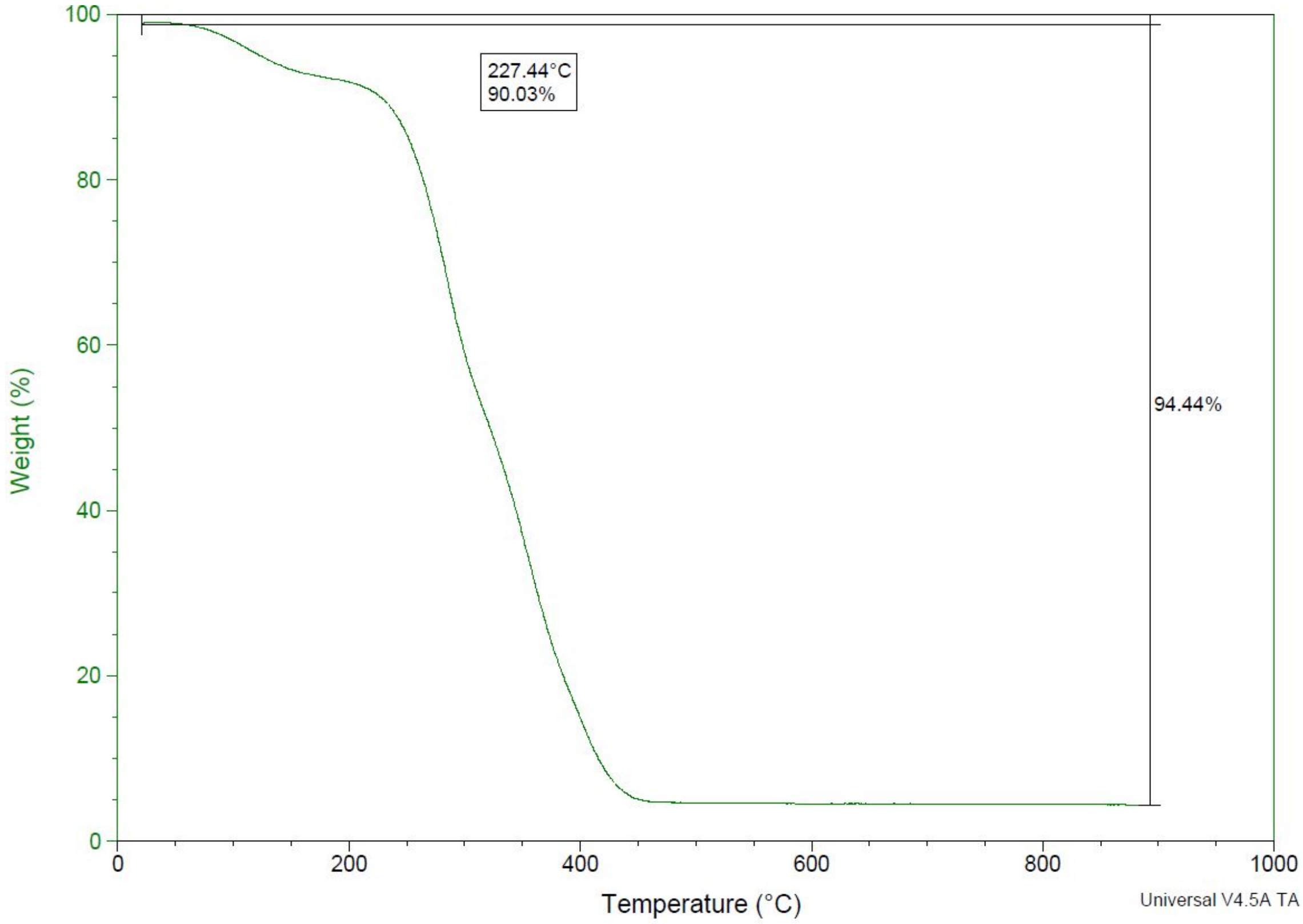

Figure S67. TGA graph of Gly_50CADC_PEI

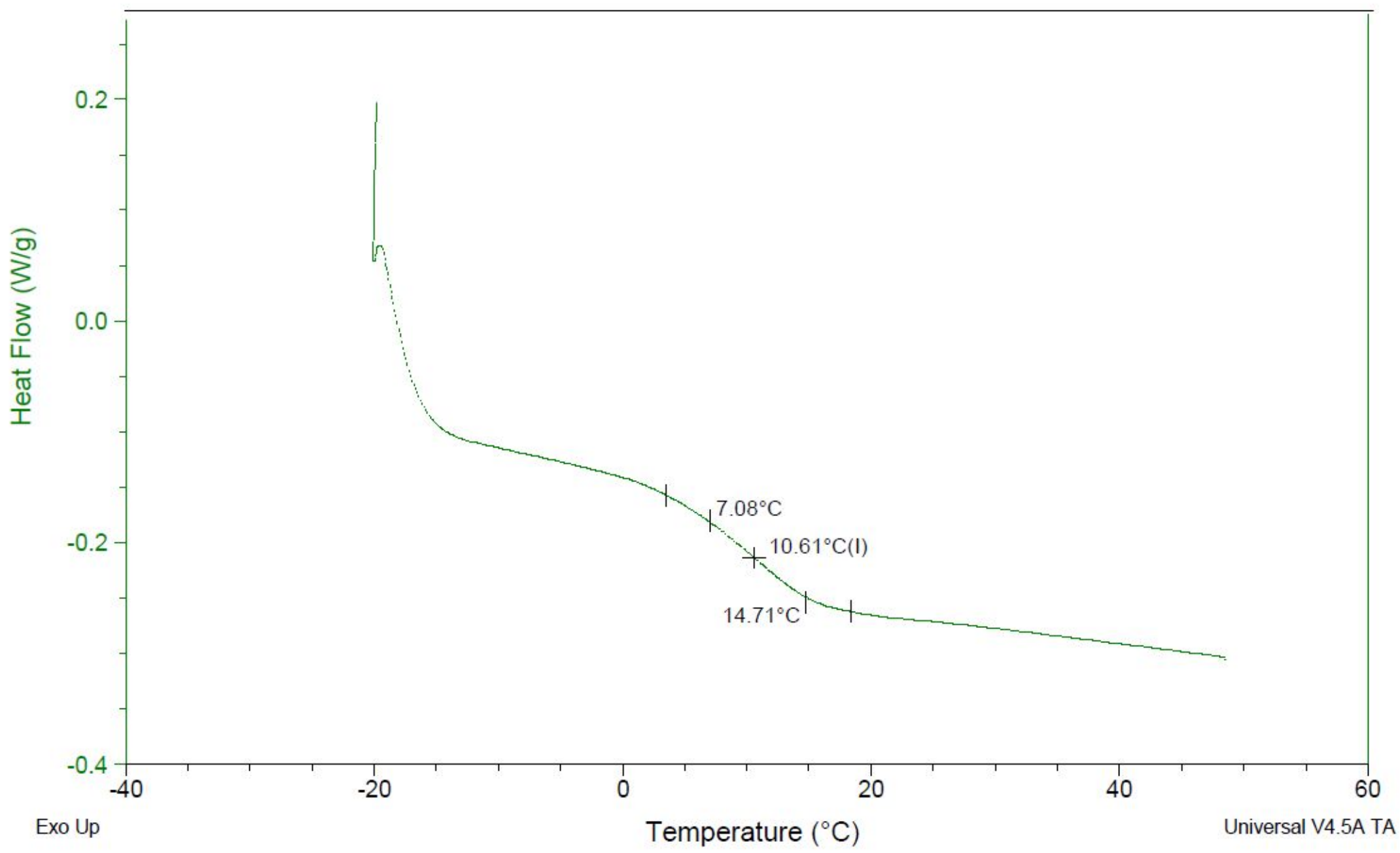

Figure S68. DSC trace of Gly_50CADC_PEI

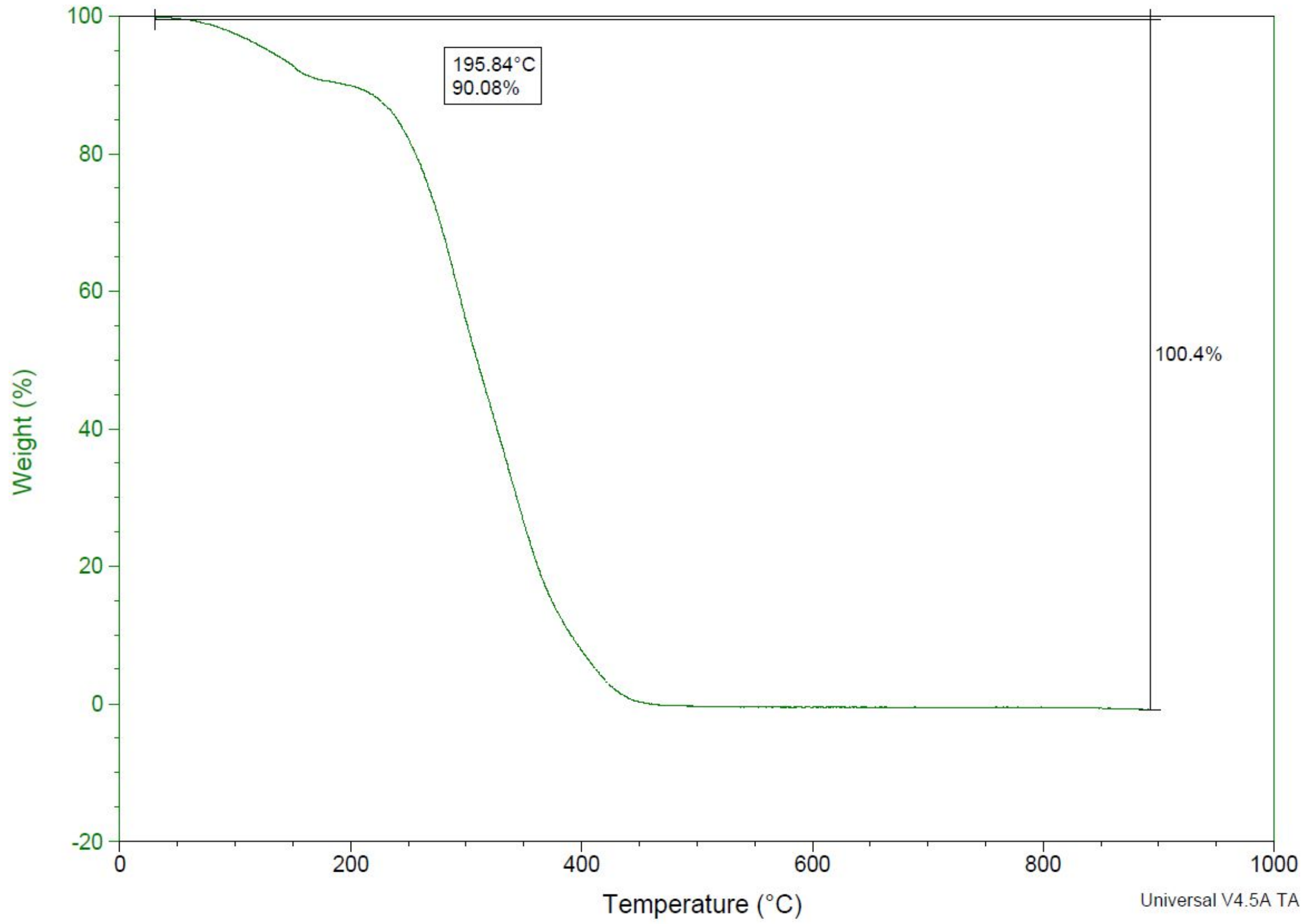

Figure S69. TGA graph of Gly_70CADC_PEI

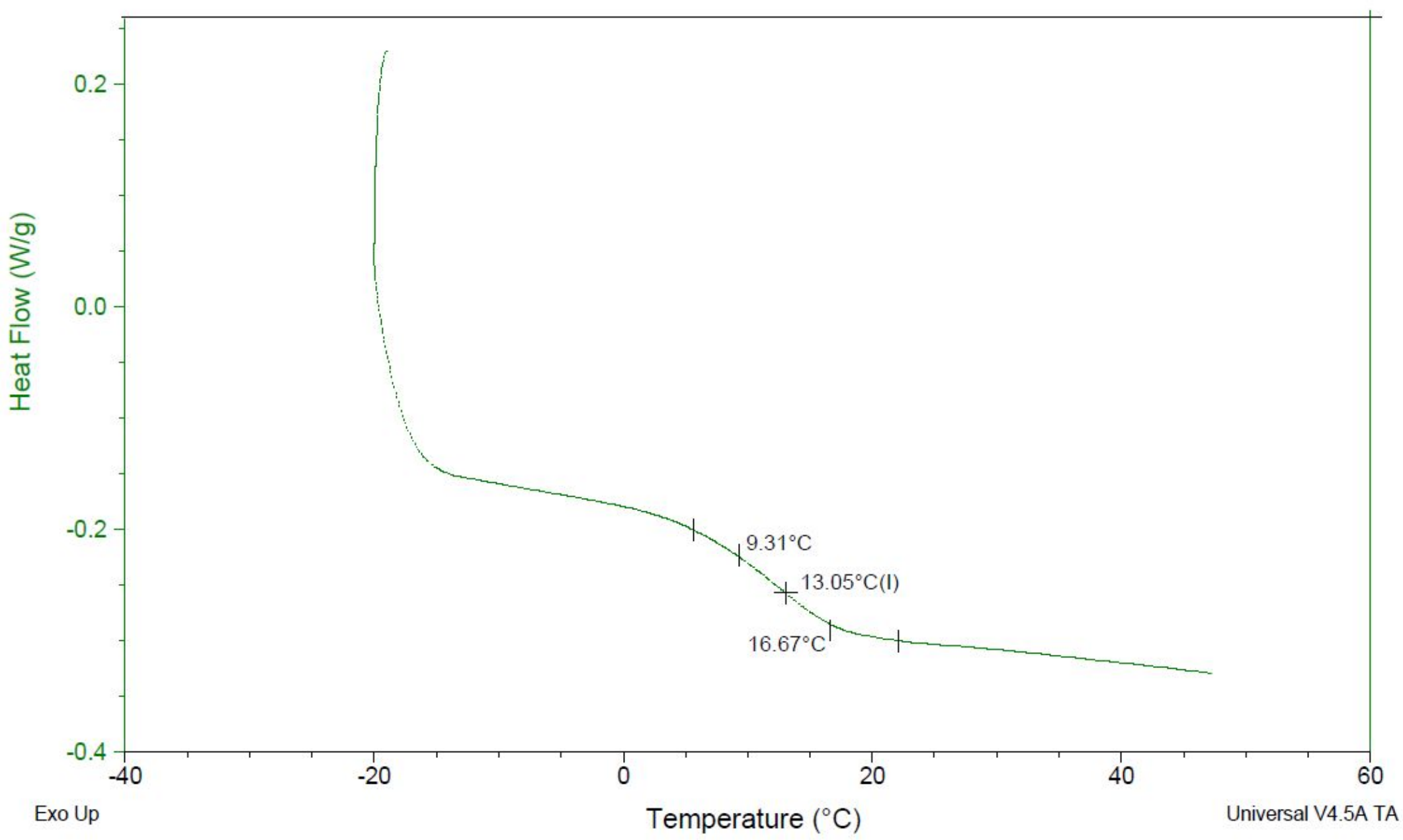

Figure S70. DSC trace of Gly_70CADC_PEI

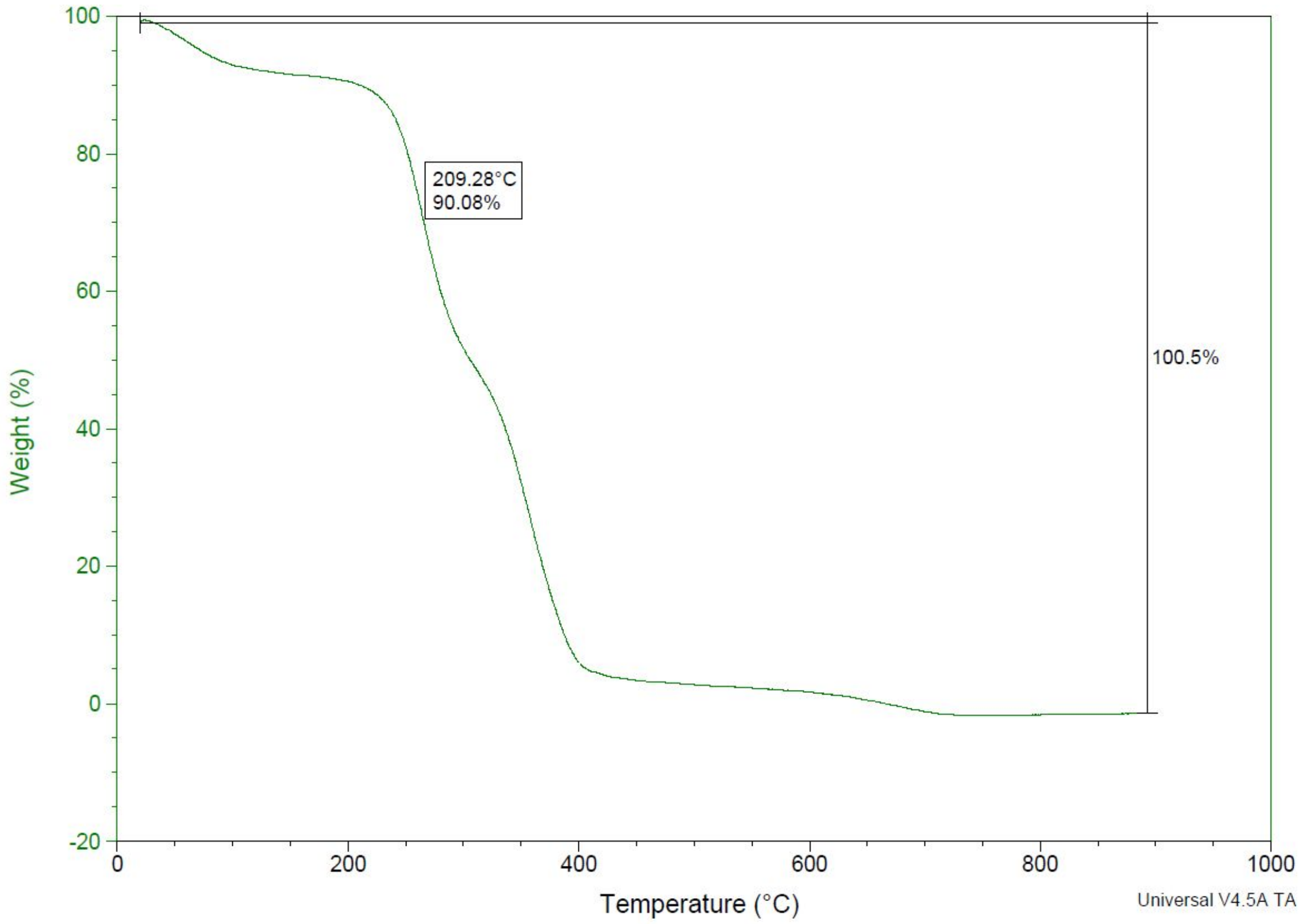

Figure S71. TGA graph of Gly_90CADC_PEI

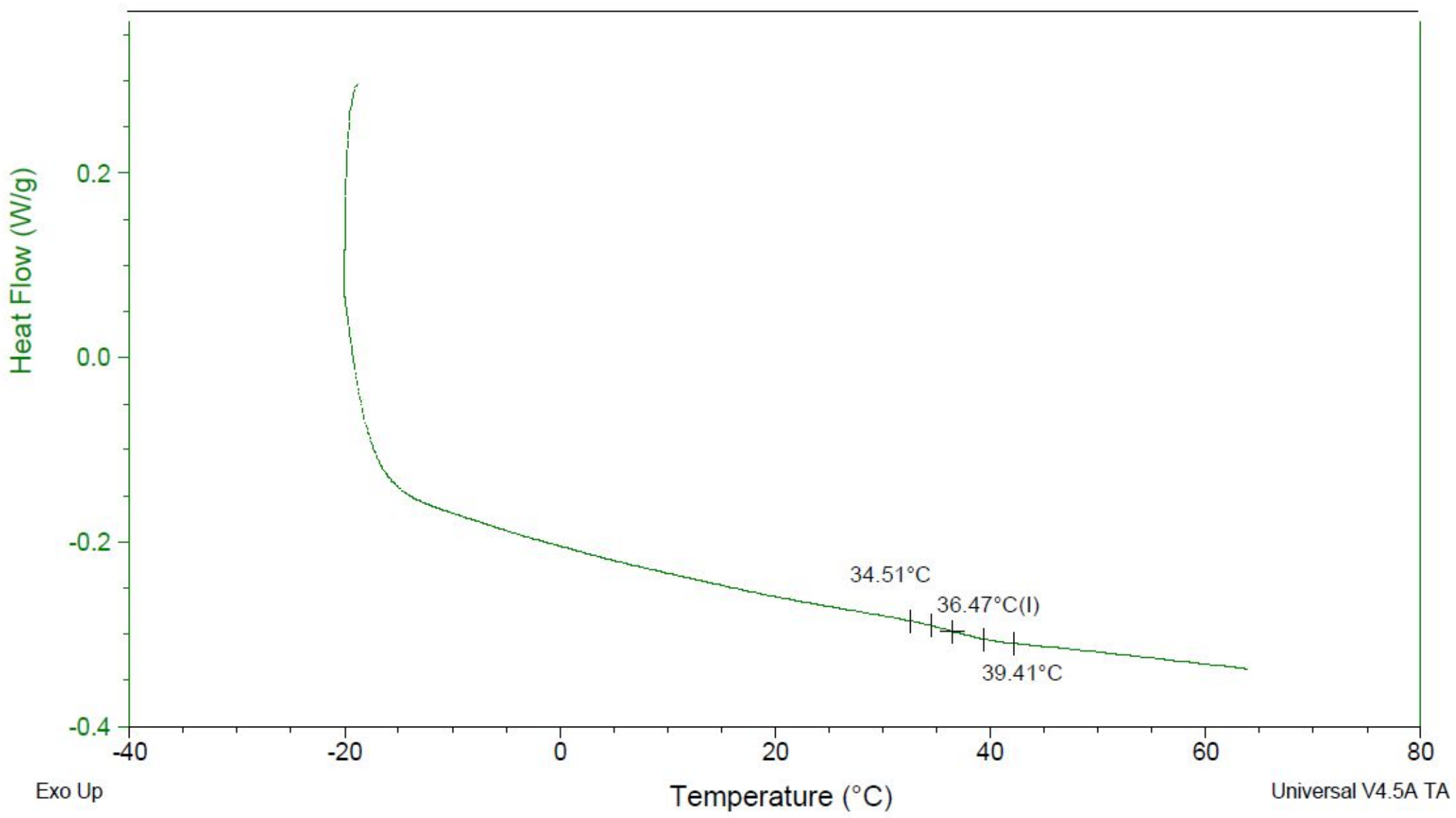

Figure S72. DSC trace of Gly_90CADC_PEI

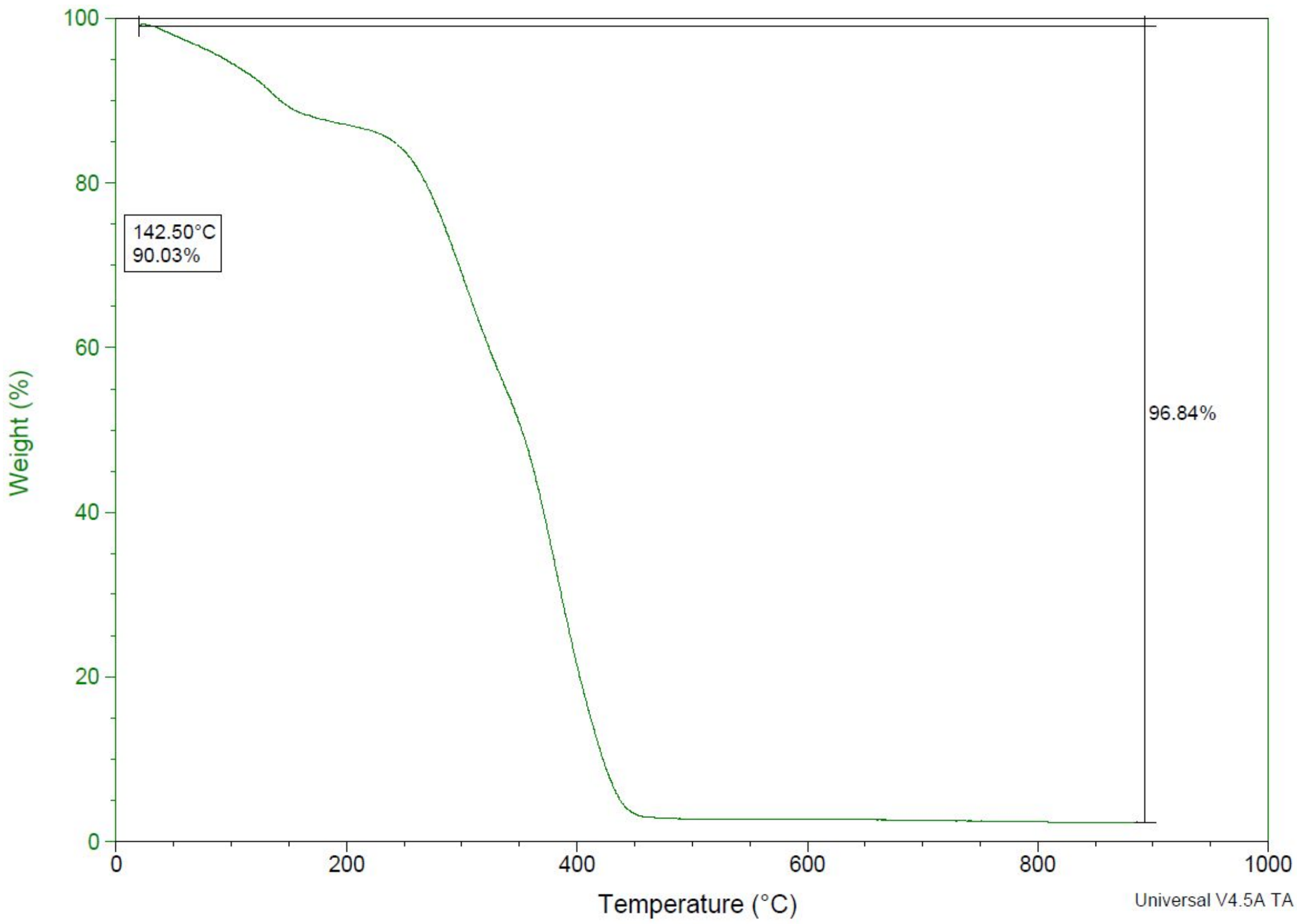

Figure S73. TGA graph of CADC_PEI

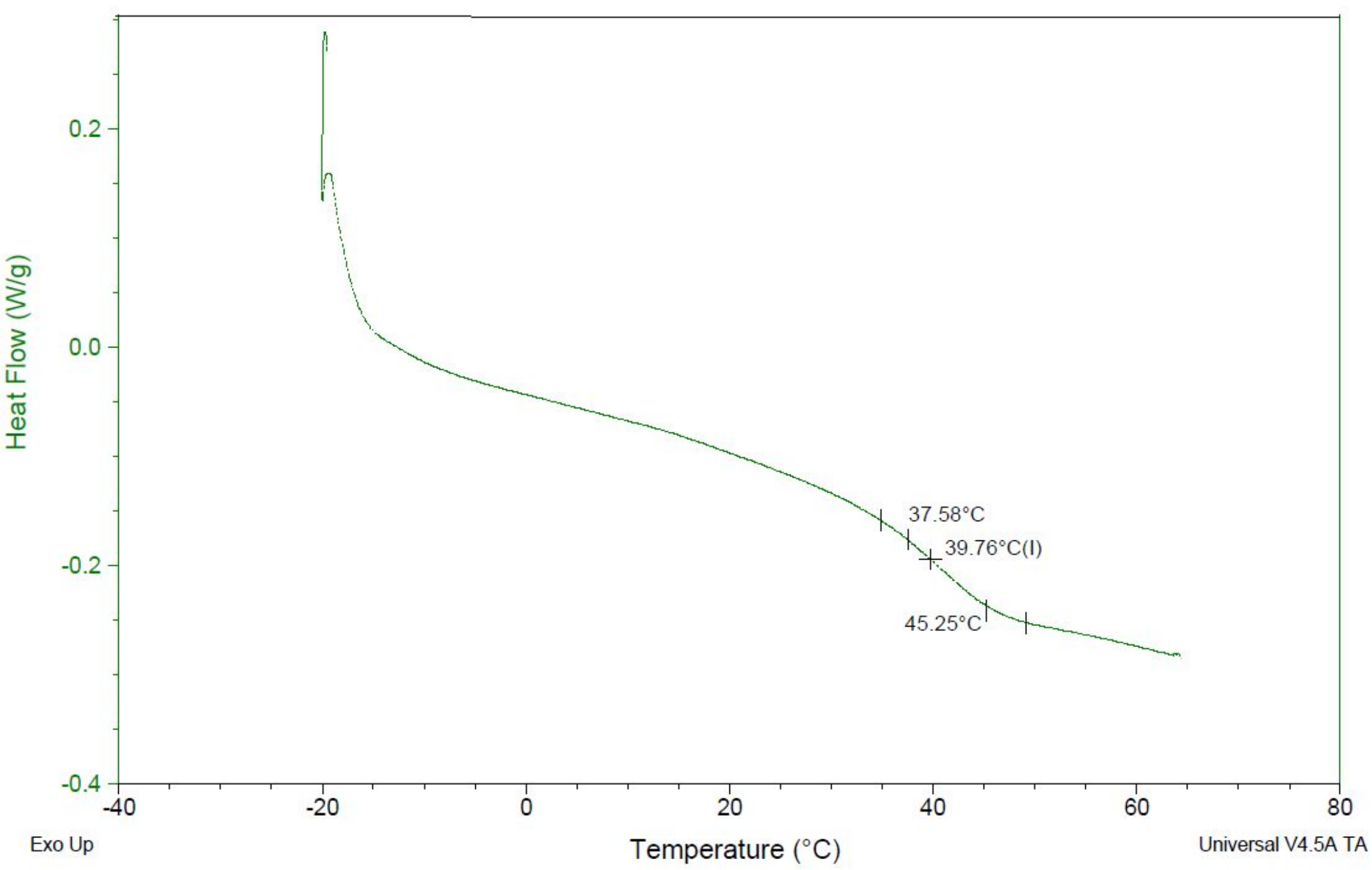

Figure S74. DSC trace of CADC_PEI

S60. DMA graphs of Gly_CADC_PEI thermosetting materials

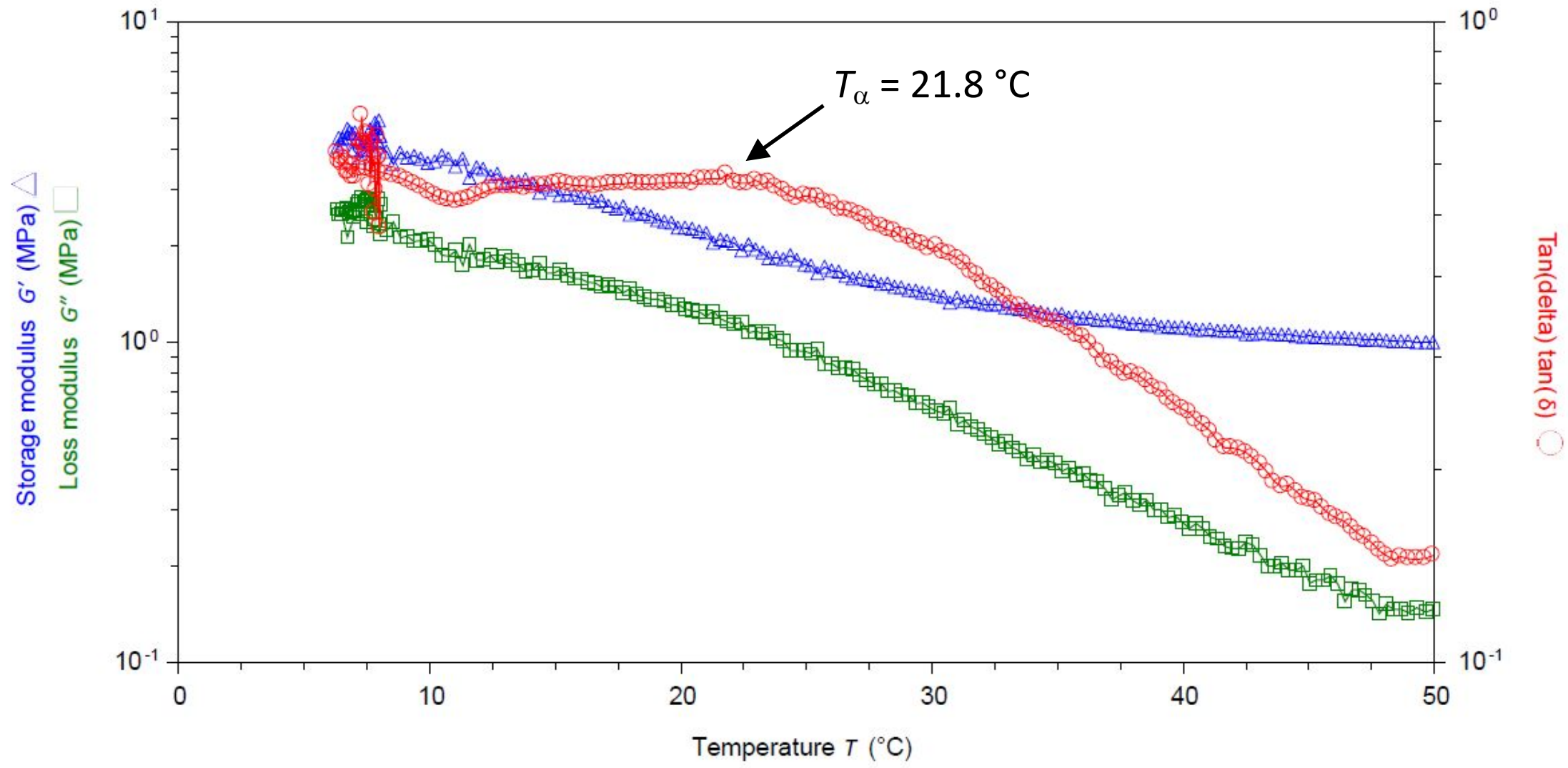

Figure S75. DMA graph of Gly_30CADC_PEI

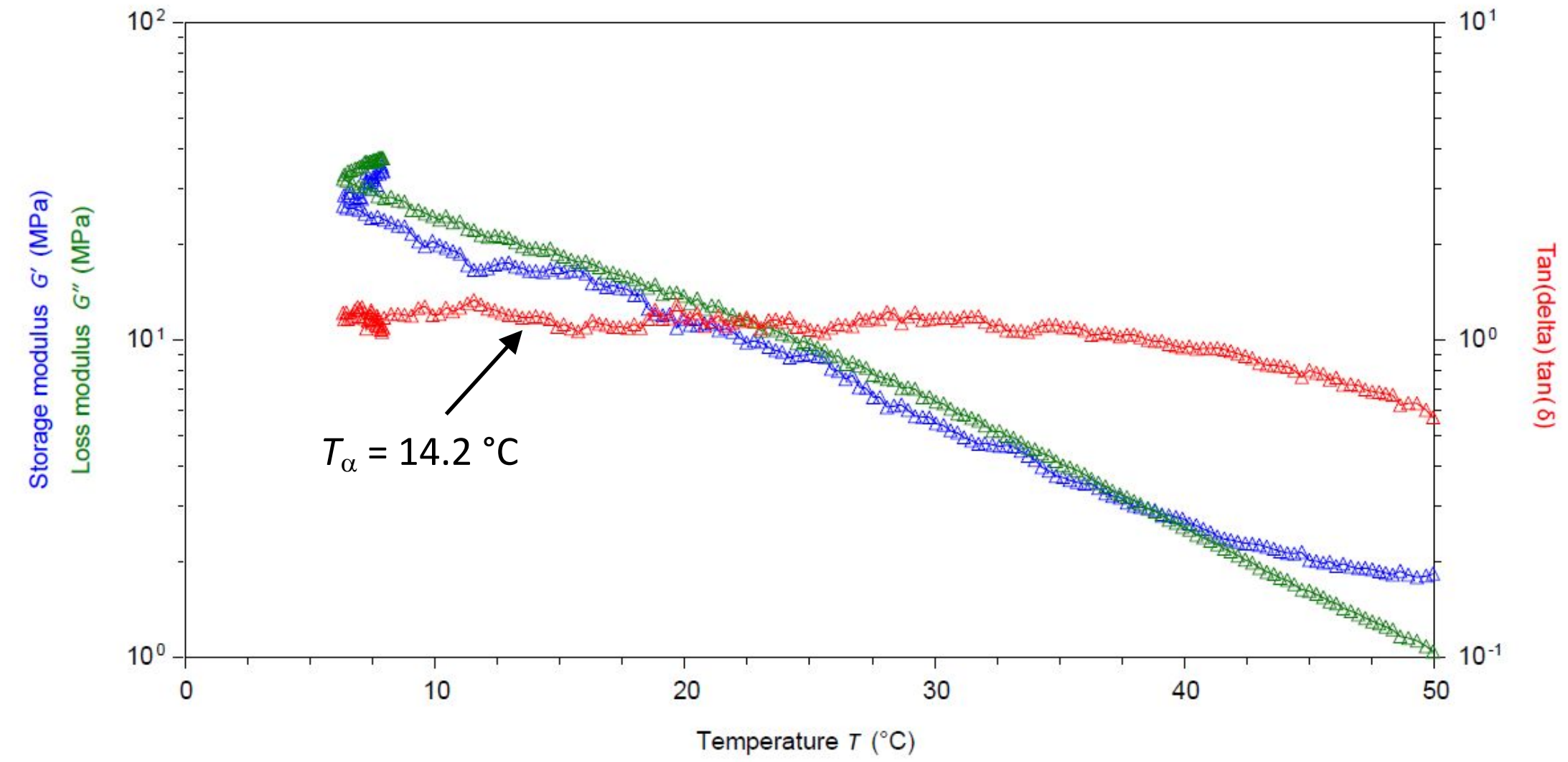

Figure S76. DMA graph of Gly_50CADC_PEI

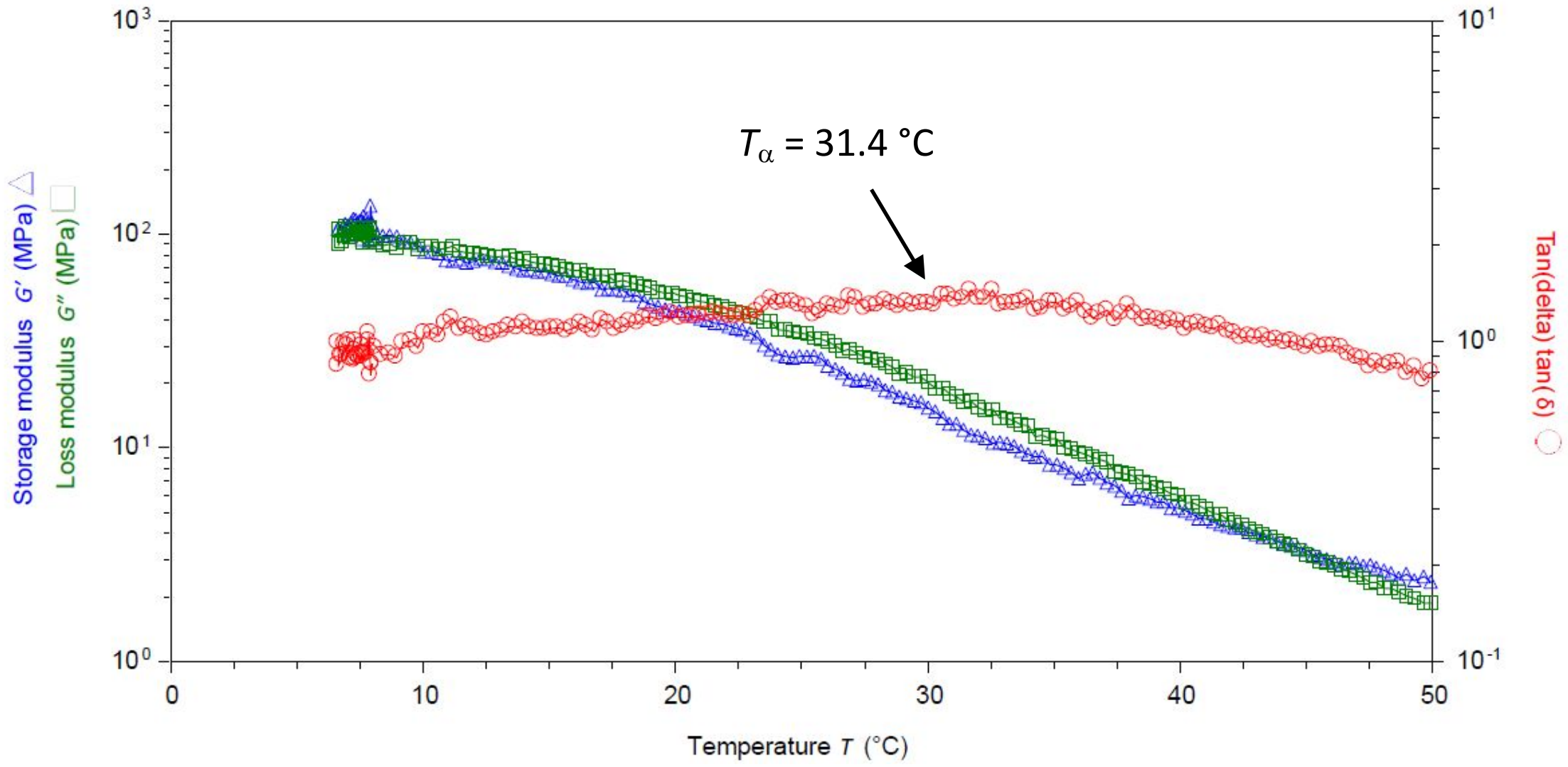

Figure S77. DMA graph of Gly_70CADC_PEI

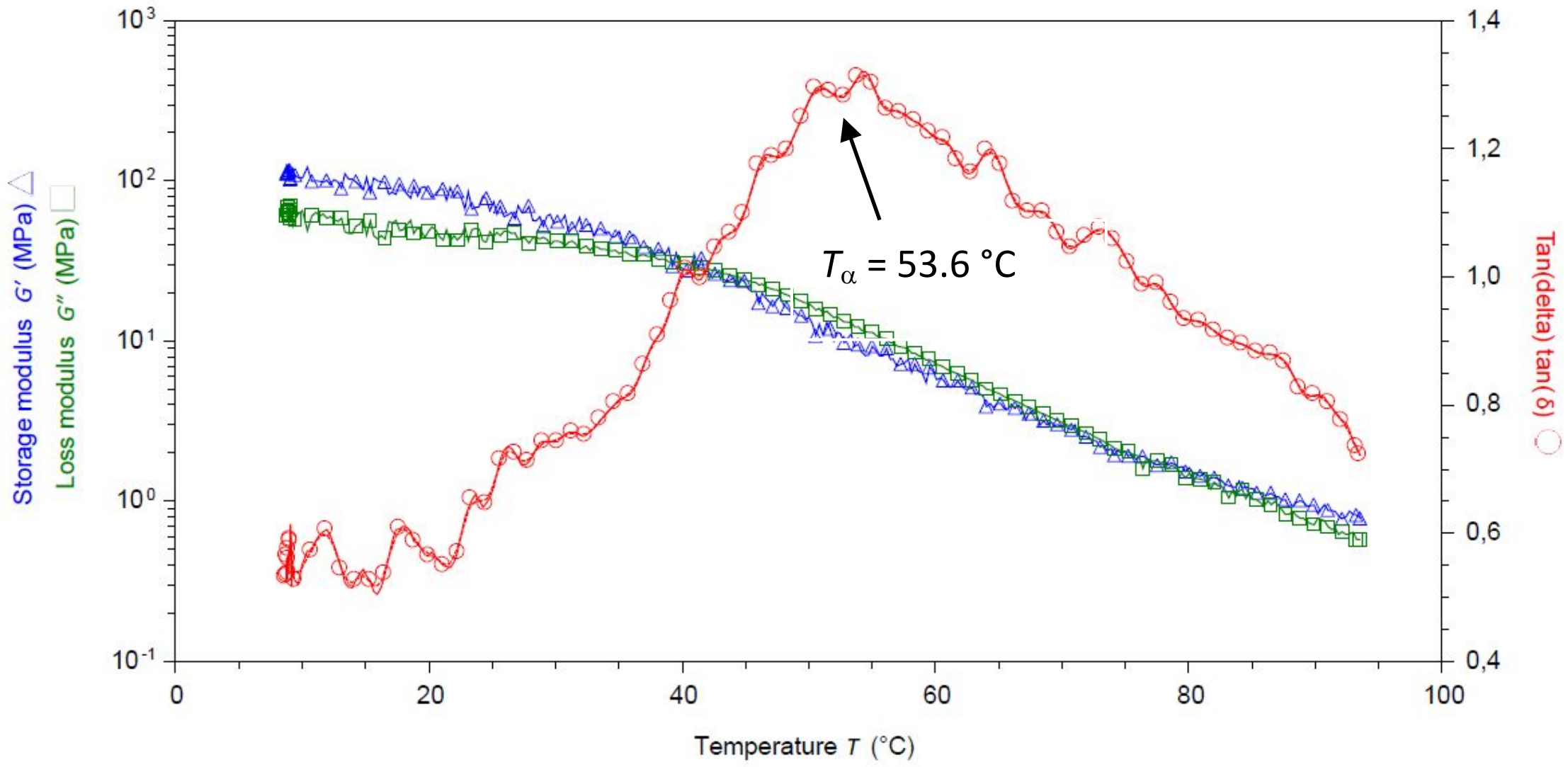

Figure S78. DMA graph of Gly_90CADC_PEI

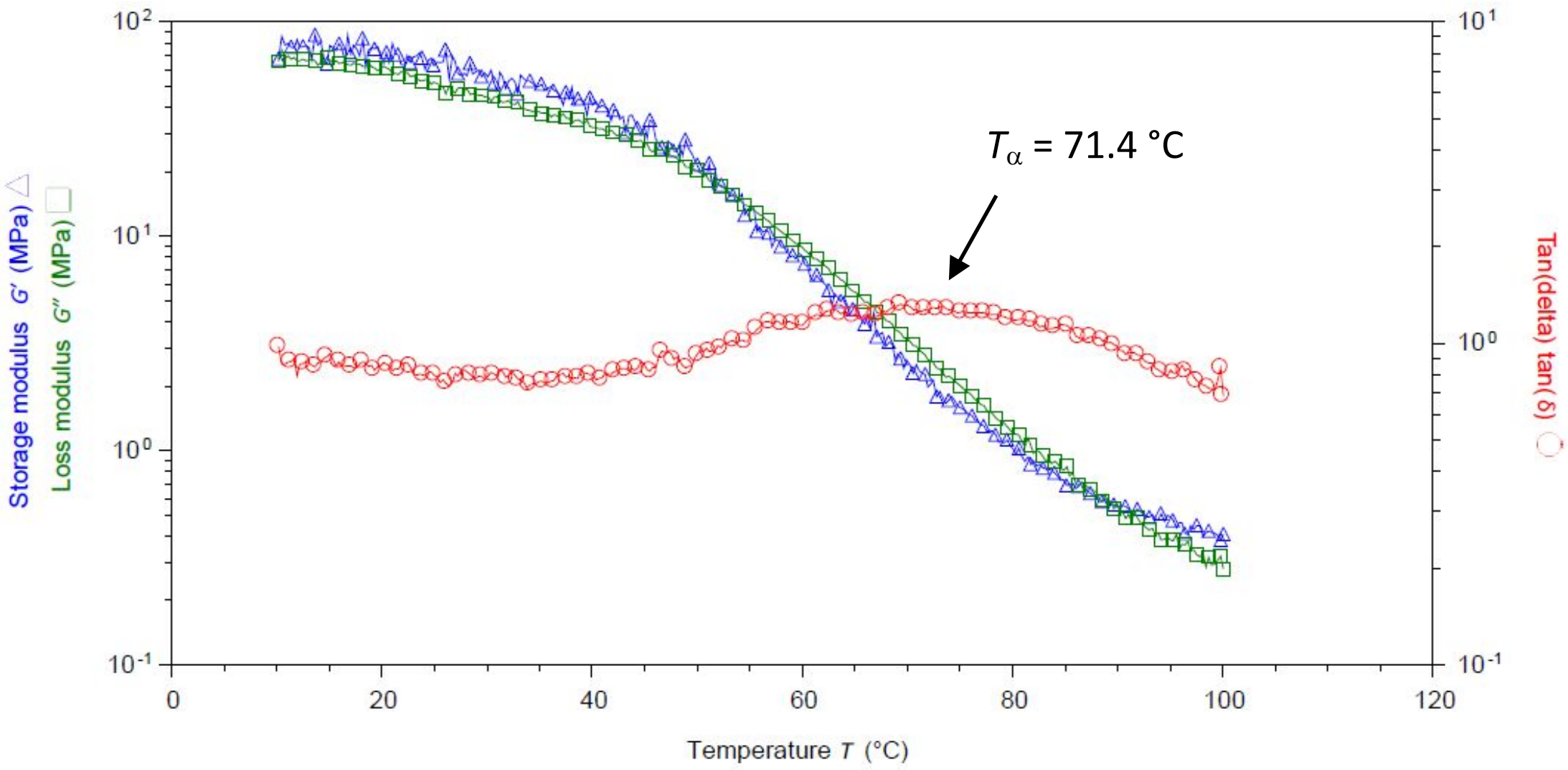

Figure S79. DMA graph of CADC_PEI

S63. Swelling tests results of Gly_CADC_PEI thermosetting materials

SAMPLES	1	2	3
**Initial mass (g)**	0.105	0.1087	0.1125
**Mass after 13 h**	0.1953	0.2146	0.214
**Mass after 24 h**	0.1957	0.2149	0.2148
**Mass after 38 h**	0.1958	0.215	0.2167
**EWC (%)**	86.4	97.8	92.6

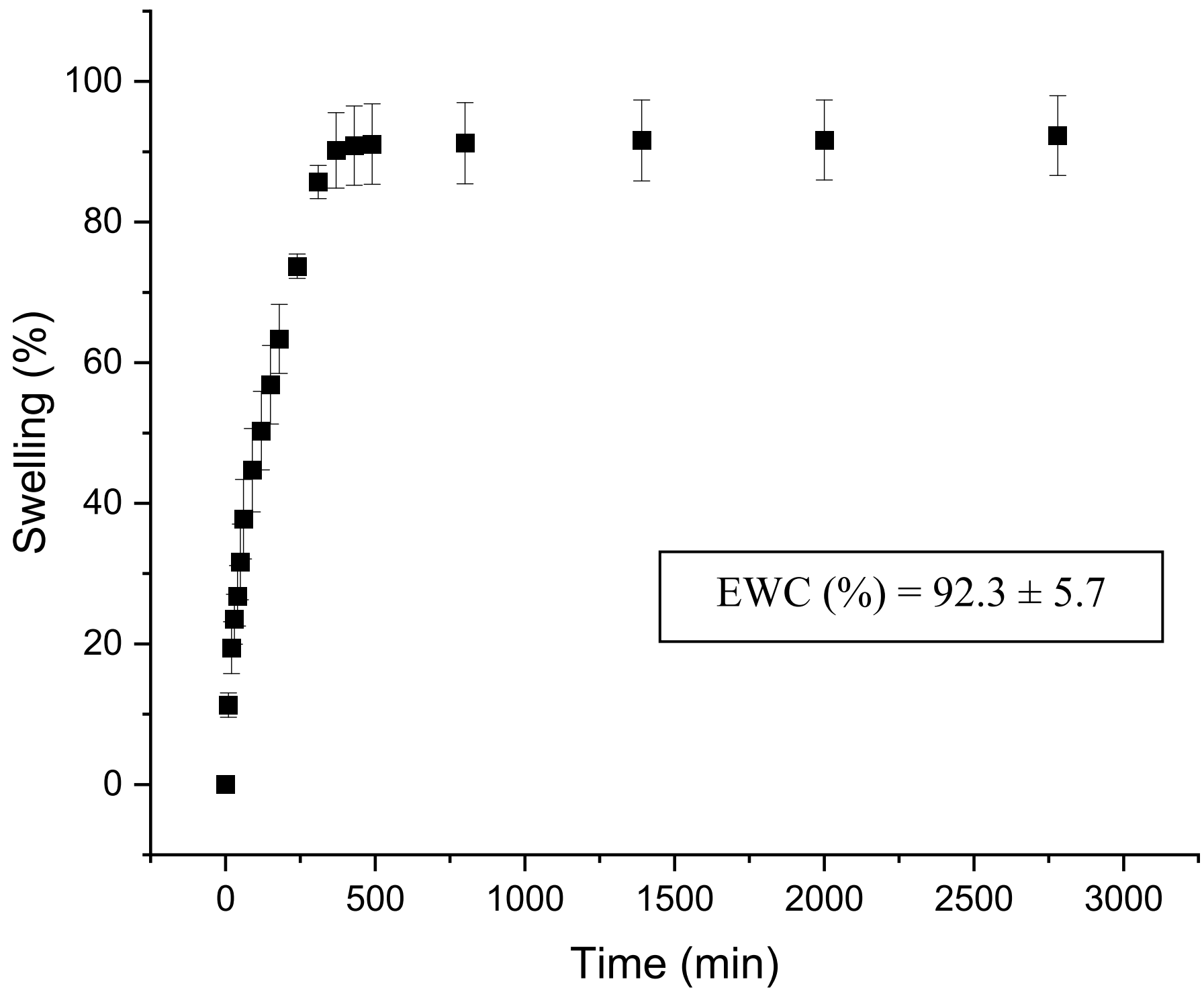

Figure S80. Swelling graph of Gly_30CADC_PEI

SAMPLES	1	2	3
**Initial mass (g)**	0.1003	0.1028	0.1089
**Mass after 14 h**	0.1815	0.188	0.1893
**Mass after 33 h**	0.1823	0.1902	0.1907
**Mass after 45 h**	0.1827	0.191	0.191
**EWC (%)**	82.1	85.8	75.4

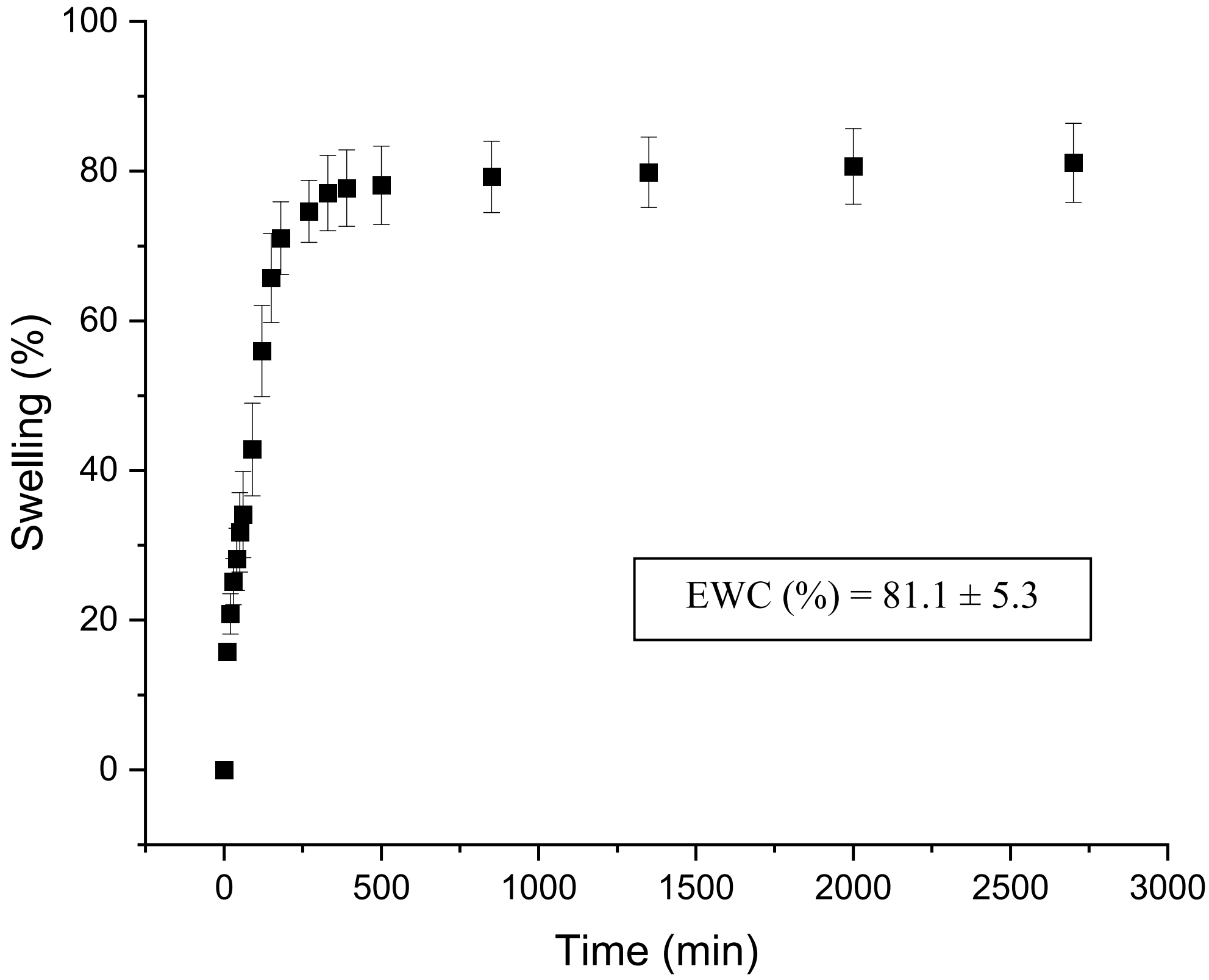

Figure S81. Swelling graph of Gly_50CADC_PEI

SAMPLES	1	2	3
**Initial mass (g)**	0.1140	0.1114	0.1088
**Mass after 6 h**	0.1940	0.1828	0.1874
**Mass after 24 h**	0.1960	0.184	0.1879
**Mass after 38 h**	0.19650	0.1849	0.1885
**EWC (%)**	70.5	66.0	73.2

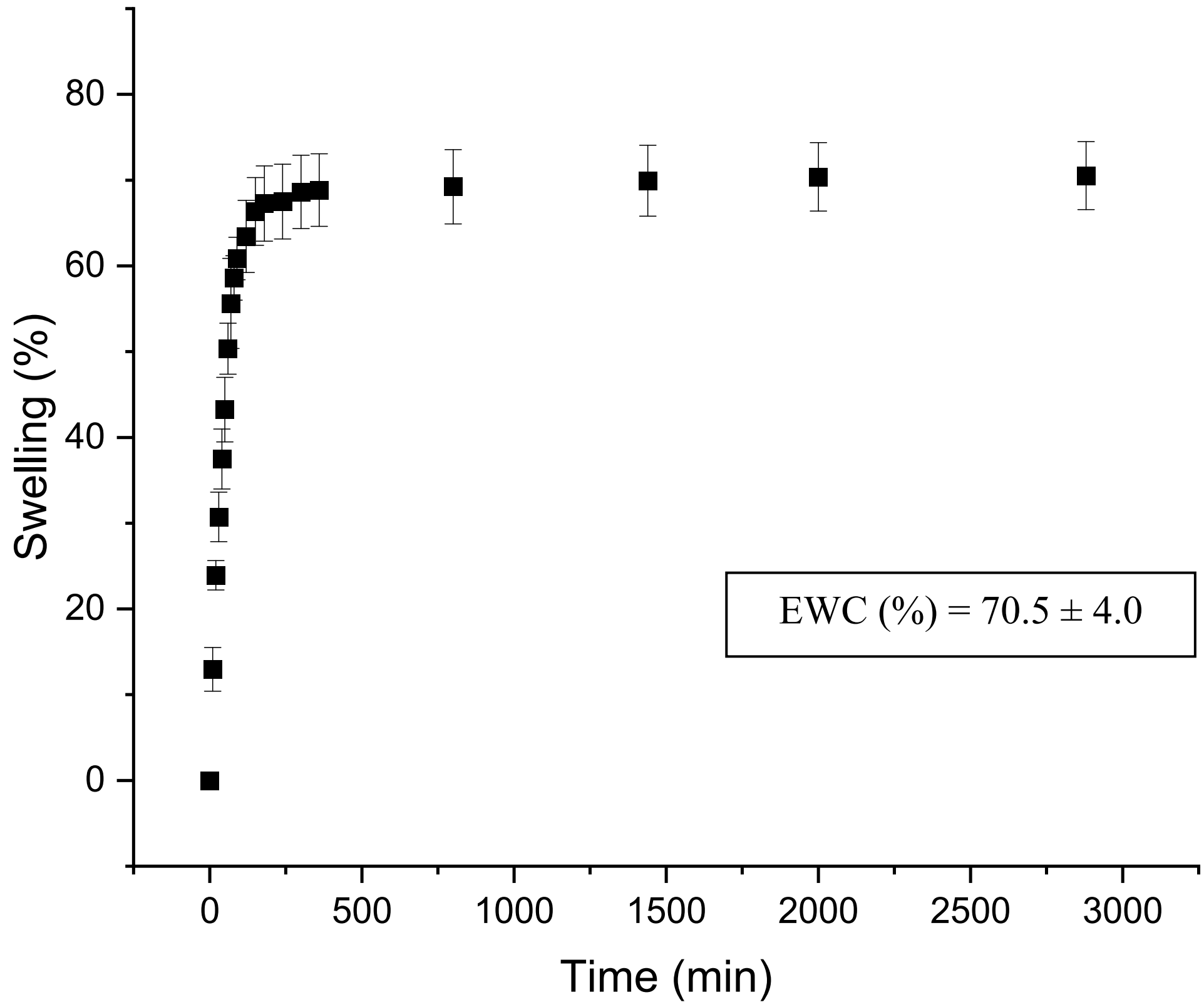

Figure S82. Swelling graph of Gly_70CADC_PEI

S66. References

(1) de la Cruz-Martínez, F.; Martínez de Sarasa Buchaca, M.; Martínez, J.; Fernández-Baeza, J.; Sánchez-Barba, L. F.; Rodríguez-Diéguez, A.; Castro-Osma, J. A.; Lara-Sánchez, A. Synthesis of Bio-Derived Cyclic Carbonates from Renewable Resources. *ACS Sust. Chem. Eng.* **2019**, *7* (24), 20126−20138.

(2) Martínez de Sarasa Buchaca, M.; de la Cruz-Martínez, F.; Francés-Poveda, E.; Fernández-Baeza, J.; Sánchez-Barba, L. F.; Garcés, A.; Castro-Osma, J. A.; Lara-Sánchez, A. Synthesis of Nonisocyanate Poly (hydroxy) urethanes from Bis(cyclic carbonates) and Polyamines. *Polymers* **2022**, *14* (13), 2719.
